# Transforming growth factor-β challenge alters the *N*-, *O*-, and glycosphingolipid glycomes in PaTu-S pancreatic adenocarcinoma cells

**DOI:** 10.1016/j.jbc.2022.101717

**Published:** 2022-02-11

**Authors:** Jing Zhang, Zejian Zhang, Stephanie Holst, Constantin Blöchl, Katarina Madunic, Manfred Wuhrer, Peter ten Dijke, Tao Zhang

**Affiliations:** 1 Oncode Institute and Department of Cell Chemical Biology, Leiden University Medical Center, Leiden, The Netherlands; 2 Center for Proteomics and Metabolomics, Leiden University Medical Center, Leiden, The Netherlands; 3 Department of Biosciences, University of Salzburg, Salzburg, Austria

**Keywords:** pancreatic ductal adenocarcinoma, transforming growth factor-β, glycosphingolipids, *N*-glycosylation, *O*-glycosylation, SRY-related HMD box 4, CCN2, cellular communication network factor 2, CDH, gene cadherin, CDKN2A, cyclin-dependent kinase inhibitor 2A, DMEM, Dulbecco's modified Eagle's medium, EGCase I, endoglycoceramidase I, EMT, epithelial-to-mesenchymal transition, ESI, electrospray ionization, FUT8, fucosyltransferase 8, GALNT3, polypeptide *N*-acetylgalactosaminyltransferase 3, GSL, glycosphingolipid, HEK, human embryonic kidney, IB, immunoblotting, IF, immunofluorescence, MGAT, *N*-acetylglucosaminyltransferase, PaTu-S, PaTu-8955S, PDAC, pancreatic ductal adenocarcinoma, PGC, porous graphitized carbon, PTHLH, parathyroid hormone–like hormone, PVDF, polyvinylidene difluoride, RP, reverse phase, SERPINE1, serpin family E member 1, sgRNA, single-guide RNA, SMAD4, SMA-related and MAD-related protein 4, SNAI2, SNAIL family transcriptional repressor 2, SOX4, sex-determining region SRY-related HMG high-mobility group box 4, SPE, solid-phase extraction, ST3GAL, ST3 β-galactoside alpha-2,3-sialyltransferase, ST6GAL1, ST6 β-galactoside α-2,6-sialyltransferase 1, TβRI, TGF-β type I receptor, TβRII, TGF-β type II receptor, TGF-β, transforming growth factor-β, TP53, tumor protein p53, VIM, vimentin

## Abstract

Pancreatic ductal adenocarcinoma (PDAC) is characterized by poor prognosis and high mortality. Transforming growth factor-β (TGF-β) plays a key role in PDAC tumor progression, which is often associated with aberrant glycosylation. However, how PDAC cells respond to TGF-β and the role of glycosylation therein is not well known. Here, we investigated the TGF-β-mediated response and glycosylation changes in the PaTu-8955S (PaTu-S) cell line deficient in SMA-related and MAD-related protein 4 (SMAD4), a signal transducer of the TGF-β signaling. PaTu-S cells responded to TGF-β by upregulating SMAD2 phosphorylation and target gene expression. We found that TGF-β induced expression of the mesenchymal marker N-cadherin but did not significantly affect epithelial marker E-cadherin expression. We also examined differences in *N*-glycans, *O*-glycans, and glycosphingolipid-linked glycans in PaTu-S cells upon TGF-β stimulation. TGF-β treatment primarily induced *N*-glycome aberrations involving elevated levels of branching, core fucosylation, and sialylation in PaTu-S cells, in agreement with TGF-β-induced changes in the expression of glycosylation-associated genes. In addition, we observed differences in *O* glycosylation and glycosphingolipid glycosylation profiles after TGF-β treatment, including lower levels of sialylated Tn antigen and neoexpression of globosides. Furthermore, the expression of transcription factor sex-determining region Y-related high-mobility group box 4 was upregulated upon TGF-β stimulation, and its depletion blocked TGF-β-induced *N*-glycomic changes. Thus, TGF-β-induced *N*-glycosylation changes can occur in a sex-determining region Y-related high-mobility group box 4–dependent and SMAD4-independent manner in the pancreatic PaTu-S cancer cell line. Our results open up avenues to study the relevance of glycosylation in TGF-β signaling in SMAD4-inactivated PDAC.

Pancreatic ductal adenocarcinoma (PDAC) is one of the most lethal tumors in the world. It is characterized by a poor prognosis and a failure to respond to therapy (1). Genomic analysis of PDAC revealed that the most frequent genetic alterations include the activation of oncogene *KRAS* and inactivation of the tumor suppressors tumor protein p53 (*TP53*), SMA-related and MAD-related protein 4 (SMAD4), and cyclin-dependent kinase inhibitor 2A (*CDKN2A*) (2, 3, 4). The accumulation of these genetic mutations contributes to the stepwise progression of PDAC. *KRAS* mutations occur in the early stage of PDAC (5), whereas mutations of *TP53*, *SMAD4*, and *CDKN2A* arise in advanced pancreatic intraepithelial neoplasias and invasive pancreatic adenocarcinomas (6, 7, 8). These common genetic abnormalities can profoundly perturb protein interactions and specific signaling pathways related to cell survival (9, 10), DNA damage repair (11), angiogenesis (12, 13), invasion (14), metastasis (15), and immune responses (16, 17).

The transforming growth factor-β (TGF-β) signaling pathway is involved in many cellular processes, such as cell proliferation, apoptosis, migration, invasion, and immune evasion, contributing to various diseases, including cancer (18, 19). TGF-β is a secreted cytokine for which cellular responses are initiated by binding to specific cell surface TGF-β type I and type II receptors, that is, TβRI and TβRII. Upon TGF-β interaction with TβRII, TβRI is recruited, and a heteromeric complex is formed (20, 21). Thereafter, the TβRII kinase phosphorylates the serine and threonine residues of TβRI, and thereby the extracellular signal is transduced across the plasma membrane (22). Subsequently, intracellular signaling by TβRI proceeds *via* the phosphorylation of SMAD proteins, that is, SMAD2 and SMAD3. Then, phosphorylated SMAD2/3 can form heteromeric complexes with the common SMAD mediator, that is, SMAD4, which translocates into the nucleus to regulate the transcription of target genes (23). SMAD4 is a critical mediator of TGF-β-induced growth arrest (24, 25) and apoptosis (26), which results in its role as a tumor suppressor at the early stages of cancer progression. However, the tumor-suppressive action of TGF-β–SMAD4 signaling is lost in nearly half of PDACs because of the inactivation of SMAD4 (27). The SMAD4 gene deletion, frameshift mutation, or single point mutation can lead to the deficiency of functional SMAD4 protein, which further prevents or disrupts transduction of the canonical TGF-β–SMAD4 signaling pathway. Phosphorylated TGF-β receptors can activate SMAD2/3-dependent and SMAD4-independent pathways; in that case, receptor-regulated SMADs can make complexes with sex-determining region Y-related high-mobility group box 4 (SOX4) or thyroid transcription factor-1 (also known as NKX2-1), which compete with SMAD4 for interaction with SMAD3 (28, 29). The SMAD3–thyroid transcription factor-1 and SMAD3–SOX4 complexes accumulate in the nucleus, regulate the expression of pro-oncogenic TGF-β target genes, and induce tumorigenesis (28, 29, 30, 31). In addition, activated TβRI can signal *via* non-SMAD signaling pathways, such as the mitogen-activated protein kinase signaling cascade, including the extracellular signal–regulated kinase 1/2, c-Jun amino terminal kinase, p38, and other pathways like IκB kinase, PI3K–AKT signaling, as well as the Rho-like GTPase activity (32, 33, 34, 35, 36).

The SMAD-dependent and non-SMAD signaling pathways are involved in multiple cellular processes, including TGF-β-induced epithelial-to-mesenchymal transition (EMT) (37, 38). EMT is a crucial step toward cell metastasis, during which the epithelial cells lose their polarity and cell–cell contacts and gain mesenchymal abilities such as enhanced migratory and invasive abilities (39). EMT can be associated with morphological or phenotypic changes accompanied by a switch in the expression of EMT marker proteins, a loss of epithelial markers, such as E-cadherin, claudin, and an increase of mesenchymal markers, including N-cadherin, vimentin (VIM), SNAIL, and SLUG (40, 41). EMT is a dynamic and reversible process, and cells can undergo a complete EMT or, more commonly, take on a hybrid E/M status named “partial EMT”. The new term epithelial–mesenchymal plasticity is used to describe the cells undergoing intermediate E/M phenotypic states (42). Both complete EMT and partial EMT exist in PDACs, and the latter is speculated to be involved in collective cell migration and result in an enhanced metastasis rate through the formation of clusters of circulating tumor cells (43). Recent studies have provided several mechanistic explanations for the TGF-β signaling in PDACs; for example, the restoration of SMAD4 in PDAC cells leads to a TGF-β-induced lethal EMT by triggering cell apoptosis *via* SOX4, indicating that EMT switches SOX4 function from protumorigenic to proapoptotic (30).

Glycosylation of cellular proteins and lipids is a common post-translational modification in cells, which affects many cellular processes, such as cell adhesion, proliferation, angiogenesis, migration, and invasion (44, 45). Dysregulated glycosylation is associated with TGF-β signaling and TGF-β-induced EMT in various cancers (46) by affecting the secretion, bioavailability of TGF-β (47), TβRII localization in cells, and interaction with TGF-β (48). Moreover, specific glycan structures and glycogenes involved in the biosynthesis of *N*-glycans, *O*-glycans, and glycosphingolipid (GSL)-linked glycans always increase or decrease during TGF-β-induced EMT in various cancers (49), such as lung cancer (50) and breast cancer (49, 51). During PDAC progression, aberrant glycosylation is highly correlated with several pathological processes (4, 52). Several reports have demonstrated a number of glycosylation changes, including increased fucosylation and sialylation in pancreatic cancer progression in PDACs (53, 54). The glycosylation changes of proteins, such as mucin 5AC, insulin-like growth factor binding protein (IGFBP3), and galectin-3-binding protein (LGALS3BP), are involved in various signaling pathways, including TGF-β, tumor necrosis factor, and NF-κB pathways (55, 56). In addition, the polypeptide *N*-acetylgalactosaminyltransferase 3 (GALNT3), one of the enzymes that catalyze the initial step in *O*-linked oligosaccharide biosynthesis, can promote the growth of pancreatic cells (57). Moreover, some aberrations in glycosylation strongly influence the properties of tumor-associated extracellular matrix and contribute to increased cell migration and invasion (58, 59, 60, 61). Indeed, the glycan-based cancer antigen 19 to 9 (sialyl Lewis A) has been recognized as the hallmark for the diagnosis and early detection of pancreatic cancer (62, 63, 64, 65). A better understanding of the aberrant glycosylation of PDAC induced by TGF-β may aid the identification of potential therapeutic targets and biomarkers. However, the underlying molecular mechanisms of TGF-β signaling in SMAD4-deficient PDAC cells and their relation to glycosylation are not well understood.

In this study, the PaTu-8988S (PaTu-S) cell line, a human epithelial-like PDAC cell line exhibiting KRAS activation and inactivation of SMAD4 and CDKN2A, was employed to investigate TGF-β responses and resulting glycosylation changes. Upon TGF-β treatment, PaTu-S cells were first analyzed for any effects on gene expression, morphological changes, loss of epithelial traits, and gain of mesenchymal markers. Next, by combining transcriptomic analysis of glycosylation-associated genes with mass spectrometry glycomics, we systematically assessed the TGF-β-induced alterations in the three major classes of cell surface glycans of PaTu-S, namely, *N*-glycans, *O*-glycans, and GSL-linked glycans. Furthermore, we investigated the critical role of SOX4 in TGF-β signaling and TGF-β-induced glycosylation in PaTu-S cells. This provides a stepping-stone for further studies on how glycosylation alterations contribute to TGF-β-mediated tumorigenesis of PDAC.

## Results

### TGF-β-induced responses in the PaTu-S cell line

**Figure 1:**
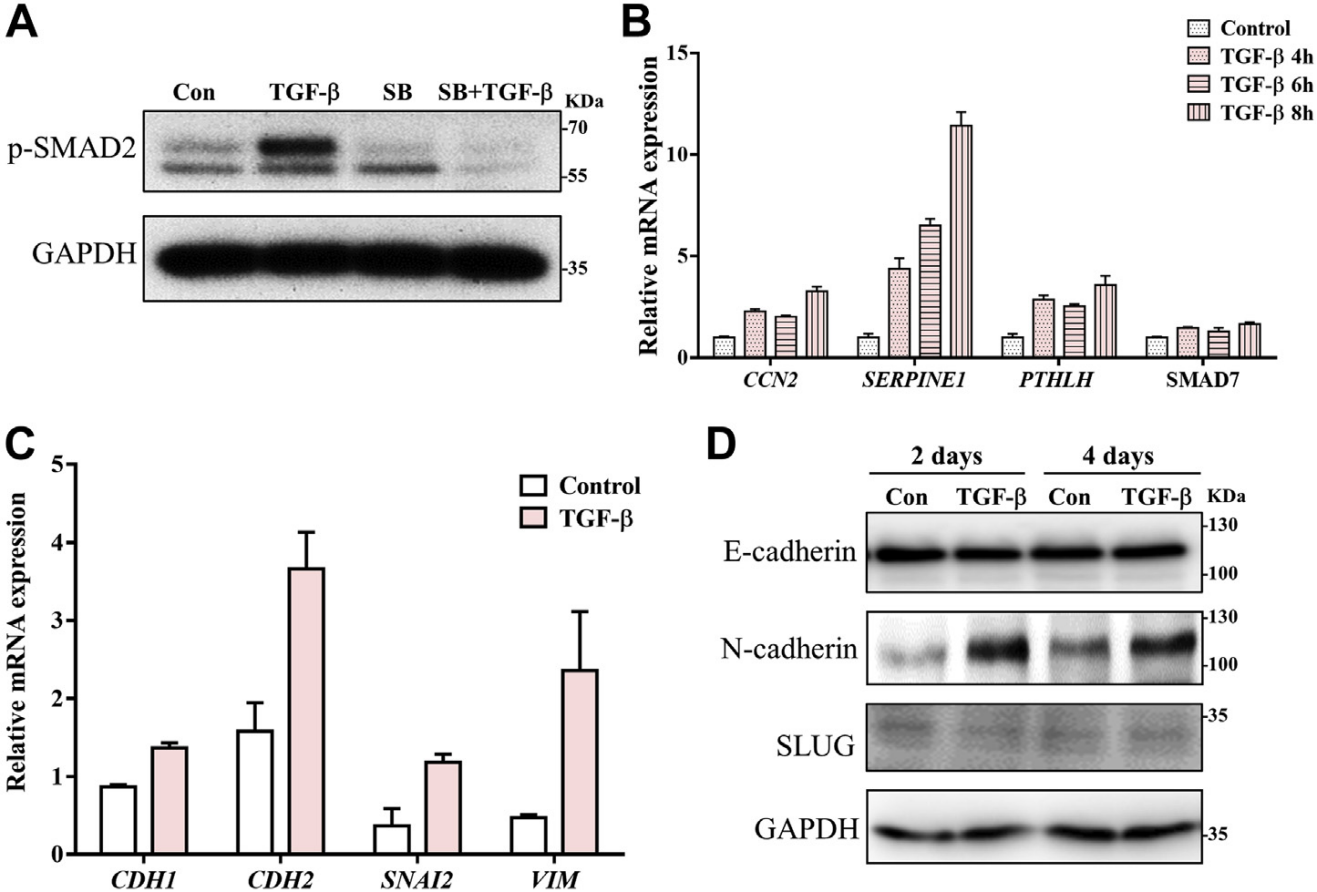
**PaTu-S cell responses to TGF-β.***A*, TGF-β-induced p-SMAD2 levels in PaTu-S cells by Western blot analysis. GAPDH, loading control. Cells were treated solely with vehicle control (Con) or TGF-β for 1 h. SB431542 (SB; 10 μM), a selective small-molecule inhibitor of TGF-β type I receptor, was added for 2 h, 1 h treatment prior to TGF-β and then in combination with TGF-β for 1 h. *B*, quantitative RT–PCR analysis of TGF-β target genes, including *CCN2*, *SERPINE1*, *PTHLH*, and *SMAD7*, in PaTu-S cells treated with vehicle control or TGF-β for 4, 6, and 8 h. *GAPDH* mRNA levels were used for normalization. *C*, quantitative RT–PCR analysis of epithelial and mesenchymal markers, including *CDH1*, *CDH2*, *SNAL2*, and *VIM*, in PaTu-S cells treated with vehicle control or TGF-β for 2 days. *GAPDH* mRNA levels were used for normalization. *D*, E-cadherin, N-cadherin, and SLUG levels by Western blot analysis in PaTu-S cells treated with vehicle control or TGF-β for 2 and 4 days. In latter case, fresh medium containing TGF-β or vehicle control was added after 2 days. GAPDH, loading control. Molecular weight markers are indicated on the *right*. Three independent experiments were performed. Representative results are shown, or the data are expressed as the mean ± SD (n = 3). TGF-β was applied at a final concentration of 2.5 ng/ml. p-SMAD2, phosphorylated SMA-related and MAD-related protein 4; PaTu-S, PaTu-8955S; TGF-β, transforming growth factor-β.

To investigate whether the PaTu-S cell line, which lacks SMAD4, is responsive to TGF-β treatment (Fig. S1*A*), we performed Western blot analysis of phosphorylated SMAD2 levels of PaTu-S cells without and with TGF-β challenge. In PaTu-S cells, SMAD2 phosphorylation was significantly upregulated upon TGF-β stimulation for 1 h, which was blocked by treatment with TβRI kinase inhibitor SB431542 (Fig. 1*A*). The expression of TGF-β target genes, including cellular communication network factor 2 (*CCN2*) (66, 67), serpin family E member 1 (*SERPINE1*) (68, 69), parathyroid hormone–like hormone (*PTHLH*), (70), and *SMAD7* (71), was induced by TGF-β treatment at multiple time points (Fig. 1*B*). These genes can be induced by TGF-β–SMAD and non-SMAD pathways and in cells in which SMAD4 was inactivated (72). In response to TGF-β stimulation for 2 days, the PaTu-S cells showed an upregulation in the expression of both the epithelial marker gene cadherin 1 (*CDH1*, encoding the protein E-cadherin) and the mesenchymal marker genes *CDH2* (encoding the protein N-cadherin), SNAIL family transcriptional repressor 2 (*SNAI2*, encoding the protein SLUG), and *VIM* (encoding the protein VIM) (Fig. 1*C*). At the protein level, the mesenchymal marker N-cadherin was increased after TGF-β stimulation, whereas E-cadherin and SLUG expression levels were not significantly affected (Fig. 1*D*). In addition, no morphological changes of PaTu-S cells were observed after 2 days of TGF-β treatment (Fig. S1*B*) or even longer treatment times (data not shown). The response of PaTu-S cells to TGF-β treatment was further analyzed by immunofluorescence (IF) staining of E-cadherin and filamentous (F)-actin. We observed that TGF-β induced an increase in the formation of lamellipodia and broadened and increased flat membrane protrusions at the leading edge of cells (Fig. S1*C*). However, in response to TGF-β, no significant changes in E-cadherin expression and localization were observed. As an increase of lamellipodia formation has been linked to an increase in cell migration (73), we next examined the TGF-β response of PaTu-S cells using an embryonic zebrafish xenograft extravasation model (Fig. S1, *D* and *E*). The PaTu-S cells were pretreated with TGF-β or vehicle control for 2 days, and thereafter, a similar number of cells were injected into the circulation of zebrafish embryos. After 4 days postinjection, we observed an increased number of invasive cell clusters (more than five cells in one cluster) in the caudal hematopoietic tissue in the TGF-β pretreatment group compared with the nontreated group. Thus, TGF-β pretreatment promoted the extravasation of PaTu-S cells (Fig. S1*D*, see representative images in Fig. S1*E*). Taken together, these results indicate that the PaTu-S cells responded to TGF-β with an upregulation of SMAD2 phosphorylation and target gene expression. In addition, upon TGF-β treatment, an increase in mesenchymal marker expression was observed but without a decrease or change in localization of epithelial markers. Moreover, TGF-β treatment of PaTu-S promoted the lamellipodia formation *in vitro* and cell extravasation *in vivo*.

### Differential glycosylation of PaTu-S cells following TGF-β stimulation

Changes in glycosylation of lipids and cell surface proteins control various cellular pathways, including TGF-β signaling. Perturbations of these pathways are associated with pathological processes, that is, cancer progression (46). Prompted by our findings regarding the effects of TGF-β on PaTu-S cell behavior, we investigated the differential glycosylation of PaTu-S upon TGF-β treatment. We performed a comprehensive glycomic analysis of *N*-glycans, *O*-glycans, and GSL-linked glycans. Cell pellets were divided into two parts: one for the analysis of *N*-glycans and *O*-glycans (74) and the other for the analysis of GSL-linked glycans. For the analysis of all three glycan classes, we used porous graphitized carbon (PGC) nano-LC–electrospray ionization (ESI)–MS/MS in negative ESI mode, enabling the discrimination between glycan isomers (75, 76). Glycan structures were assigned on the basis of the obtained LC–MS/MS data and general glycobiological knowledge, which are supplied in Fig. S10 (*N*-glycan), Fig. S11 (*O*-glycan), and Fig. S12 (GSL-linked glycan). Relative quantification of each glycan with and without TGF-β treatment was performed and summarized in Table S1 (*N*-glycan), Table S2 (*O*-glycan), and Table S3 (GSL-linked glycan). Glycomic signatures were complemented by analyzing expression levels of glycosylation-associated genes in the cells. Glycan species, traits, or ratios reflecting certain biosynthetic steps were calculated to facilitate the biological interpretation of the data and to relate the MS glycomics data to transcriptomics data.

### Significant differences of *N*-glycosylation in PaTu-S cells with and without TGF-β treatment

**Figure 2:**
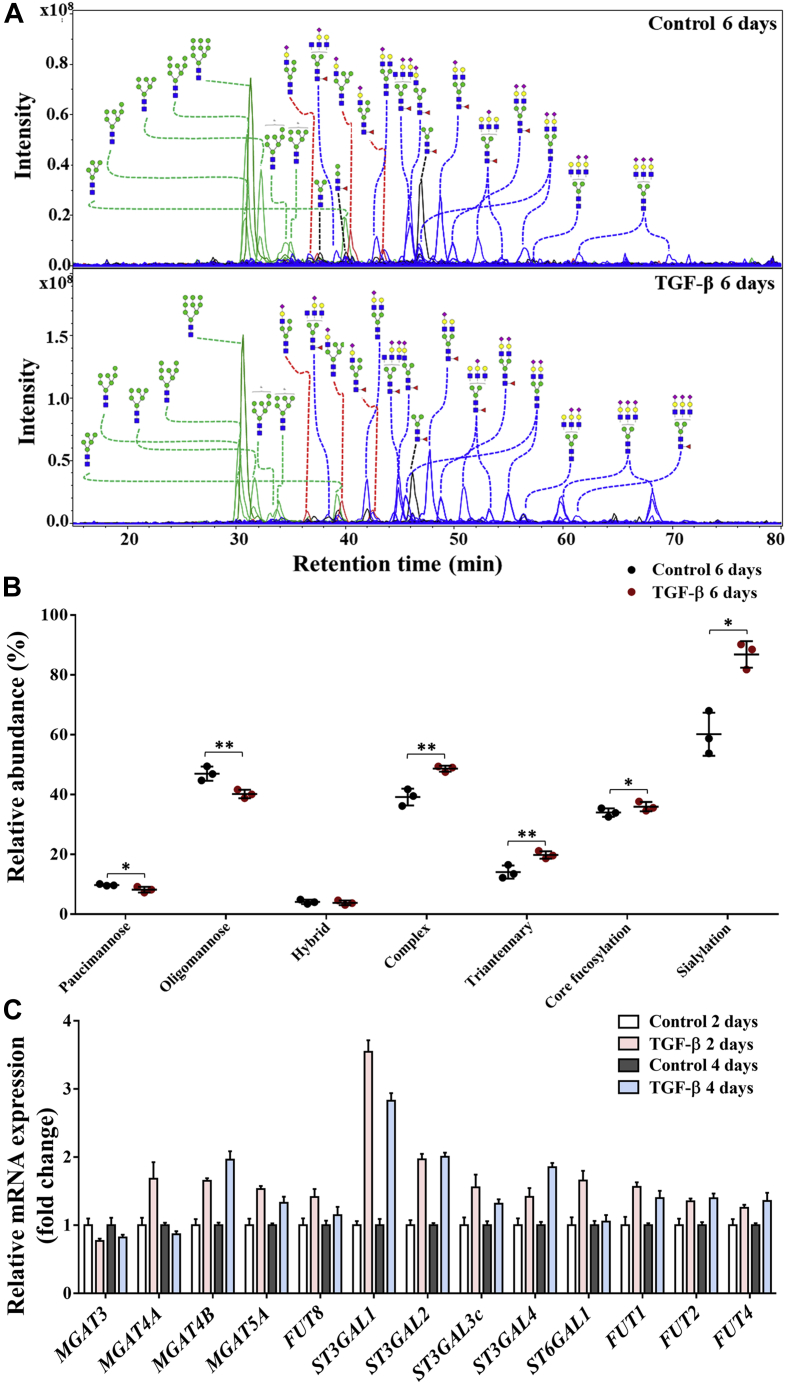
**Differences of *N*-glycosylation in PaTu-S cell line with and without TGF-β treatment.***A*, combined extracted ion chromatograms (EICs) of *N*-glycans were derived from PaTu-S cells treated with vehicle control or TGF-β for 6 days and analyzed by PGC nano-LC–ESI–MS/MS. *Green trace*: oligomannose; *red trace*: hybrid type; *black trace*: paucimannose; and *blue trace*: complex type. *B*, relative abundance of *N*-glycan classes treated with or without TGF-β for 6 days. *C*, quantitative RT–PCR analysis of *N*-glycan-associated glycosyltransferase gene expression levels in PaTu-S cells treated with vehicle control or TGF-β for 2 days or 4 days. *GAPDH* was used for normalization to get the relative mRNA expression, and then gene expression levels in the control groups (2 and 4 days) were used for further normalize the genes in TGF-β-treated groups (2 and 4 days) to get fold-change data. Representative results are shown of three independent experiments, or the data are expressed as the mean ± SD (n = 3). ∗*p* ≤ 0.05, ∗∗*p* ≤ 0.01, and ∗∗∗*p* ≤ 0.001. Fresh medium with TGF-β (2.5 ng/ml) or vehicle control was added every 2 days in all experiments. ESI, electrospray ionization; PaTu-S, PaTu-8955S; PGC, porous graphitized carbon; TGF-β, transforming growth factor-β.

A total of 30 major *N*-glycan isomers spanning 27 different glycan compositions were manually identified from the investigated samples. In agreement with our previous work on *N*-glycan analysis of PaTu-S cell lines based on MALDI-TOF MS (77), the *N*-glycome data of PaTu-S were found to span four main *N*-glycan classes with major amounts of oligomannose (∼47%) and complex type *N*-glycans (∼39%) and lesser contributions of paucimannose (∼10%) and hybrid-type *N*-glycans (∼4%) (Fig. 2*A*). More than 39% of the *N*-glycans were fucosylated, and roughly 60% were sialylated. In addition, approximately 12% of oligomannose type *N*-glycans were phosphorylated. The complex *N*-glycans were primarily diantennary (∼65%), with triantennary structures also present in significant amounts (∼15%).

Upon TGF-β treatment, various *N*-glycosylation changes were observed. Complex *N*-glycans were increased and accounted for ∼49% of the *N*-glycome after TGF-β treatment in contrast to ∼39% in controls (Fig. 2*B*). Specifically, triantennary *N*-glycans increased from ∼14% to ∼21% with TGF-β treatment (Fig. 2*B*), which is exemplified by the rather late-eluting, triantennary and trisialylated *N*-glycan species (Fig. 2*A*). These data are in accordance with the significant upregulation of *N*-acetylglucosaminyltransferase (*MGAT*) *4A*, *MGAT4B*, and *MGAT5A* mRNA transcripts, three genes that encode enzymes involved in the synthesis of triantennary *N*-glycans, upon TGF-β treatment for 2 and 4 days (Figs. 2*C* and S2*B*). Notably, sialylation was high in PaTu-S cells after TGF-β treatment (0.9 sialic acid per glycan on average) with a relative abundance at ∼90%, compared with ∼60% under control conditions (Fig. 2*B*), which is in line with the expression patterns of ST3 β-galactoside α-2,3-sialyltransferase (*ST3GAL*)*2*, *ST3GAL3*, *ST3GAL4*, and ST6 β-galactoside α-2,6-sialyltransferase 1 (*ST6GAL1*) (Figs. 2*C* and S2*B*). In addition, a slightly higher level of core fucosylation was observed (Fig. 2*B*), in line with the upregulation of fucosyltransferase 8 (*FUT8*) (Figs. 2*C* and S2*B*). Moreover, TGF-β treatment induced a decrease of oligomannose *N*-glycans from ∼47% to ∼39% in the PaTu-S cell line (Fig. 2*B*). For a complete overview of the glycan quantification data and glycosylation-associated gene expression levels, see Fig. S2.

### Differences of *O*-glycosylation in PaTu-S cells with and without TGF-β treatment

**Figure 3:**
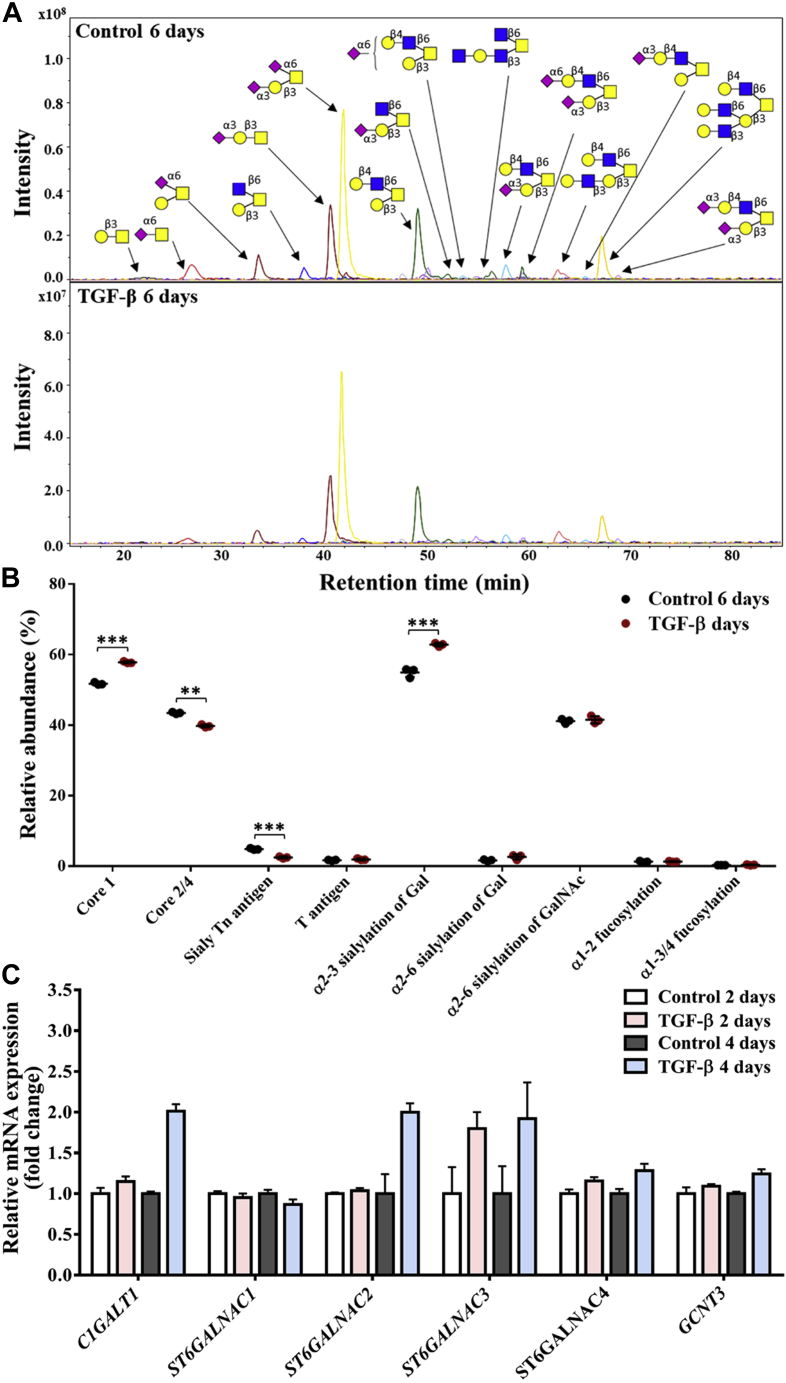
**Differences of *O*-glycosylation in PaTu-S cell line without or with TGF-β treatment.***A*, combined EICs of *O*-glycans were derived from PaTu-S cells treated with vehicle control or TGF-β for 6 days; both conditions show similar *O*-glycan profile. *B*, relative abundance of structural *O*-glycan classes in PaTu-S cells treated with vehicle control or TGF-β for 6 days. *C*, quantitative RT–PCR analysis of *O*-glycosylation-associated gene expression levels in PaTu-S cells treated with vehicle control or TGF-β for 2 days or 4 days. *GAPDH* was used for normalization to get the relative mRNA expression, and then gene expression levels in the control groups (2 and 4 days) were used to further normalize the genes in TGF-β-treated groups (2 and 4 days) to get fold-change data. Representative results are shown of three independent experiments, or the data are expressed as the mean ± SD (n = 3). ∗*p* ≤ 0.05, ∗∗*p* ≤ 0.01, and ∗∗∗*p* ≤ 0.001. Fresh medium containing TGF-β (2.5 ng/ml) or vehicle control was added every 2 days in all experiments. EIC, extracted ion chromatogram; PaTu-S, PaTu-8955S; TGF-β, transforming growth factor-β.

Following the *N*-glycan analysis, we analyzed 22 *O*-glycan species spanning 16 glycan compositions with relative quantification (Fig. S3*A*). The *O*-glycans mainly consisted of core 1 (∼52%) and core 2 structures (∼41%), with low levels of core 4 structures also present (∼2%). A high level of sialylation (more than 97%) was observed in PaTu-S-derived *O*-glycans, whereas roughly 2% of the structures were fucosylated. The *O*-glycan profiles were very similar in TGF-β-treated PaTu-S cells and nontreated cells (Fig. 3*A*), showing a few minor and consistent changes. An increase in core 1 *O*-glycans was observed together with a decrease in core 2/4 *O*-glycans (Fig. 3*B*), probably resulting from the significant upregulation of *C1GALT1* and *GCNT3* (Figs. 3*C* and S3*B*). A lower level of sialyl Tn antigen was detected upon TGF-β treatment (Fig. 3*B*), which is in line with the slightly decreased *ST6GALNAC1* (Figs. 3*C* and S3*B*). For sialylation, a significant increase of α2,3 sialylation of galactose from 55% to 62% was observed (Fig. 3*B*) in accordance with the elevated levels of *ST3GALs* (Figs. 2*C* and S2*B*) and in parallel to the sialylation differences of the *N*-glycome (Fig. 2*B*). In contrast, α2,6 sialylation of galactose, α2,6 sialylation of GalNAc, α1,2 fucosylation of galactose, or α1,3/4 fucosylation was unaffected (Fig. 3*B*). Interestingly, we detected no difference of α2,6 sialylation of GalNAc between PaTu-S cells with and without TGF-β stimulation (Fig. 3*B*), despite the upregulation of α2,6 sialyltransferase-associated genes *ST6GALNACs* (*2*, 3, and 4) (Figs. 3*C* and S3*B*). For a complete overview of the glycan quantification data and glycosylation-associated gene expression levels, see Fig. S3.

### Neoexpression of globosides in GSL glycomics of PaTu-S cells following TGF-β treatment

**Figure 4:**
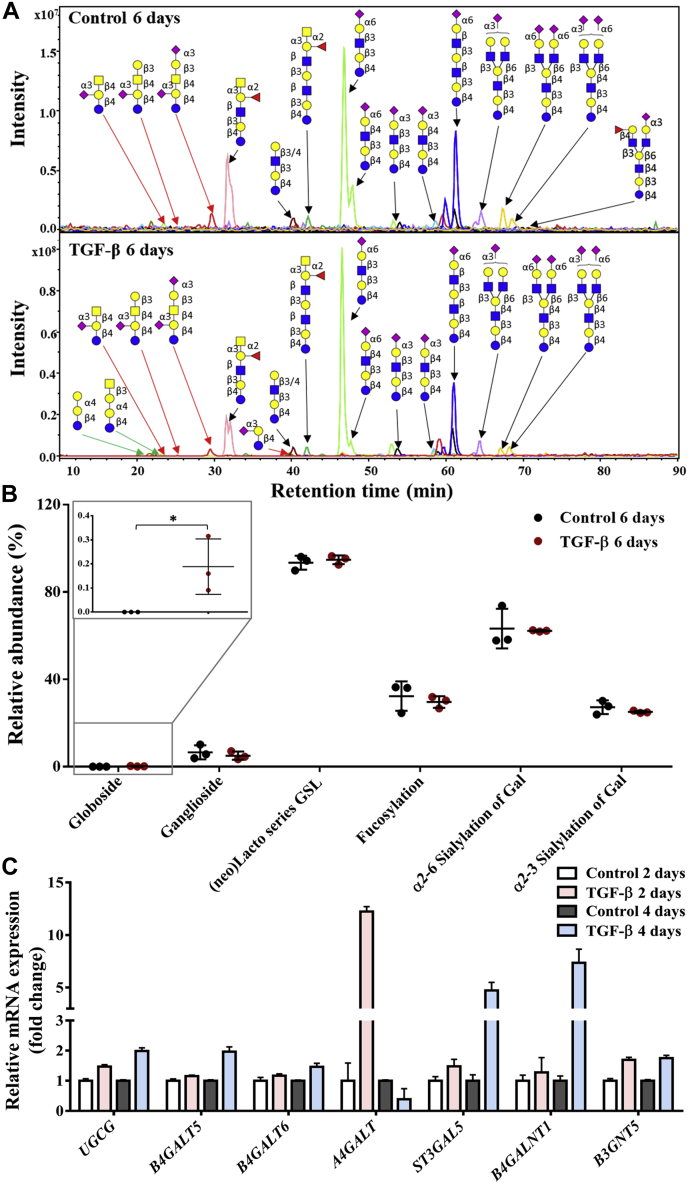
**Differences of glycosphingolipid (GSL)-glycans in PaTu-S cell line without or with TGF-β treatment.***A*, combined EICs of GSL-linked glycans were derived from PaTu-S cells treated with vehicle control or TGF-β for 6 days. *Green arrow*: globosides, *red arrow*: gangliosides, and *black arrow*: (neo)lacto-series GSLs. *B*, relative abundance of structural GSL-linked glycan in PaTu-S cells treated with vehicle control or TGF-β for 6 days. *C*, quantitative RT–PCR analysis of GSL-associated gene expression levels in PaTu-S cells treated with vehicle control or TGF-β for 2 days or 4 days. *GAPDH* was used for normalization to get the relative mRNA expression, and then gene expression levels in the control groups (2 and 4 days) were used to further normalize the genes in TGF-β-treated groups (2 and 4 days) to get fold-change data. Representative results are shown of three independent experiments, or the data are expressed as the mean ± SD (n = 3). ∗*p* ≤ 0.05, ∗∗*p* ≤ 0.01, and ∗∗∗*p* ≤ 0.001. Fresh medium containing TGF-β (2.5 ng/ml) or vehicle control was added every 2 days in all experiments. EIC, extracted ion chromatogram; PaTu-S, PaTu-8955S; TGF-β, transforming growth factor-β.

Next, GSL-linked glycans were analyzed after enzymatic release of the glycan head group using endoglycoceramidase I (EGCase I) from purified GSLs derived from PaTu-S cells. GSL-linked glycan profiles stayed largely unchanged upon TGF-β treatment except for the globoside fraction, which appeared to be specifically induced by the treatment, albeit with overall low expression levels compared with the other GSL classes (Figs. 4, *A* and *B* and S4). Importantly, globosides including Gb3 and Gb4 were specifically present in PaTu-S cells after TGF-β treatment, as there was no globoside detected in the untreated sample (Fig. 4*B*). Consistent with the glycomic data, upon TGF-β treatment, gene transcript data of day 2 also showed a 12-fold increased expression of *A4GALT*, which is the key gene for the biosynthesis of globosides (Figs. 4*C* and S4*B*). Although the expression levels of multiple GSL-associated genes were promoted after TGF-β stimulation for 4 days (Figs. 4*C* and S4*B*), there was no significant difference in gangliosides, (neo)lacto series GSLs, α2,6 sialic acid on galactose, α2,3 sialic acid on galactose, and fucosylation (Fig. 4*B*). For a complete overview of the GSL quantification data and GSL-associated gene expression levels, see Fig. S4.

### SOX4 is required for TGF-β-induced promotion of *N*-glycosylation

**Figure 5:**
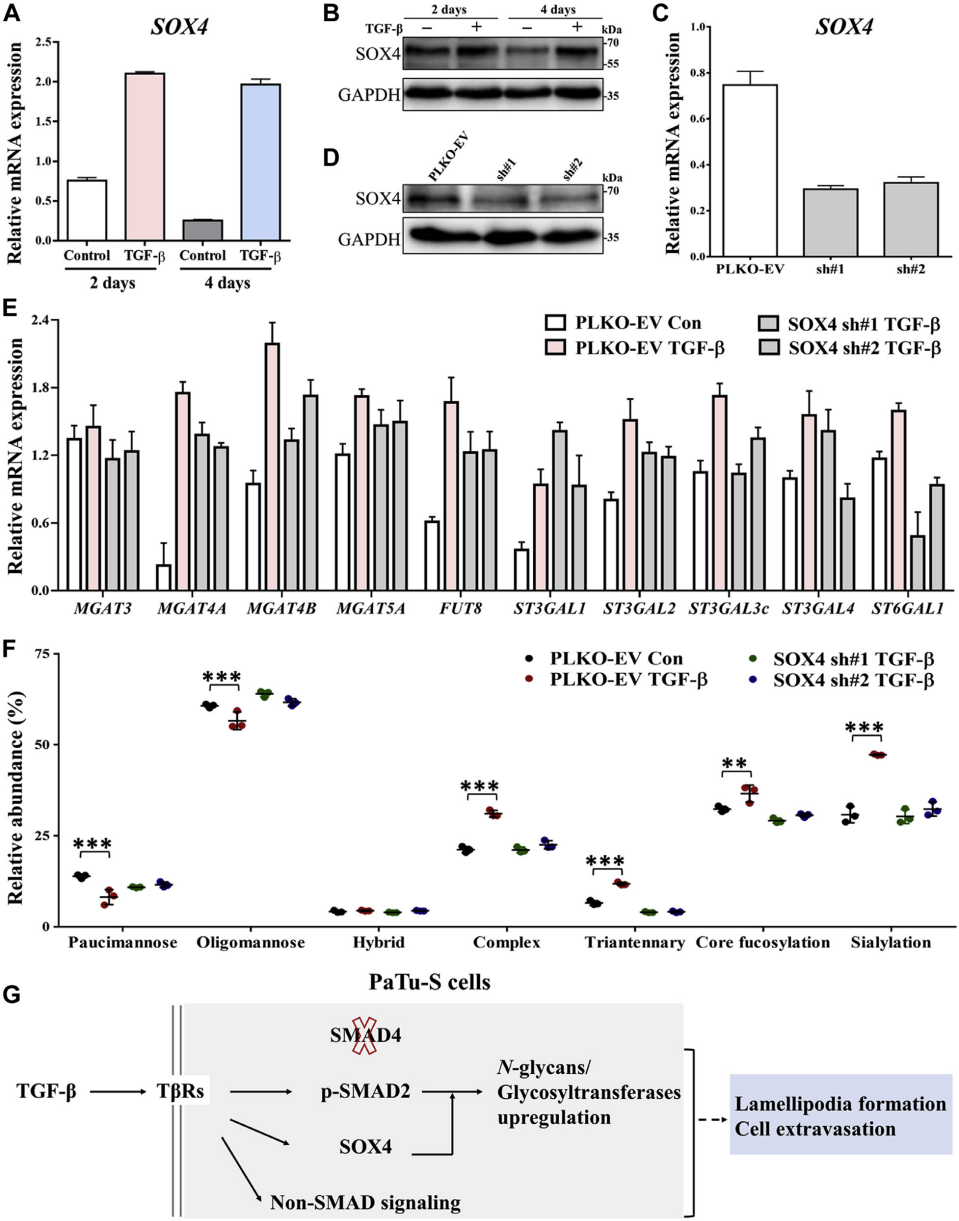
**SOX4, a TGF-β target gene/protein, is needed for TGF-β-induced upregulation of *N*-glycans in PaTu-S cells.***A*, quantitative RT–PCR (qRT–PCR) analysis of the *SOX4* in PaTu-S cells treated with vehicle control or TGF-β for 2 and 4 days. *GAPDH* mRNA levels were used for normalization. *B*, immunoblotting of cell lysates for SOX4 and GAPDH (as loading control); cells were treated with vehicle control or TGF-β for 2 and 4 days. The molecular weight markers are indicated on the *right*. *C*, PaTu-S cells were stably infected with two SOX4 shRNAs (sh#1 and sh#2) or empty vector shRNA (PLKO-EV). qRT–PCR analysis was used for the expression of *SOX4* mRNA. *D*, Western blot analysis of SOX4 expression in PaTu-S cells infected with PLKO-EV, SOX4 sh#1, and SOX4 sh#2. The molecular weight markers are indicated on the *right* and GAPDH, loading control. *E*, qRT–PCR analysis of *N*-glycosylation-associated transcripts in PaTu-S cells with PLKO-EV, SOX4 sh#1, and SOX4 sh#2 after treatment with vehicle control (Con) or TGF-β for 2 days. *F*, relative abundance of structural *N*-glycan classes derived from PaTu-S cells with PLKO-EV, SOX4 sh#1, and SOX4 sh#2 was treated with vehicle control or TGF-β for 6 days on PGC nano-LC–ESI–MS/MS in negative-ion mode. *G*, summary diagram showing the essential role of SOX4 in TGF-β-induced upregulation of *N*-glycans and associated glycosyltransferases in SMAD4-deficient PaTu-S cells. The role, if any, of changes in glycosylation in TGF-β-induced biological responses, such as the F-actin formation and cell extravasation, requires further studies. Fresh medium containing TGF-β (2.5 ng/ml) or vehicle control was added every 2 days in all experiments. Representative results are shown of three independent experiments, or the data are expressed as the mean ± SD (n = 3). ESI, electrospray ionization; PaTu-S, PaTu-8955S; PGC, porous graphitized carbon; SMAD4, SMA-related and MAD-related protein 4; SOX4, sex-determining region SRY-related HMG high-mobility group box 4; TGF-β, transforming growth factor-β.

SOX4 is a key transcriptional target of the TGF-β signaling pathway (28) in various cell types, including breast epithelial cells (78), glioma cells (79), and pancreatic cancers (30). Importantly, SOX4 is induced by TGF-β in an SMAD4-independent manner and promotes tumorigenesis in SMAD4-null PDAC cells (30). Similarly, we found that SOX4 mRNA (Fig. 5*A*) and protein expression levels (Fig. 5*B*) were upregulated in PaTu-S cells upon TGF-β stimulation for 2 and 4 days. To investigate the role of SOX4 in regulating the TGF-β-induced changes in glycan profile, we depleted the SOX4 using two shRNAs in PaTu-S cells. Expression of SOX4 was significantly decreased both at the mRNA (Fig. 5*C*) and protein levels (Fig. 5*D*) in SOX4 knockdown cells. Moreover, SOX4 induction by TGF-β was eliminated by the shRNA-mediated SOX4 depletion (Fig. S5*A*). The SOX4 knockdown efficiency was further validated by the monitoring (downregulated) expression of the established SOX4 target gene, *Nestin* (Fig. S5, *B* and *C*) (30). TGF-β-induced upregulation of *N*-glycosylation-associated genes, including *MGAT4A* and *MGAT4B*, was attenuated by SOX4 depletion (Figs. 5*E* and S5*D*). Accordingly, the relative abundance of complex and specifically triantennary *N*-glycans did not increase anymore with SOX4 depletion even after TGF-β treatment for 6 days (Figs. 5*F* and S5*E*). The same attenuation was observed in sialylation, as a result of the lower expression of *ST3GAL2*, *ST3GAL3*, *ST3GAL4*, and *ST6GAL1* in the TGF-β-treated SOX4 knockdown cells compared with the empty vector group in the presence of TGF-β (Figs. 5*E* and S5*D*). The TGF-β-induced increase in core fucosylation was downregulated by SOX4 knockdown in PaTu-S cells as well as the downregulation of *FUT8* expression (Figs. 5, *E* and *F* and S5, *D* and *E*). To further validate our results obtained by shRNA-mediated SOX4 depletion in TGF-β-induced upregulation of *N*-glycosylation, we used an alternative method to knockdown SOX4 expression, that is, by CRISPR interference. Quantitative PCR and Western blot assays revealed the decreased expression of SOX4 at the gene and protein levels, respectively, in guide-RNA-mediated SOX4 knockdown cells (Fig. S6, *A* and *B*). Consistently, TGF-β-induced increases in complex structures, as well as the gene expression levels including *MGAT4A* and *MGAT4B*, were attenuated by CRISPR interference–mediated SOX4 depletion (Fig. S6, *C* and *D*). Correspondingly, the upregulation of core fucosylation with TGF-β stimulation was inhibited, resulting from the downregulated expression level of the gene *FUT8* by SOX4 knockdown (Fig. S6, *C* and *D*). SOX4 depletion also significantly decreased the relative abundance of sialylation and expression levels of associated genes, that is, *ST3GAL2*, *ST3GAL4*, and *ST6GAL1* (Fig. S6, *C* and *D*). These results indicated that SOX4 is a critical mediator for TGF-β signaling in the PaTu-S cell line. SOX4 plays a key role in the TGF-β-mediated glycosylation responses, in particular in increasing the levels of sialylation, branching, and core fucosylation within the *N*-glycan pool.

In addition, we expanded our study to other SMAD4-deficient pancreatic adenocarcinoma cell lines such as CFPAC-1 and BxPC-3 cells (80). Consistent with the results obtained with PaTu-S cells, we observed that TGF-β induced pSmad2 levels in CFPAC-1 and BxPC-3 cells (Fig. S7*A*). This response was more efficient in BxPC-3 than CFPAC-1 cells, and therefore, BxPC-3 cells were chosen for further study. Upon treatment of BxPC-3 cells with TGF-β, typical TGF-β target genes, such as *CCN2*, *SERPINE1*, and *SMAD7*, were found to be upregulated (Fig. S7*B*). Moreover, TGF-β induced the expression of mesenchymal markers, including *CDH2*, *SNAI1*, and *VIM*, at the mRNA level, but only the protein expression of N-cadherin was increased by TGF-β in BxPC-3 cells (Fig. S7, *C* and *D*). The results obtained with BxPC3 are comparable to the results we obtained using PaTu-S cells. However, the *N*-glycosylation-associated genes, including *MGAT4A*, *MGAT4B*, *MGAT5A*, *FUT8*, *ST6GAL1*, and *FUT1*, were only slightly induced by TGF-β treatment for 2 days or 4 days (Fig. S7*E*). Thus, the glycomic response to TGF-β in BxPC-3 cells is weaker than that obtained in PaTu-S cells. To investigate the role of SOX4 in BxPC-3 cells, we depleted the SOX4 with two independent shRNAs in BxPC-3 cells and validated the SOX4 knockdown by quantitative RT–PCR and Western blotting (Fig. S8, *A* and *B*). The TGF-β-induced upregulation of *N*-glycosylation-associated genes, *MGAT5A* and *ST6GAL1*, was inhibited by SOX4 knockdown (Fig. S8*C*). Thus, SOX4 plays a role in TGF-β-mediated increase of *N*-glycan-associated genes in multiple SMAD4-deficient PDAC lines.

## Discussion

Here, we provide a comprehensive analysis of TGF-β-induced *N*-glycan, *O*-glycan, and GSL-linked glycan patterns in PaTu-S pancreatic adenocarcinoma cells. Our study demonstrated the significant upregulation of branching, sialylation, and core fucosylation of *N*-glycans in TGF-β-treated PaTu-S cells in an SMAD4-independent manner. These *N*-glycosylation changes were found to be largely mirrored by transcriptomic changes of the underlying glycotransferase-associated genes. Indeed, several studies have shown that TGF-β-mediated *N*-glycosylation changes are involved in the TGF-β–SMAD4 signaling pathway but rarely in SMAD4-independent pathways. For example, *ST6GAL1* and its enzymatic products including α2,6-sialylation are significantly increased during TGF-β-SMAD-induced EMT in the mouse epithelial GE11 (SMAD4 active) cell line, which further regulates the cell migration and invasion (81). In our study, the similar upregulation of *ST6GAL1* gene expression level and sialylation was observed in the SMAD4-deficient PaTu-S cell line. Thus, further functional studies are required to investigate the role of these specific glycosylation changes during TGF-β stimulation in SMAD4-deficient PDAC. In addition, the significant upregulation of *O*-glycosylation by TGF-β treatment, which is independent of SMAD4, in the PaTu-S cell line only happened in several types of glycans such as α2,3 sialylation of galactose and core 1 structures. Importantly, the sialylated Tn antigen was notably decreased by TGF-β stimulation in Patu-S cells, which is carried by various glycoproteins, and is associated with cancer progression, invasion, and metastasis in some published studies (82, 83). In line with this TGF-β-induced downregulation, we have also observed the loss of sialylated Tn antigen in the mesenchymal-like Pa-Tu-8988T cells (84), which was derived from the same patient as the PaTu-S cell line. These results together indicate a potential role of sialylated Tn antigen in TGF-β signaling and response in the PaTu-S cell line.

GSLs were shown to be involved in the TGF-β-induced EMT process in normal murine mammary gland NMuMG (85) and human mammary carcinoma MCF7 cells (86), both of which express SMAD4. The promotion or inhibition of TGF-β-induced EMT and cell migration and metastasis by certain GSL, including gangliosides GM2, Gg4, GM3, and GD2, have been studied in the SMAD4-dependent TGF-β response. In the SMAD4-deficient PaTu-S cell line, the only significant differences were the specific expression of globosides Gb3 and Gb4 despite the upregulation of GSL-associated genes upon TGF-β stimulation. This phenomenon suggests that increased gene expression may not result in an increased level of protein expression/activity (87, 88). The neoexpression of globosides following TGF-β treatment is an interesting observation since Gb3 has been reported to be associated with tumor invasion and metastasis in many cancers, including lung cancer (89), colon cancer (90, 91), breast cancer (92), gastric adenocarcinoma (93), and pancreatic cancer (94). In addition, A4GALT, a key enzyme for globoside biosynthesis, can induce EMT and mediate cell–cell adhesion in ovarian cancer cells (88). In our previous study, globosides Gb3 and Gb4 were specifically expressed in the mesenchymal-like Pa-Tu-8988T cells compared with epithelial PaTu-S cells (84). Again, these findings suggest that the globosides may contribute to the invasion and metastasis in PDAC. In the future, it will be of interest to investigate the role of sialylated Tn antigen and globosides, especially Gb3, in TGF-β-mediated PDAC progression.

Importantly, we showed that *SOX4*, a TGF-β transcriptional target gene, is required for TGF-β-induced increases in *N*-glycosylation. Knockdown of SOX4 in PaTu-S cells resulted in the attenuation of TGF-β-mediated increases in branching structures, sialylation, and core fucosylation. Since SOX4 was shown to promote tumorigenesis in PDAC cells independent of SMAD4 expression (30), it will be interesting to investigate the relationship between TGF-β–SOX4-mediated glycosylation changes and tumorigenesis in SMAD4-deficient cells. A recent study demonstrated that the integrin αvβ6–TGF-β–SOX4 pathway regulates multiple signaling events relevant for T cell–mediated tumor immunity in triple-negative breast cancer cells (95). Our previous study of glycosylation in PaTu-S cells revealed that these cells bind to dendritic cells *via* galectins and other lectins, which may have effects on the immune response (84). Thus, the TGF-β–SOX4 signaling pathway–induced upregulation of *N*-glycosylation especially branching, sialylation, and core fucosylation might impact on immune responses in PaTu-S cells. We extended our results on PaTu-S cells to include another SMAD4-deficient PDAC line, that is, BxPC3 cells. We found a relatively weak glycomic response to TGF-β, and that SOX4 depletion decreased the TGF-β-induced increase in *N*-glycan-associated genes in BxPC-3 cells (Fig. S8*C*).

In our study, loss of SMAD4 had no effect on the phosphorylation of the upstream protein, SMAD2, upon TGF-β stimulation in multiple SMAD4-deficient PDAC lines. Interestingly, we found that TGF-β can still significantly promote the mRNA expression of TGF-β target genes, including *CCN2*, *SERPINE1*, *PTHLH*, and *SMAD7*, in SMAD4-deficient PaTu-S and BxPC3 cell lines. This upregulation of TGF-β target genes indicates that some genes do not require SMAD4 for their regulation. Large-scale microarray analysis, which used a tetracycline-inducible siRNA of SMAD4 (96), identified two populations of TGF-β target genes based upon their (in)dependency on SMAD4 in human immortalized keratinocytes (HaCaT) cells (72). Indeed, many TGF-β target genes such as *PTHLH* and *SMAD7* were not affected by SMAD4 depletion. Although the *SERPINE1* gene was classified in the category of SMAD4-dependent genes in this study (72), the authors also emphasized that this gene still displayed a residual induction with TGF-β stimulation after SMAD4 silencing (72). The previous study also demonstrated that the gene expression level of *CCN2* (encoding the protein CTGF) can be induced by the combination of direct SMAD phosphorylation and indirect Janus kinase–signal transducer and activator of transcription 3 activation in activated hepatic stellate cells (97). These reports suggest that TGF-β target genes can be regulated by both SMAD4-dependent and SMAD4-independent pathways. Our data regarding the TGF-β-induced changes in gene expression in the SMAD4-deficient PaTu-S and BxPC-3 cell lines provide further support for this notion. Moreover, we found that SOX4 knockdown had no effect on the TGF-β-mediated increase of target genes *CCN2*, *SERPINE1*, *PTHLH*, and *SMAD7* in PaTu-S cells (Fig. S9). This indicates that expression of these TGF-β-induced target genes is regulated in an SMAD4-independent and SOX4-independent manner.

We investigated the changes in expression of EMT markers upon TGF-β treatment both at the gene and protein levels in PaTu-S and BxPC-3 cells. Mesenchymal markers such as N-cadherin (encoded by the *CDH2* gene), SLUG (encoded by the *SNAL2* gene), and VIM (encoded by the *VIM* gene) are induced by TGF-β treatment for 2 days at the mRNA level. At the protein level, N-cadherin (but not SLUG and VIM [data not shown]) was upregulated in response to TGF-β stimulation for 2 and 4 days in PaTu-S and BxPC-3 cells. In addition, expression and localization of the epithelial marker E-cadherin were barely affected by TGF-β after 2 and 4 days of treatment. Our results indicate that these two SMAD4-deficient cell lines, including the PaTu-S and BxPC-3 cell lines, do not undergo a complete TGF-β-induced EMT. Similar results were also shown in the previous study (30) that SMAD4-mutant cells retained E-cadherin expression after TGF-β treatment for 36 h. In addition, we found that TGF-β promotes lamellipodia formation in PaTu-S cells, using IF staining of the actin cytoskeleton and extravasation using a zebrafish xenograft model. Indeed, TGF-β leads to rearrangements of the actin filament network *via* canonical and noncanonical TGF-β signaling (98, 99, 100). In human prostate carcinoma cells, TGF-β treatment induced a rapid formation of lamellipodia through the SMAD-independent signaling pathway, which requires the activation of the Rho-family GTPases including CDC42 and RHOA (101). Thus, the investigation of the role of altered glycosylation in TGF-β-induced lamellipodia formation in PaTu-S cells may offer clues as to how TGF-β mediates invasion in the zebrafish model and also provides a basis for further studies.

## Experimental procedures

### Materials and chemicals

Ammonium acetate, ammonium bicarbonate, cation exchange resin beads (AG50W-X8), trifluoroacetic acid, potassium hydroxide, and sodium borohydride were obtained from Sigma–Aldrich. Guanidine hydrochloride (8 M) was obtained from Thermo Fisher Scientific. TGF-β3 was generously provided by Dr A. Hinck (University of Pittsburg, PA). DTT and HPLC SupraGradient acetonitrile were obtained from Biosolve (Valkenswaard), and other reagents and solvents, such as chloroform, methanol, ethanol, 2-propanol, and glacial acetic acid, were from Merck. MultiScreen HTS 96 multiwell plates (pore size of 0.45 μm) with high protein-binding membrane (hydrophobic Immobilon-P polyvinylidene difluoride [PVDF] membrane) were purchased from Millipore and conical 96-well Nunc plates from Thermo Fisher. The 50 mg TC18-reverse phase (RP) cartridges were from Waters. Peptide:*N*-glycosidase F (lyophilized, glycerol free) was purchased from Roche Diagnostics. SB431542 was from Tocris Biosciences (catalog no.: 1614), and Alexa Fluor 488 phalloidin was purchased from Thermo Fisher Scientific (catalog no.: A12379). EGCase I (recombinant clone derived from *Rhodococcus triatomea* and expressed in *Escherichia coli*) and 10× EGCase I buffer (500 mM Hepes, 1 M NaCl, 20 mM DTT, and 0.1% Brij 35, pH 5.2) were purchased from New England BioLabs, Inc. Ultrapure water was used for all preparations and washes, generated from a Q-Gard 2 system (Millipore).

### Primers and antibodies

The DNA sequences of human forward and reverse primers that were used to detect the expression of specific genes are listed in Table S4. The antibodies that were used for immunoblotting (IB) and IF: phosphor-SMAD2 1:1000 dilution (IB: catalog no.: 3108; Cell signaling), GAPDH 1:1000 dilution (IB: catalog no.: MAB374; Millipore), E-cadherin 1:1000 dilution (IB and IF: catalog no.: 610181; BD Biosciences), N-cadherin 1:1000 dilution (IB: catalog no.: 610920; BD Biosciences), SLUG 1:1000 dilution (IB: catalog no.: 9585; Cell Signaling), SOX4 1:1000 dilution (IB: Diagenode; catalog no.: C15310129), vinculin (IB: catalog no.: V9131; Sigma), and Alexa Fluor 555 secondary antibody 1:500 dilution (IF: catalog no.: A-21422; Thermo Fisher Scientific).

### Cell culture

PaTu-S cell line was obtained from DSMZ culture bank (102). Human embryonic kidney (HEK) 293T, A549-VIM-red fluorescent protein, pancreatic adenocarcinoma BxPC-3 and CFPAC-1 cell lines were originally purchased from American Type Culture Collection. PaTu-S, HEK293T, A549-VIM-red fluorescent protein, and CFPAC-1 cells were cultured in Dulbecco’s modified Eagle's medium (DMEM) with 10% fetal bovine serum and 100 U/ml penicillin–streptomycin. BxPC-3 cells were maintained in RPMI1640 and supplemented with 10% fetal bovine serum and 100 U/ml penicillin–streptomycin. These cell lines were frequently tested for the absence of *mycoplasma* contamination and authenticated by short tandem repeat profiling. For all the experiments mentioned in this study, the PaTu-S cells were always starved in DMEM with 0.5% serum for 6 h before adding ligands. The concentration of TGF-β was applied as 2.5 ng/ml, and the same volume of ligand buffer (4 mM HCl and 0.1% bovine serum albumin) was used as a vehicle control.

### Western blotting

Cells were lysed with radioimmunoprecipitation assay lysis buffer (150 mM NaCl, 0.1% Triton X-100, 0.5% sodium deoxycholate, 0.1% SDS, 50 mM Tris–HCl, pH 8.0) and freshly added protease and phosphatase inhibitors (catalog no.: 11836153001; Roche) for 10 min at 4 °C. After centrifugation at 11 × 10^3^*g* for 10 min at 4 °C, the protein concentrations were measured using the DC protein assay (Pierce). Equal amounts of proteins were loaded on the gel, proteins were separated by SDS-PAGE, and thereafter protein transferred onto 45 μm PVDF membrane (IPVH00010; Merck Millipore). Western blotting analysis was performed by using specific primary and secondary antibodies, and signals were visualized with chemiluminescence. All the experiments were performed with biological triplicates, and representative results are shown.

### Real-time quantitative RT–PCR

Total RNAs were isolated using the NucleoSpin RNA II kit (catalog no.: 740955; BIOKE′) according to the instructions from the manufacturer. The complementary DNA synthesis was performed with 1 μg of RNA using RevertAid First Strand cDNA Synthesis Kit (catalog no.: K1621; Thermo Fisher Scientific). Real-time RT–PCR experiments were conducted with SYBR Green (Promega) in CFX Connect Detection System (catalog no.: 1855201; Bio-Rad). GAPDH mRNA levels were used to normalize specific target gene expression. For the fold changes of genes, control groups including 2 and 4 days were further used to normalize the gene expression with TGF-β stimulation for 2 and 4 days, respectively. Data are shown as technical triplicates and representative of three independent biological experiments.

### Plasmid construction, single-guide RNA design, and shRNA selection

The human SOX4 single-guide RNAs (sgRNAs) were designed using the online tool CHOPCHOP (https://chopchop.cbu.uib.no/), and two independent sgRNAs with the lowest off activity were chosen. The two complementary sgRNAs contain BveI cut sites, and their sequences are listed in Table S4. The pLKO.1-puro.U6.sgRNA BveI stuffer lentiviral vector was used as a backbone to generate AA19 pLKO.1-SOX4-sgRNA plasmid and obtained from Addgene. The dCas9-KRAB-T2A-puro lentiviral plasmid was from Addgene (catalog no.: 99372).

SOX4 shRNAs for lentiviral transduction were obtained from Sigma (MISSION shRNA library). We tested six SOX4 shRNAs; the two most effective shRNAs for PaTu-S cells were used for further experiments. These were sh#1-SOX4 (TRCN0000018217: 5′-GAAGAAGGTGAAGCGCGTCTA-3′) with PLKO.1-puro vector and sh#2-SOX4 (TRCN0000274207: 5′-GAAGAAGGTGAAGCGCGTCTA-3′) with a vector of TRC2-PLKO-puro. These two shRNAs have almost identical target sequences, but the sh#2-SOX4 contains a woodchuck hepatitis post-transcriptional regulatory element in its backbone vector. For the SOX4 knockdown of BxPC-3 cells, we used two independent shRNAs, including sh#2-SOX4 and sh#12-SOX4 (TRCN0000018213: 5′-CCTTTCTACTTGTCGCTAAAT-3′). The PLKO.1-puro empty vector (catalog no.: SHC001; Sigma) was used as a negative control (it does not contain an shRNA insert).

### Lentiviral transduction and generation of stable cell lines

Lentiviruses were produced by transfecting HEK293T cells with shRNA plasmids, PLKO empty vector, PLV-mCherry, AA19 pLKO.1-SOX4-sgRNA plasmids, or dCas9-KRAB-T2A-puro lentiviral plasmid, and three packaging plasmids that are pCMV-G protein of the vesicular stomatitis virus, pMDLg-RRE (gag-pol), and pRSV-REV as described (103). The viral supernatants were harvested at 48 h post-transfection and filtered through a 0.45 μm polyethersulfone filter. Viruses were either directly used for infection or stored at −80 °C as soon as possible to avoid loss of titer.

To generate stable SOX4 knockdown cell lines, we first prepared a 1:1 dilution of the lentivirus in DMEM complemented with 5 ng/ml of Polybrene (Sigma). Thereafter, PaTu-S cells or BxPC-3 cells were infected with the lentiviral dilution at a low cell density (30%). After infection for 48 h, cells were selected with 2 μg/ml of puromycin for 1 week in order to generate the SOX4-depleted cells.

The PaTu-S mCherry cells were infected with PLV-mCherry lentivirus, and a single colony that highly expresses mCherry was isolated by fluorescence-activated cell sorting for high mCherry expression. Thereafter, high mCherry sorted (PaTu-S mCherry) cells were cultured and expanded.

### IF staining

Cells were seeded onto sterile 18 mm-side square glass coverslips (catalog no.: 631-1331; Menzel Gläser), and complete medium was added. After overnight growth, cells were prestarved with DMEM containing 0.5% serum for 6 h and then treated with vehicle control or with TGF-β for 2 and 4 days. Thereafter, the cells were fixed with 4% paraformaldehyde for 30 min and permeabilized with 0.1% Triton X-100 for 10 min at room temperature. The cells were blocked with 5% bovine serum albumin (catalog no.: A2058; Sigma–Aldrich) in 0.1% PBS with Tween for 1 h. Subsequently, the 1:1000 diluted primary antibody of E-cadherin in PBS was added to cells for 1 h of incubation. After three times washing with PBS, the mixture of 1:500 diluted Alexa Fluor 555 secondary antibody and 1:1000 diluted Alexa Fluor 488 phalloidin was added to the cells for 1 h. Thereafter, the cells were washed with PBS three times and mounted with VECTASHIELD antifade mounting medium with 4′,6-diamidino-2-phenylindole (catalog no.: H-1200; Vector Laboratories). Images were taken by SP8 confocal microscopy (Leica Microsystems). All the experiments were performed in biological triplicates, and representative results are shown.

### Zebrafish extravasation assay

We used the transgenic green fluorescent zebrafish Tg *fli*:enhanced GFP strain for our xenograft studies. The experiments were carried out according to the standard guidelines approved by the local Institutional Committee for Animal Welfare of Leiden University. Zebrafish extravasation assay was performed as previously described (104). In brief, PaTu-S mCherry cells were pretreated with TGF-β for 2 days, and approximately, 400 cells were injected at the ducts of Cuvier into the zebrafish embryos at 48 h postfertilization. Then the injected zebrafish embyos were kept at 33 °C for 4 days after injection. The latter temperature is a compromise for both the fish and cells. Thereafter, the fish were fixed with 4% paraformaldehyde and imaged by an inverted SP5 confocal microscopy (Leica Microsystems). The number of invasive cell clusters (more than five cells were defined as a cluster) between the vessels in the caudal hematopoietic tissue region was counted.

### Preparation of *N*-glycan and *O*-glycan alditols released from PaTu-S cells

*N*-glycan and *O*-glycan alditols released from PaTu-S cells were prepared using a 96-well plate sample preparation method performed as previously described (74, 84). In brief, lysates from 0.5 × 10^6^ cells were applied to the hydrophobic Immobilon-P PVDF membrane in a 96-well plate format. Protein denaturation was achieved by applying 75 μl denaturation mix (72.5 μl 8 M guanidine hydrochloride and 2.5 μl 200 mM DTT) in each well, followed by shaking for 15 min and incubating at 60 °C in a moisture box for 30 min. Subsequently, the unbound material was removed by centrifugation.

The *N*-glycan was released by adding peptide:*N*-glycosidase F (2 U of enzyme diluted with water to 15 μl) to each well and incubated overnight at 37 °C. Released *N*-glycans were collected from the PVDF plate by centrifugation, and the glycosylamine versions of the released *N*-glycans were hydrolyzed by adding 20 μl of 100 mM ammonium acetate (pH 5), incubated at room temperature for 1 h, and dried in a SpeedVac concentrator 5301 (Eppendorf) at 35 ˚C. Collected *N*-glycans were then reduced and desalted, followed by PGC cleanup using a 96-well plate-based protocol (74, 76). Samples were dried in a SpeedVac concentrator directly in PCR plates and redissolved in 10 μl of water prior to PGC nano-LC–ESI–MS/MS analysis.

After removal of *N*-glycans, the *O*-glycans were released from the same PVDF membrane–immobilized sample *via* reductive β-elimination. Briefly, 50 μl of 0.5 M NaBH_4_ in 50 mM KOH was applied onto each PVDF membrane well after rewetting with 3 μl of methanol. Plates were placed for 15 min on a horizontal shaker and incubated in a humidified plastic box for 16 h at 50 °C. After cooling to RT, released *O*-glycans were recovered by centrifugation at 1000*g* for 2 min into 96-well collection plates. The wells were rewetted by 3 μl of methanol and washed three times with 50 μl of water with 10 min incubation steps on a horizontal shaker prior to centrifugation at 500*g* for 2 min. Prior to desalting, the collected samples were concentrated to approximately 30 μl under vacuum in a SpeedVac concentrator at 35 °C for 2 h. Subsequently, 3 μl of glacial acetic acid was added to quench the reaction, followed by brief centrifugation to collect the samples at the bottom of the wells. The following high-throughput desalting and PGC solid-phase extraction (SPE) purification were performed. The purified *O*-glycan alditols were resuspended in 10 μl of water prior to PGC nano-LC–ESI–MS/MS analysis.

### Preparation of GSL-linked glycan alditols released from PaTu-S cells

Extraction of GSLs and preparation of GSL-linked glycan alditols from PaTu-S cells were performed in triplicate as previously described (84). Shortly, 2 × 10^6^ cells were harvested, washed, and resuspended with 200 μl of water. The cell samples were lysed by vortexing and sonication for 30 min. Chloroform (550 μl) was added to the samples, followed by 15 min of sonication. Methanol (350 μl) was added to the cell pellets and incubated for 4 h with shaking at room temperature. The upper phase containing GSLs was collected after centrifugation at 2700*g* for 20 min. Then, 400 μl of chloroform/methanol (2:1, v/v) was added, followed by adding 400 μl of methanol/water (1:1, v/v). After sonication and centrifugation, the upper phase was collected and pooled to the previous sample. The process of adding methanol/water (1:1, v/v), sonication, centrifugation, and removing the upper phase was repeated twice more. In each replicate, the upper phase was collected and replaced by the same volume of methanol/water (1:1, v/v). The combined upper phases were dried under vacuum in an Eppendorf Concentrator 5301 (Eppendorf) at 30 °C.

Before the purification of the GSLs using RP SPE, the samples were dissolved in 100 μl methanol and vortexed for 10 min, followed by the addition of 100 μl water. TC18-RP-cartridges were prewashed with 2 ml of chloroform/methanol (2:1, v/v) and 2 ml of methanol followed by equilibration with 2 ml methanol/water (1:1, v/v). The extracted GSLs were loaded to the cartridge three times and washed with 2 ml methanol/water (1:1, v/v). The GSLs were eluted from the column with 2 ml methanol and 2 ml chloroform/methanol (2:1, v/v). The samples containing the eluate were evaporated under nitrogen for 1 h and dried under vacuum in an Eppendorf concentrator at 30 °C.

To release the glycans from the GSLs, a mixture of EGCase I (12 mU, 2 μl), EGCase I buffer (4 μl), and water (34 μl) (pH 5.2) was added to each sample and incubated for 36 h at 37 °C. The released glycans were collected and loaded on TC18-RP-cartridges, which had been preconditioned with 2 ml of methanol and 2 ml of water. The samples were washed with 200 μl of water, and residual glycans were loaded to the cartridge. Then, 500 μl of water was added to the cartridge to wash the glycans from the column. The flow-through and wash fractions were pooled and dried in an Eppendorf concentrator at 30 °C.

The reduction was carried out with slight modifications following the same procedure as described in previous work (76, 84). In brief, GSL-linked glycans were reduced to alditols in 20 μl of sodium borohydride (500 mM) in potassium hydroxide (50 mM) for 2 h at 50 °C. Subsequently, 2 μl of glacial acetic acid was added to acidify the solution and quench the reaction. The desalting of GSL-linked glycans was performed as previously described. Glycan alditols were eluted with 50 μl of water twice. The combined flow-through and eluate were pooled and dried under vacuum in an Eppendorf concentrator at 30 °C. The carbon SPE cleanup was performed, and the purified glycan alditols were resuspended in 20 μl of water prior to PGC nano-LC–ESI–MS/MS analysis.

### Analysis of *N*-glycan, *O*-glycan, and GSL-linked glycan alditols released from PaTu-S cells using PGC nano-LC–ESI–MS/MS

The analysis of glycan alditols was performed using PGC nano-LC–ESI–MS/MS following a method described previously (74, 84). Measurements were performed on an Ultimate 3000 UHPLC system (Thermo Fisher Scientific) equipped with a home-packed PGC trap column (5 μm Hypercarb, 320 μm × 30 mm) and a home-packed PGC nanocolumn (3 μm Hypercarb 100 μm × 150 mm) coupled to an amaZon ETD speed ion trap (Bruker). Mobile phase A consisted of 10 mM ABC, whereas mobile phase B was 60% (v/v) acetonitrile/10 mM ABC. The trap column was packed with 5 μm particle size PGC stationary phase from Hypercarb PGC analytical column (size 30 × 4.6 mm, 5 μm particle size; Thermo Fisher Scientific), whereas the PGC nanocolumn was packed with 3 μm particle size PGC stationary phase from Hypercarb PGC analytical column (size 100 × 4.6 mm, 3 μm particle size; Thermo Fisher Scientific).

To analyze glycans, 2 μl injections were performed, and trapping was achieved on the trap column using a 6 μl/min loading flow in 1% buffer B for 5 min. Separation was achieved with a multistep gradient of B: 1 to 9% in 1 min and 9 to 49% in 80 min for *N*-glycan and 1 to 52% over 72 min for *O*-glycans followed by a 10 min wash step using 95% of B at a flow rate of 0.6 μl/min. To separate GSL-linked glycans, a linear gradient from 1% to 50% buffer B over 73 min was applied at a 0.6 μl/min flow rate. The column was held at a constant temperature of 45 °C.

Ionization was achieved using the nanoBooster source (Bruker) with a capillary voltage of 1000 V applied and a dry gas temperature of 280 °C at 5 l/min and isopropanol enriched nitrogen at 3 psi. MS spectra were acquired within an *m/z* range of 500 to 1850 for *N*-glycans, 380 to 1850 for *O*-glycans, and 340 to 1850 for GSL-linked glycans in enhanced mode using negative-ion mode, smart parameter setting was set to *m/z* 1200, 900, and 900, respectively. MS/MS spectra were recorded using the top three highest intensity peaks. Structures of detected glycans were studied by MS/MS in negative mode (105). Glycan structures were assigned on the basis of the known MS/MS fragmentation patterns in negative-ion mode (75, 106, 107), elution order, and general glycobiological knowledge, with the help of Glycoworkbench (108) and Glycomod (109) software. Relative quantification of individual glycans was performed by normalizing the total peak area of all glycans within one sample to 100%. Relative abundances of specific glycan-derived traits were displayed by summing the relative intensities of each glycan structure containing the epitope multiplied by the number of epitopes per glycan. Structures are depicted according to the Consortium of Functional Glycomics. *Blue square* is *N*-acetylglucosamine; *yellow square* is *N*-acetylgalactosamine; *green circle* is mannose; *yellow circle* is galactose; *red triangle* is fucose; and *purple diamond* is *N*-acetylneuraminic acid.

### Statistical analysis

Statistical analyses were performed with a Student’s unpaired *t* test using Prism 8 software. The numerical data from triplicates, PGC nano-LC–ESI–MS/MS, and the Zebrafish assay are expressed as the mean ± SD. *p* Value is indicated by *asterisks* in the figures, ∗*p* ≤ 0.05, ∗∗*p* ≤ 0.01, and ∗∗∗*p* ≤ 0.001. *p* ≤ 0.05 was considered statistically significant.

## Data availability

The raw mass spectrometric data files that support the findings of this study are available in GlycoPOST in mzXML format, with the identifier GPST000218, accessible *via* the following link: https://glycopost.glycosmos.org/entry/GPST000218.

## Supporting information

This article contains supporting information.

## Conflict of interest

The authors declare that they have no conflicts of interest with the contents of this article.
