# Supplementary material for: Transforming growth factor-β challenge alters the N-, O-, and glycosphingolipid glycomes in PaTu-S pancreatic adenocarcinoma cells
Source: J Biol Chem. 2022 Feb 11;298(3):101717. doi: 10.1016/j.jbc.2022.101717 (PMC8914387; doi:10.1016/j.jbc.2022.101717)
Supplement: Supplemental Figures S1–S9 and Tables S1–S4 [file mmc1.pdf]

Transforming growth factor-β challenge alters the *N*-, *O*-, and glycosphingolipid glycomes in PaTu-S pancreatic adenocarcinoma cells

Jing Zhang[1], Zejian Zhang[2,3], Stephanie Holst[2], Constantin Blöchl[2,4], Katarina Madunic[2], Manfred Wuhrer[2], Peter ten Dijke[1*] and Tao Zhang[2*]

[1]Oncode Institute and Dept. of Cell Chemical Biology, Leiden University Medical Center, 2300 RC Leiden, The Netherlands. J.Zhang.MCB@lumc.nl; P.ten_Dijke@lumc.nl

[2]Center for Proteomics and Metabolomics, Leiden University Medical Center, Leiden, The Netherlands. m.wuhrer@lumc.nl; T.Zhang@lumc.nl; stephanie.holst@gmx.net

[3]Current address: Department of Medical Research Center, Peking Union Medical College Hospital, Chinese Academy of Medical Sciences and Peking Union Medical College, Beijing, China

[4]Department of Biosciences, University of Salzburg, Salzburg, Austria. constantin.bloechl@sbg.ac.at

Supporting Information

Figure S1. Responses to TGF-β in PaTu-S cell line.

Figure S2. Changes of *N*-glycosylation in PaTu-S cell line without or with TGF-β treatment.

Figure S3. Changes of *O*-glycosylation in PaTu-S cell line without or with TGF-β treatment.

Figure S4. Changes of glycosphingolipids (GSLs) in PaTu-S cell line without or with TGF-β treatment.

Figure S5. *N*-glycosylation changes in PaTu-S cells with SOX4 deletion.

Figure S6. TGF-β-induced upregulation of *N*-glycosylation is attenuated by SOX4 knockdown using CRISPR interference (CRISPRi).

Figure S7. CFPAC-1 and BxPC-3 cells response to TGF-β.

Figure S8. TGF-β-induced upregulation of *N*-glycosylation associated transcripts is attenuated by SOX4 depletion in BxPC-3 cells.

Figure S9. mRNA expression levels of TGF-β target genes in PaTu-S cells with SOX4 deletion.

Table S1. Relative quantification of *N*-glycans without or with TGF-β treatment.

Table S2. Relative quantification of *O*-glycans without or with TGF-β treatment.

Table S3. Relative quantification of GSL-glycans without or with TGF-β treatment.

Table S4. Sequences of primers and plasmids.

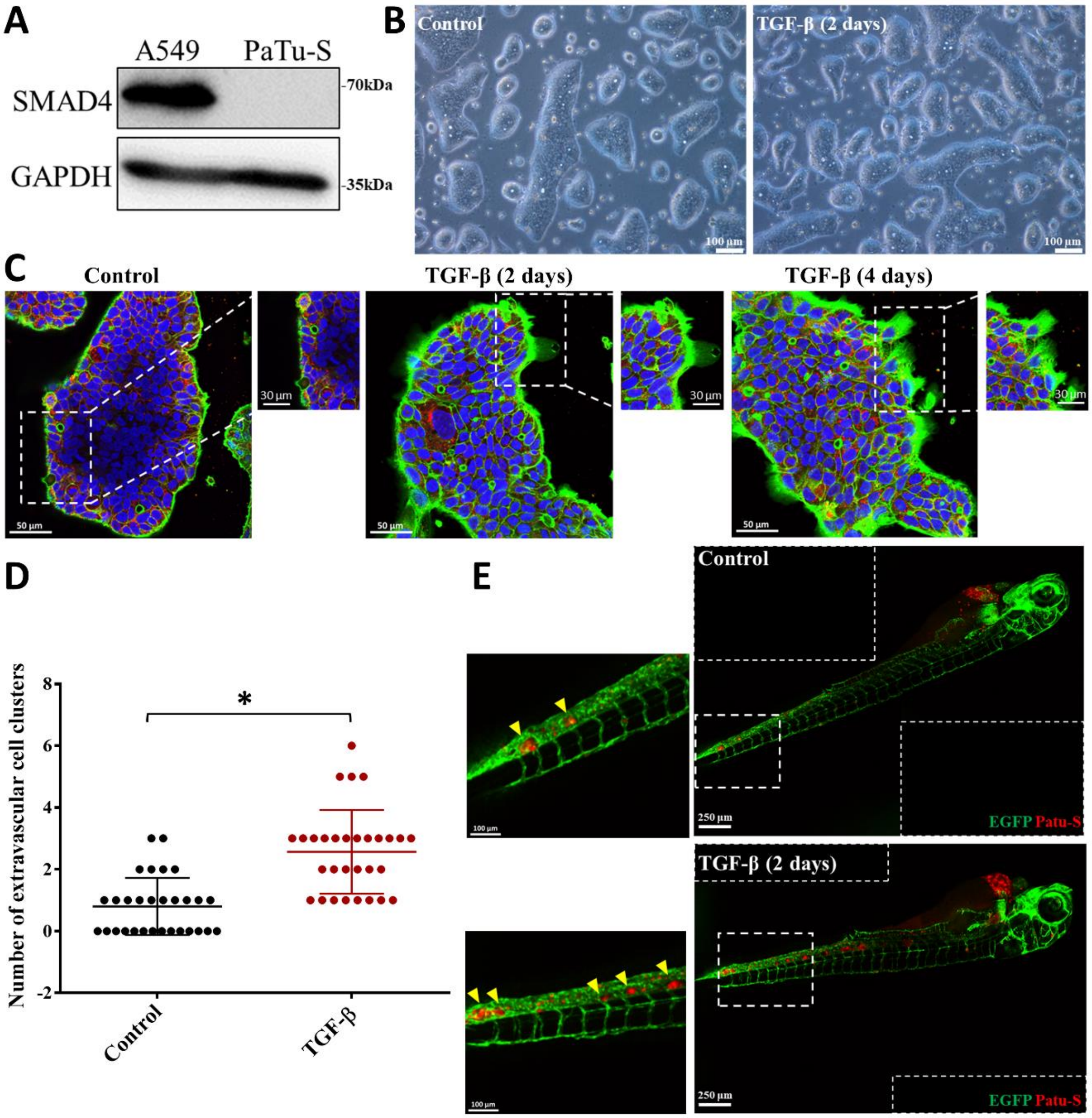

Figure S1. Responses to TGF-β in PaTu-S cell line. (A) The PaTu-S cell line was validated for its SMAD4 deficiency using Western Blot analysis. Analysis of A549-VIM-RFP cell lysates was included as a SMAD4 expressing cell line. GAPDH, loading control. **(B)** Morphological analysis of PaTu-S cells treated with vehicle control (Con) or TGF-β for 2 days. Scale bar = 100 µm. **(C)** PaTu-S cells were double stained with anti-E-cadherin antibody and fluorescein-phalloidin to detect the expression of the epithelial marker, E-cadherin (red) and the formation of filamentous (F)-actin (green), respectively after treatment with vehicle control or TGF-β for 2 days and 4 days. Nuclei were counterstained with DAPI (blue). Dashed boxes indicate the areas of the enlarged images that are shown

in the right panels. At the borders of the cell colonies lamellipodia are visible; the level of these structures were stimulated in response to TGF-β treatment. Images were captured with confocal microscopy. Scale bar = 50 or 30 μm. Experiments were performed in triplicate with similar results, and representative results are shown. **(D)** mCherry labeled PaTu-S cells were pretreated with vehicle control or TGF-β for 2 days, and thereafter injected into ducts of Cuvier of zebrafish embryos. The number of extravasated cell clusters were analyzed four days after injection. Thirty embryos were analyzed in each group. Data is expressed as the mean ± s.d., $^*P \leq 0.05$, unpaired Student's t test, n=1. **(E)** Representative images of zebrafish from control and TGF-β treatment groups. Two zoom-in pictures (outlined with a dotted square) of extravasated cells are shown in the left panels. Yellow arrows indicate the invasive cells. Scale bar = 250 μm or 100 μm. TGF-β was applied at final concentration of 2.5 ng/mL in all experiments.

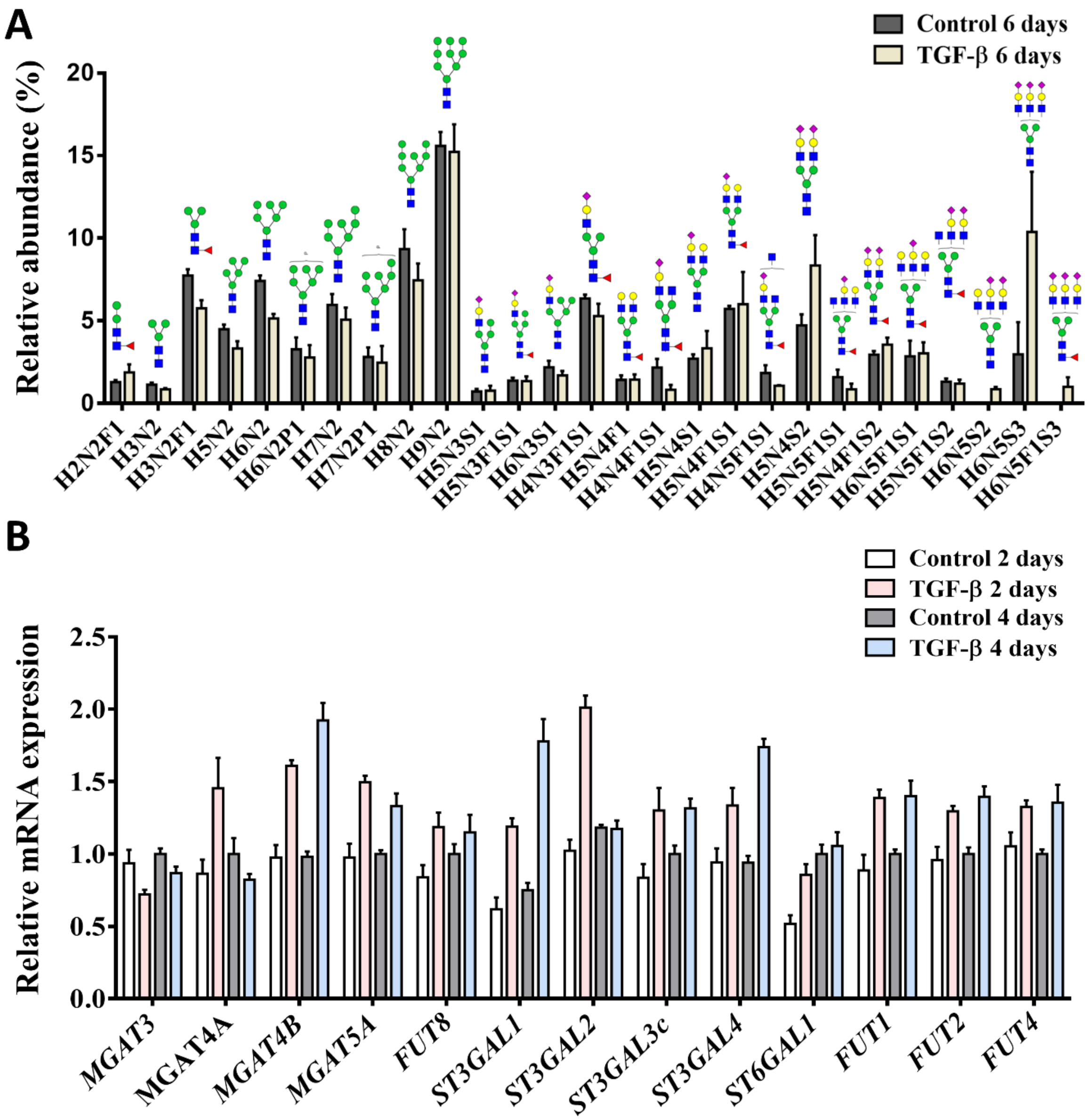

Figure S2. Changes of *N*-glycosylation in PaTu-S cell line without or with TGF-β treatment. (A) Relative quantification of individual *N*-glycans derived from PaTu-S cells treated with vehicle control or TGF-β for 6 days was measured on PGC nano-LC-ESI-MS/MS in negative ion mode. **(B)** qRT-PCR analysis of *N*-glycosylation associated transcripts expression levels in PaTu-S cells treated with vehicle control or TGF-β for 2 days or 4 days. *GAPDH* mRNA levels were used for normalization. Fresh medium containing TGF-β (2.5 ng/mL) or vehicle control was added every 2 days. Representative results are shown of three independent experiments, or the data is expressed as the mean ± s.d. (n=3). Blue square, *N*-acetylglucosamine; yellow square, *N*-acetylgalactosamine;

green circle, mannose; yellow circle, galactose; red triangle, fucose; purple diamond, *N*-acetylneuraminic acid.

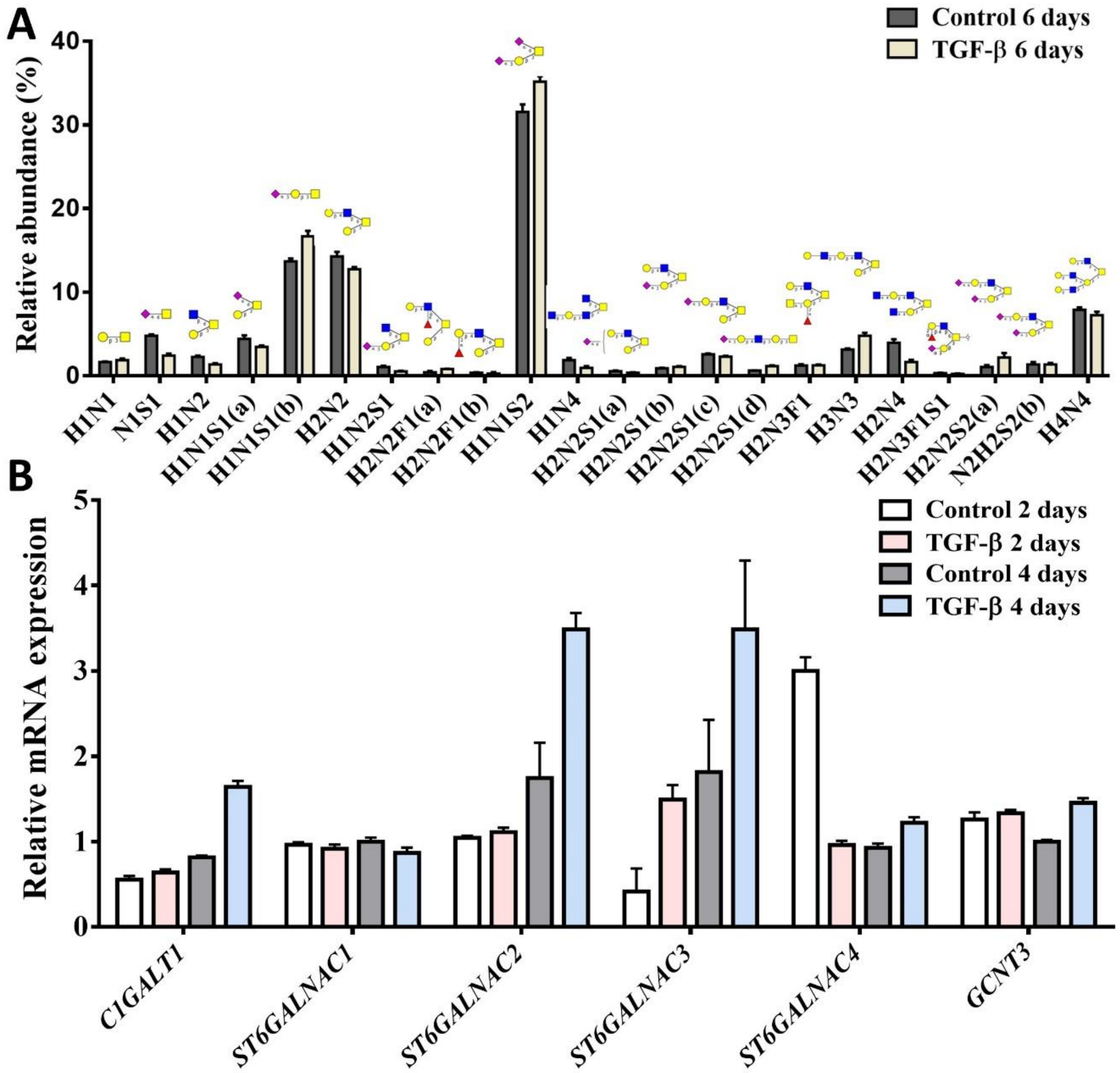

Figure S3. Changes of *O*-glycosylation in PaTu-S cell line without or with TGF-β treatment. (A) Relative quantification of individual *O*-glycans derived from PaTu-S cells treated with vehicle control or TGF-β was measured on PGC nano-LC-ESI-MS/MS in negative ion mode. **(B)** qRT-PCR analysis of *O*-glycosylation associated transcripts expression levels in PaTu-S cells treated with vehicle control or TGF-β for 2 days or 4 days. *GAPDH* mRNA levels were used for normalization. Fresh medium containing TGF-β (2.5 ng/mL) or vehicle control was added every 2 days. Representative results are shown of three independent experiments or the data is expressed as the mean ± s.d. (n=3). Blue square, *N*-acetylglucosamine; yellow square, *N*-acetylgalactosamine; green circle, mannose; yellow circle, galactose; red triangle, fucose; purple diamond, *N*-acetylneuraminic acid.

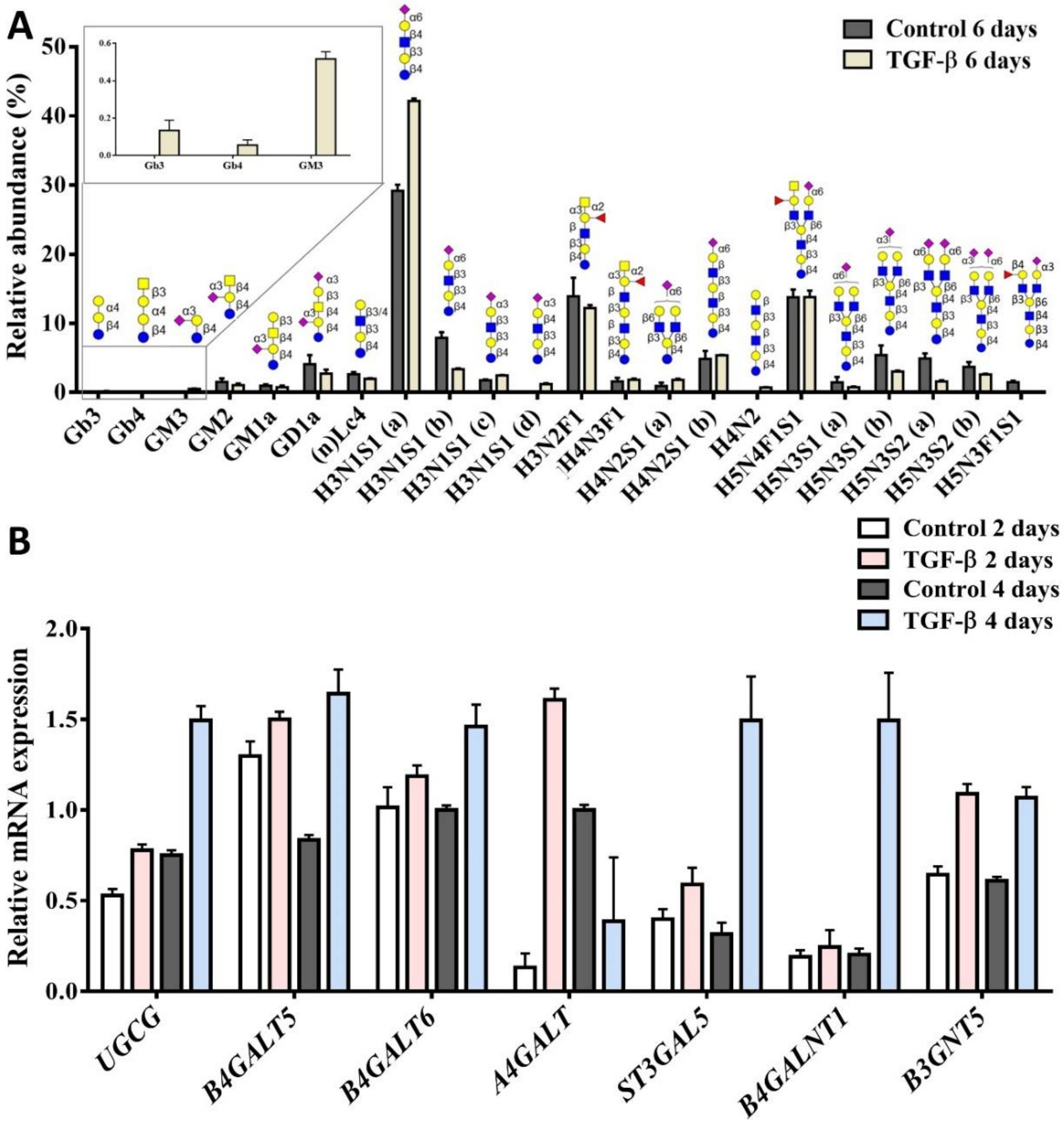

Figure S4. Changes of glycosphingolipids (GSLs) in PaTu-S cell line without or with TGF-β treatment. (A) Relative quantification of individual GSL-glycans derived from PaTu-S cells treated with vehicle control or with TGF-β for 6 days was measured on PGC nano-LC-ESI-MS/MS in negative ion mode. **(B)** qRT-PCR analysis of GSL-associated transcripts expression levels in PaTu-S cells treated with vehicle control or TGF-β for 2 days or 4 days. *GAPDH* mRNA levels were used for normalization. Fresh TGF-β (2.5 ng/mL) or vehicle control was added every 2 days. Representative results are shown of three independent experiments or the data is expressed as the mean ± s.d. (n=3). Blue

square, *N*-acetylglucosamine; yellow square, *N*-acetylgalactosamine; green circle, mannose; yellow circle, galactose; red triangle, fucose; purple diamond, *N*-acetylneuraminic acid.

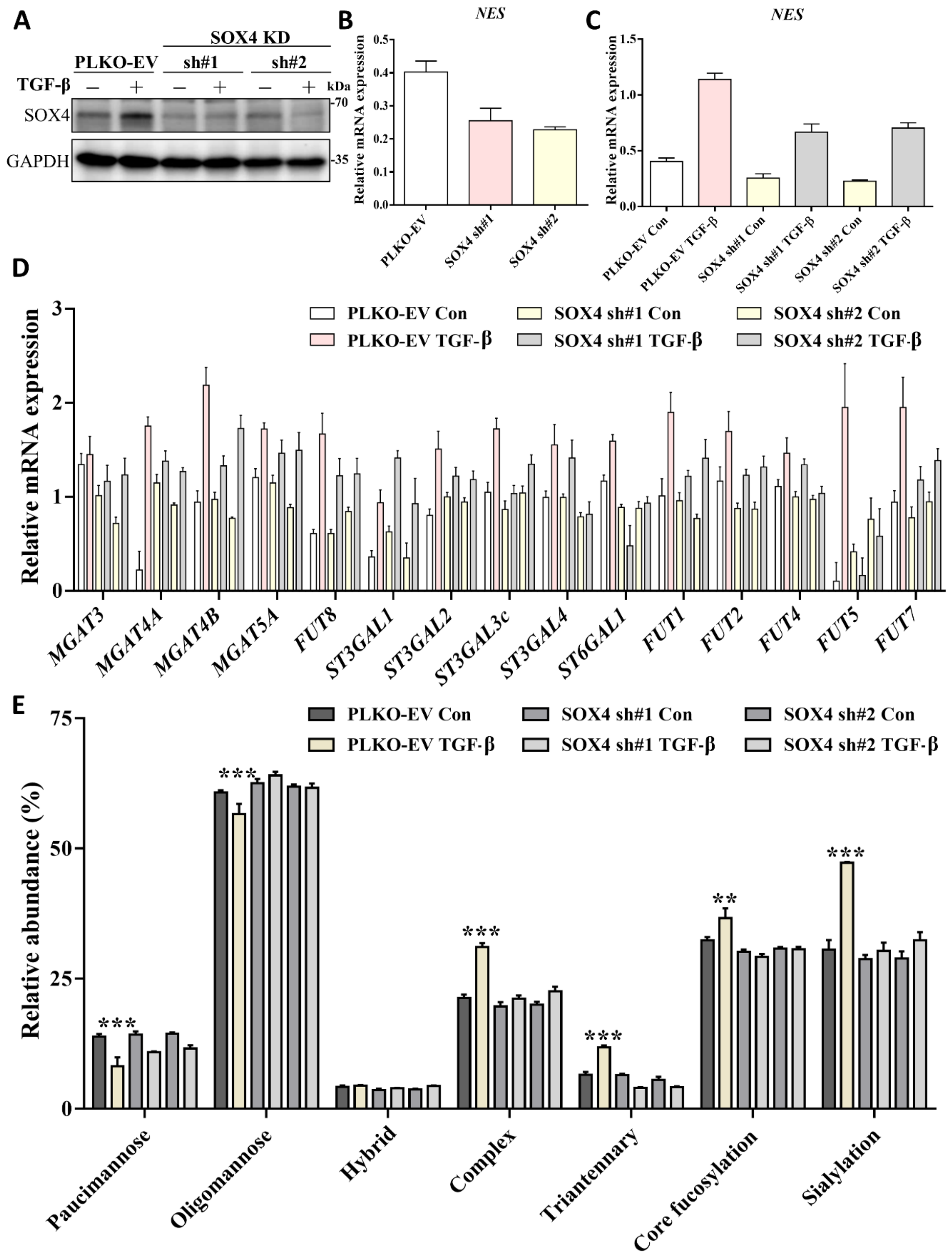

Figure S5. *N*-glycosylation changes in PaTu-S cells with SOX4 deletion. (A) SOX4 immunoblot in PaTu-S cells with two SOX4 shRNAs (sh#1 and sh#2) or empty vector shRNA (PLKO-EV) after treatment with vehicle control or TGF-β for 2 days. The

molecular weight markers are indicated on the right. GAPDH, loading control. **(B)** qRT-PCR analysis of SOX4 target gene *NES* in SOX4 knock down cells. **(C)** qRT-PCR analysis of SOX4 target gene *NES* after SOX4 depletion and vehicle control or TGF-β treatment for 4 days. **(D)** qRT-PCR analysis of *N*-glycosylation-associated transcripts in PaTu-S cells stably infected with PLKO-EV, SOX4 sh#1 and SOX4 sh#2 after treatment with vehicle control (Con) or TGF-β for 2 days. **(E)** Relative abundance of structural *N*-glycan classes in PaTu-S cells with PLKO-EV, SOX4 sh#1 and SOX4 sh#2 treated with vehicle control or TGF-β for 6 days on PGC nano-LC-ESI-MS/MS in negative ion mode. *GAPDH* mRNA levels were used for normalization in all qPCR assays. Fresh medium containing TGF-β (2.5 ng/mL) or vehicle control was added every 2 days in all experiments. Representative results are shown of three independent experiments, or the data is expressed as the mean ± s.d. (n=3). *$P \leq 0.05$, **$P \leq 0.01$, ***$P \leq 0.001$.

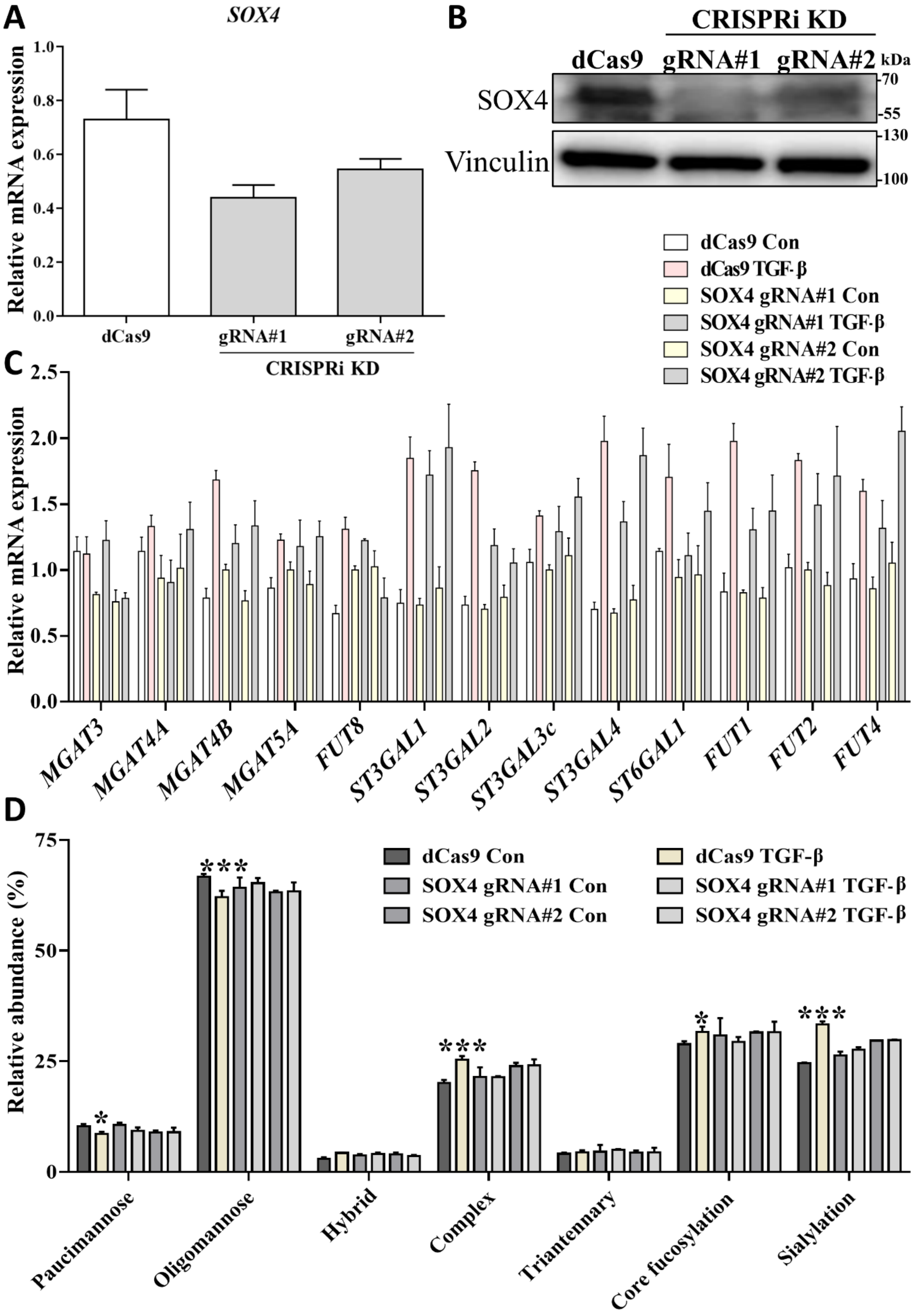
A
SOX4
Relative mRNA expression
dCas9
gRNA#1
gRNA#2
CRISPRi KD
B
CRISPRi KD
dCas9
gRNA#1
gRNA#2
kDa
SOX4
Vinculin
70
55
130
100
dCas9 Con
dCas9 TGF-β
SOX4 gRNA#1 Con
SOX4 gRNA#1 TGF-β
SOX4 gRNA#2 Con
SOX4 gRNA#2 TGF-β
C
Relative mRNA expression
MGAT3
MGAT4A
MGAT4B
MGAT5A
FUT8
ST3GAL1
ST3GAL2
ST3GAL3c
ST3GAL4
ST6GAL1
FUT1
FUT2
FUT4
D
Relative abundance (%)
dCas9 Con
dCas9 TGF-β
SOX4 gRNA#1 Con
SOX4 gRNA#1 TGF-β
SOX4 gRNA#2 Con
SOX4 gRNA#2 TGF-β
Paucimannose
Oligomannose
Hybrid
Complex
Triantennary
Core fucosylation
Sialylation

Figure S6. TGF-β-induced upregulation of *N*-glycosylation is attenuated by SOX4 knockdown using CRISPR interference (CRISPRi). (A) qRT-PCR analysis of SOX4 in PaTu-S cells infected with two independent guide RNAs (gRNA#1 and gRNA#2) or dCas9. **(B)** Immunoblotting of SOX4 in stably infected PaTu-S cells with gRNA#1, gRNA#2 or dCas9. The molecular weight markers are indicated on the right. Vinculin, loading control. **(C)** qRT-PCR analysis of *N*-glycosylation associated transcripts in PaTu-S cells with gRNA#1, gRNA#2 or dCas9 after treatment with vehicle control (Con) or TGF-β for 2 days. **(D)** Relative abundance of *N*-glycan classes treated with or without TGF-β for 6 days and analyzed by PGC nano-LC-ESI-MS/MS. *GAPDH* mRNA levels were used for normalization in the qRT-PCR assays. Fresh medium containing TGF-β (2.5 ng/mL) or vehicle control was added every 2 days in all experiments. Representative results are shown of three independent experiments, or the data is expressed as the mean ± s.d. (n=3). $^*P \leq 0.05$, $^{**}P \leq 0.01$, $^{***}P \leq 0.001$.

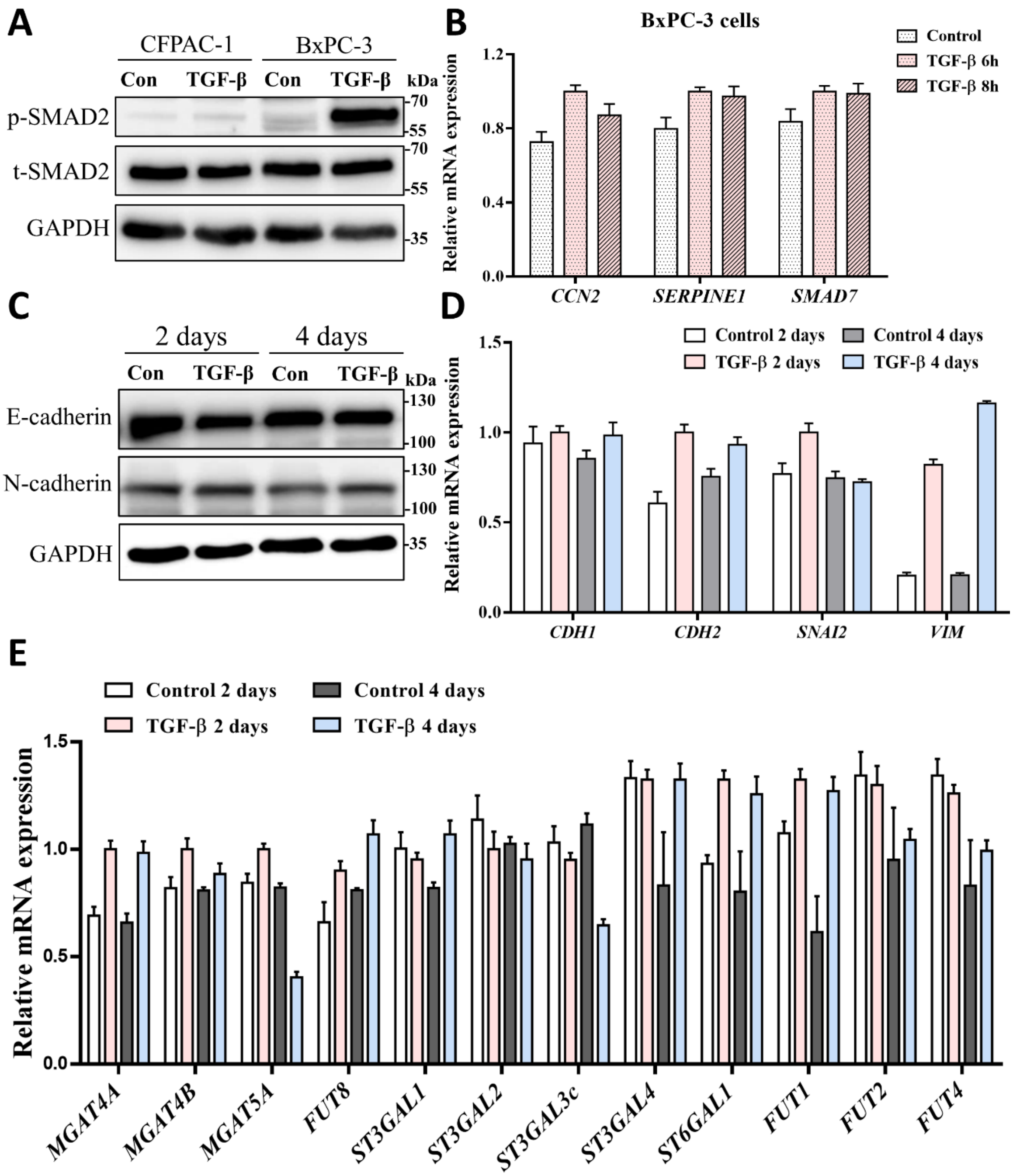

Figure S7. CFPAC-1 and BxPC-3 cells response to TGF-β (A) Western blot analysis of p-SMAD2 levels in CFPAC-1 and BxPC-3 cells treated with vehicle control (Con) or TGF-β for 1 h. GAPDH, loading control. **(B)** qRT-PCR analysis of TGF-β target genes, including *CCN2*, *SERPINE1* and *SMAD7* in BxPC-3 cells treated with vehicle control or TGF-β for 6 and 8 h. **(C)** Western blot analysis of E-cadherin and N-cadherin levels in BxPC-3 cells treated with vehicle control or TGF-β for 2 and 4 days. GAPDH, loading

control. **(D)** qRT-PCR analysis of epithelial and mesenchymal markers including *CDH1*, *CDH2*, *SNAL2* and *VIM* in BxPC-3 cells treated with vehicle control or TGF-β for 2 days and 4 days. **(E)** qRT-PCR analysis of *N*-glycan associated glycosyltransferases gene expression levels in BxPC-3 cells treated with vehicle control or TGF-β for 2 days or 4 days. *GAPDH* mRNA levels were used for normalization in all qRT-PCR experiments. Fresh medium containing TGF-β (2.5 ng/mL) or vehicle control was added every 2 days in all experiments. Three independent experiments were performed. Representative results are shown, or the data is expressed as the mean ± s.d. (n=3).

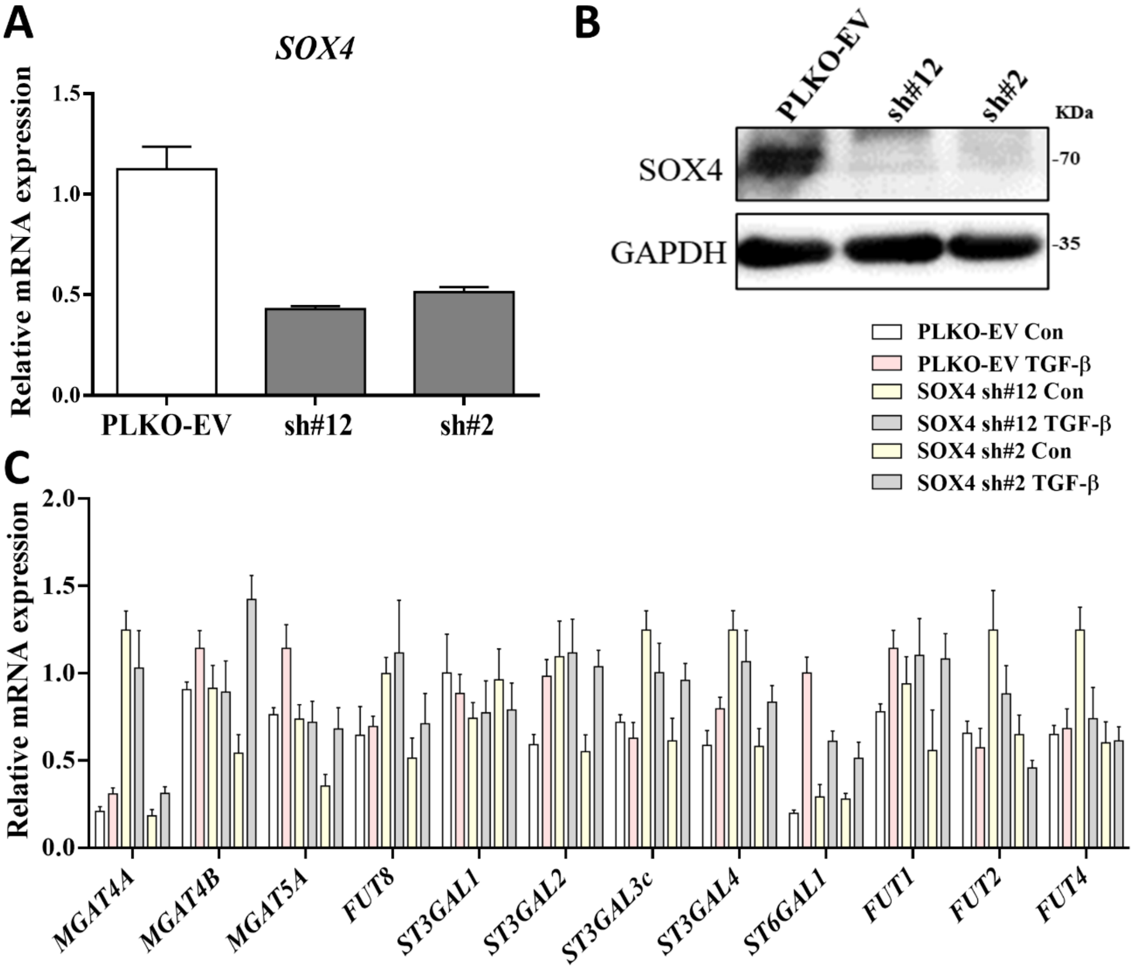

Figure S8. TGF-β-induced upregulation of *N*-glycosylation associated transcripts is attenuated by SOX4 depletion in BxPC-3 cells. (A) qRT-PCR analysis of *SOX4* in BxPC-3 cells that were stably infected of two independent shRNAs (sh#12 and sh#2) and PLKO-EV. **(B)** Immunoblotting of SOX4 in BxPC-3 cells with SOX4 sh#1, SOX4 sh#2 and PLKO-EV. GAPDH, loading control. **(C)** qRT-PCR analysis of *N*-glycosylation associated transcripts in BxPC-3 cells stably infected with PLKO-EV, SOX4 sh#1 and SOX4 sh#2 after treatment with vehicle control (Con) or TGF-β (2.5 ng/mL) for 2 days. *GAPDH* mRNA levels were used for normalization in all qRT-PCR assays. Three independent experiments were performed. Representative results are shown, or the data is expressed as the mean ± s.d. (n=3).

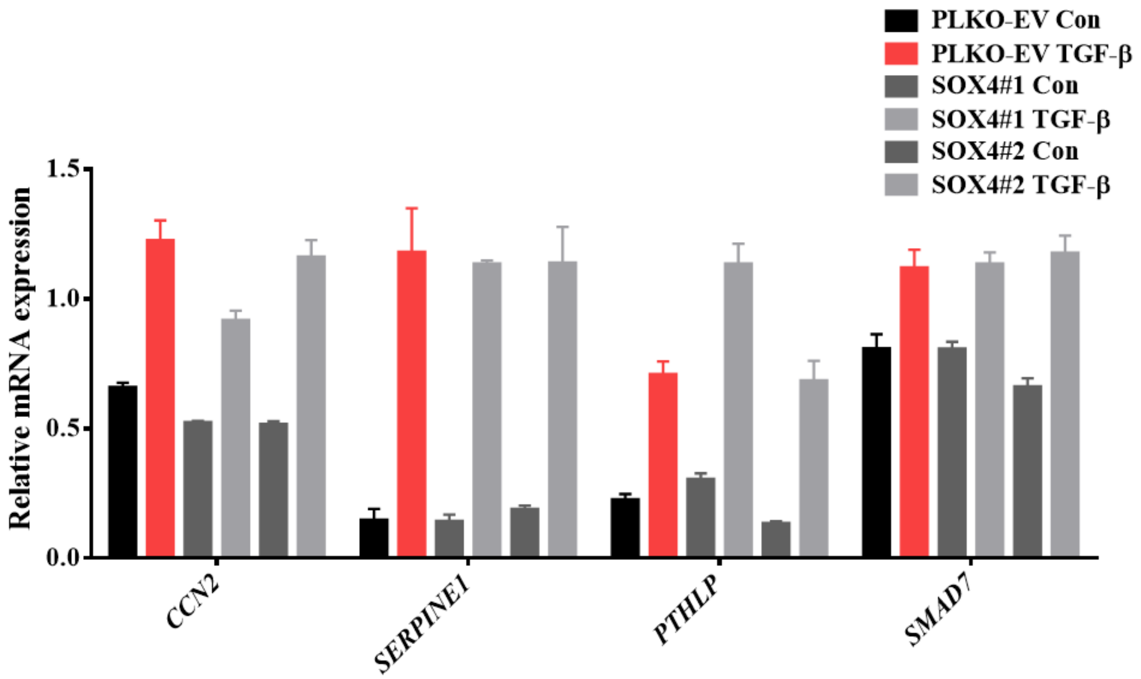

Figure S9. mRNA expression levels of TGF-β target genes in PaTu-S cells with SOX4 deletion. qRT-PCR analysis of *CCN2*, *SERPINE1*, *PTHLH* and *SMAD7* in PaTu-S cells infected with PLKO-EV, SOX4 sh#1 and SOX4 sh#2. after vehicle control (Con) or TGF-β (2.5 ng/mL) treatment for 6 h. *GAPDH* mRNA levels were used for normalization. Data are expressed as the mean ± s.d, of triplicates and are representative of at least two independent experiments.

Table S1. Relative quantification of *N*-glycans released from 0.5 million cells using a 96-well plate sample preparation method and PGC nano-LC-ESI-MS/MS. Proposed structures were assigned based on MS/MS fragmentation and glycobiological pathway constraints. Structures are depicted according to the CFG (Consortium of Functional Glycomics). Blue square: *N*-acetylglucosamine, green circle: mannose, yellow circle: galactose, red triangle: fucose, pink diamond: *N*-acetylneuraminic acid. Composition: H: hexose; N: *N*-acetylhexosamines; S: *N*-acetylneuraminic acid; P: phosphate; a, b, c, d: isomer number. SD: standard variation.

Glycan number	Composition	Proposed structure	Relative abundance %. (SD %)		Observed ions	Theoretical	Deviation	Matched fragments for MS/MS
			Control	TGF-β	$[M\text{-}H]^{X-}$	$[M\text{-}H]^{X-}$	$\Delta[M\text{-}H]^{X-}$	
1	H2N2F1	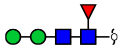	1.29 (±0.12)	1.88 (±0.48)	895.34^{1-}	895.37^{1-}	0.03^{1-}	11
2	H3N2	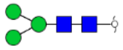	1.13 (±0.13)	0.85 (±0.08)	911.36^{1-}	911.36^{1-}	0.00^{1-}	14
3	H3N2F1	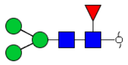	7.74 (±0.39)	5.77 (±0.49)	1057.39^{1-}	1057.46^{1-}	0.07^{1-}	18
4	H5N2	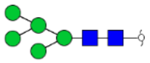	4.49 (±0.29)	3.32 (±0.44)	1235.47^{1-}	1235.44^{1-}	0.03^{1-}	13
5	H6N5	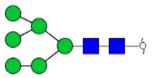	7.42 (±0.33)	5.15 (±0.26)	1397.50^{1-}	1397.50^{1-}	0.00^{1-}	15
6	H6N5P1	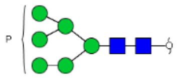	3.28 (±0.71)	2.77 (±0.76)	738.24^{2-}	738.23^{2-}	0.01^{2-}	19

**7**	**H7N2**	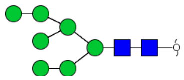	**5.97 (±0.65)**	**5.07 (±0.73)**	**779.31^{2-}**	**779.27^{2-}**	**0.04^{2-}**	**21**
**8**	**H7N2P1**	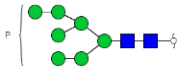	**2.81 (±0.58)**	**2.46 (±1.02)**	**819.28^{2-}**	**819.25^{2-}**	**0.03^{2-}**	**16**
**9**	**H8N2**	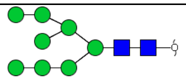	**9.34 (±1.21)**	**7.45 (±1.01)**	**860.31^{2-}**	**860.30^{2-}**	**0.01^{2-}**	**18**
**10**	**H9N2**	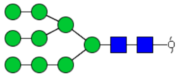	**15.59 (±0.84)**	**15.22 (±1.67)**	**941.35^{2-}**	**941.32^{2-}**	**0.03^{2-}**	**15**
**11**	**H5N3S1**	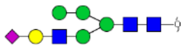	**0.72 (±0.15)**	**0.77 (±0.30)**	**864.32^{2-}**	**864.31^{2-}**	**0.01^{2-}**	**15**
**12**	**H5N3F1S1**	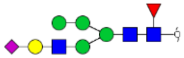	**1.37 (±0.18)**	**1.35 (±0.29)**	**937.37^{2-}**	**937.34^{2-}**	**0.03^{2-}**	**17**
**13**	**H6N3S1**	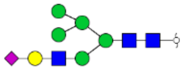	**2.19 (±0.39)**	**1.70 (±0.26)**	**945.36^{2-}**	**945.33^{2-}**	**0.03^{2-}**	**18**
**14**	**H4N3F1S1**	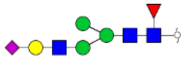	**6.35 (±0.23)**	**5.29 (±0.74)**	**856.33^{2-}**	**856.30^{2-}**	**0.03^{2-}**	**16**
**15**	**H5N4F1**	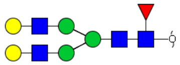	**1.43 (±0.27)**	**1.45 (±0.31)**	**893.37^{2-}**	**893.33^{2-}**	**0.04^{2-}**	**20**
**16**	**H4N4F1S1**	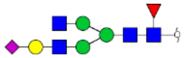	**2.17 (±0.52)**	**0.83 (±0.30)**	**957.89^{2-}**	**957.85^{2-}**	**0.04^{2-}**	**18**

17	H5N4S1	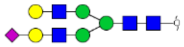	2.71 (±0.18)	3.35 (±1.95)	965.87^{2-}	965.84^{2-}	0.03^{2-}	14
18	H5N4F1S1	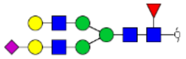	5.73 (±0.12)	6.00 (±0.12)	1038.92^{2-}	1038.87^{2-}	0.05^{2-}	20
19	H4N5F1S1	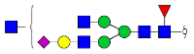	1.84 (±0.48)	1.07 (±0.03)	1059.39^{2-}	1059.39^{2-}	0.00^{2-}	23
20	H5N4S2	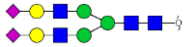	4.73 (±0.67)	8.35 (±1.83)	1111.43^{2-}	1111.42^{2-}	0.01^{2-}	10
21	H5N5F1S1	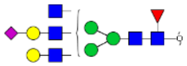	1.60 (±0.45)	0.85 (±0.36)	1140.39^{2-}	1140.41^{2-}	0.02^{2-}	19
22	H5N4S2F1	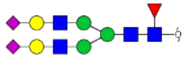	2.96 (±0.21)	3.56 (±0.42)	1184.46^{2-}	1184.42^{2-}	0.04^{2-}	12
23	H6N5F1S1	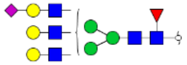	2.86 (±0.21)	3.04 (±0.42)	1221.44^{2-}	1221.45^{2-}	0.01^{2-}	16
24	H5N5F1S2	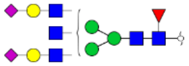	1.31 (±0.93)	1.20 (±0.66)	1285.96^{2-}	1285.96^{2-}	0.00^{2-}	3
25	H6N5S2	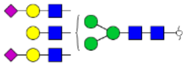	n.d.	0.24 (±0.13)	1293.96^{2-}	1293.96^{2-}	0.00^{2-}	11
26	H6N5S3	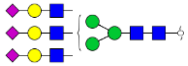	2.97 (±1.95)	10.38 (±3.64)	959.36^{3-}	959.34^{3-}	0.02^{3-}	7

27	H6N5F1S3	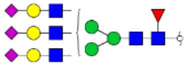	n.d.	1.01 (±0.58)	1008.02^{3-}	1008.02^{3-}	0.00^{3-}	5

Table S2. Relative quantification of *O*-glycans released from 0.5 million cells using a 96-well plate sample preparation method and PGC nano-LC-ESI-MS/MS. Proposed structures were assigned based on MS/MS fragmentation and glycobiological pathway constraints. Structures are depicted according to the CFG (Consortium of Functional Glycomics). Blue square: *N*-acetylglucosamine, yellow square: *N*-acetylgalactosamine, yellow circle: galactose, red triangle: fucose, pink diamond: *N*-acetylneuraminic acid. Composition: H: hexose; N: *N*-acetylhexosamines; S: *N*-acetylneuraminic acid. a, b, c, d: isomer number. SD: standard variation.

Glycan number	Composition	Proposed structure	Relative abundance %. (SD %)		Observed ions	Theoretical	Deviation	Matched fragments for MS/MS
			Control	TGF-β	[M-H]$^{X-}$	[M-H]$^{X-}$	Δ[M-H]$^{X-}$	
**1**	**H1N1**	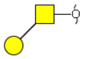	**1.64 (±0.09)**	**1.88 (±0.19)**	**384.13^{1-}**	**384.15^{1-}**	**0.02^{1-}**	**6**
**2**	**N1S1**	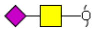	**4.79 (±0.18)**	**2.44 (±0.22)**	**513.19^{1-}**	**513.19^{1-}**	**0.00^{1-}**	**2**
**3**	**H1N2**	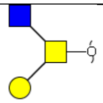	**2.27 (±0.16)**	**1.36 (±0.15)**	**587.23^{1-}**	**587.23^{1-}**	**0.00^{1-}**	**7**
**4a**	**H1N1S1**	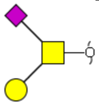	**4.43 (±0.41)**	**3.46 (±0.15)**	**675.24^{1-}**	**675.25^{1-}**	**0.01^{1-}**	**10**
**4b**	**H1N1S1**	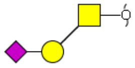	**13.67 (±0.34)**	**16.68 (±0.66)**	**675.24^{1-}**	**675.25^{1-}**	**0.00^{1-}**	**5**
**5**	**H2N2**	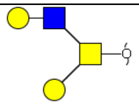	**14.27 (±0.56)**	**12.76 (±0.25)**	**749.28^{1-}**	**749.28^{1-}**	**0.00^{1-}**	**10**

**6**	**H1N2S1**	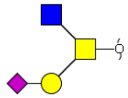	**1.06 (±0.16)**	**0.53 (±0.08)**	**878.33^{1-}**	**878.33^{1-}**	**0.00^{1-}**	**7**
**7a**	**H2N2F1**	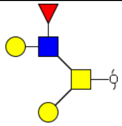	**0.43 (±0.15)**	**0.83 (±0.03)**	**895.28^{1-}**	**895.34^{1-}**	**0.06^{1-}**	**7**
**7b**	**H2N2F1**	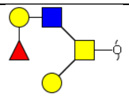	**0.37 (±0.15)**	**0.28 (±0.17)**	**895.28^{1-}**	**895.34^{1-}**	**0.06^{1-}**	**9**
**8**	**H1N1S2**	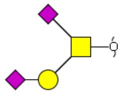	**31.56 (±0.88)**	**35.17 (±0.63)**	**966.34^{1-}**	**966.34^{1-}**	**0.00^{1-}**	**6**
**9**	**H1N4**	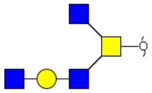	**1.87 (±0.26)**	**0.94 (±0.21)**	**993.41^{1-}**	**993.39^{1-}**	**0.02^{1-}**	**11**
**10a**	**H2N2S1**	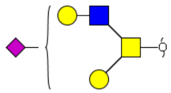	**0.55 (±0.07)**	**0.37 (±0.07)**	**1040.34^{1-}**	**1040.39^{1-}**	**0.05^{1-}**	**5**
**10b**	**H2N2S1**	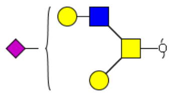	**0.91 (±0.07)**	**1.08 (±0.08)**	**1040.39^{1-}**	**1040.39^{1-}**	**0.00^{1-}**	**7**
**10c**	**H2N2S1**	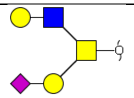	**2.58 (±0.12)**	**2.31 (±0.09)**	**1040.37^{1-}**	**1040.39^{1-}**	**0.02^{1-}**	**10**
**10d**	**H2N2S1**	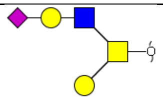	**0.64 (±0.03)**	**1.14 (±0.12)**	**1040.37^{1-}**	**1040.39^{1-}**	**0.02^{1-}**	**11**

**11**	**H2N3F1**	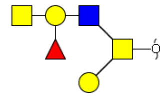	**1.23 (±0.15)**	**1.26 (±0.10)**	**1098.42^{1-}**	**1098.39^{1-}**	**0.03^{1-}**	**14**
**12**	**H3N3**	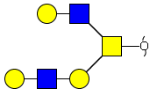	**3.15 (±0.15)**	**4.80 (±0.35)**	**1114.42^{1-}**	**1114.42^{1-}**	**0.00^{1-}**	**12**
**13**	**H2N4**	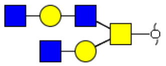	**3.96 (±0.40)**	**1.65 (±0.25)**	**1155.45^{1-}**	**1155.44^{1-}**	**0.01^{1-}**	**13**
**14**	**H2N3F1S1**	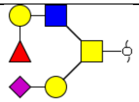	**0.32 (±0.07)**	**0.24 (±0.05)**	**1186.50^{1-}**	**1186.44^{1-}**	**0.06^{1-}**	**15**
**15a**	**H2N2S2**	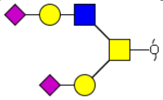	**1.05 (±0.23)**	**2.19 (±0.54)**	**665.21^{2-}**	**665.23^{2-}**	**0.02^{2-}**	**8**
**15b**	**H2N2S2**	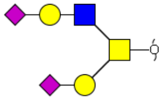	**1.36 (±0.28)**	**1.37 (±0.18)**	**665.23^{2-}**	**665.23^{2-}**	**0.00^{2-}**	**6**
**16**	**H4N4**	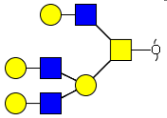	**7.89 (±0.29)**	**7.25 (±0.41)**	**739.27^{2-}**	**739.27^{2-}**	**0.00^{2-}**	**11**

Table S3. Relative quantification of GSL-glycans released from 2 million cells using PGC nano-LC-ESI-MS/MS. Glycan structures were assigned based on MS/MS fragmentation and glycobiological pathway constraints. Structures are depicted according to the CFG (Consortium of Functional Glycomics). Blue square: *N*-acetylglucosamine, yellow square: *N*-acetylgalactosamine, blue circle: glucose, yellow circle: galactose, red triangle: fucose, pink diamond: *N*-acetylneuraminic acid. a, b: isomer number; SD: standard variation.

**Glycan number**	**Composition**	**Proposed structure**	**Relative abundance %. (SD %)**		**Observed ions**	**Theoretical**	**Deviation**	**Matched fragments for MS/MS**
			**Control**	**TGF-β**	**$[M-H]^{X-}$**	**$[M-H]^{X-}$**	**$\Delta[M-H]^{X-}$**	
**1**	**H3**	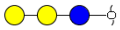	**n.d.**	**0.13 (±0.09)**	**505.19^{1-}**	**505.34^{1-}**	**0.15^{1-}**	**12**
**2**	**H3N1**	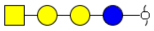	**n.d.**	**0.06 (±0.05)**	**708.28^{1-}**	**708.26^{1-}**	**0.02^{1-}**	**14**
**3**	**H2S1**	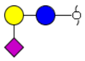	**n.d.**	**0.52 (±0.07)**	**634.27^{1-}**	**634.22^{1-}**	**0.05^{1-}**	**7**
**4**	**H2N1S1**	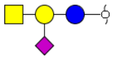	**1.52 (±0.88)**	**1.04 (±0.46)**	**837.32^{1-}**	**837.30^{1-}**	**0.02^{1-}**	**5**
**5**	**H3N1S1**	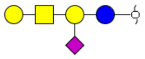	**0.97 (±0.37)**	**0.76 (±0.40)**	**999.35^{1-}**	**999.35^{1-}**	**0.00^{1-}**	**5**
**6**	**H3N1S2**	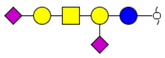	**4.09 (±2.24)**	**2.73 (±0.99)**	**644.73^{2-}**	**644.74^{2-}**	**0.01^{2-}**	**8**

7	H3N1	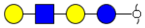	2.62 (±0.55)	1.97 (±0.13)	708.28^{1-}	708.26^{1-}	0.02^{1-}	7
8	H3N1S1	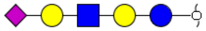	29.19 (±1.51)	42.17 (±0.64)	999.39^{1-}	999.35^{1-}	0.04^{1-}	7
9	H3N1S1	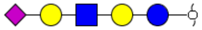	7.89 (±1.40)	3.35 (±0.24)	999.37^{1-}	999.35^{1-}	0.02^{1-}	8
10	H3N1S1	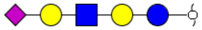	1.74 (±0.21)	2.44 (±0.10)	999.37^{1-}	999.35^{1-}	0.02^{1-}	10
11	H3N1S1	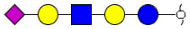	n.d.	1.19 (±0.23)	999.37^{1-}	999.35^{1-}	0.02^{1-}	9
12	H3N2F1	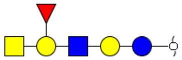	13.91 (±4.67)	12.20 (±0.73)	1057.43^{1-}	1057.39^{1-}	0.04^{1-}	12
13	H4N3F2	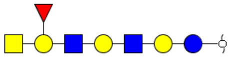	1.59 (±0.93)	1.84 (±0.28)	1422.55^{1-}	1422.53^{1-}	0.02^{1-}	21
14	H4N2S1	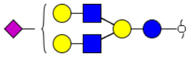	0.92 (±0.86)	1.80 (±0.30)	1364.56^{1-}	1364.48^{1-}	0.08^{1-}	10
15	H4N2S1	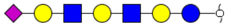	4.84 (±2.02)	5.36 (±0.11)	1364.52^{1-}	1364.48^{1-}	0.04^{1-}	10
16	H4N2	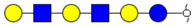	n.d.	0.71 (±0.08)	1073.36^{1-}	1073.39^{1-}	0.03^{1-}	15

**17**	**H5N4F1S1**	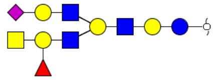	**13.76 (±1.95)**	**13.78 (±1.64)**	**1038.88^{2-}**	**1038.87^{2-}**	**0.01^{2-}**	**27**
**18**	**H5N3S1**	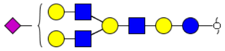	**1.44 (±1.39)**	**0.74 (±0.20)**	**864.32^{2-}**	**864.32^{2-}**	**0.00^{2-}**	**18**
**19**	**H5N3S1**	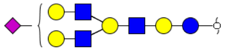	**5.41 (±1.41)**	**3.03 (±0.22)**	**864.32^{2-}**	**864.32^{2-}**	**0.00^{2-}**	**20**
**20**	**H5N3S2**	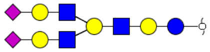	**4.92 (±1.26)**	**1.58 (±0.27)**	**1009.84^{2-}**	**1009.86^{2-}**	**0.02^{2-}**	**7**
**21**	**H5N3S2**	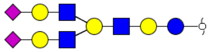	**3.68 (±1.24)**	**2.61 (±0.14)**	**1009.84^{2-}**	**1009.86^{2-}**	**0.02^{2-}**	**8**
**22**	**H5N3F1S1**	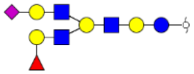	**1.49 (±0.35)**	**n.d.**	**937.32^{2-}**	**937.34^{2-}**	**0.02^{2-}**	**20**

Table S4. Sequences of primers and sqRNAs

**Primers**			
**Species**	**Gene name**	**Forward (5' to 3')**	**Reverse (5' to 3')**
Human	*GAPDH*	TGCACCACCAACTGCTTAGC	GGCATGGACTGTGGTCATGAG
Human	*CCN2*	TTGCGAAGCTGACCTGGAAGAGAA	AGCTCGGTATGTCTTCATGCTGGT
Human	*SERPINE1*	CACAAATCAGACGGCAGCACT	CATCGGGCGTGGTGAACTC
Human	*PTHLH*	AGACTGGTTCAGCAGTGGAG	TTCTTCCCAGGTGTCTTGAG
Human	*SMAD7*	TCCAGATGCTGTGCCTTCC	GTCCGAATTGAGCTGTCCG
Human	*CDH1*	CAGCCGCTTTCAGATTTTCAT	CCCGGTATCTTCCCCGC
Human	*CDH2*	CAGACCGACCCAAACAGCAAC	GCAGCAACAGTAAGGACAAACATC
Human	*SNAI2*	TGTTGCAGTGAGGGCAAGAA	GACCCTGGTTGCTTCAAGGA
Human	*VIM*	CCAAACTTTTCCTCCCTGAACC	CGTGATGCTGAGAAGTTTCGTTGA
Human	*MGAT3*	GCACTTCTTCAAGACCCTGTCCTA	AGAAAAAGCTGGACACCAGGTTA
Human	*MGAT4A*	CTGTGGAAGTTTTGCCTTTTAAGAG	TGAAATGGGATTGAGACTTGGA
Human	*MGAT4B*	CGGAGGACAAGCTCTTCAACA	AGGGCCTCCTTGTCTGACTGA
Human	*MGAT5A*	GATGTGCTTTTCTGAATCCCAAGT	GCCGCCCGATGAAAACT
Human	*FUT8*	TCTTCATCCCCGTCCTCCA	GAGACACCCACCACACTGCA
Human	*ST3GAL1*	GGGCAGACAGCAAAGGGAA	GGCCGTCACGTTAGACTCAAA
Human	*ST3GAL2*	ACAGGTGGACAGAGCATCAC	CCCGTACACGTTCACCTCAT
Human	*ST3GAL3c*	GGGTCACGAATTGACGACTATG	GTGATGCGCAGTGTCGTTTT
Human	*ST3GAL4*	ATAAGAAGCGGGTGCGAAAGGG	TCCGTGGCTGTTGCATTGGC
Human	*ST6GAL1*	CATCCAAGCGCAAGACTGACG	TGTGCCCTGGTTGAGATGCTTC
Human	*FUT1*	GCAGGCCATGGACTGGTT	CCTGGGAGGTGTCGATGTTT
Human	*FUT2*	CTCGCTACAGCTCCCTCATCTT	CGTGGGAGGTGTCAATGTTCT
Human	*FUT4*	GAGCTACGCTGTCCACATCACC	CAGCTGGCCAAGTTCCGTATG
Human	*FUT5*	GTCCCGAGACGATGCCACT	CCGGTGACAGGTTCCACTG
Human	*FUT7*	TCCGCGTGCGACTGTTC	ACCCTCAAGGTCCTCATAGACTTG
Human	*B3GNT3*	GTGGGACTTCCACGACTCCTT	GCACCTTGTCTCCTGCCACT
Human	*GAL4*	GTATAAGAGCCACCACCGCC	GTAGTAAGGCAGCGTCGGG
Human	*C1GALT1*	CCAGAGAAGCAAAGGTCACCA	TTCCCGAAAGTGTATTTCTGACATC
Human	*ST6GALNAC1*	GACGCTGACCCTTCGCTATT	TGCGTTGATGCACATAAGCC
Human	*ST6GALNAC2*	TCCGCGACTATGTGATGCTG	TGATTTCAAGAACCTGTCCCCT
Human	*ST6GALNAC3*	TGCCCAAATATACGTGACCACA	TCACTCTGTACTGTCCTTCCCA
Human	*ST6GALNAC4*	GTTCACCATGATCCTCGCG	TGACACTCATCTAGCCGGCC
Human	*GCNT3*	TGTTCACATTTGCTGCCACG	GTGGAGGAGGACACAATCCTTT
Human	*UGCG*	TGATCAGGTGGACCAAACTACG	ATCTGAACACATGGTGGGCT
Human	*B4GALT5*	TGCAGGCTATTCTGTGAGCC	CCTCAGCAGAGCATACCTTCC
Human	*B4GALT6*	CCATACCTCCCCTGTCCAGA	TTTTGGCCTCCAATGACCCC
Human	*A4GALT*	AACACTGGGGTGATGCAGG	AAATCCATCTCGATATTCTGCAGTG
Human	*ST3GAL5*	GCACCACTGTCTGACCTTGA	CCAGAATGGCAGGGTTTCCT
Human	*B4GALNT1*	TGAGGACCCTCAGGCCG	GCCACATCCTGTCTAACGCT
Human	*B3GNT5*	CCAGCGACTTTAGCTCCGAT	TTCCATGCCACCTCCAAGTC

Human	*NES*	TCAGCTTTCAGGACCCCAAG	GGTGTCTCAAGGGTAGCAGG
**sgRNA guides**			
**Species**	**Name**	**Forward (5' to 3')**	**Reverse (5' to 3')**
Human	*SOX4* sgRNA#1	ACCGCGGAATCGGCACTAAGGAGT	AAACACTCCTTAGTGCCGATTCCG
Human	*SOX4* sgRNA#2	ACCGTAGAGACCCGACAGCGAAAC	AAACGTTTCGCTGTCGGGTCTCTA
