# Supplementary material for: Transforming growth factor-β challenge alters the N-, O-, and glycosphingolipid glycomes in PaTu-S pancreatic adenocarcinoma cells
Source: J Biol Chem. 2022 Feb 11;298(3):101717. doi: 10.1016/j.jbc.2022.101717 (PMC8914387; doi:10.1016/j.jbc.2022.101717)

Supplementary Figure S10
for

Transforming growth factor-β challenge alters the *N*-, *O*-, and glycosphingolipid glycomes in PaTu-S pancreatic adenocarcinoma cells

Jing Zhang[1], Zejian Zhang[2,3], Stephanie Holst[2], Constantin Blöchl[2,4], Katarina Madunic[2], Manfred Wuhrer[2], Peter ten Dijke[1*] and Tao Zhang[2*]

[1]Oncode Institute and Dept. of Cell Chemical Biology, Leiden University Medical Center, 2300 RC Leiden, The Netherlands.
[2]Center for Proteomics and Metabolomics, Leiden University Medical Center, Leiden, The Netherlands.
[3]Current address: Department of Medical Research Center, Peking Union Medical College Hospital, Chinese Academy of Medical Sciences and Peking Union Medical College, Beijing, China
[4]Department of Biosciences, University of Salzburg, Salzburg, Austria.

Supplementary Figure S10. Annotated MS/MS for *N*-glycans. *N*-glycans have been numbered according to Supplementary Table 1. Glycan schemes were derived from GlycoWorkbench. Annotation was based on the presence of structural feature ions and common knowledge of known glycan synthetic pathways.

Glycan 1

H2N2F1

**Monoisotopic mass:**	**896.35 Da**
**Charge observed:**	**1-**
**Theoretical ion:**	***m/z* 895.34**
**Observed ion:**	***m/z* 895.37**
**Mass deviation:**	***m/z* 0.03**
**Retention time:**	**38.1 min**

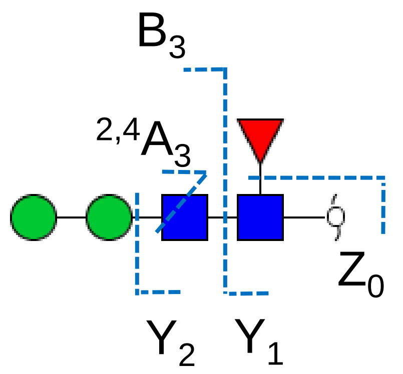

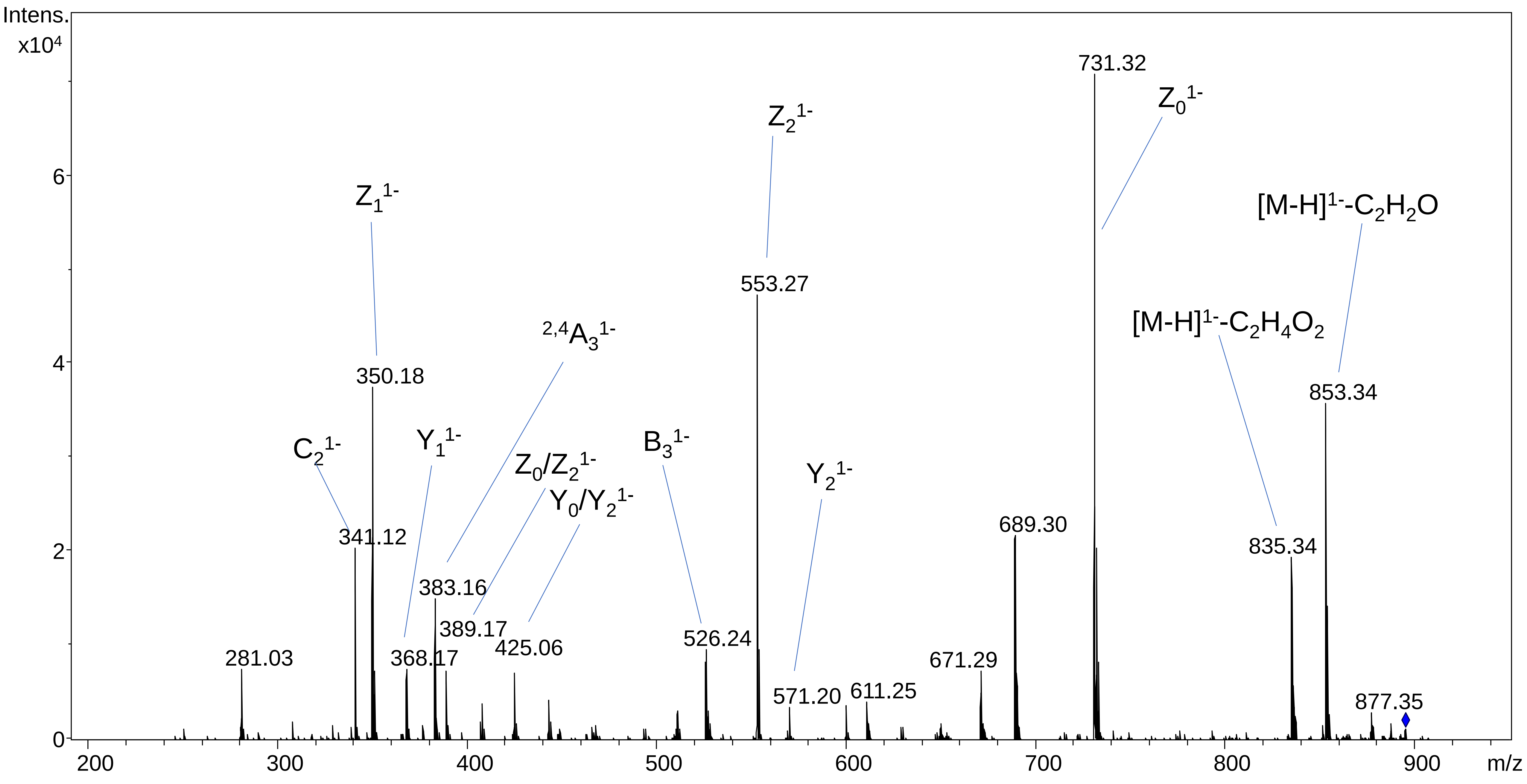

Glycan 2

H3N2

**Monoisotopic mass:**	**912.35 Da**
**Charge observed:**	**1-**
**Theoretical ion:**	***m/z* 911.36**
**Observed ion:**	***m/z* 911.36**
**Mass deviation:**	***m/z* 0.00**
**Retention time:**	**36.0 min**

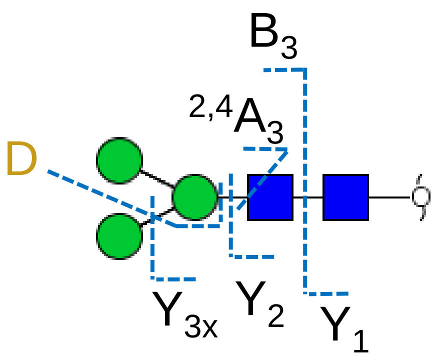

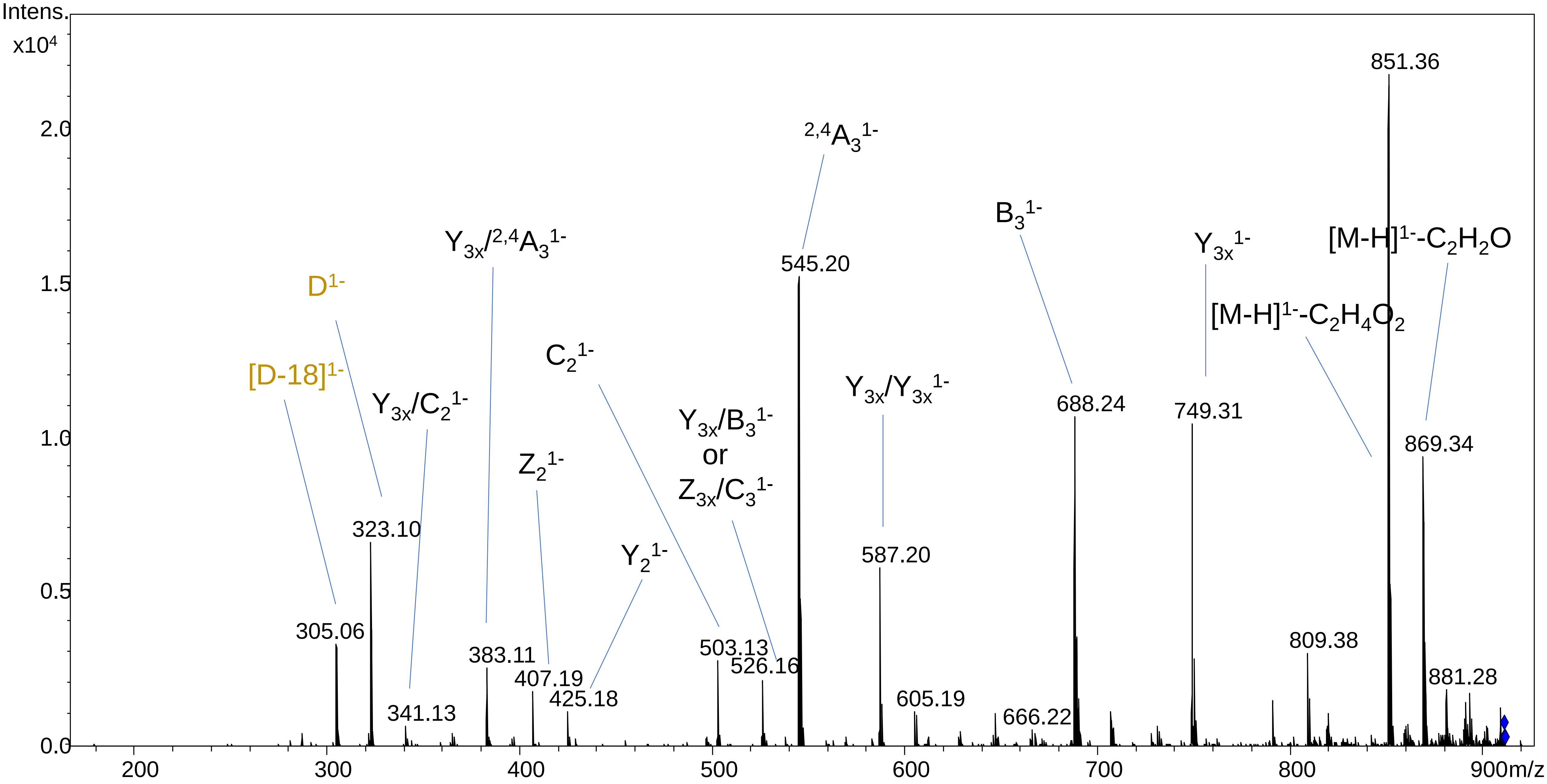

Glycan 3

H3N2F1

**Monoisotopic mass:**	**1058.40 Da**
**Charge observed:**	**1-**
**Theoretical ion:**	***m/z* 1057.39**
**Observed ion:**	***m/z* 1057.46**
**Mass deviation:**	***m/z* 0.07**
**Retention time:**	**45.1 min**

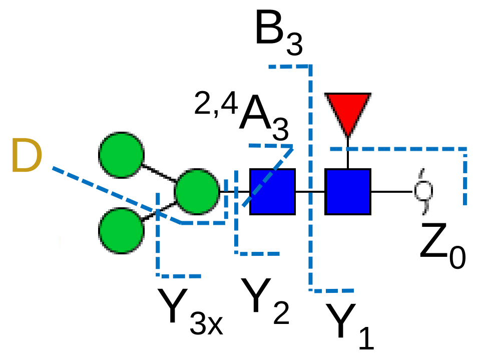

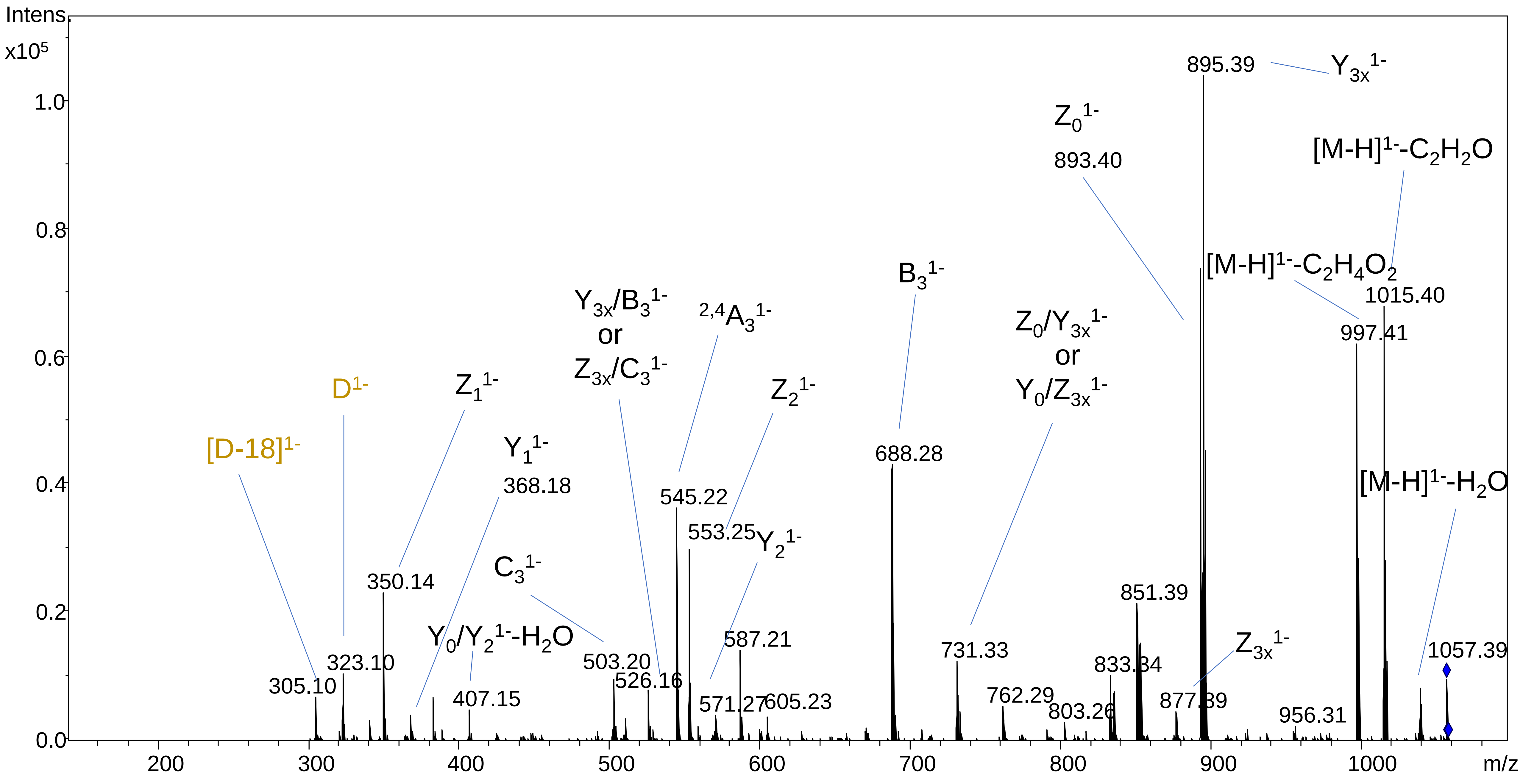

Glycan 4

H5N2

**Monoisotopic mass:**	**1236.45 Da**
**Charge observed:**	**1-**
**Theoretical ion:**	***m/z* 1235.44**
**Observed ion:**	***m/z* 1235.47**
**Mass deviation:**	***m/z* 0.03**
**Retention time:**	**38.1 min**

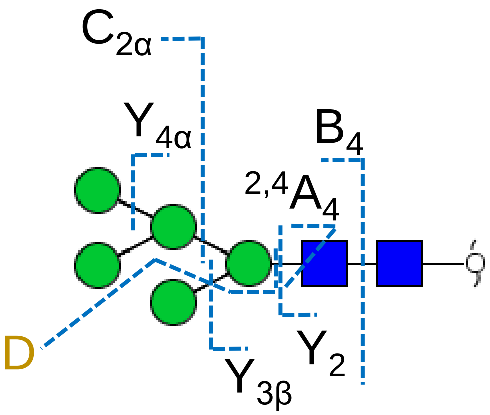

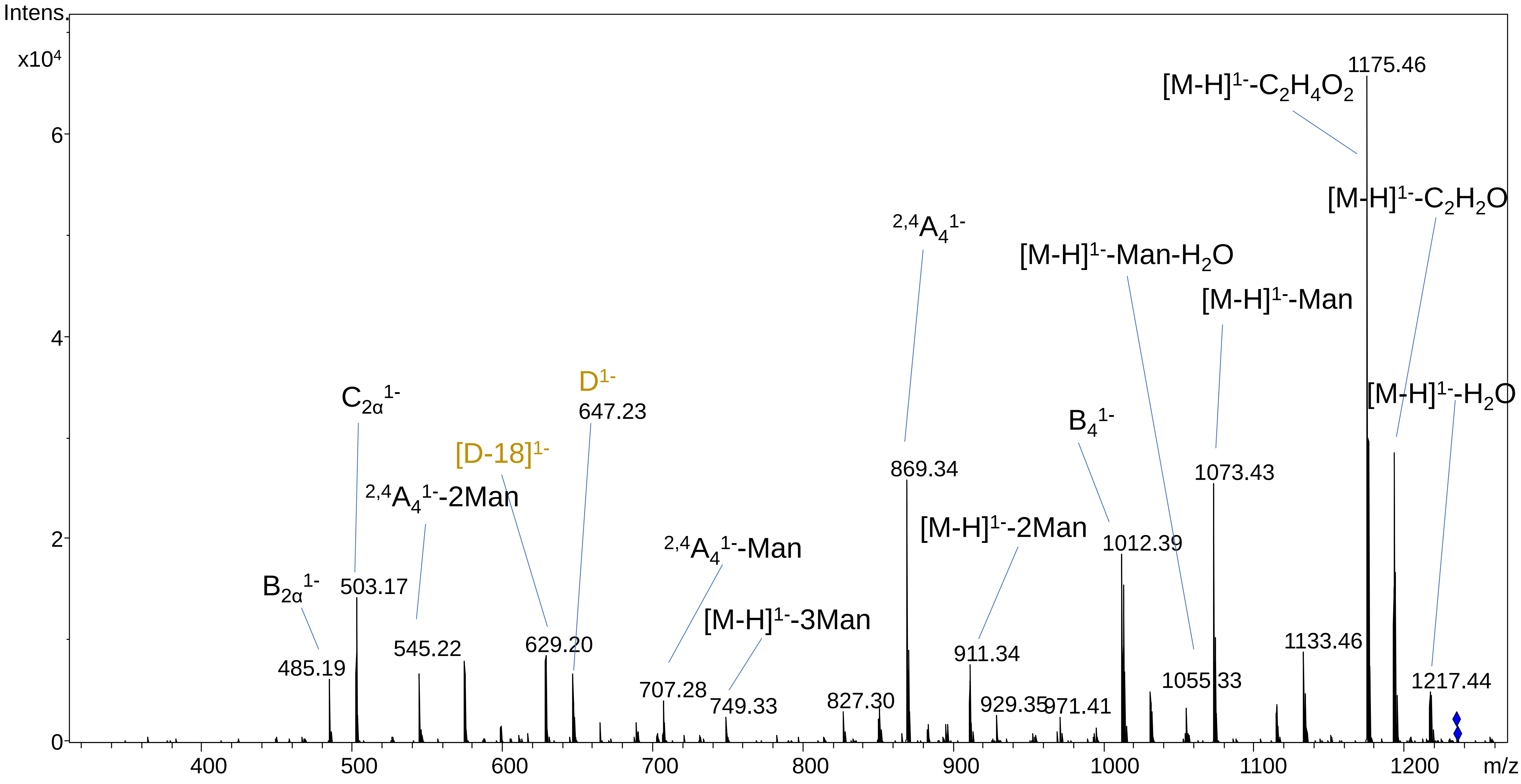

Glycan 5

H6N2

**Monoisotopic mass:**	**1398.51 Da**
**Charge observed:**	**1-**
**Theoretical ion:**	***m/z* 1397.50**
**Observed ion:**	***m/z* 1397.50**
**Mass deviation:**	***m/z* 0.00**
**Retention time:**	**30.5 min**

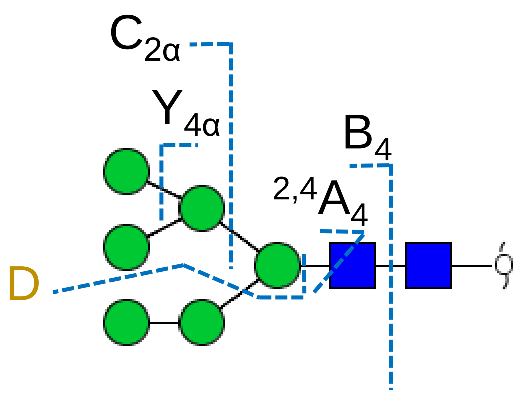

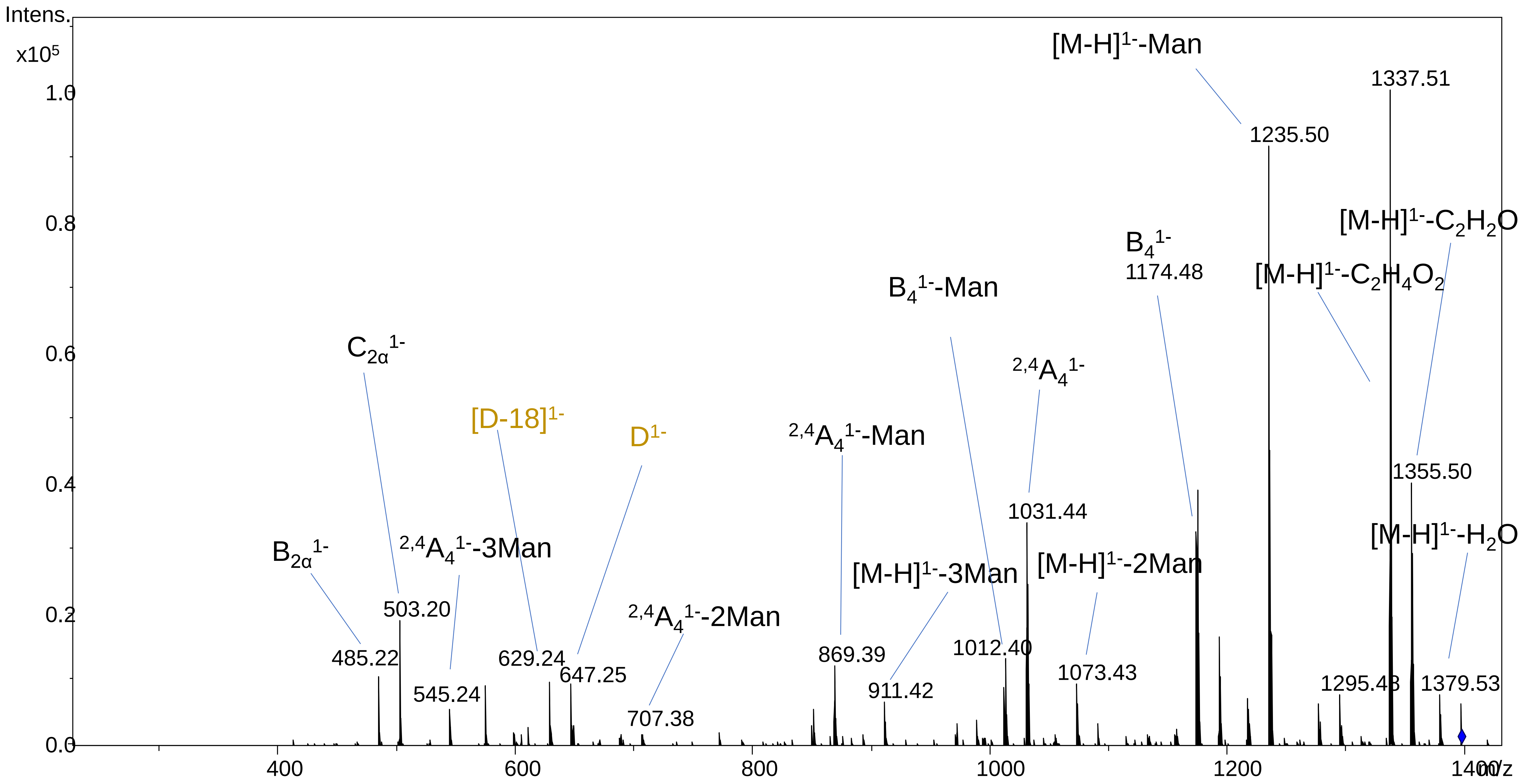

Glycan 6

H6N2P1

**Monoisotopic mass:**	**1478.47 Da**
**Charge observed:**	**2-**
**Theoretical ion:**	***m/z* 738.23**
**Observed ion:**	***m/z* 738.24**
**Mass deviation:**	***m/z* 0.01**
**Retention time:**	**32.8 min**

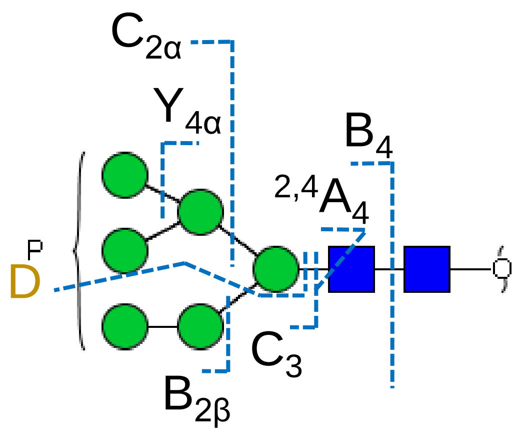

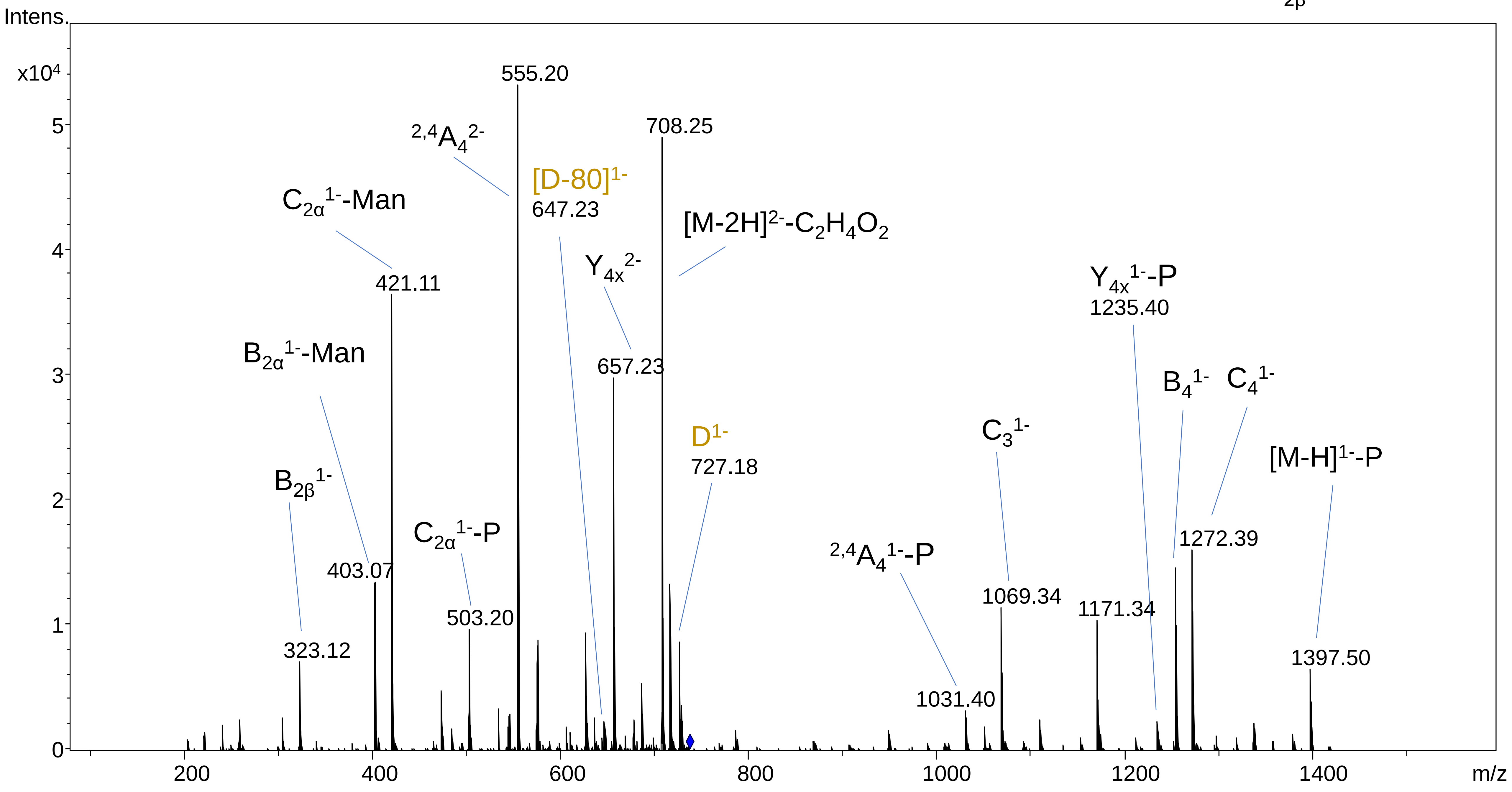

Glycan 7

H7N2

**Monoisotopic mass:**	**1560.55 Da**
**Charge observed:**	**2-**
**Theoretical ion:**	***m/z* 779.27**
**Observed ion:**	***m/z* 779.31**
**Mass deviation:**	***m/z* 0.04**
**Retention time:**	**28.9 min**

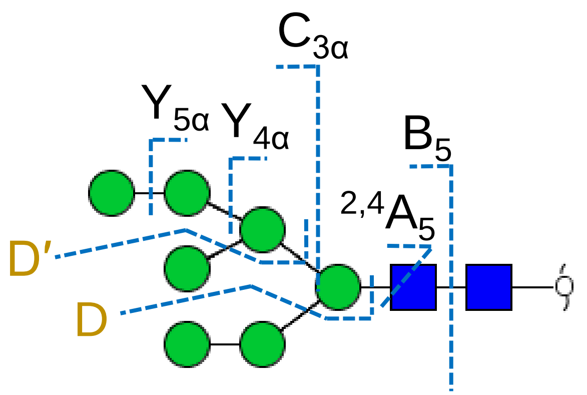

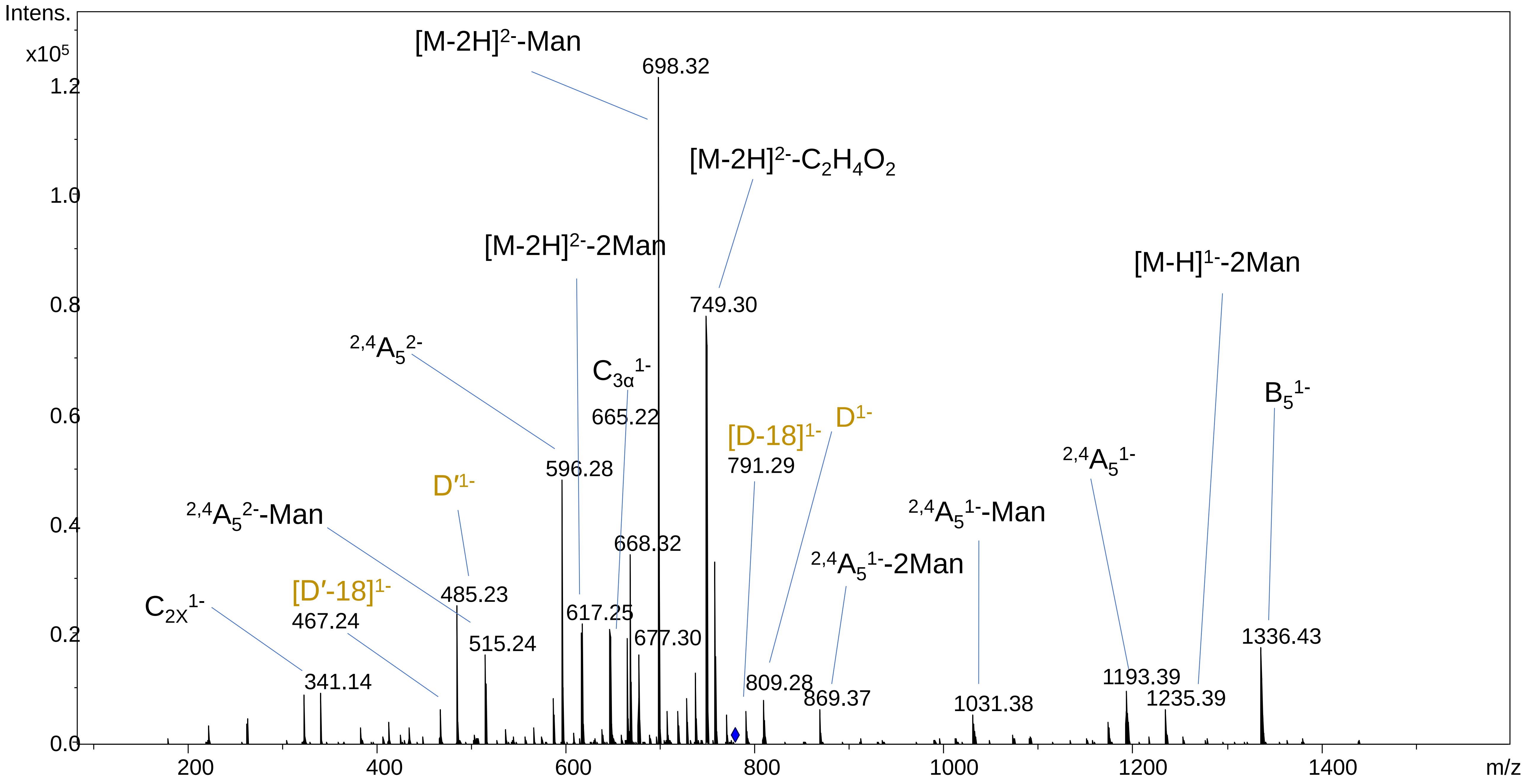

Glycan 8

H7N2P1

**Monoisotopic mass:**	**1640.52 Da**
**Charge observed:**	**2-**
**Theoretical ion:**	***m/z* 819.25**
**Observed ion:**	***m/z* 819.28**
**Mass deviation:**	***m/z* 0.03**
**Retention time:**	**32.6 min**

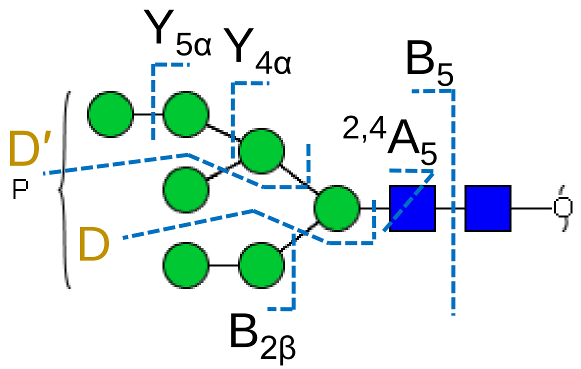

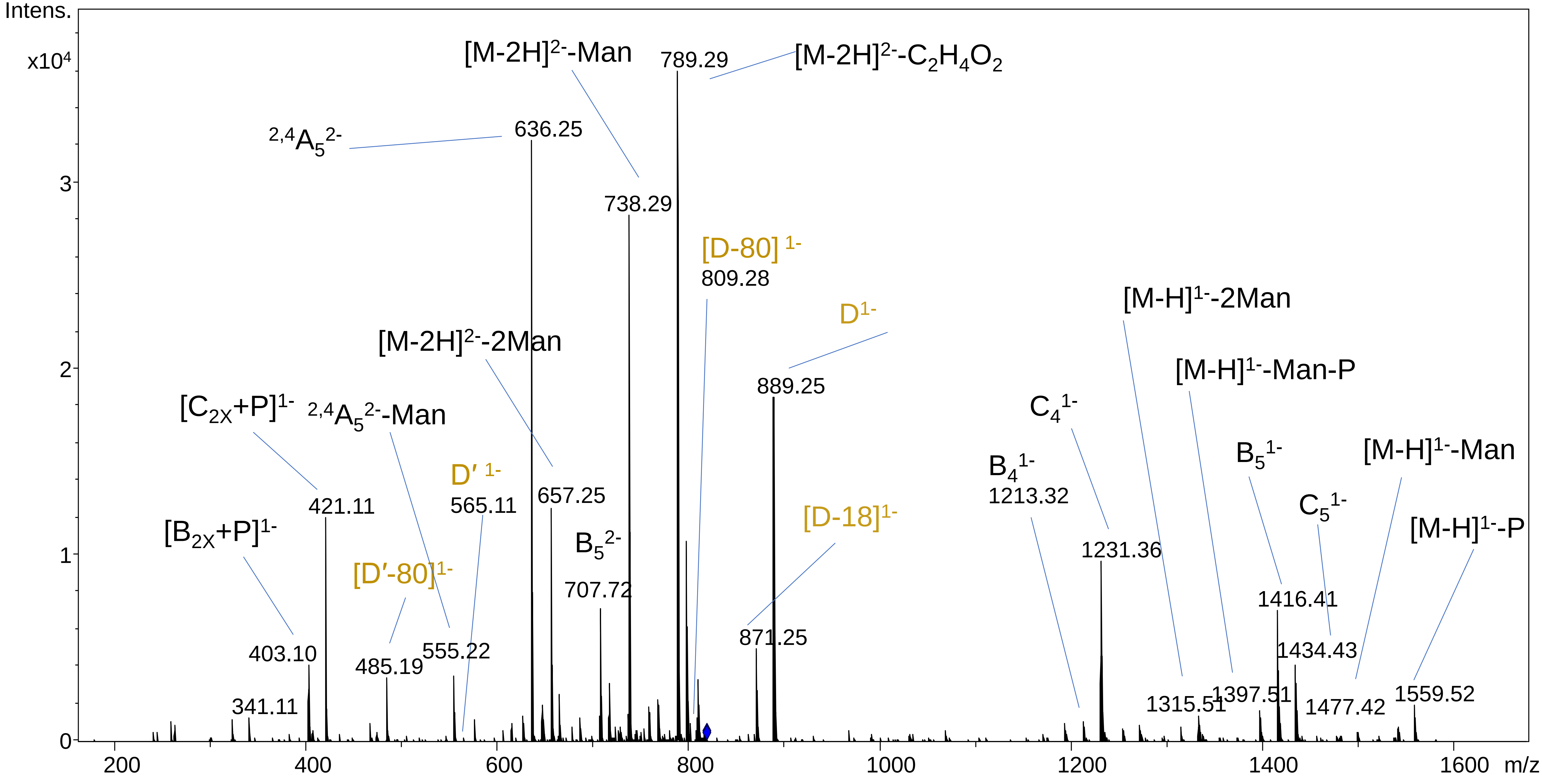

Glycan 9

H8N2

**Monoisotopic mass:**	**1722.61 Da**
**Charge observed:**	**2-**
**Theoretical ion:**	***m/z* 860.30**
**Observed ion:**	***m/z* 860.31**
**Mass deviation:**	***m/z* 0.01**
**Retention time:**	**29.0 min**

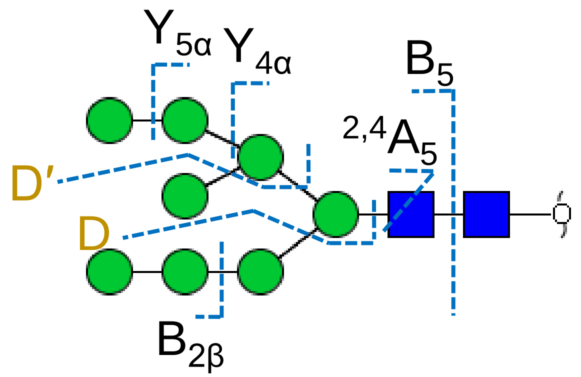

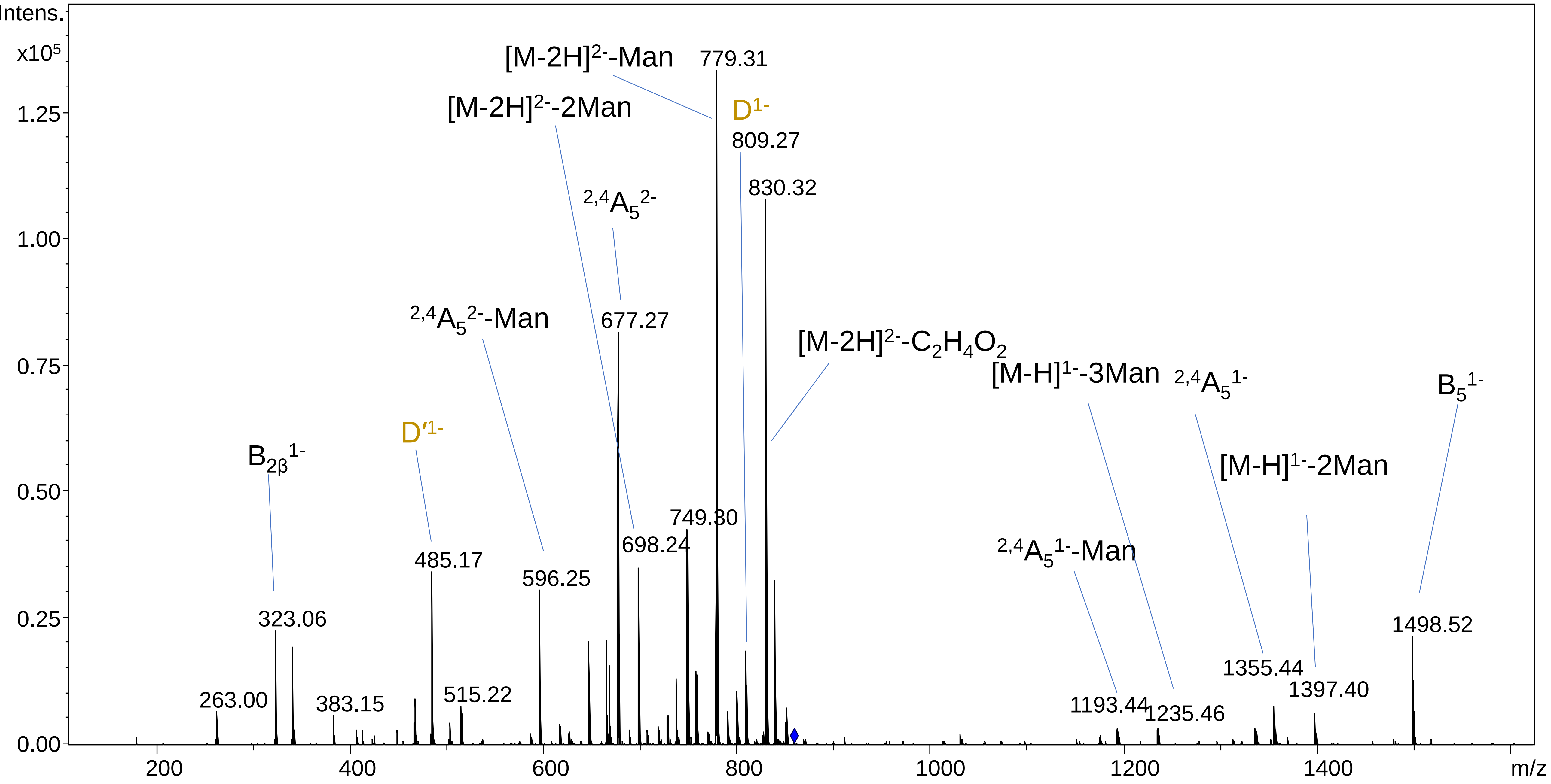

Glycan 10

H9N2

**Monoisotopic mass:**	**1884.66 Da**
**Charge observed:**	**2-**
**Theoretical ion:**	***m/z* 941.32**
**Observed ion:**	***m/z* 941.35**
**Mass deviation:**	***m/z* 0.03**
**Retention time:**	**29.4 min**

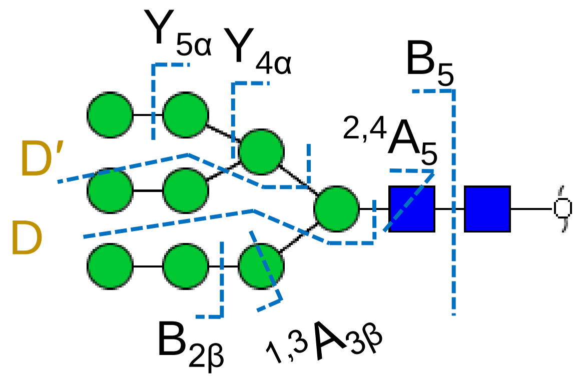

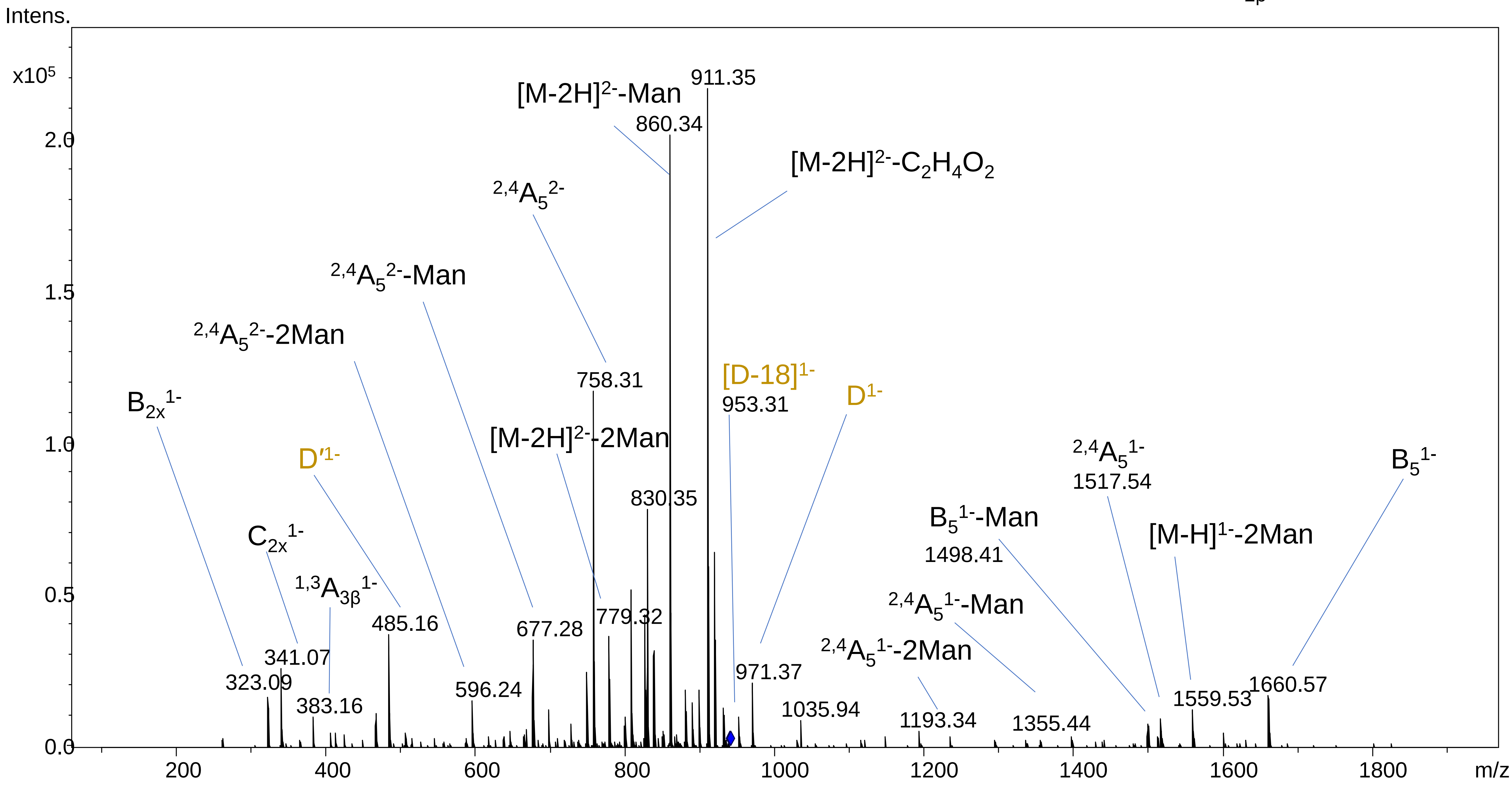

Glycan 11

H5N3S1

**Monoisotopic mass:**	**1730.63 Da**
**Charge observed:**	**2-**
**Theoretical ion:**	***m/z* 864.31**
**Observed ion:**	***m/z* 864.32**
**Mass deviation:**	***m/z* 0.01**
**Retention time:**	**35.4 min**

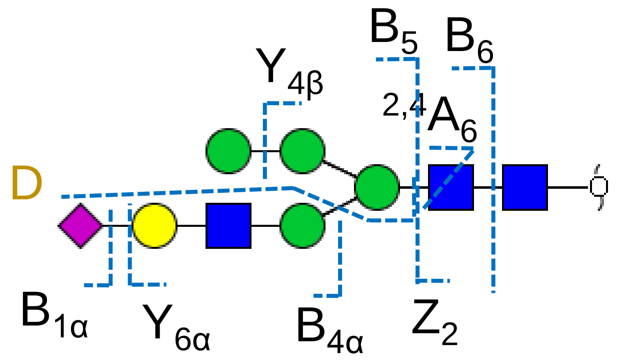

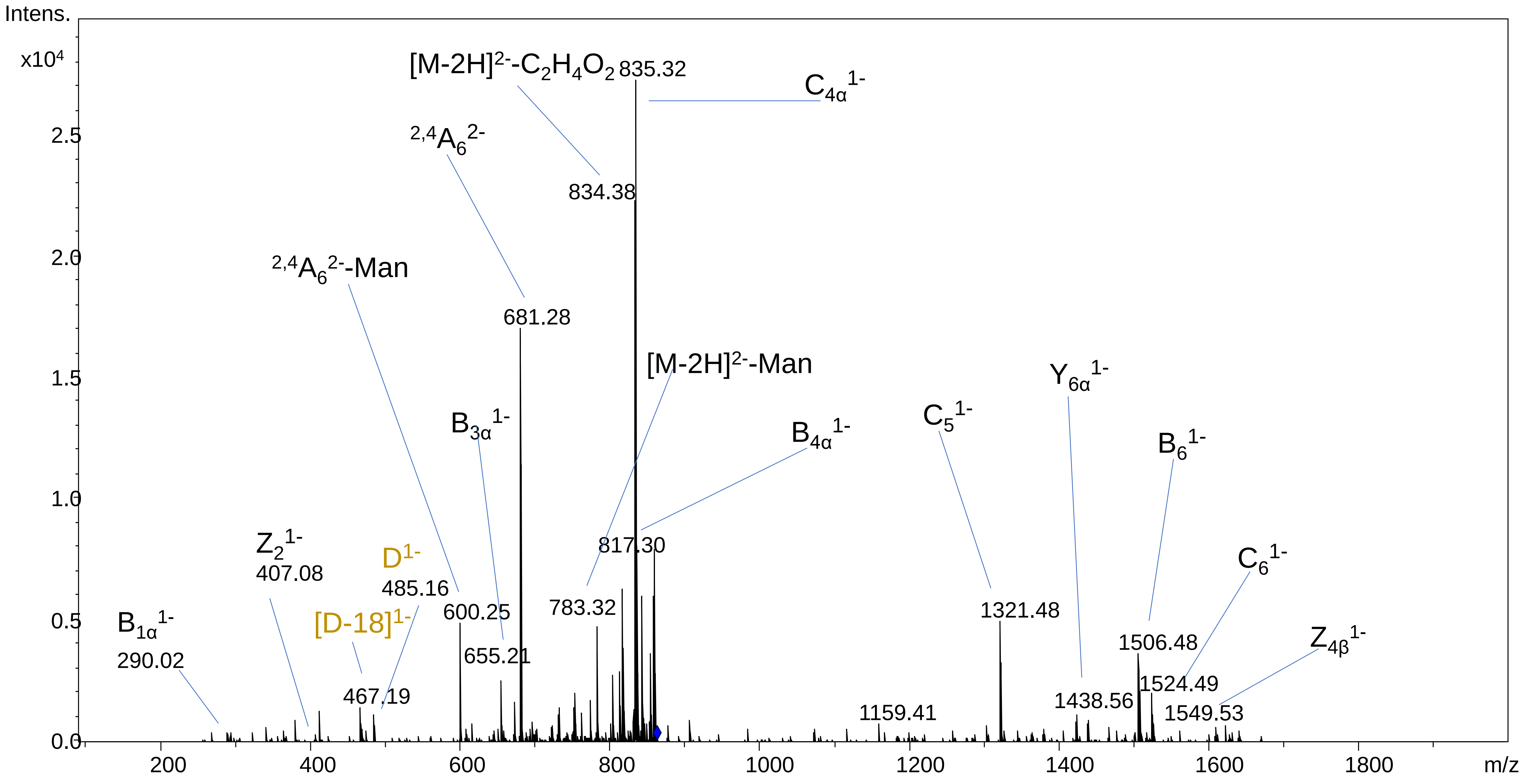

Glycan 12

H5N3F1S1

**Monoisotopic mass:**	**1875.68 Da**
**Charge observed:**	**2-**
**Theoretical ion:**	***m/z* 937.34**
**Observed ion:**	***m/z* 937.37**
**Mass deviation:**	***m/z* 0.03**
**Retention time:**	**41.7 min**

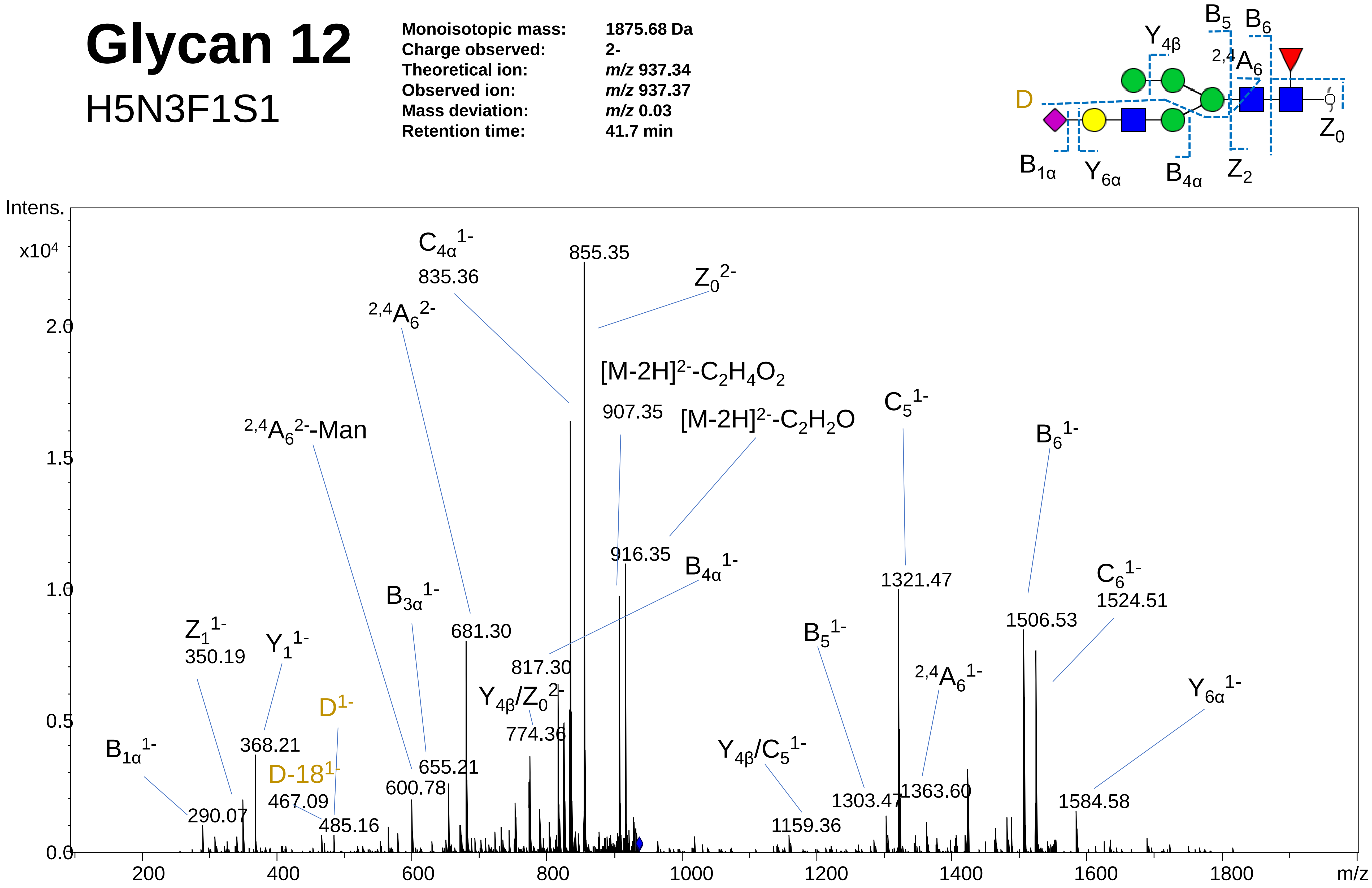

Glycan 13

H6N3S1

**Monoisotopic mass:**	**1892.68 Da**
**Charge observed:**	**2-**
**Theoretical ion:**	***m/z* 945.33**
**Observed ion:**	***m/z* 945.36**
**Mass deviation:**	***m/z* 0.03**
**Retention time:**	**38.5 min**

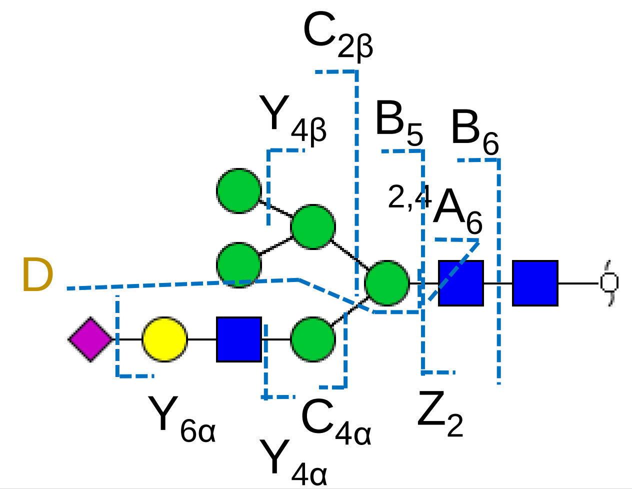

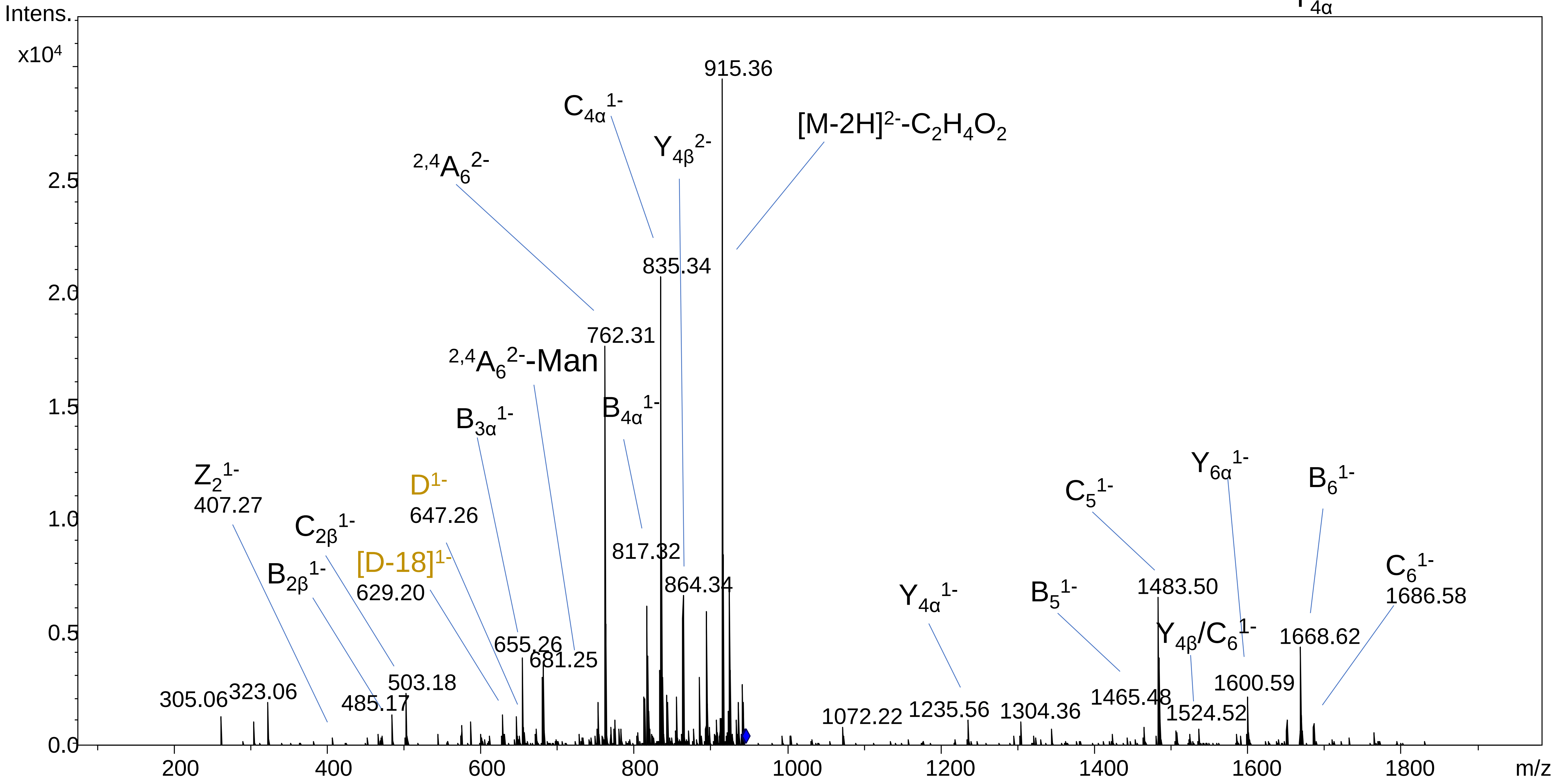

Glycan 14

H4N3F1S1

**Monoisotopic mass:**	**1714.63 Da**
**Charge observed:**	**2-**
**Theoretical ion:**	***m/z* 856.30**
**Observed ion:**	***m/z* 856.33**
**Mass deviation:**	***m/z* 0.03**
**Retention time:**	**43.8 min**

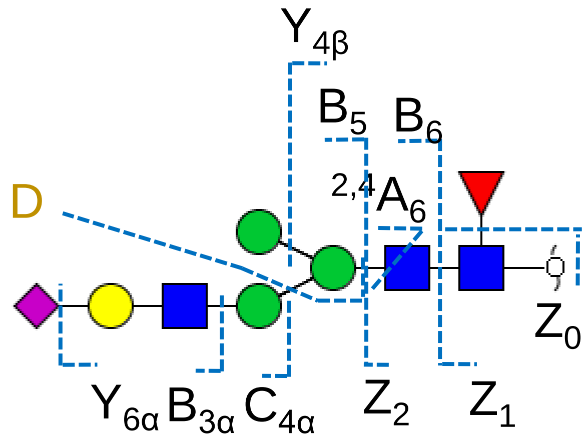

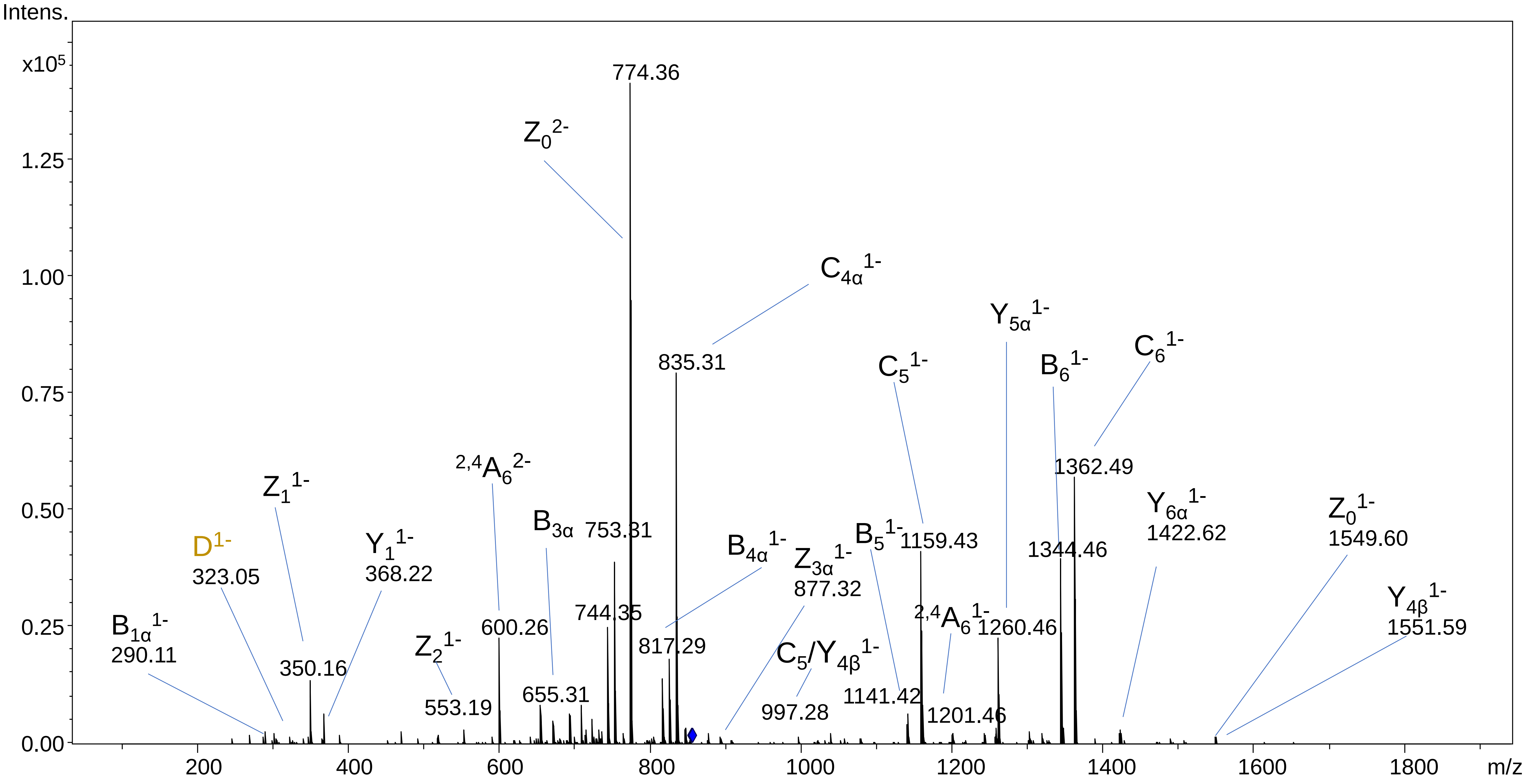

Glycan 15

H5N4F1

Monoisotopic mass: 1788.67 Da
Charge observed: 2-
Theoretical ion: *m/z* 893.33
Observed ion: *m/z* 893.37
Mass deviation: *m/z* 0.04
Retention time:

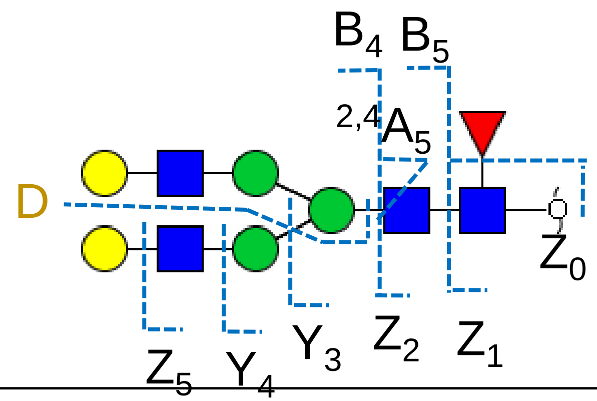

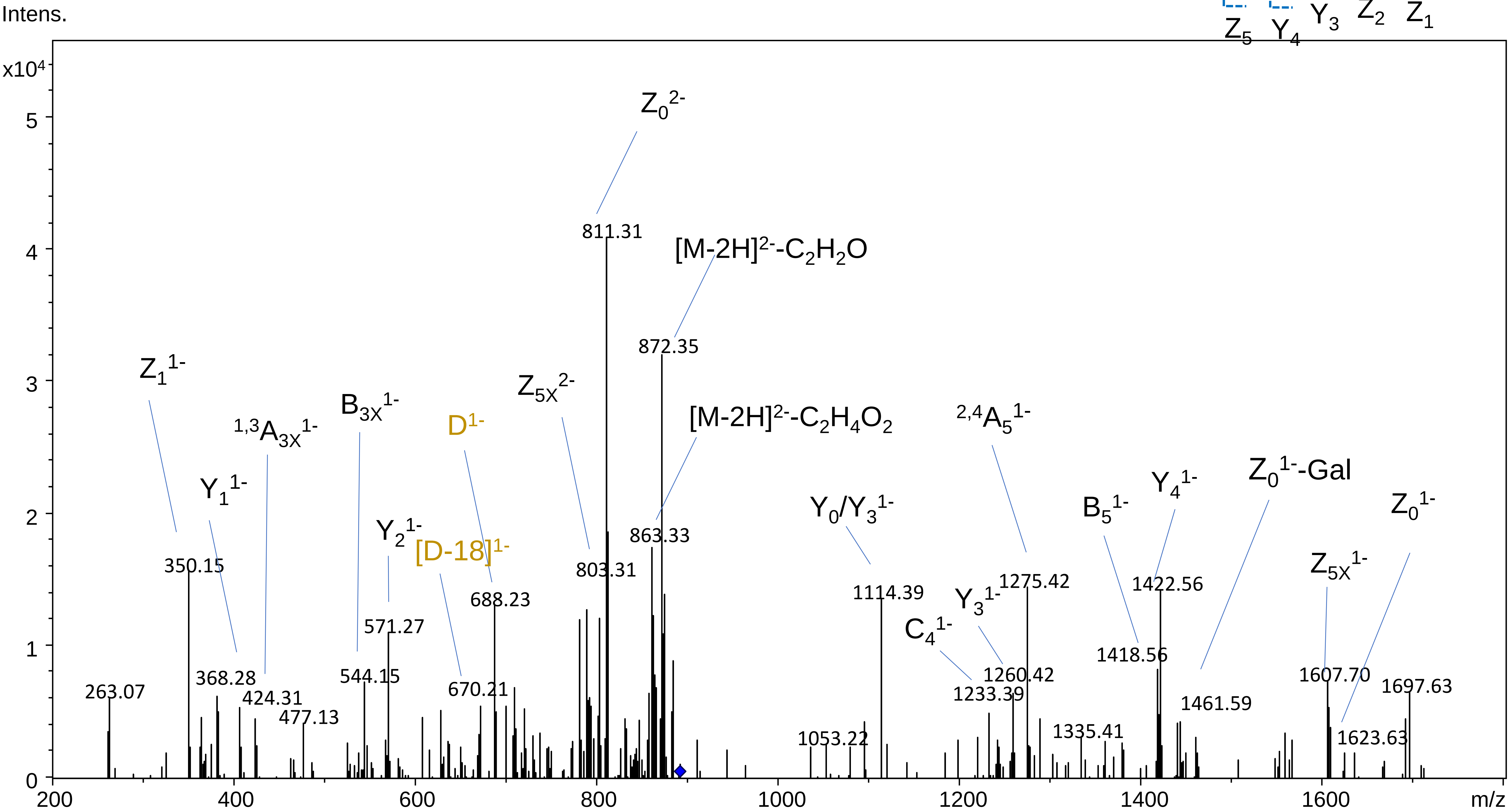

Glycan 16

H4N4F1S1

**Monoisotopic mass:**	**1917.70 Da**
**Charge observed:**	**2-**
**Theoretical ion:**	***m/z* 957.85**
**Observed ion:**	***m/z* 957.89**
**Mass deviation:**	***m/z* 0.04**
**Retention time:**	**44.7 min**

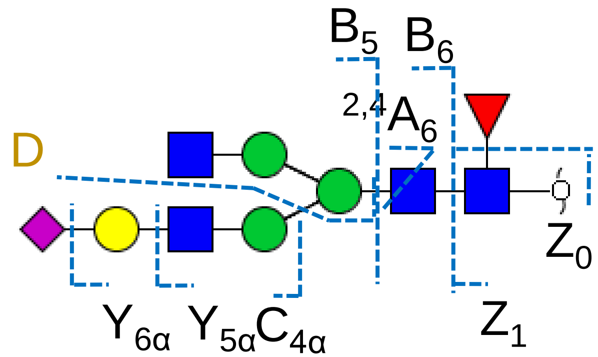

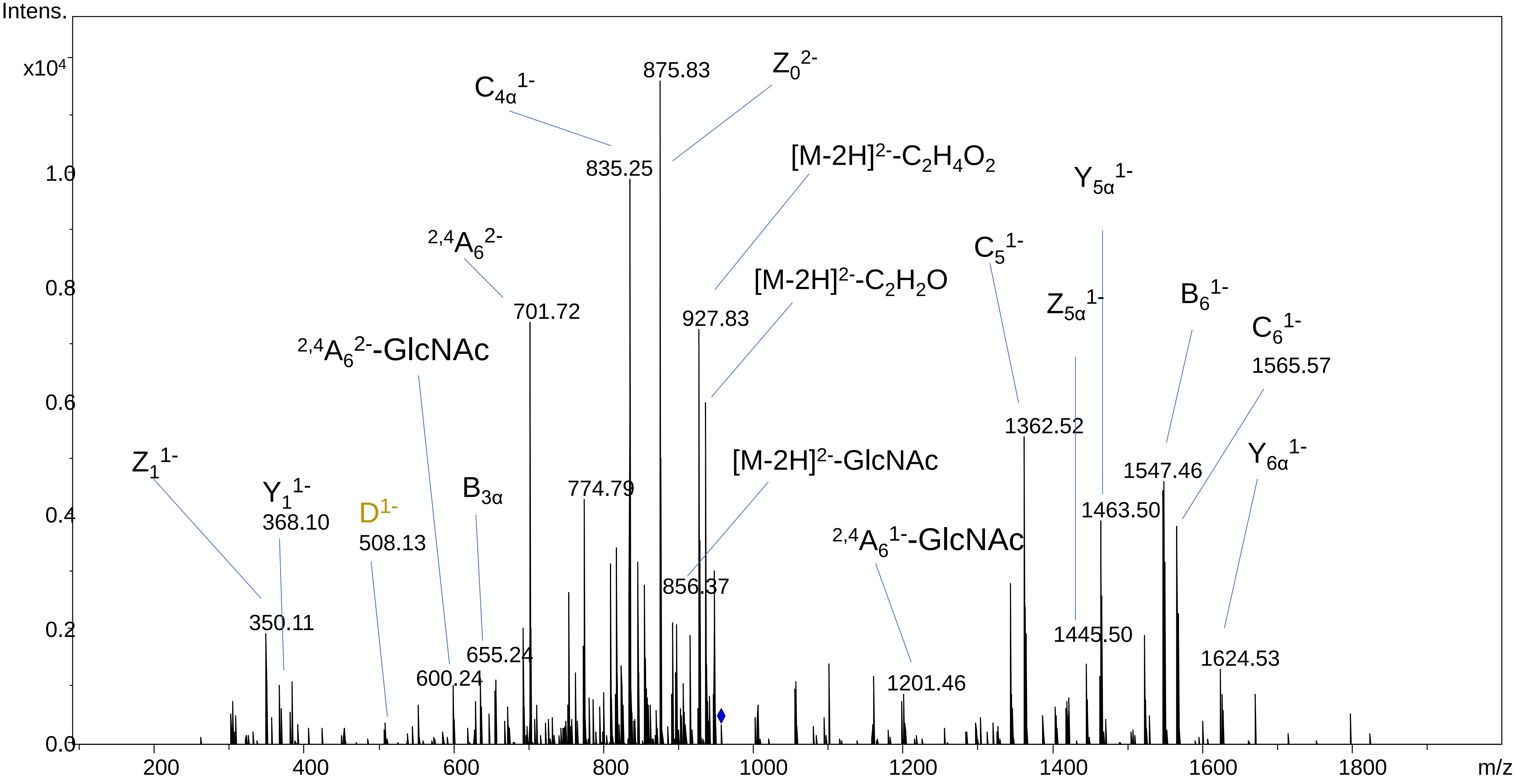

Glycan 17

H5N4S1

**Monoisotopic mass:**	**1933.70 Da**
**Charge observed:**	**2-**
**Theoretical ion:**	***m/z* 965.84**
**Observed ion:**	***m/z* 965.87**
**Mass deviation:**	***m/z* 0.03**
**Retention time:**	**40.9 min**

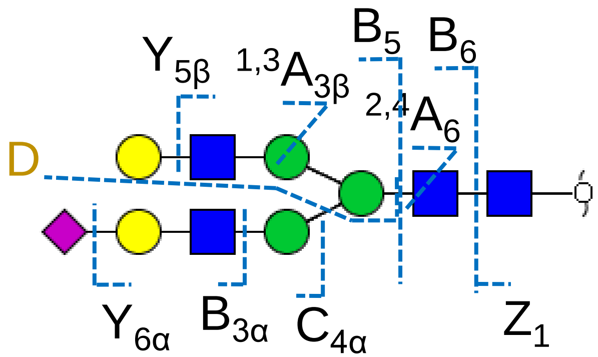

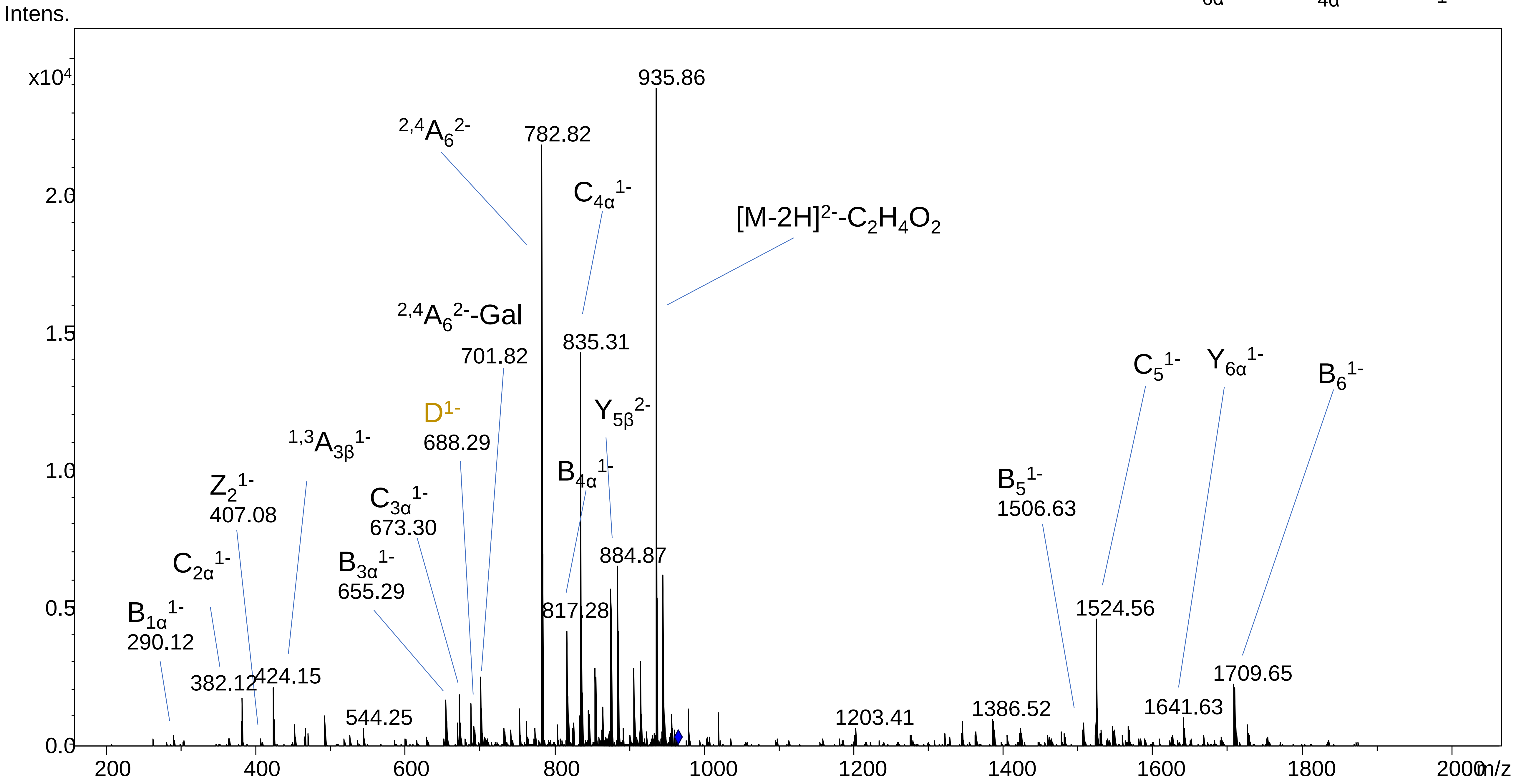

Glycan 18

H5N4F1S1

**Monoisotopic mass:**	**2079.76 Da**
**Charge observed:**	**2-**
**Theoretical ion:**	***m/z* 1038.87**
**Observed ion:**	***m/z* 1038.92**
**Mass deviation:**	***m/z* 0.05**
**Retention time:**	**46.7 min**

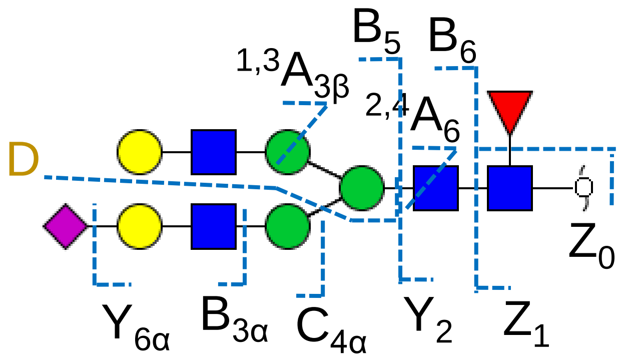

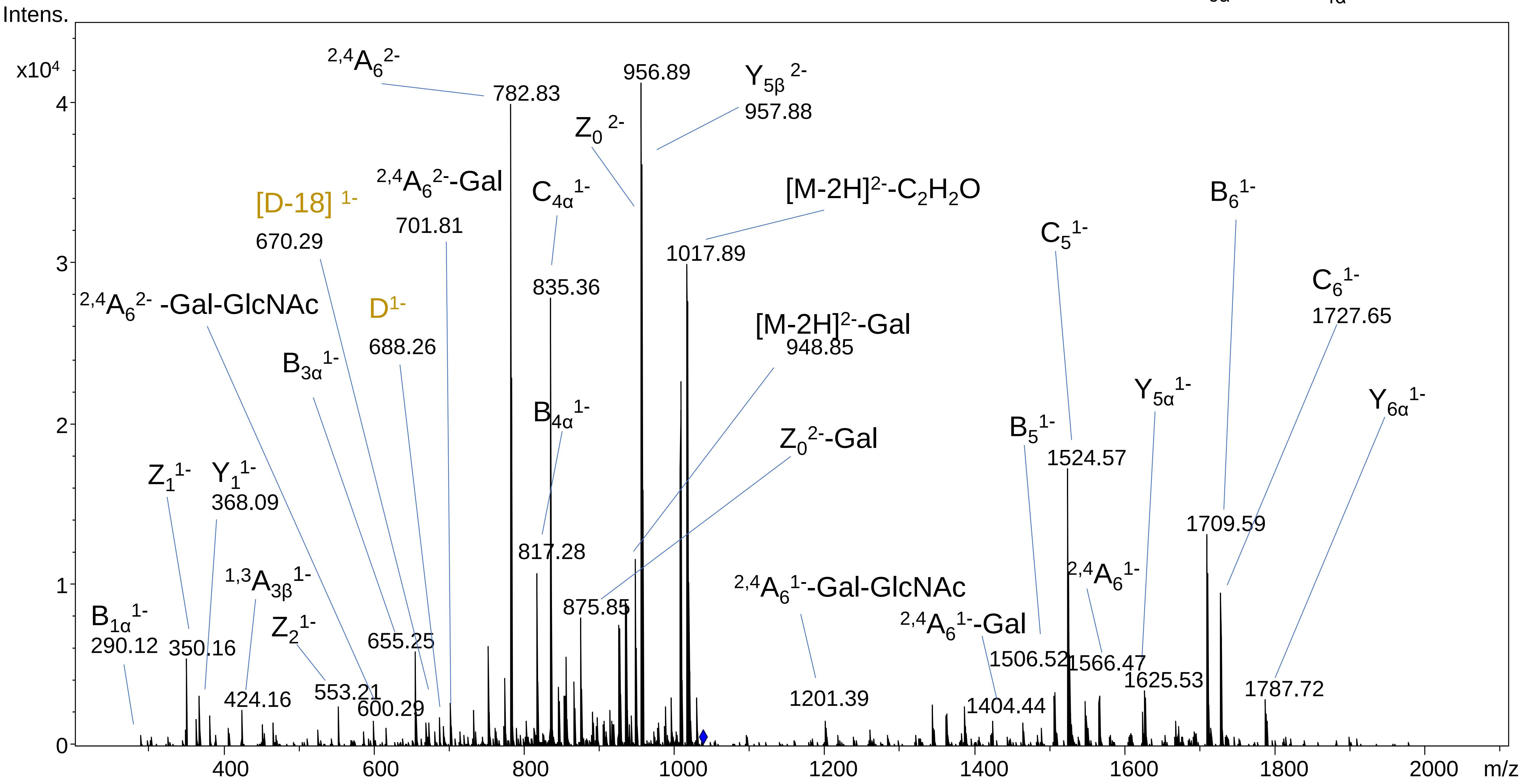

Glycan 19

H4N5F1S1

**Monoisotopic mass:**	**2200.78 Da**
**Charge observed:**	**2-**
**Theoretical ion:**	***m/z* 1059.39**
**Observed ion:**	***m/z* 1059.39**
**Mass deviation:**	***m/z* 0.00**
**Retention time:**	**37.0 min**

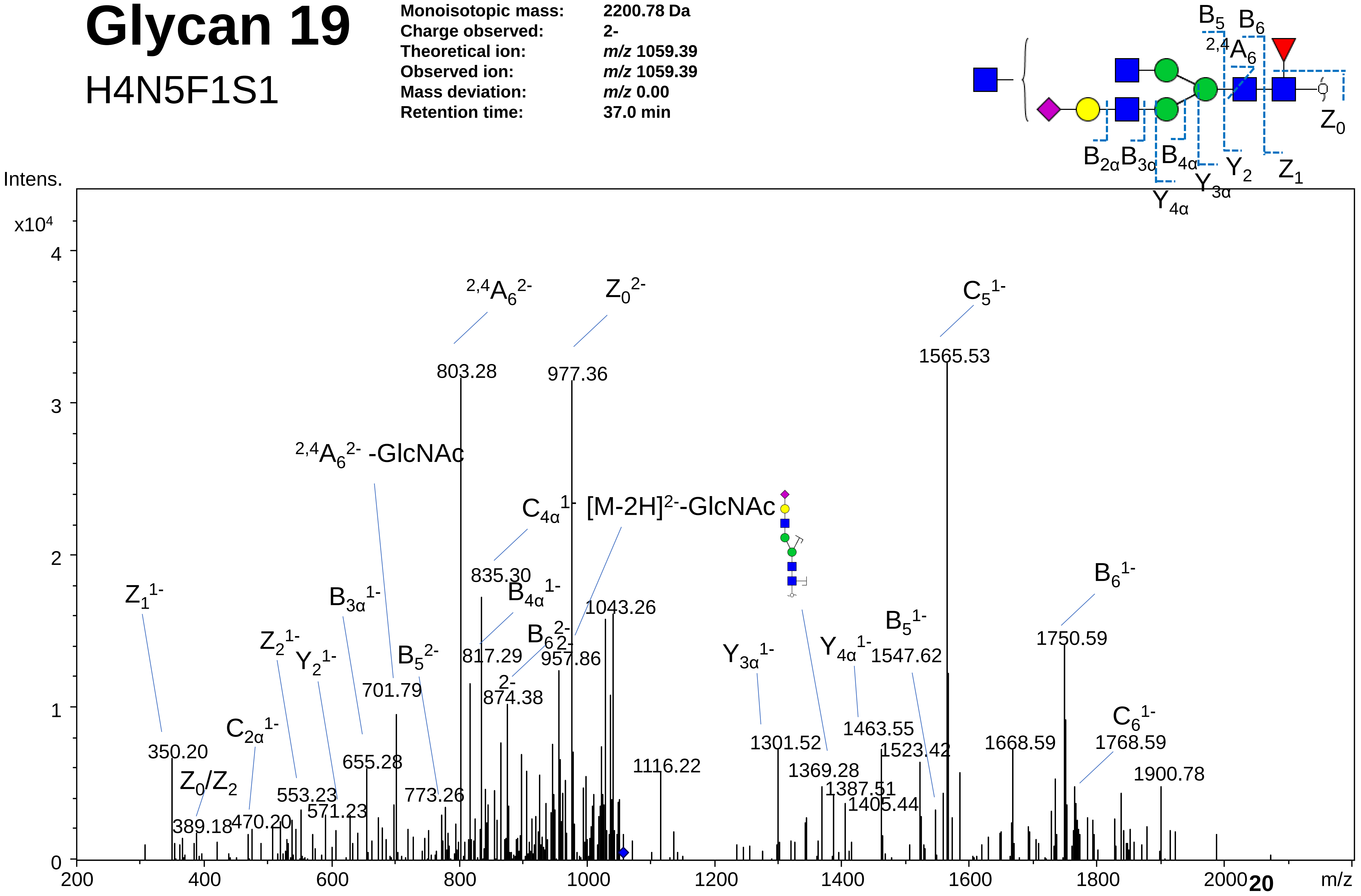

Glycan 20a

H5N4S2

**Monoisotopic mass:**	**2224.80 Da**
**Charge observed:**	**2-**
**Theoretical ion:**	***m/z* 1111.42**
**Observed ion:**	***m/z* 1111.43**
**Mass deviation:**	***m/z* 0.01**
**Retention time:**	**44.6 min**

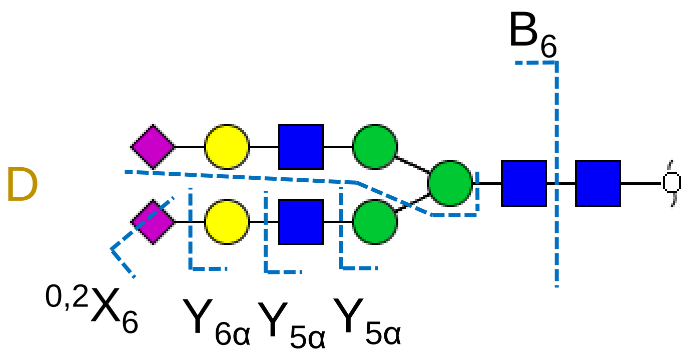

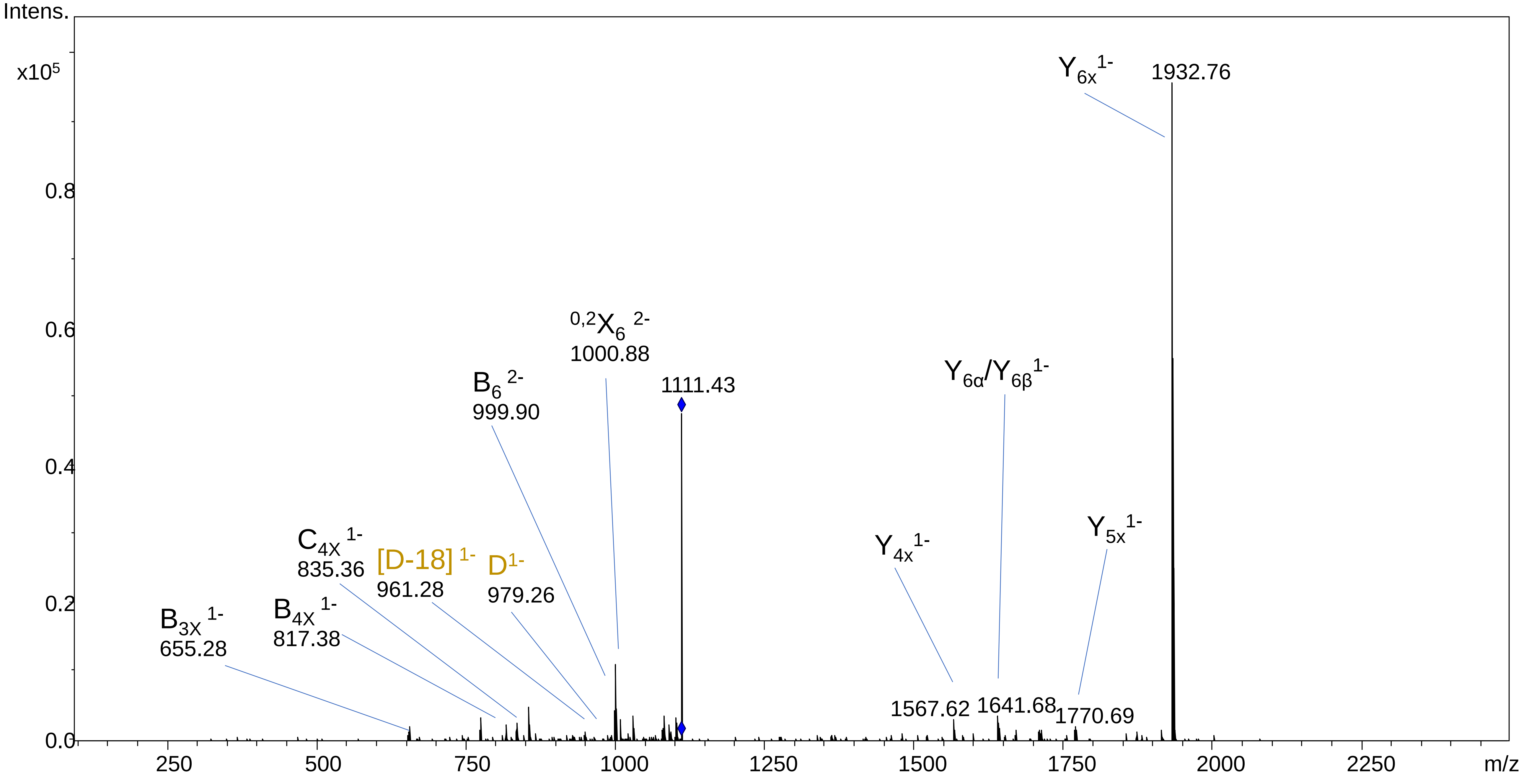

Glycan 20b

H5N4S2

**Monoisotopic mass:**	**2224.80 Da**
**Charge observed:**	**2-**
**Theoretical ion:**	***m/z* 1111.42**
**Observed ion:**	***m/z* 1111.43**
**Mass deviation:**	***m/z* 0.01**
**Retention time:**	**54.0 min**

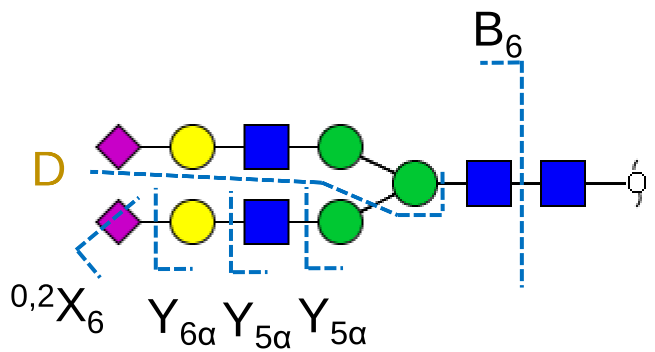

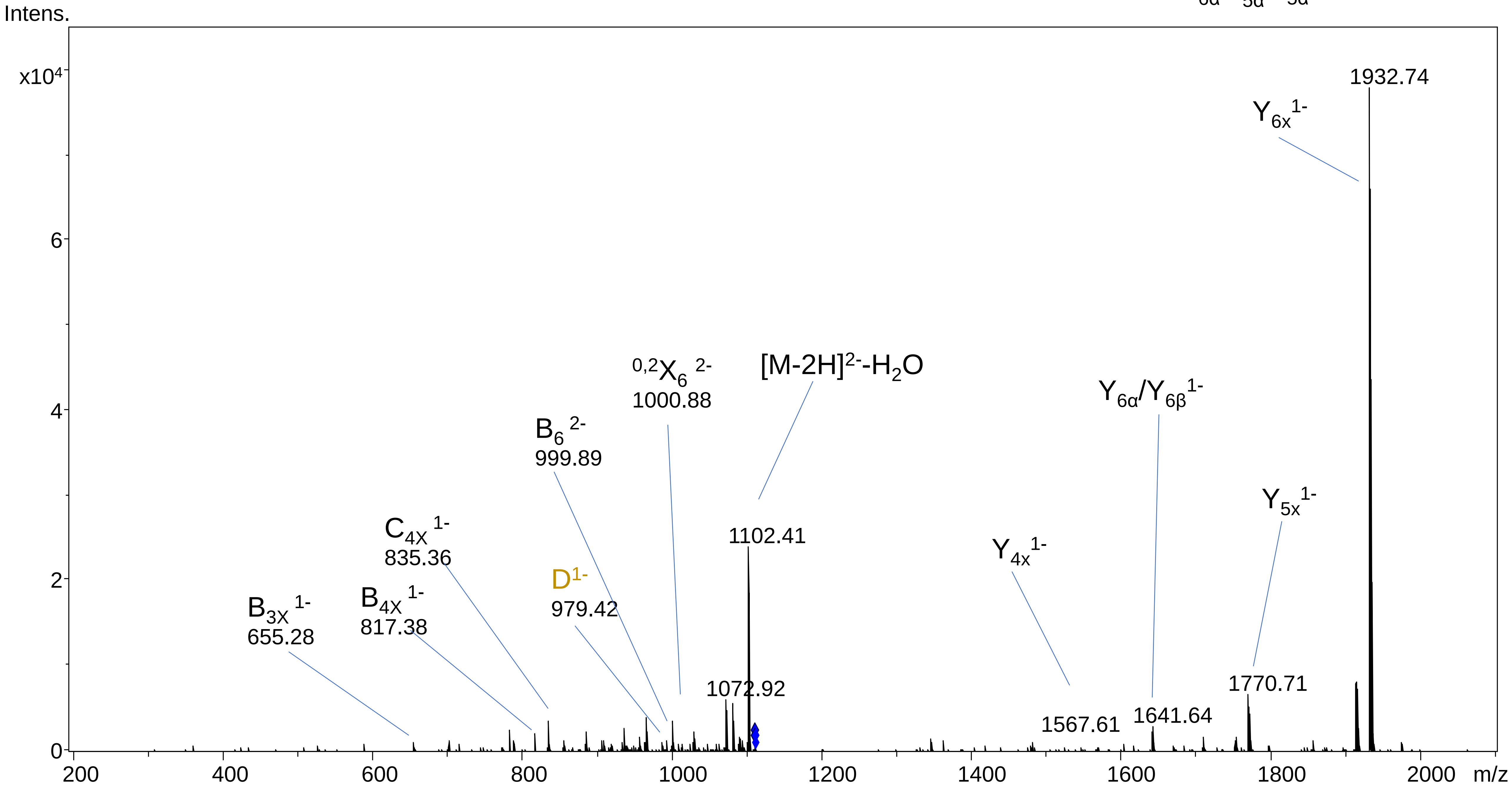

Glycan 21

H5N5F1S1

**Monoisotopic mass:**	**2282.83 Da**
**Charge observed:**	**2-**
**Theoretical ion:**	***m/z* 1140.41**
**Observed ion:**	***m/z* 1140.39**
**Mass deviation:**	***m/z* 0.02**
**Retention time:**	**37.3 min**

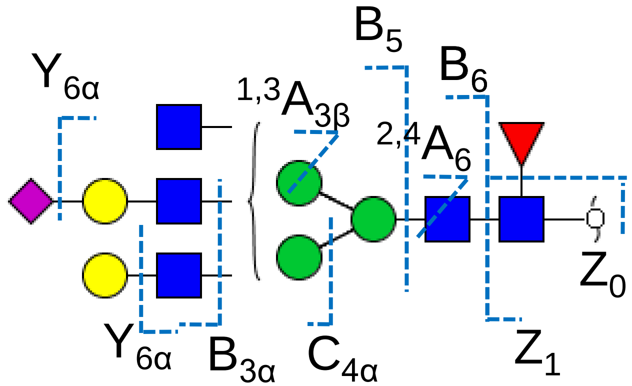

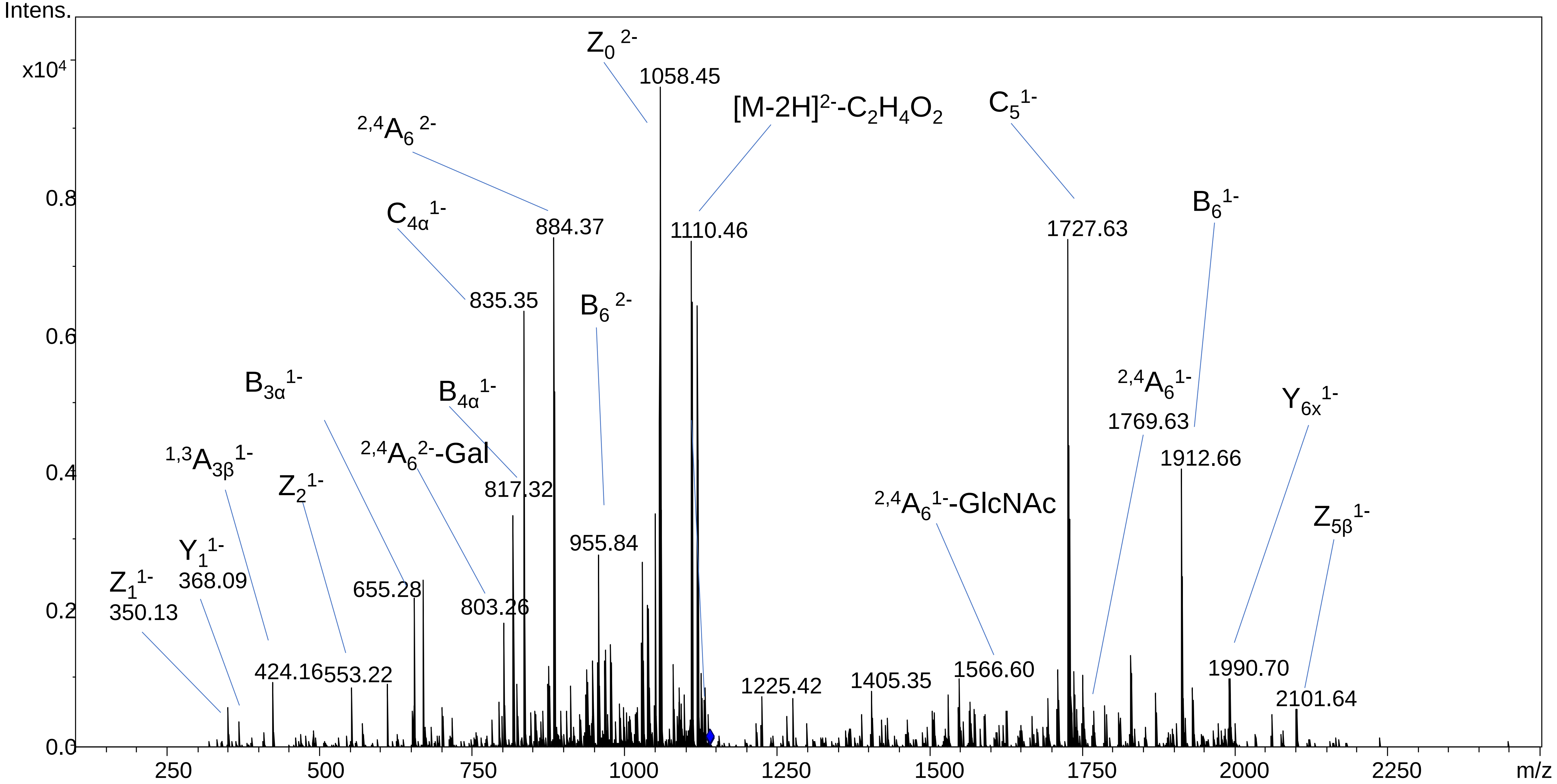

Glycan 22

H5N4S2F1

**Monoisotopic mass:**	**2370.86 Da**
**Charge observed:**	**2-**
**Theoretical ion:**	***m/z* 1184.42**
**Observed ion:**	***m/z* 1184.46**
**Mass deviation:**	***m/z* 0.04**
**Retention time:**	**49.9 min**

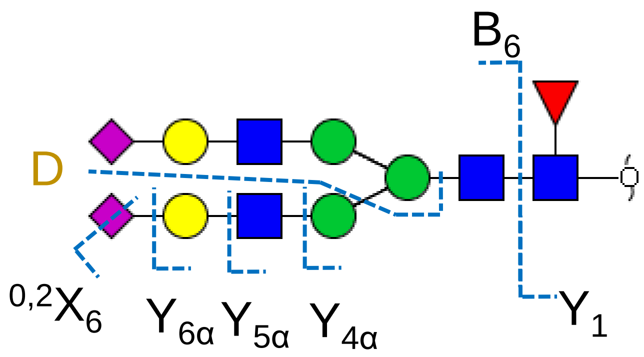

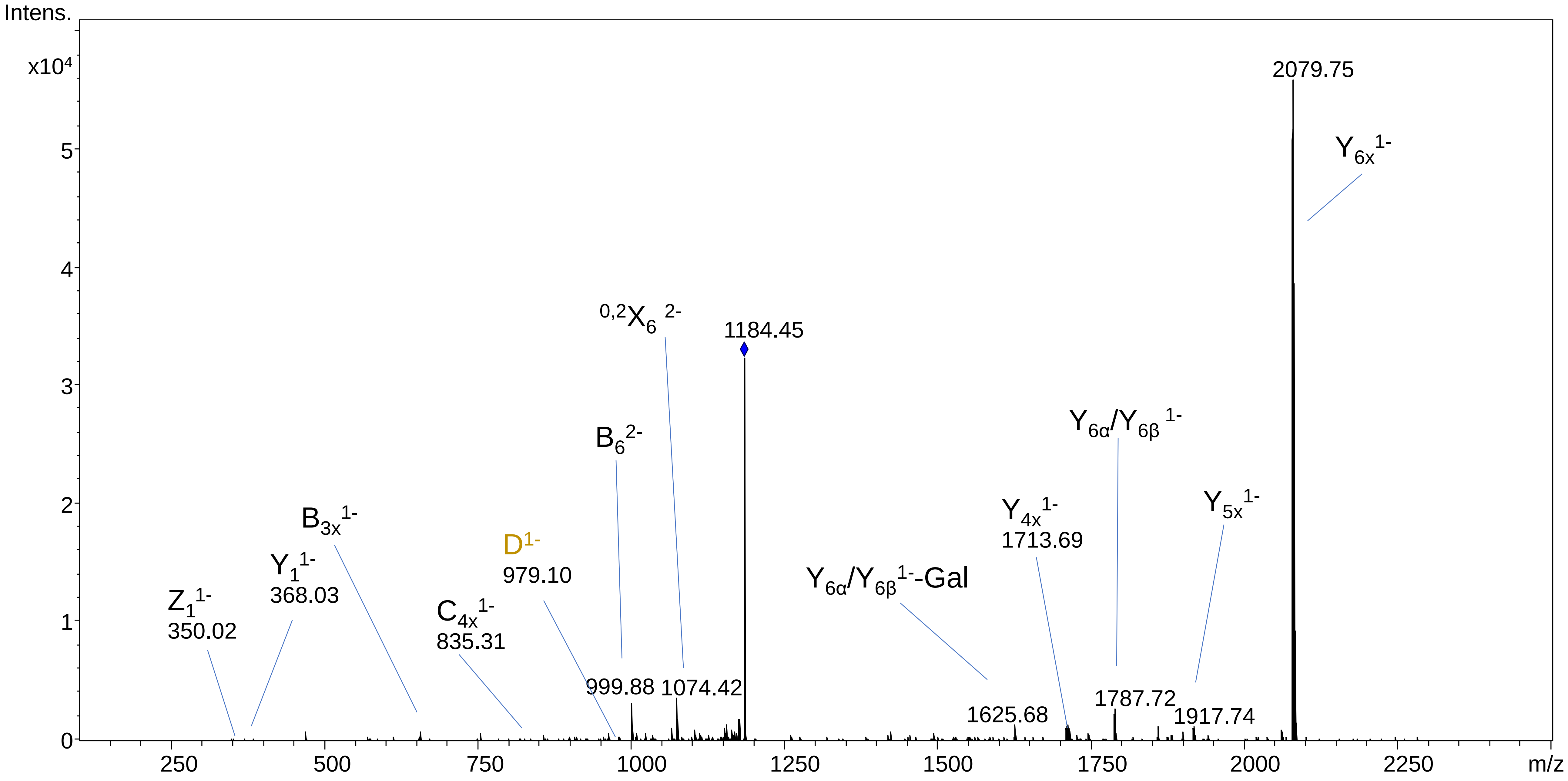

Glycan 23

H6N5F1S1

Monoisotopic mass: **2444.89 Da**
Charge observed: **2-**
Theoretical ion: ***m/z* 1221.45**
Observed ion: ***m/z* 1221.44**
Mass deviation: ***m/z* 0.01**
Retention time: **47.9 min**

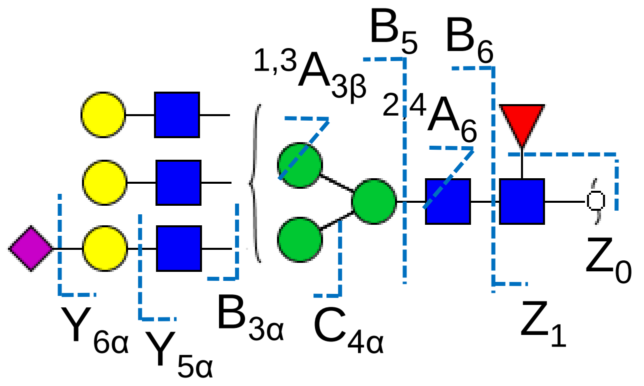

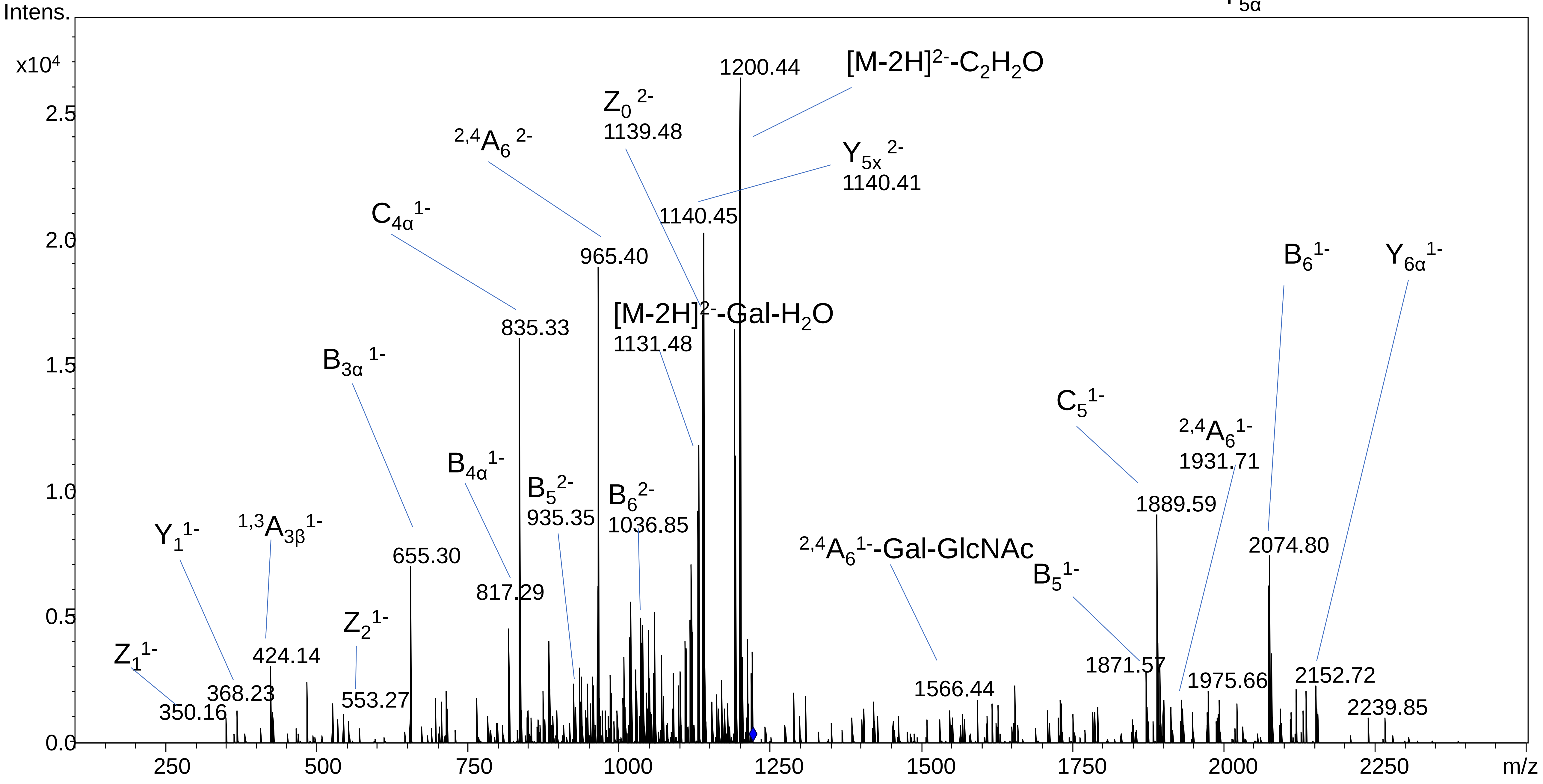

Glycan 24

H5N5F1S2

Monoisotopic mass: 2572.96 Da
Charge observed: 2-
Theoretical ion: *m/z* 1285.96
Observed ion: *m/z* 1285.96
Mass deviation: *m/z* 0.00
Retention time: 43.8 min

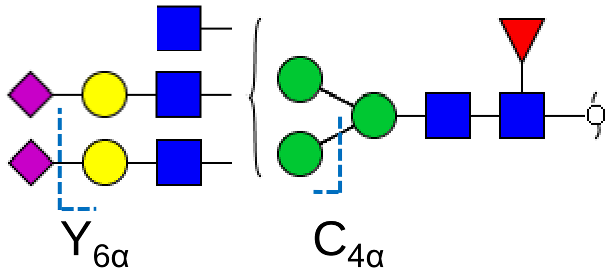

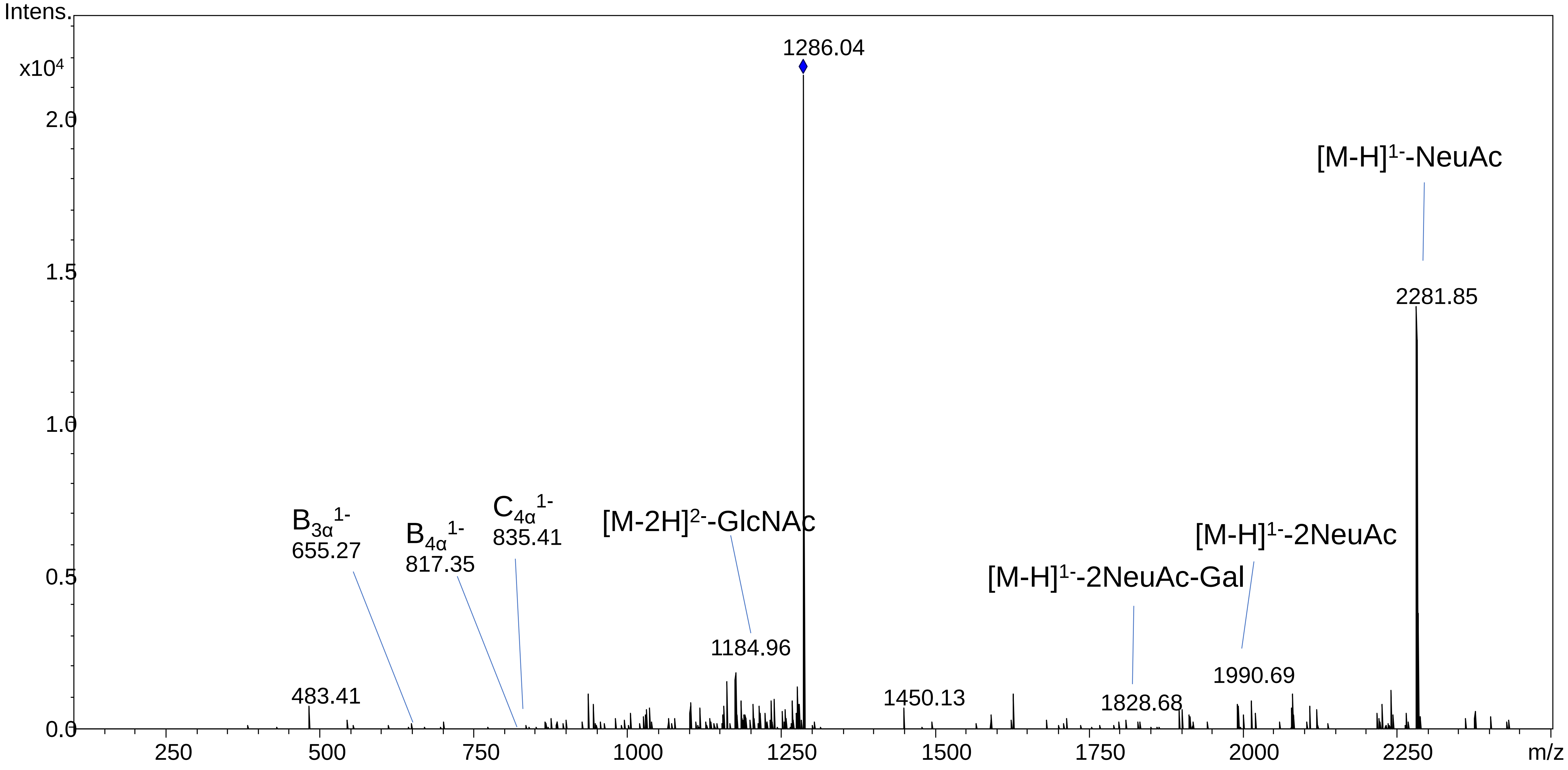

Glycan 25

H6N5S2

Monoisotopic mass: 2589.93 Da
Charge observed: 2-
Theoretical ion: *m/z* 1293.96
Observed ion: *m/z* 1293.96
Mass deviation: *m/z* 0.00
Retention time: 55.4 min

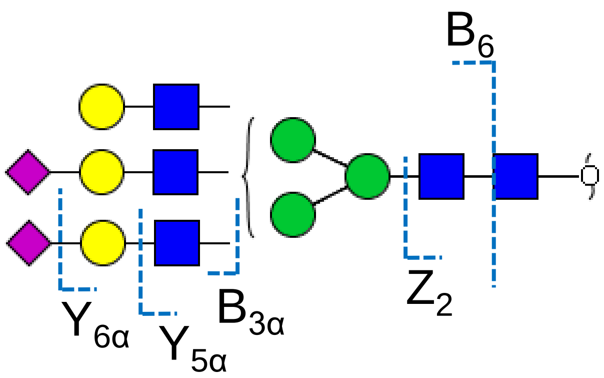

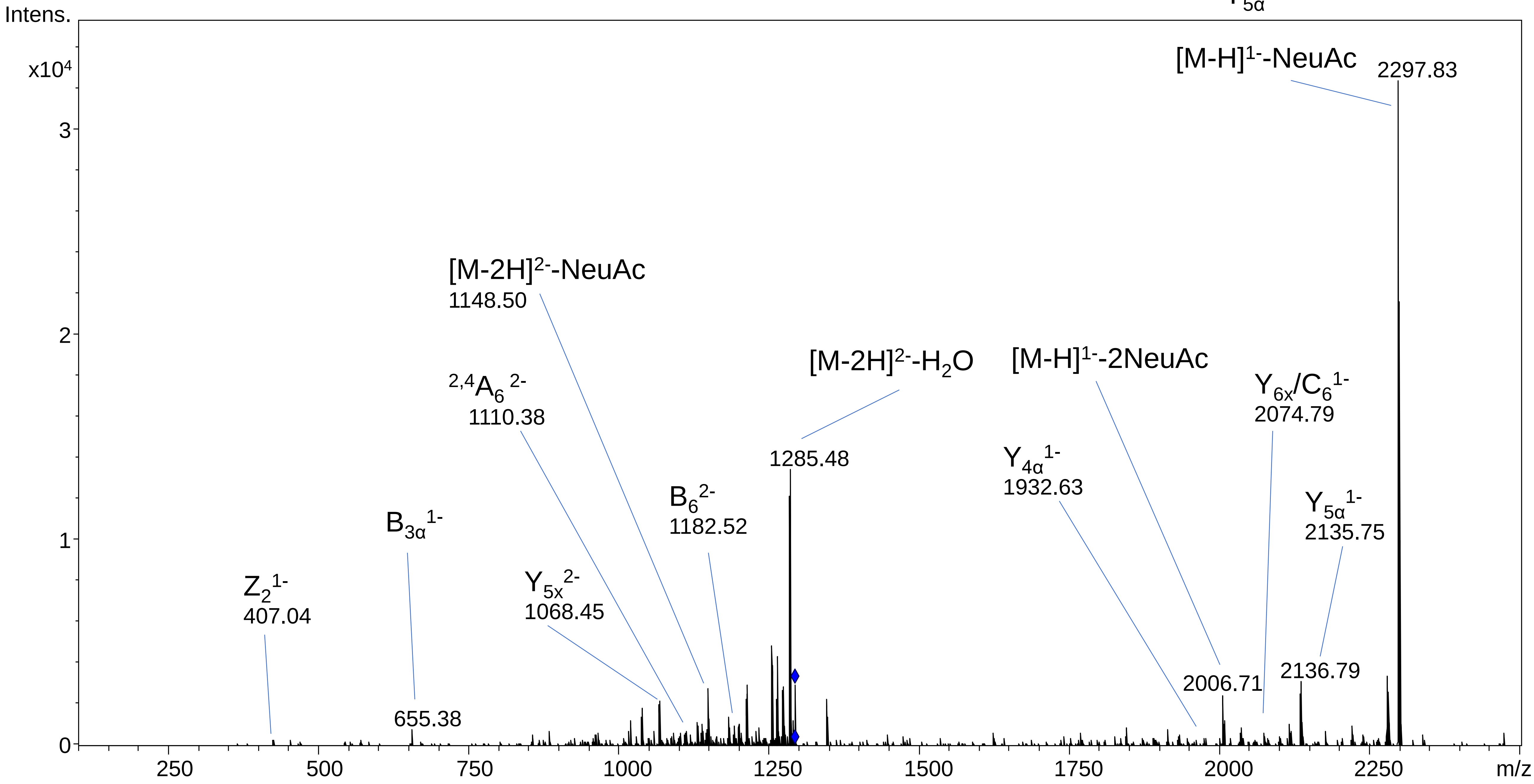

Glycan 26a

H6N5S3

**Monoisotopic mass:**	**2881.03 Da**
**Charge observed:**	**3-**
**Theoretical ion:**	***m/z* 959.34**
**Observed ion:**	***m/z* 959.36**
**Mass deviation:**	***m/z* 0.02**
**Retention time:**	**58.8 min**

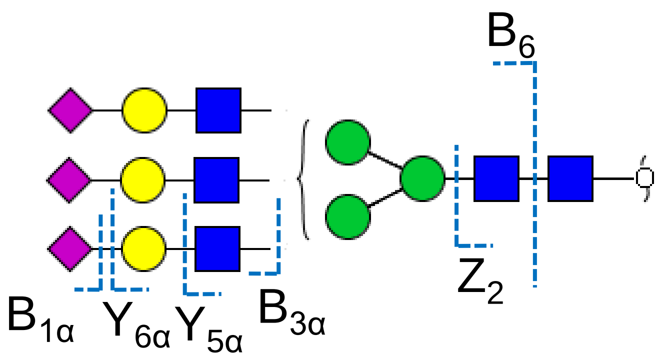

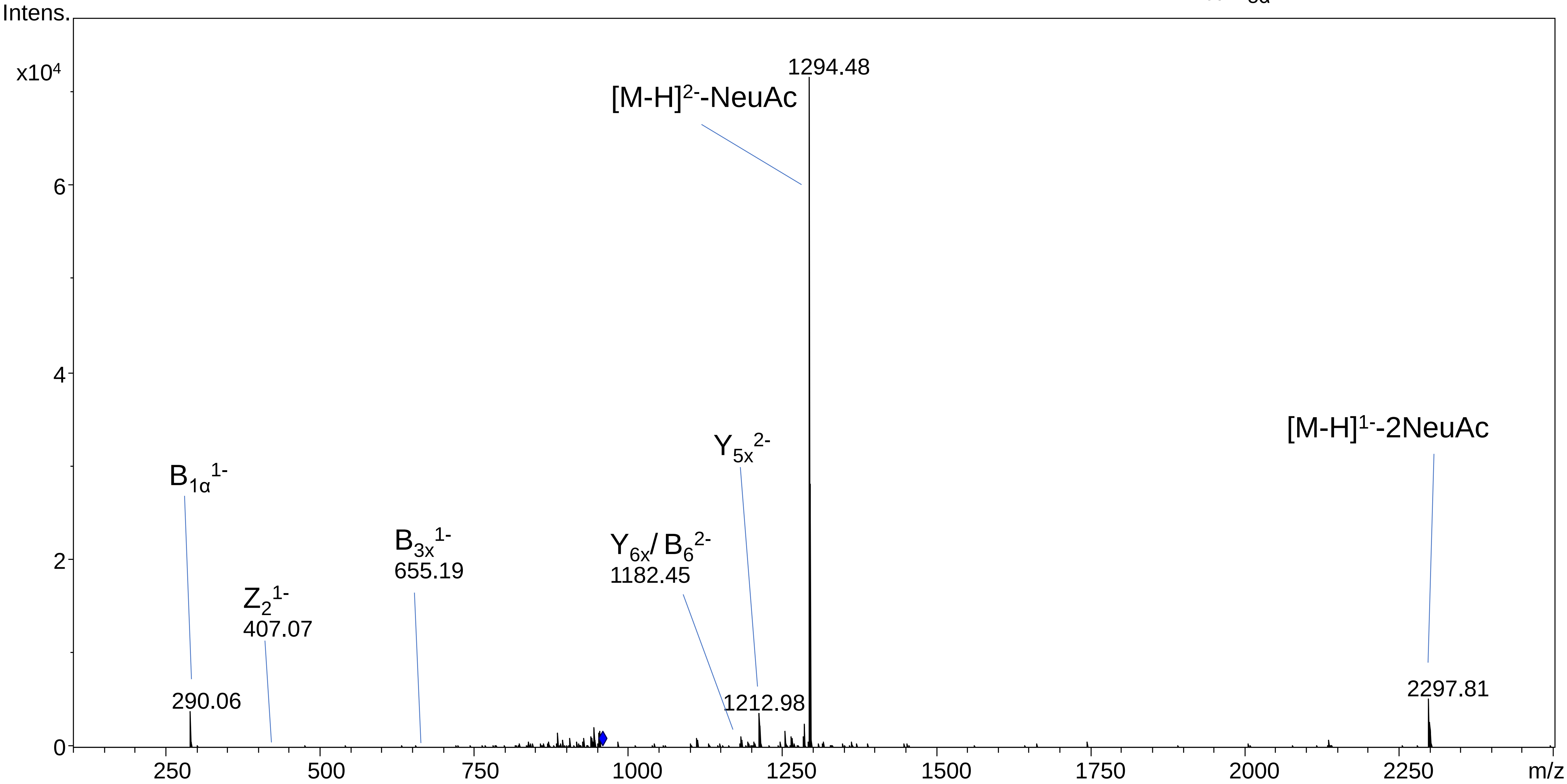

Glycan 26b

H6N5S3

Monoisotopic mass: 2881.03 Da
Charge observed: 3-
Theoretical ion: *m/z* 959.34
Observed ion: *m/z* 959.36
Mass deviation: *m/z* 0.02
Retention time: 67.0 min

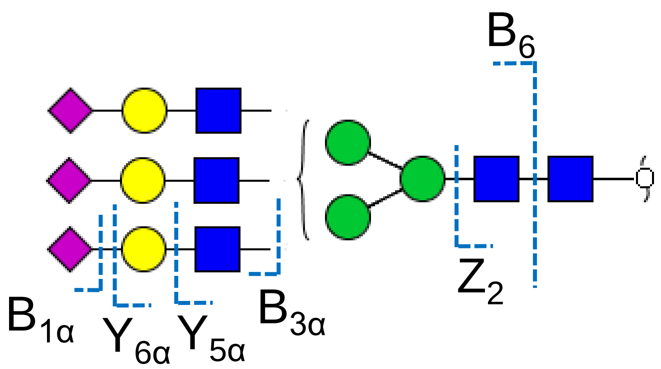

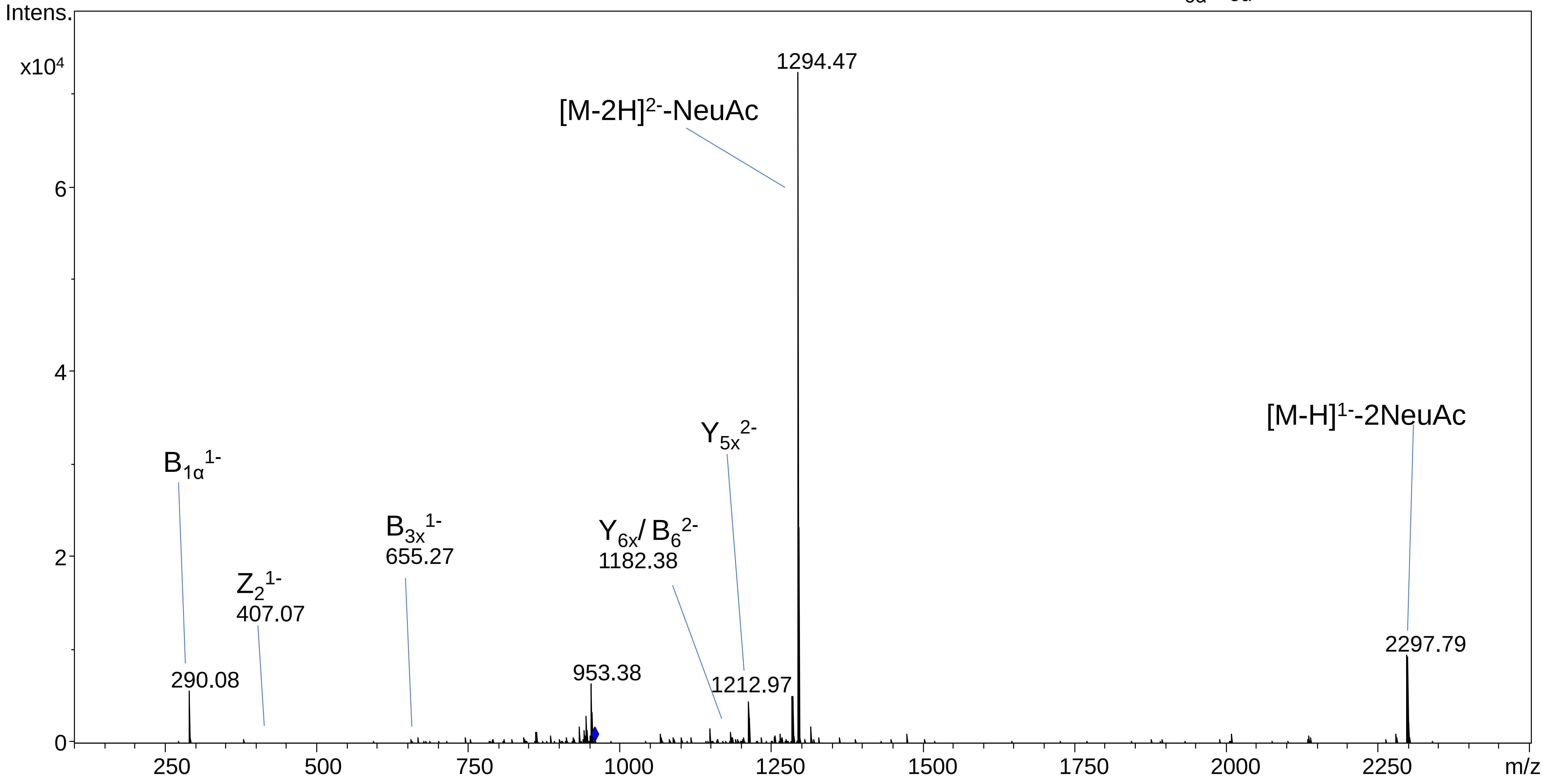

Glycan 27

H6N5F1S3

**Monoisotopic mass:**	**3027.08 Da**
**Charge observed:**	**3-**
**Theoretical ion:**	***m/z* 1008.02**
**Observed ion:**	***m/z* 1008.02**
**Mass deviation:**	***m/z* 0.00**
**Retention time:**	**60.5 min**

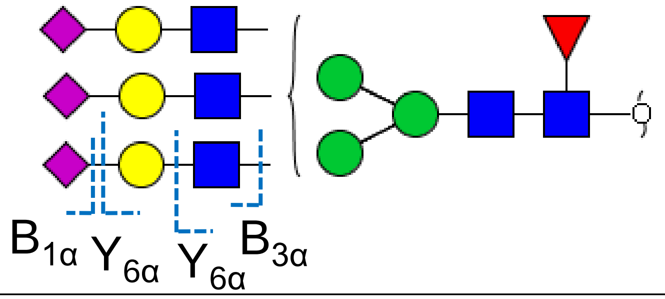

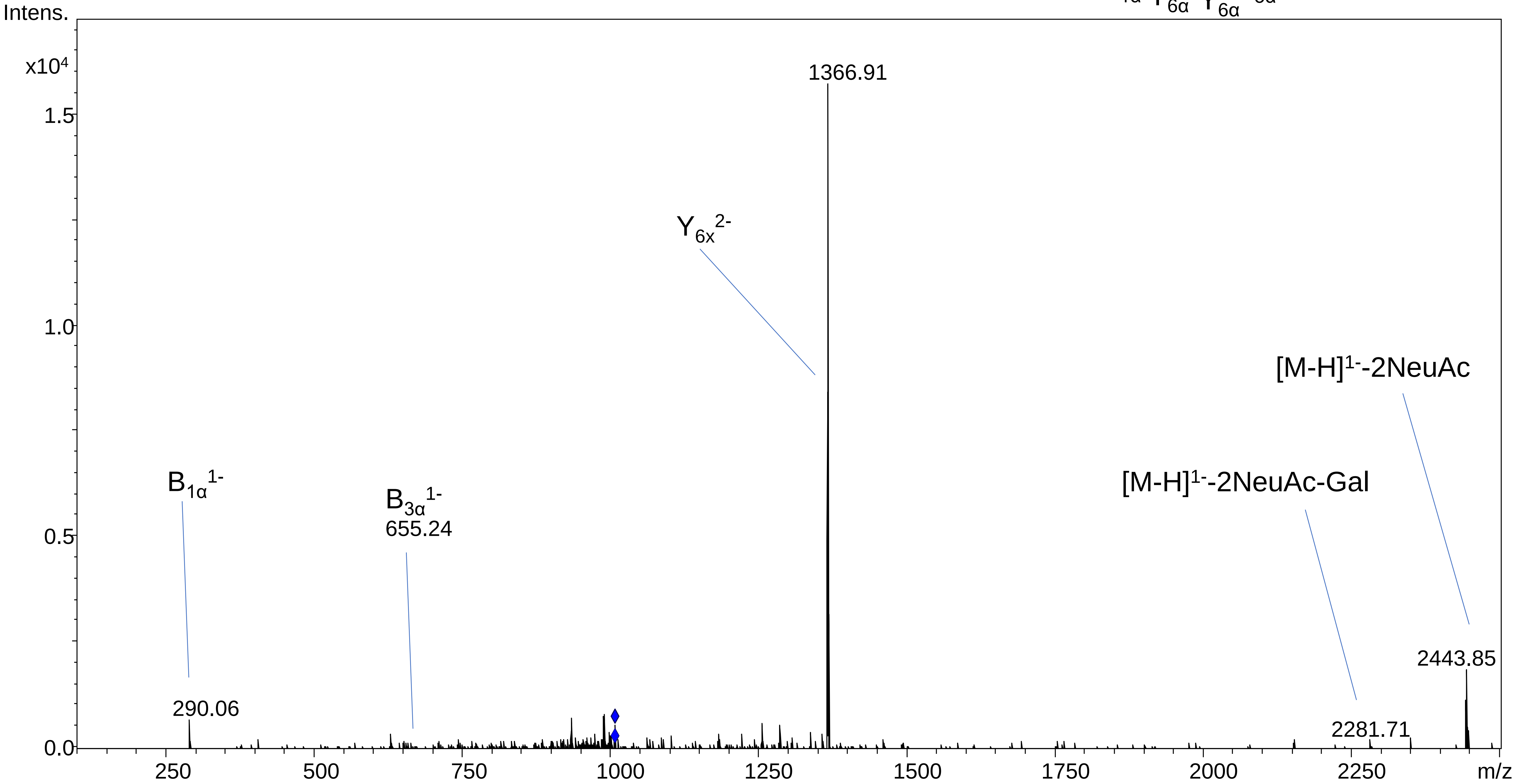

Supplement: Supplemental Figure S10 [file mmc2.pdf]
