# Supplementary material for: Transforming growth factor-β challenge alters the N-, O-, and glycosphingolipid glycomes in PaTu-S pancreatic adenocarcinoma cells
Source: J Biol Chem. 2022 Feb 11;298(3):101717. doi: 10.1016/j.jbc.2022.101717 (PMC8914387; doi:10.1016/j.jbc.2022.101717)

Supplementary Figure S11
for

Transforming growth factor-β challenge alters the *N*-, *O*-, and glycosphingolipid glycomes in PaTu-S pancreatic adenocarcinoma cells

Jing Zhang[1], Zejian Zhang[2,3], Stephanie Holst[2], Constantin Blöchl[2,4], Katarina Madunic[2], Manfred Wuhrer[2], Peter ten Dijke[1*] and Tao Zhang[2*]

[1]Oncode Institute and Dept. of Cell Chemical Biology, Leiden University Medical Center, 2300 RC Leiden, The Netherlands.
[2]Center for Proteomics and Metabolomics, Leiden University Medical Center, Leiden, The Netherlands.
[3]Current address: Department of Medical Research Center, Peking Union Medical College Hospital, Chinese Academy of Medical Sciences and Peking Union Medical College, Beijing, China
[4]Department of Biosciences, University of Salzburg, Salzburg, Austria.

Supplementary Figure S11. Annotated MS/MS for *O*-glycans. *O*-glycans have been numbered according to Supplementary Table S2. Glycan schemes were derived from GlycoWorkbench. Annotation was based on the presence of structural feature ions and common knowledge of known glycan synthetic pathways.

Glycan 1

H1N1

**Monoisotopic mass:**	**385.16 Da**
**Charge observed:**	**1-**
**Theoretical ion:**	***m/z* 384.15**
**Observed ion:**	***m/z* 384.13**
**Mass deviation:**	***m/z* 0.03**
**Retention time:**	**18.4 min**

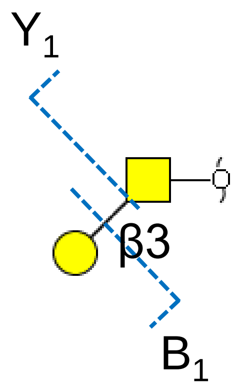

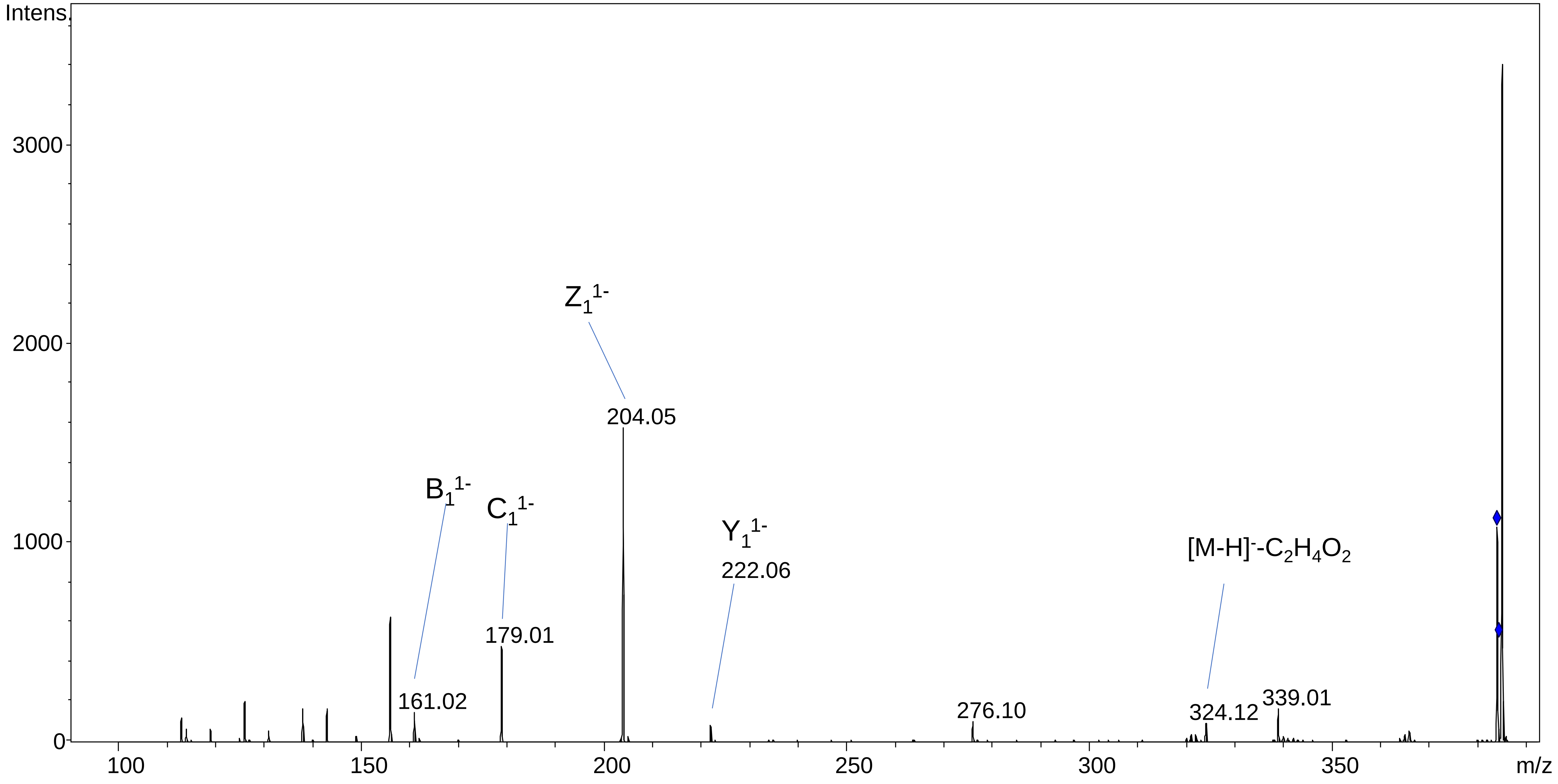

Glycan 2

N1S1

**Monoisotopic mass:**	**514.20 Da**
**Charge observed:**	**1-**
**Theoretical ion:**	***m/z* 513.19**
**Observed ion:**	***m/z* 513.19**
**Mass deviation:**	***m/z* 0.00**
**Retention time:**	**26.1 min**

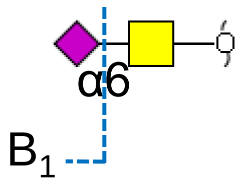

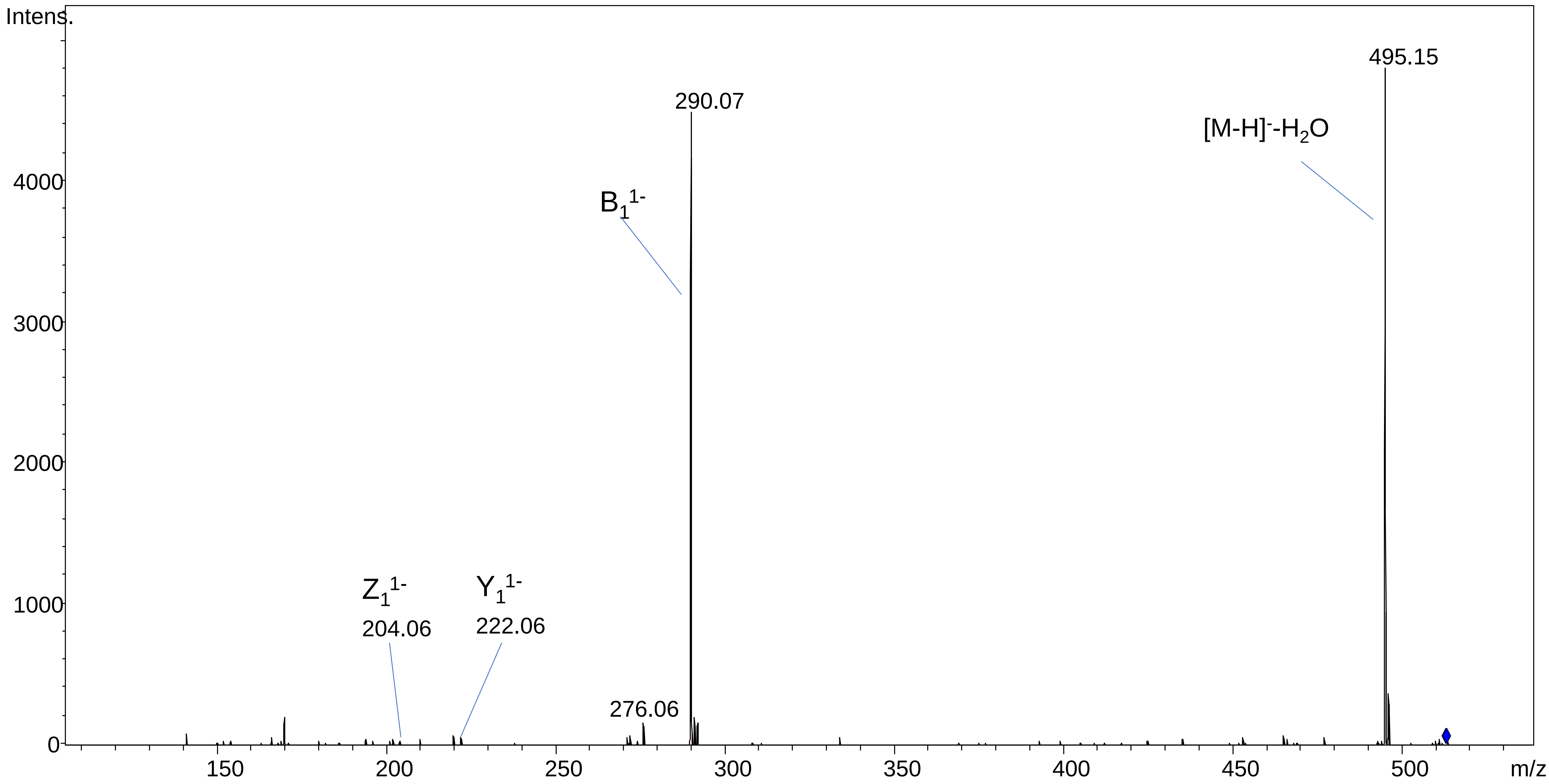

Glycan 3

H1N2

Monoisotopic mass: **588.24 Da**
Charge observed: **1-**
Theoretical ion: ***m/z* 587.23**
Observed ion: ***m/z* 587.23**
Mass deviation: ***m/z* 0.00**
Retention time: **38.0 min**

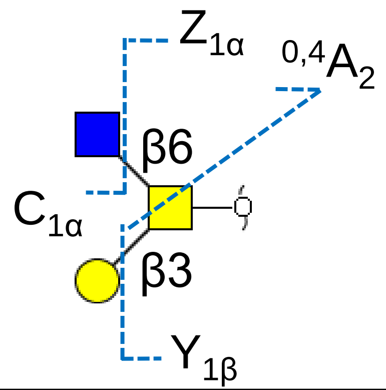

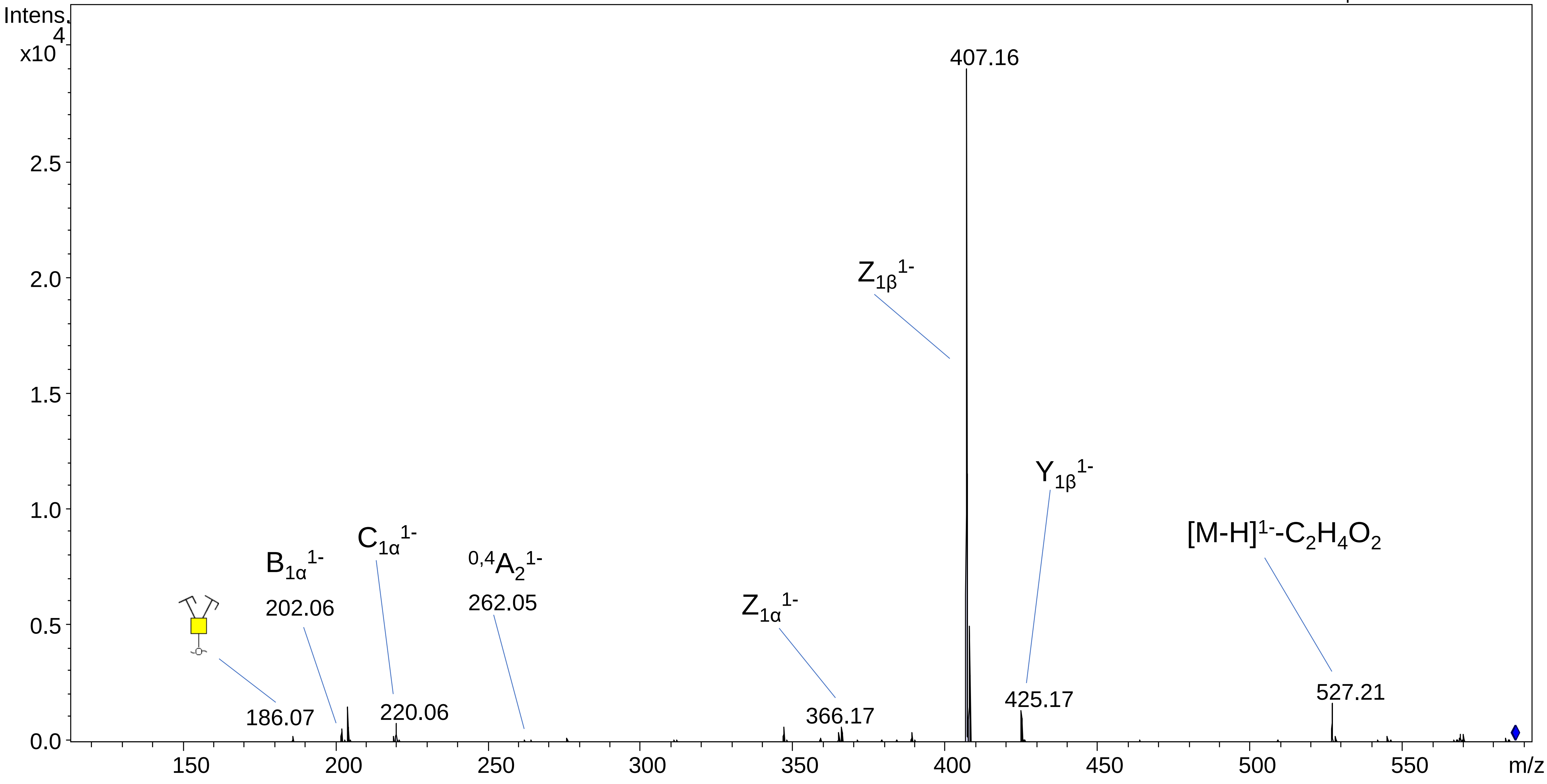

Glycan 4

H1N1S1

Monoisotopic mass: **676.26 Da**
Charge observed: **1-**
Theoretical ion: ***m/z* 675.25**
Observed ion: ***m/z* 675.24**
Mass deviation: ***m/z* 0.01**
Retention time: **33.5 min**

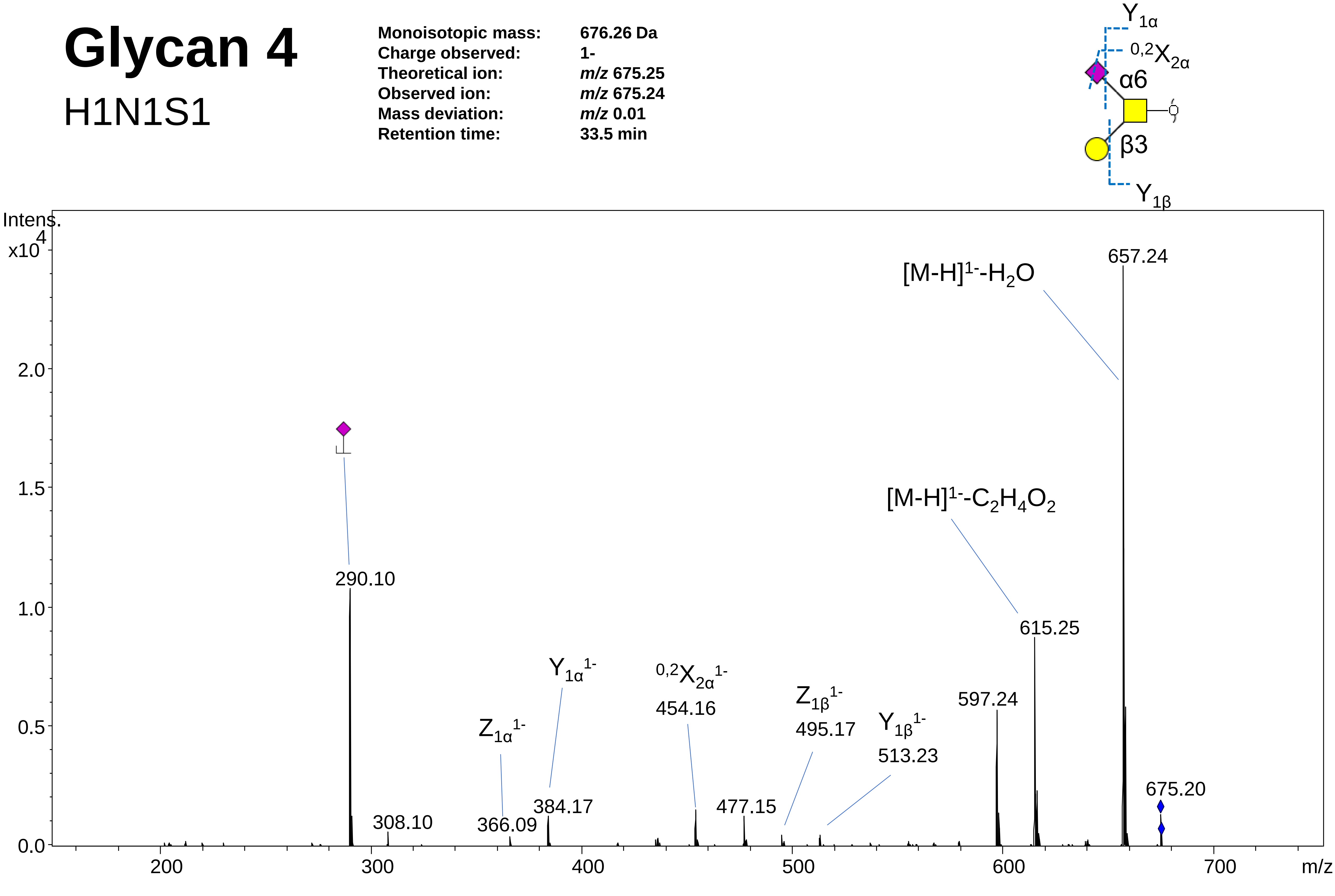

Glycan 5

H1N1S1

**Monoisotopic mass:**	**676.26 Da**
**Charge observed:**	**1-**
**Theoretical ion:**	***m/z* 675.25**
**Observed ion:**	***m/z* 675.25**
**Mass deviation:**	***m/z* 0.00**
**Retention time:**	**40.6 min**

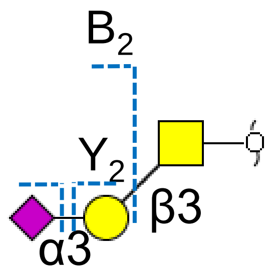

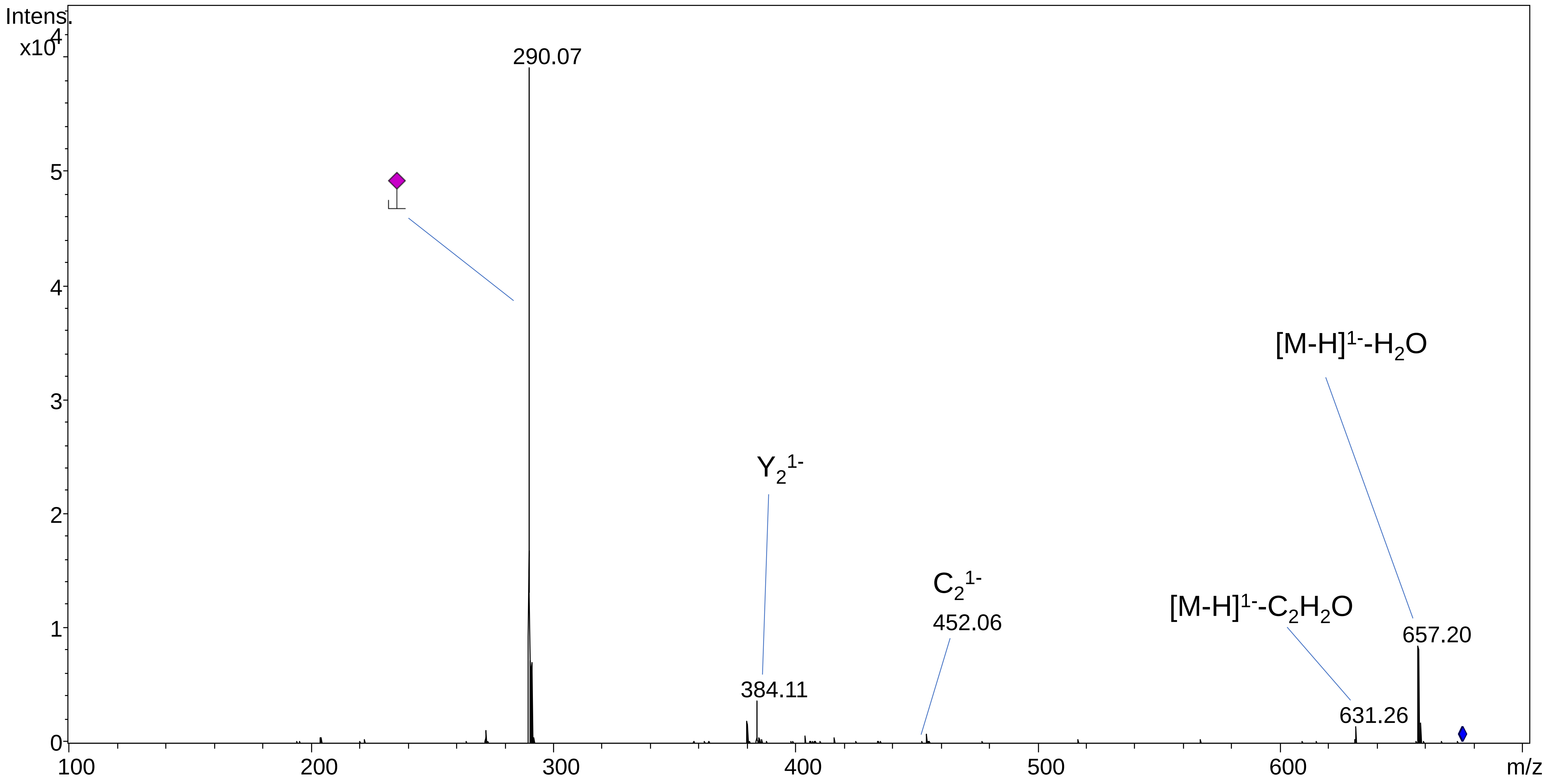

Glycan 6

H2N2

**Monoisotopic mass:**	**750.29 Da**
**Charge observed:**	**1-**
**Theoretical ion:**	***m/z* 749.28**
**Observed ion:**	***m/z* 749.28**
**Mass deviation:**	***m/z* 0.00**
**Retention time:**	**49.2 min**

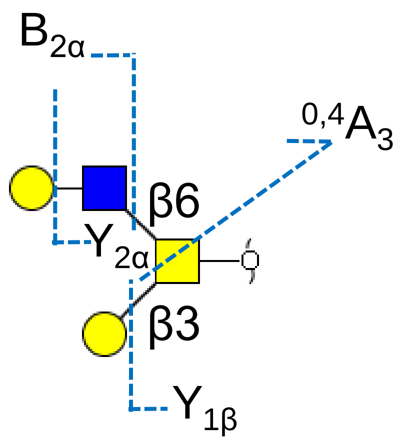

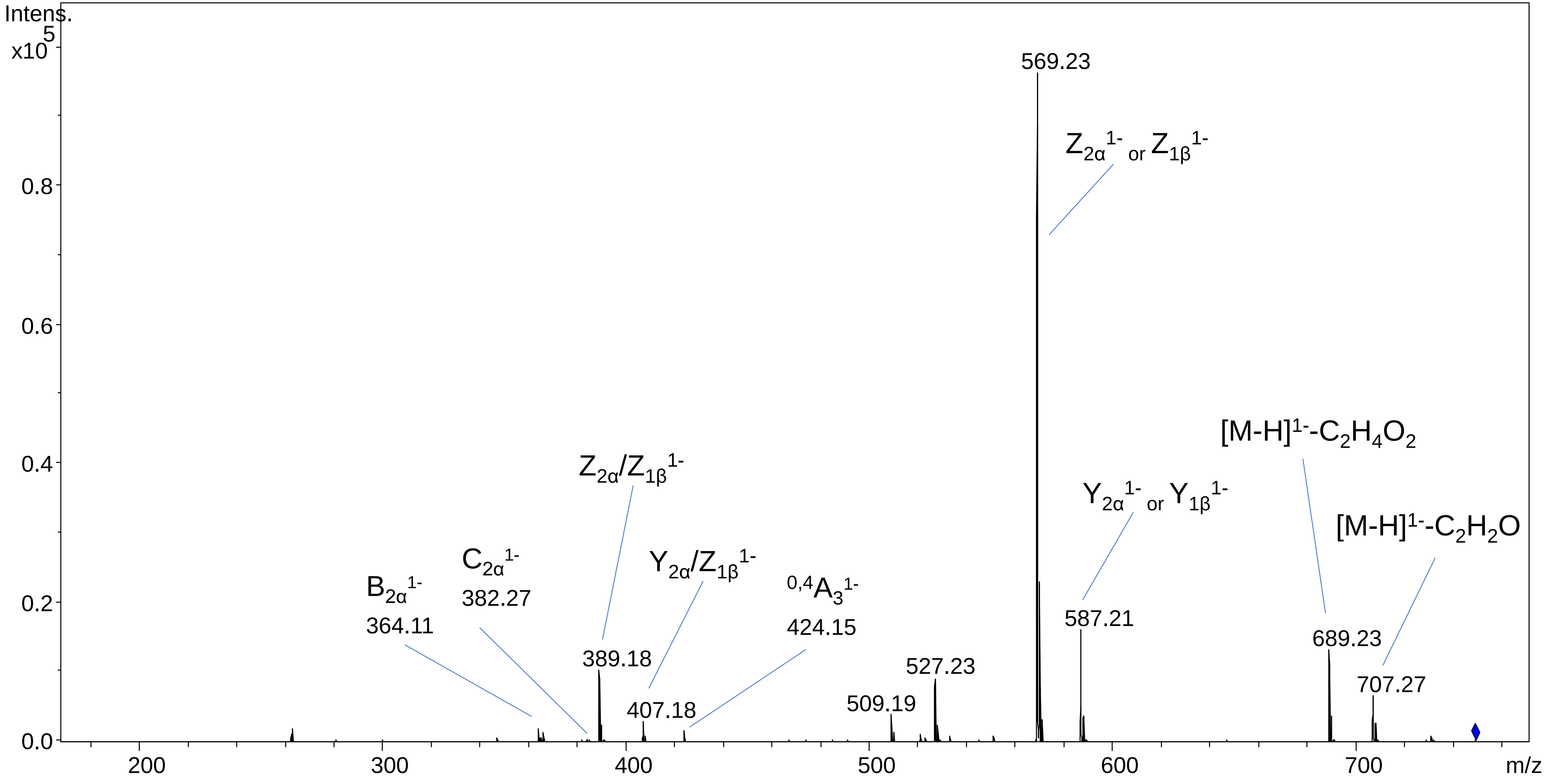

Glycan 7

H1N2S1

**Monoisotopic mass:**	**879.34 Da**
**Charge observed:**	**1-**
**Theoretical ion:**	***m/z* 878.33**
**Observed ion:**	***m/z* 878.33**
**Mass deviation:**	***m/z* 0.00**
**Retention time:**	**49.8 min**

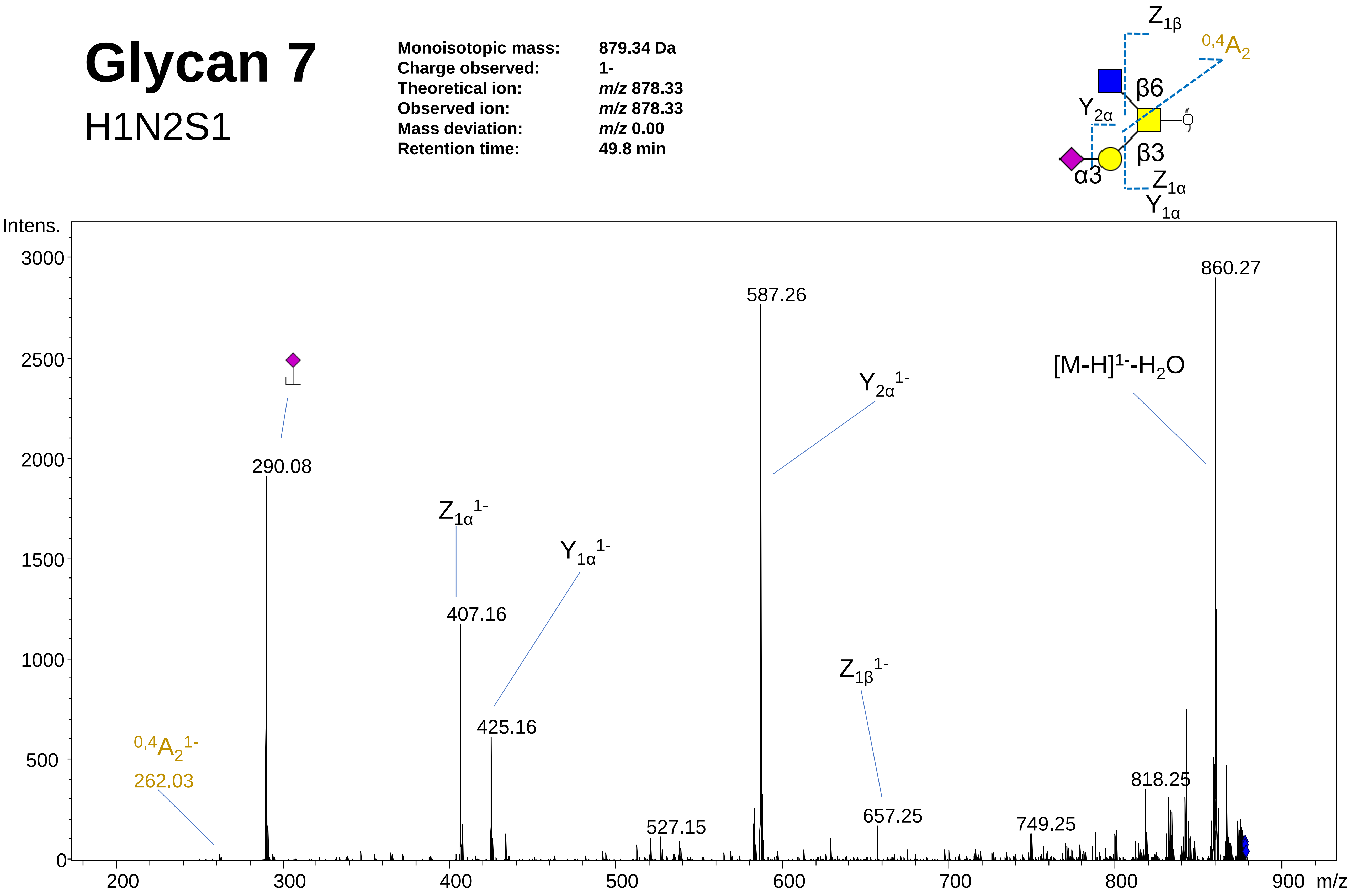

Glycan 8

H1N1S2

**Monoisotopic mass:**	**967.35 Da**
**Charge observed:**	**1-**
**Theoretical ion:**	***m/z* 966.34 *m/z***
**Observed ion:**	***m/z* 966.34 *m/z***
**Mass deviation:**	***m/z* 0.00 *m/z***
**Retention time:**	**41.7 min**

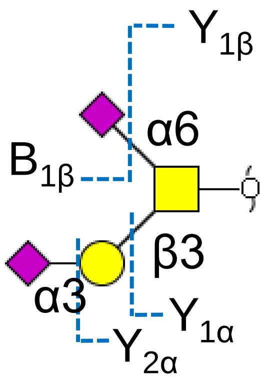

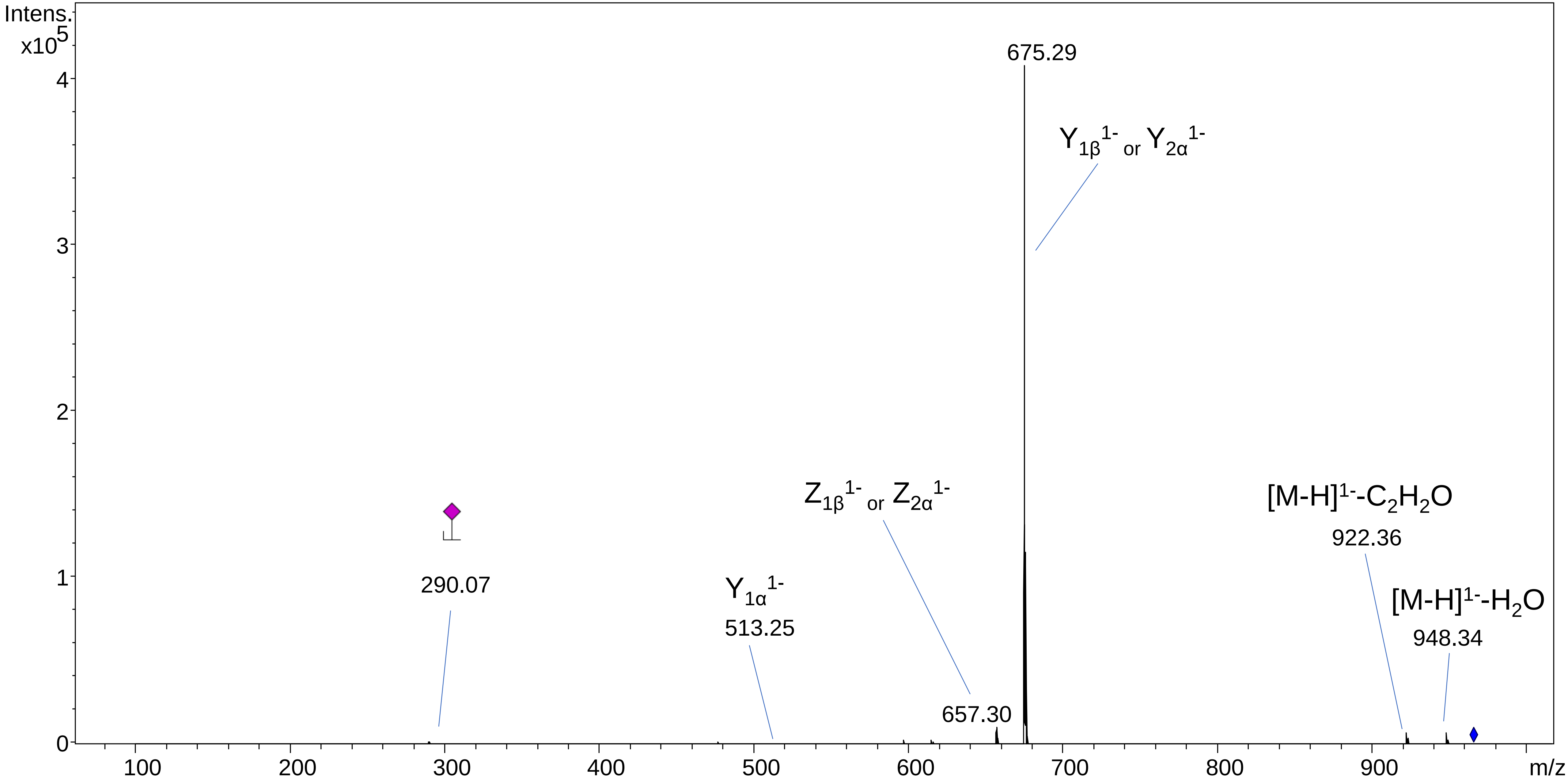

Glycan 9

H1N4

**Monoisotopic mass:**	**994.40 Da**
**Charge observed:**	**1-**
**Theoretical ion:**	***m/z* 993.39**
**Observed ion:**	***m/z* 993.41**
**Mass deviation:**	***m/z* 0.02**
**Retention time:**	**50.2 min**

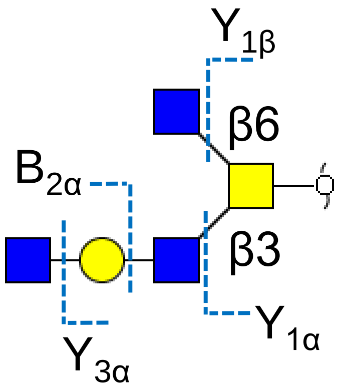

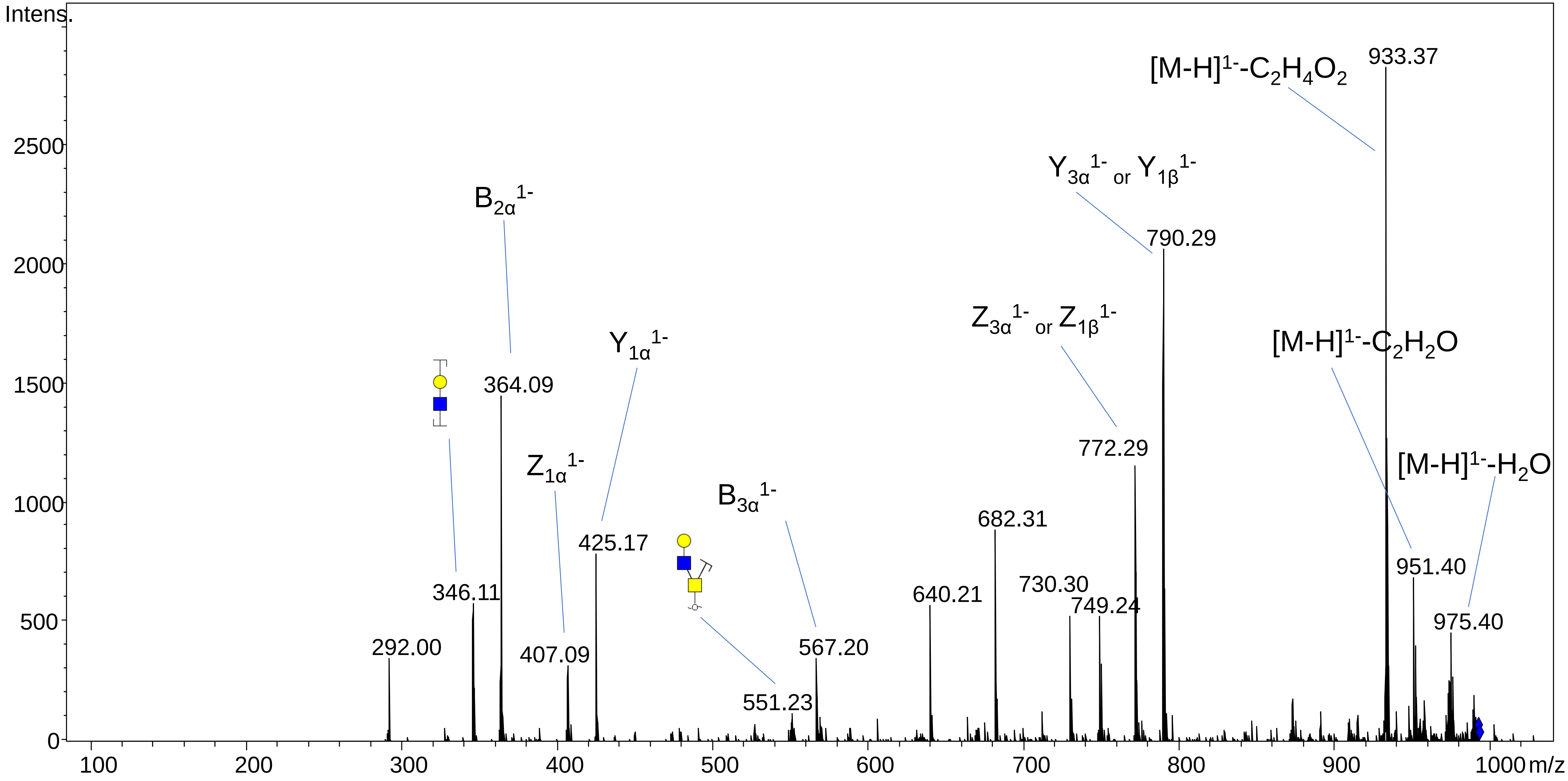

Glycan 10

H2N2S1

**Monoisotopic mass:**	**1041.40 Da**
**Charge observed:**	**1-**
**Theoretical ion:**	***m/z* 1040.39**
**Observed ion:**	***m/z* 1040.34**
**Mass deviation:**	***m/z* 0.05**
**Retention time:**	**51.4 min**

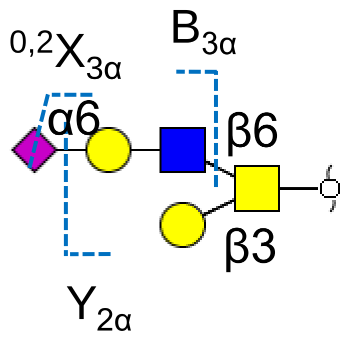

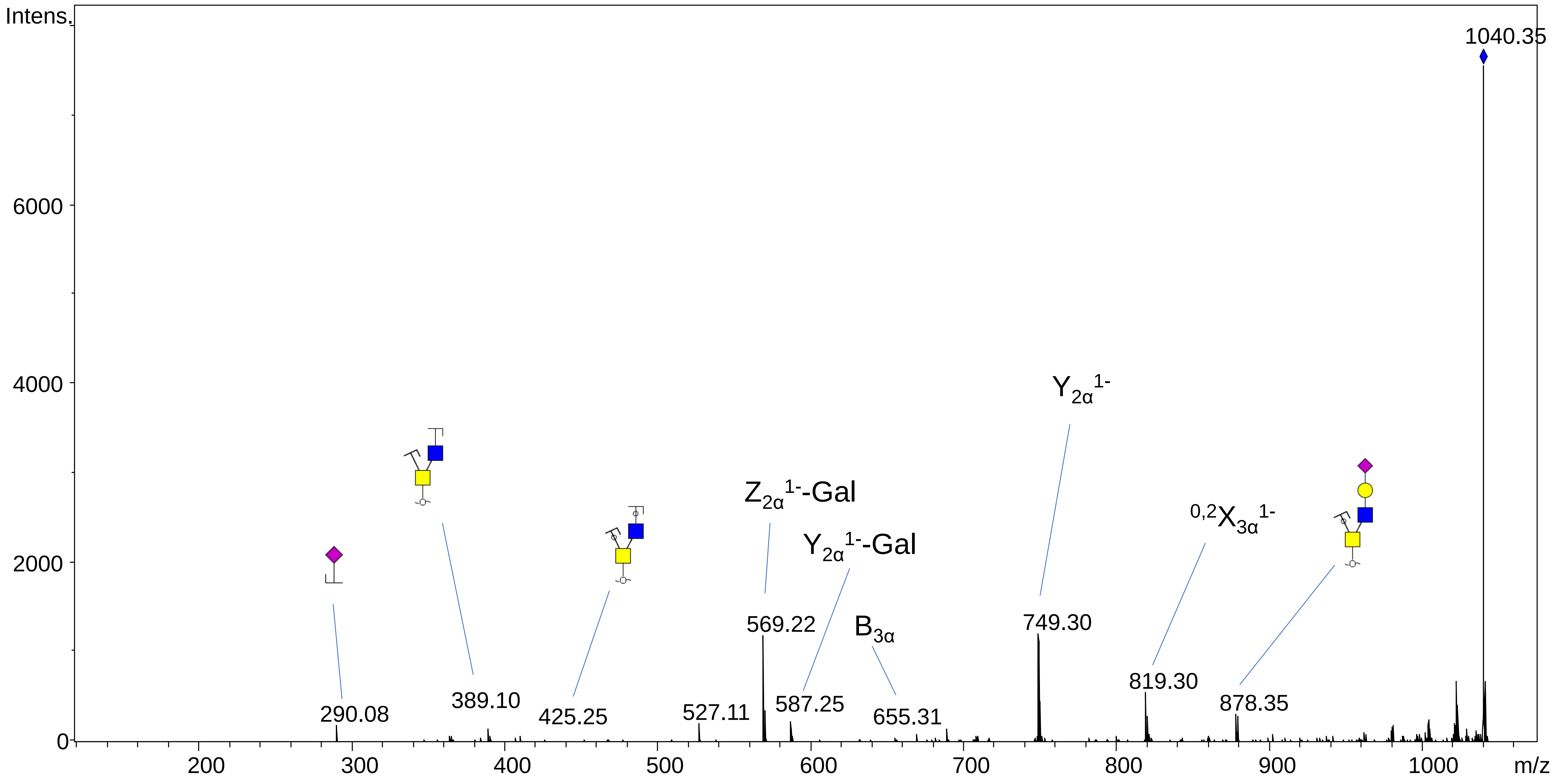

Glycan 11

H2N2S1

Monoisotopic mass: **1041.40 Da**
Charge observed: **1-**
Theoretical ion: ***m/z* 1040.39**
Observed ion: ***m/z* 1040.39**
Mass deviation: ***m/z* 0.00**
Retention time: **53.6 min**

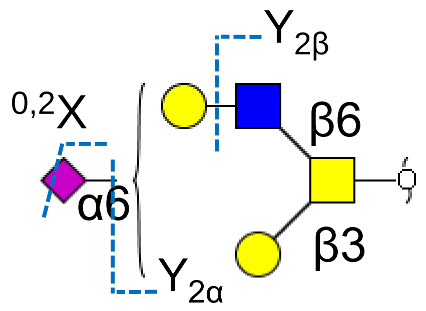

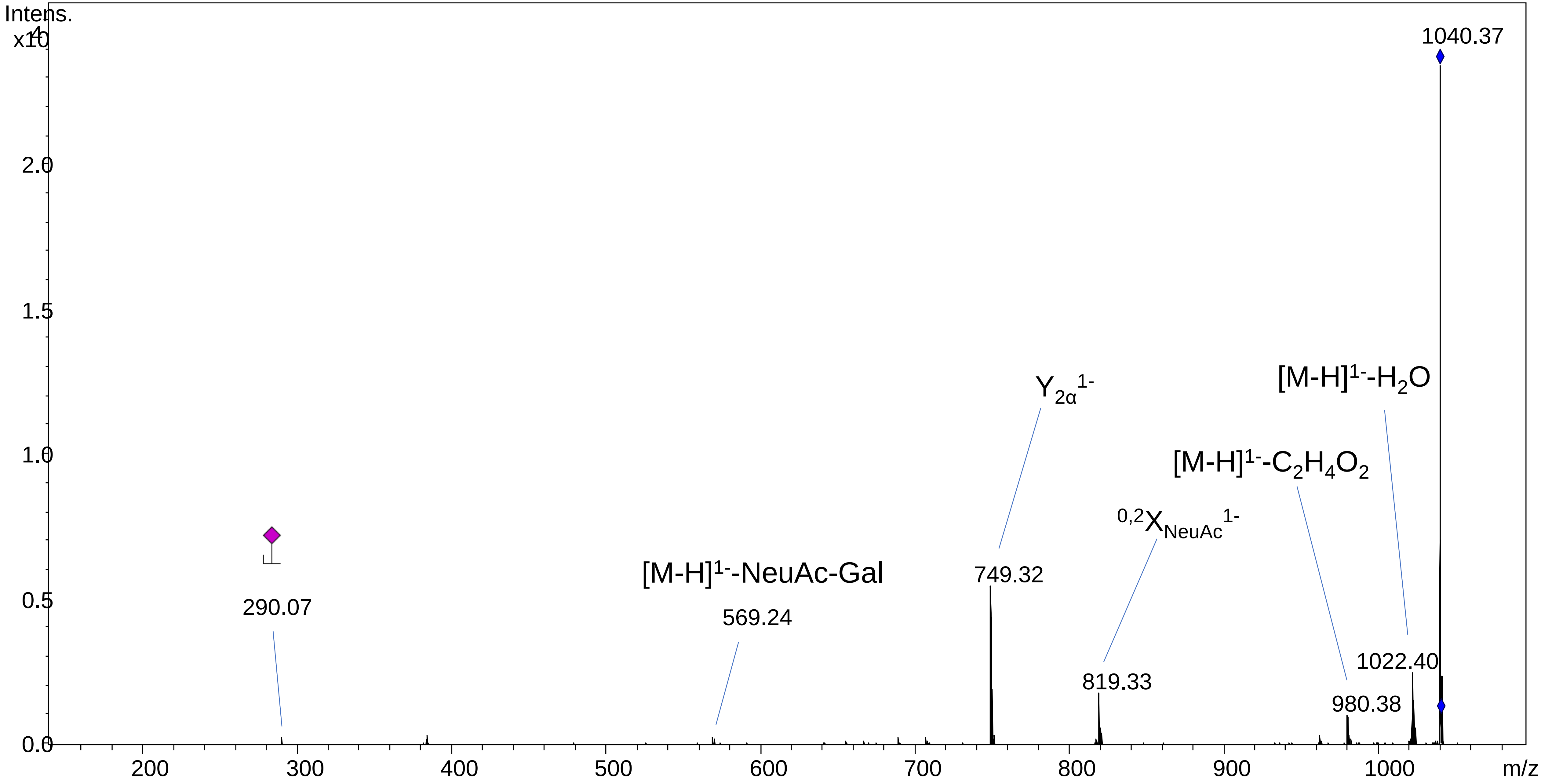

Glycan 12

H2N2S1

**Monoisotopic mass:**	**1041.40 Da**
**Charge observed:**	**1-**
**Theoretical ion:**	***m/z* 1040.39**
**Observed ion:**	***m/z* 1040.37**
**Mass deviation:**	***m/z* 0.02**
**Retention time:**	**57.9 min**

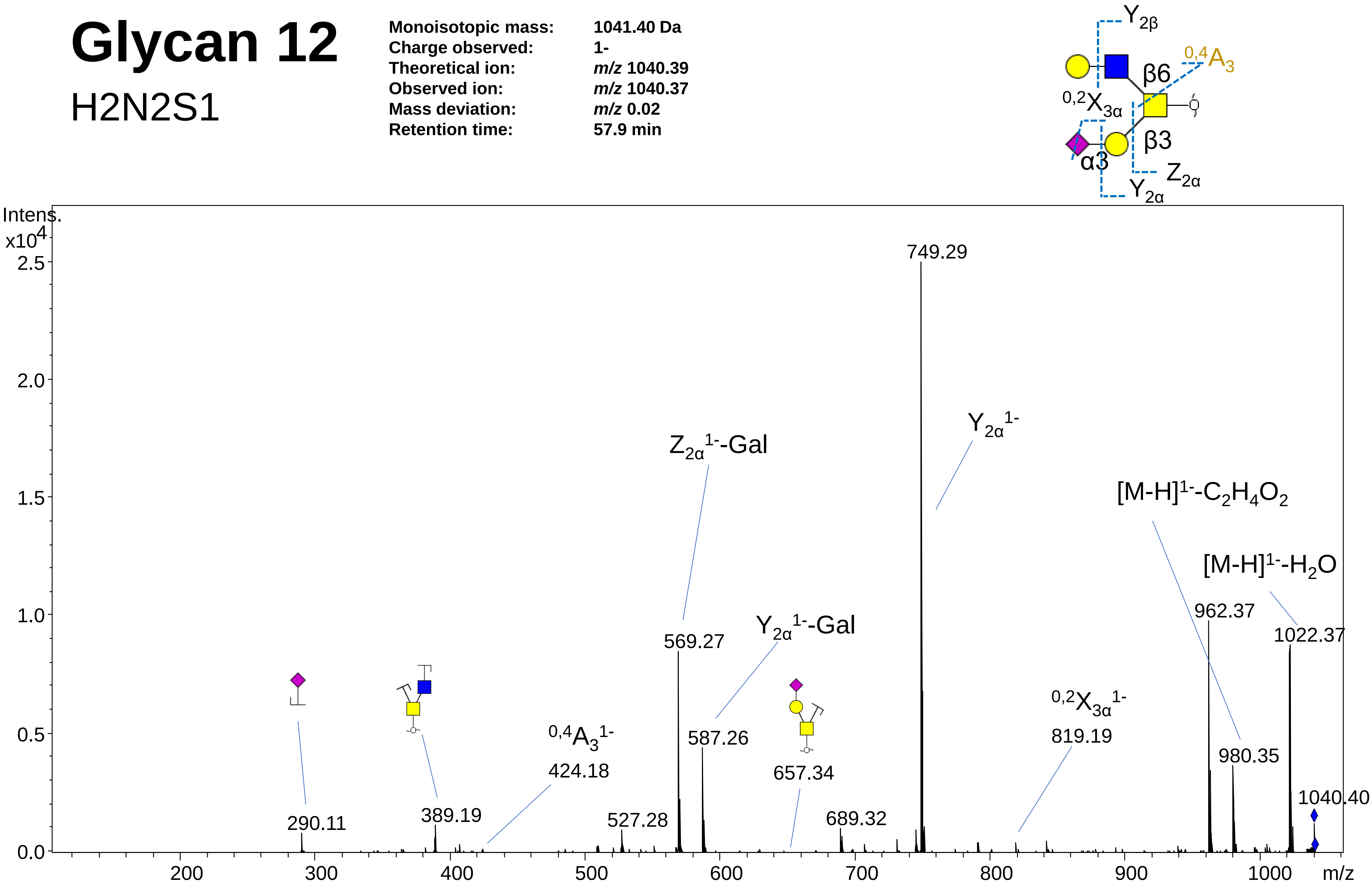

Glycan 13

H2N2S1

Monoisotopic mass: 1041.40 Da
Charge observed: 1-
Theoretical ion: *m/z* 1040.39
Observed ion: *m/z* 1040.37
Mass deviation: *m/z* 0.02
Retention time: 65.7 min

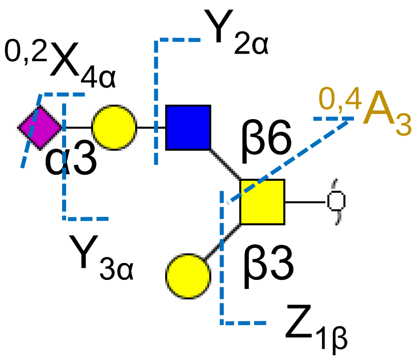

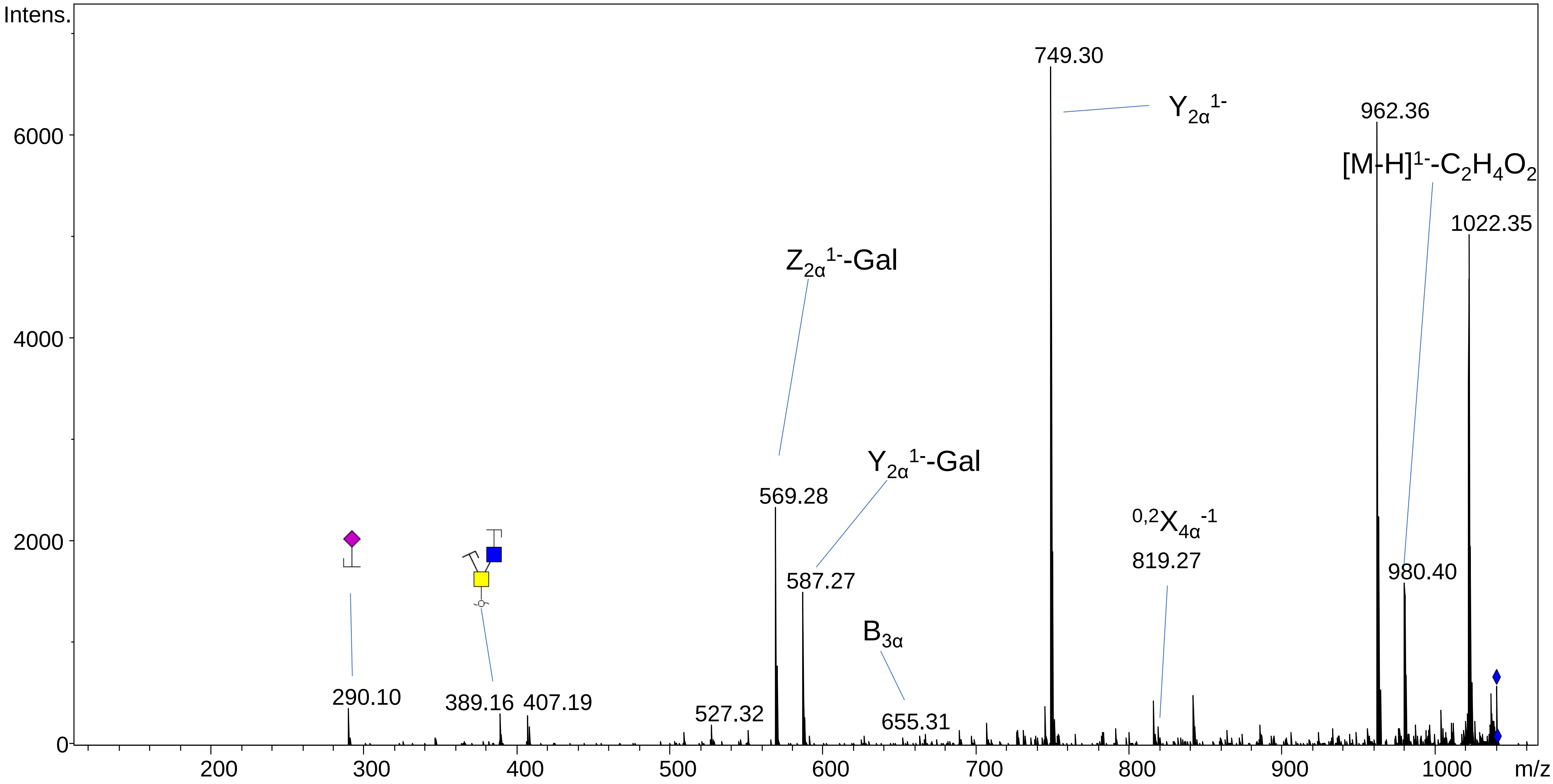

Glycan 14

H2N3F1

**Monoisotopic mass:**	**1099.43 Da**
**Charge observed:**	**1-**
**Theoretical ion:**	***m/z* 1098.42**
**Observed ion:**	***m/z* 1098.39**
**Mass deviation:**	***m/z* 0.03**
**Retention time:**	**47.7 min**

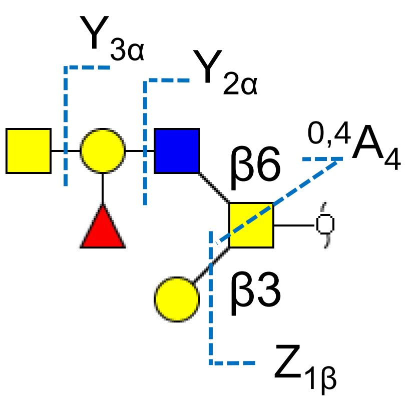

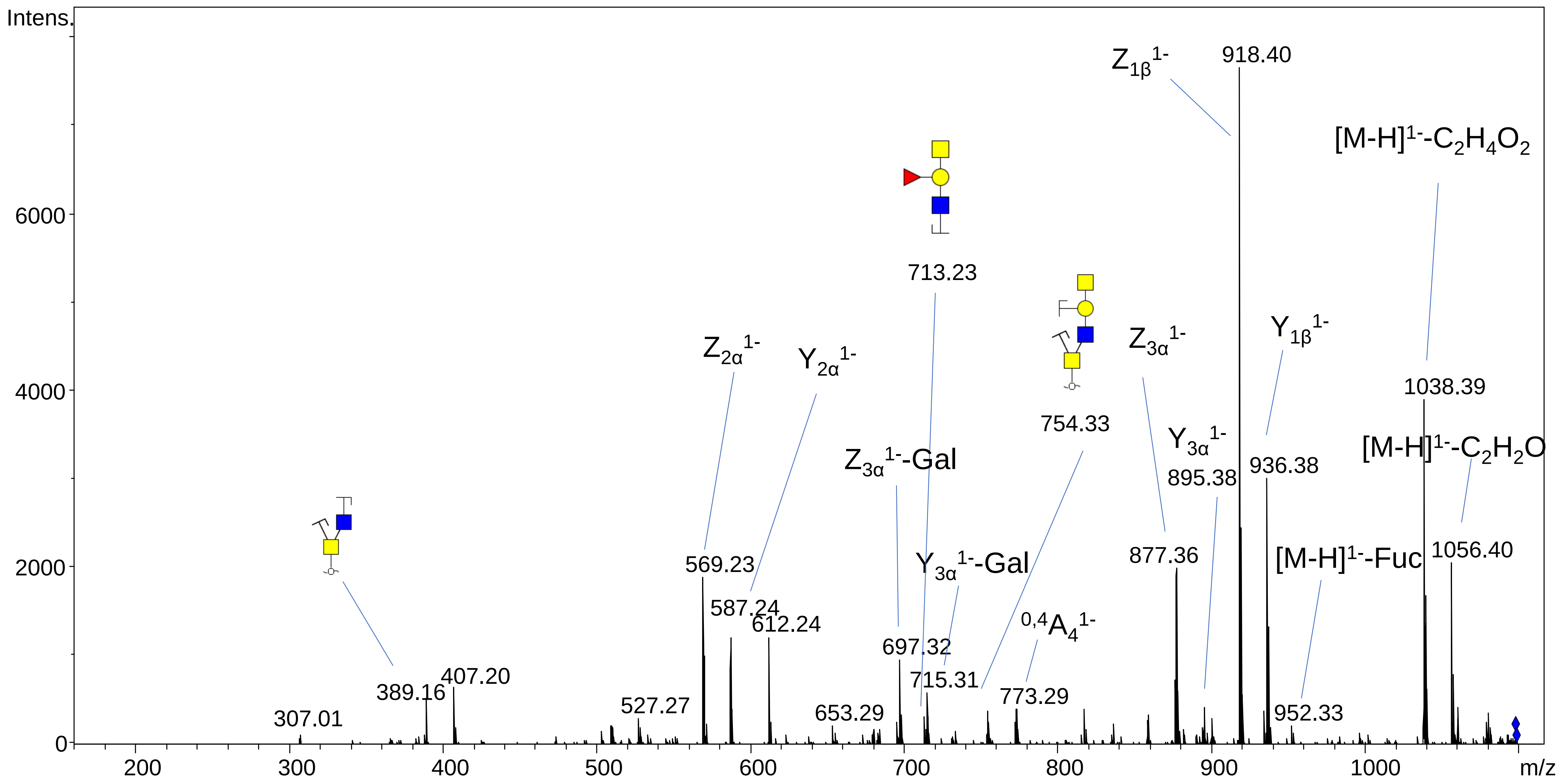

Glycan 15

H3N3

**Monoisotopic mass:**	**1115.43 Da**
**Charge observed:**	**1-**
**Theoretical ion:**	***m/z*** **1114.42**
**Observed ion:**	***m/z*** **1114.42**
**Mass deviation:**	***m/z*** **0.00**
**Retention time:**	**63.1 min**

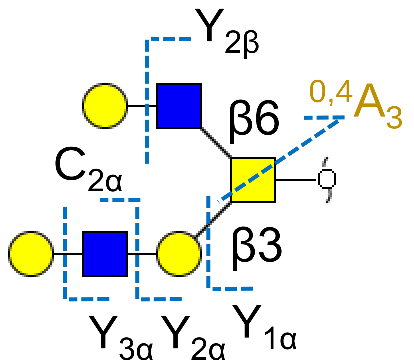

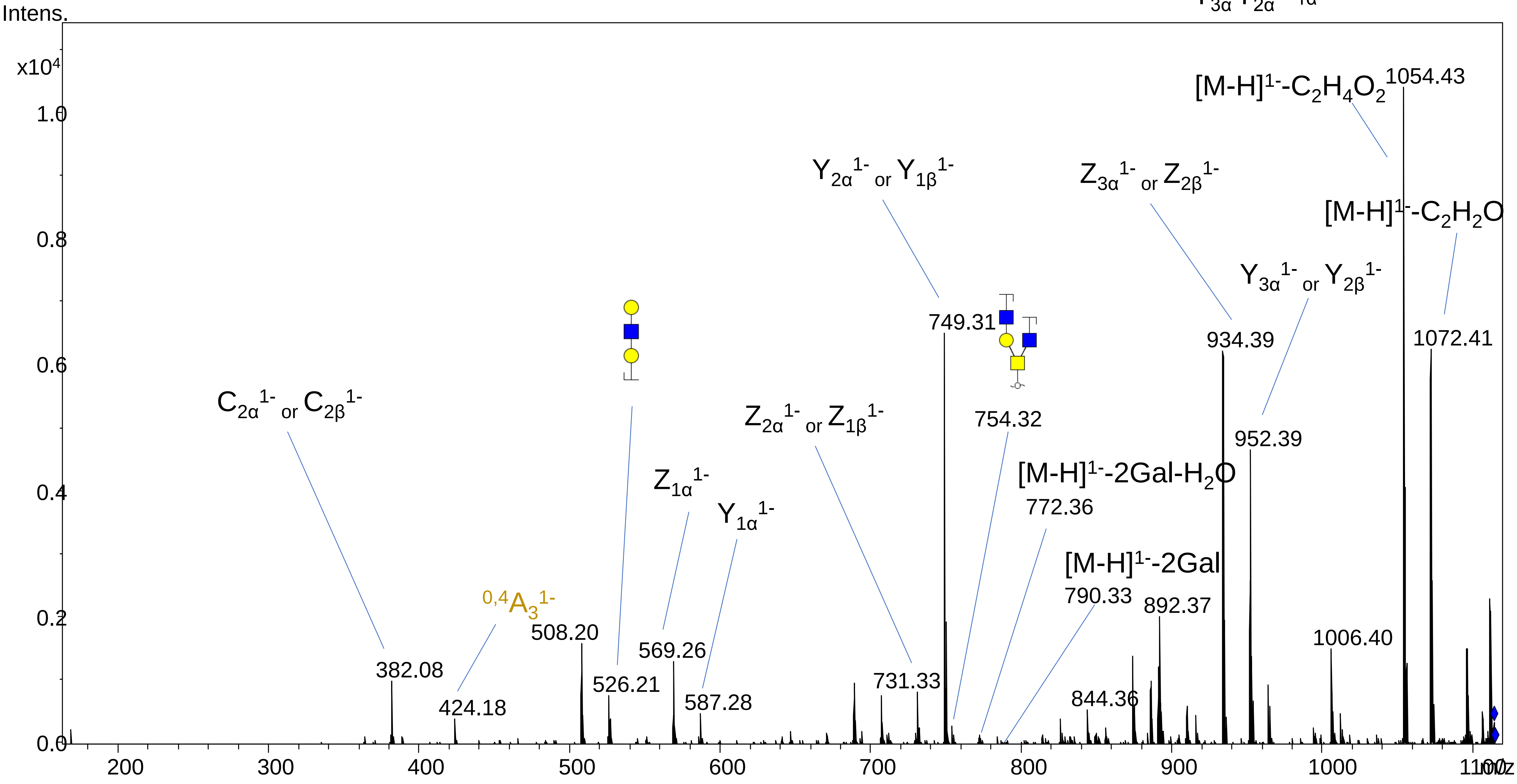

Glycan 16

H2N4

**Monoisotopic mass:**	**1156.45 Da**
**Charge observed:**	**1-**
**Theoretical ion:**	***m/z* 1155.44**
**Observed ion:**	***m/z* 1155.45**
**Mass deviation:**	***m/z* 0.01**
**Retention time:**	**56.5 min**

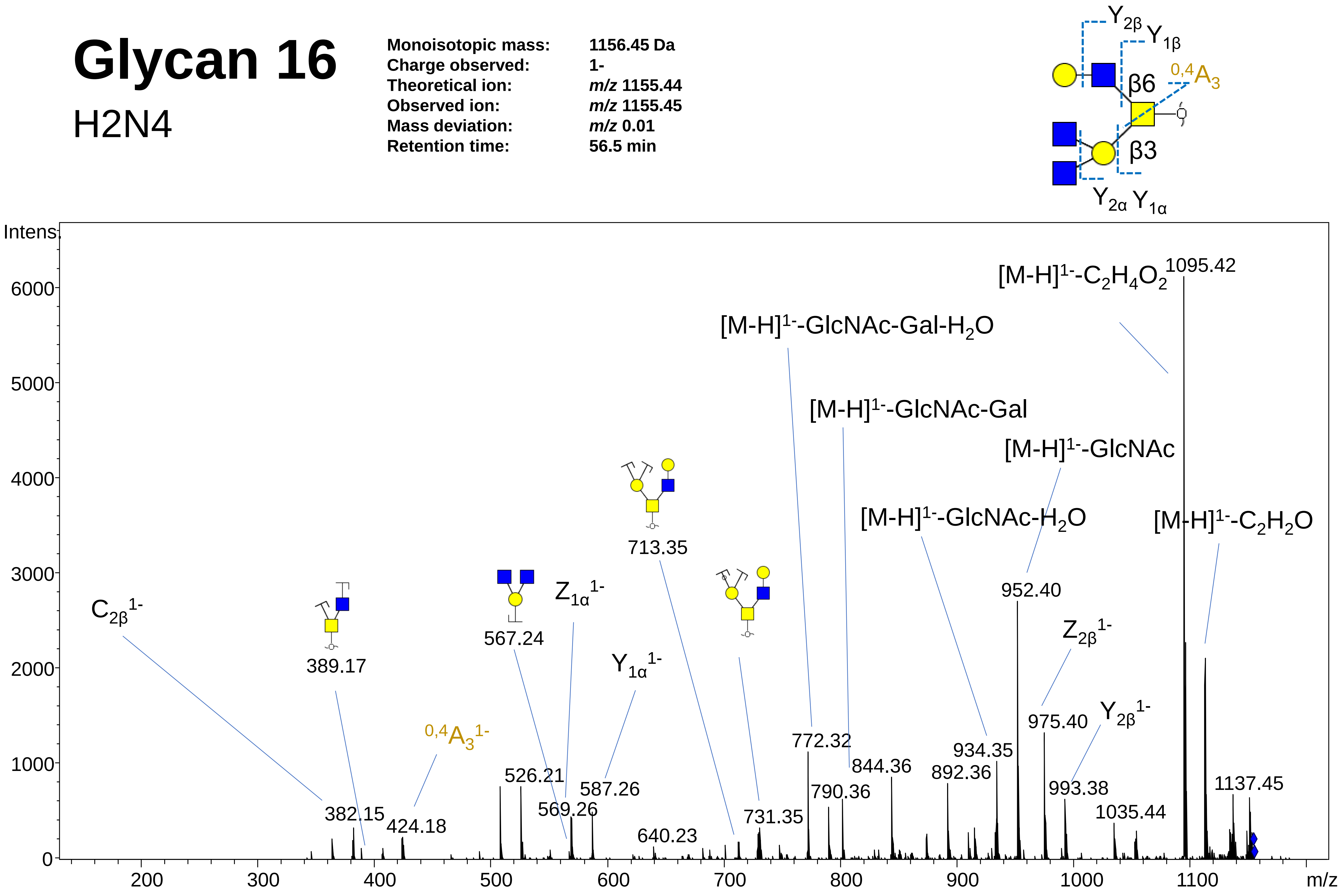

Glycan 17

H2N3F1S1

**Monoisotopic mass:**	**1187.44 Da**
**Charge observed:**	**1-**
**Theoretical ion:**	***m/z* 1186.44**
**Observed ion:**	***m/z* 1186.50**
**Mass deviation:**	***m/z* 0.06**
**Retention time:**	**47.7 min**

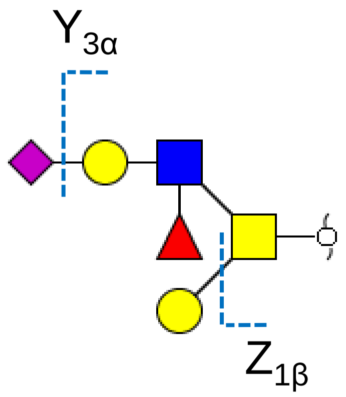

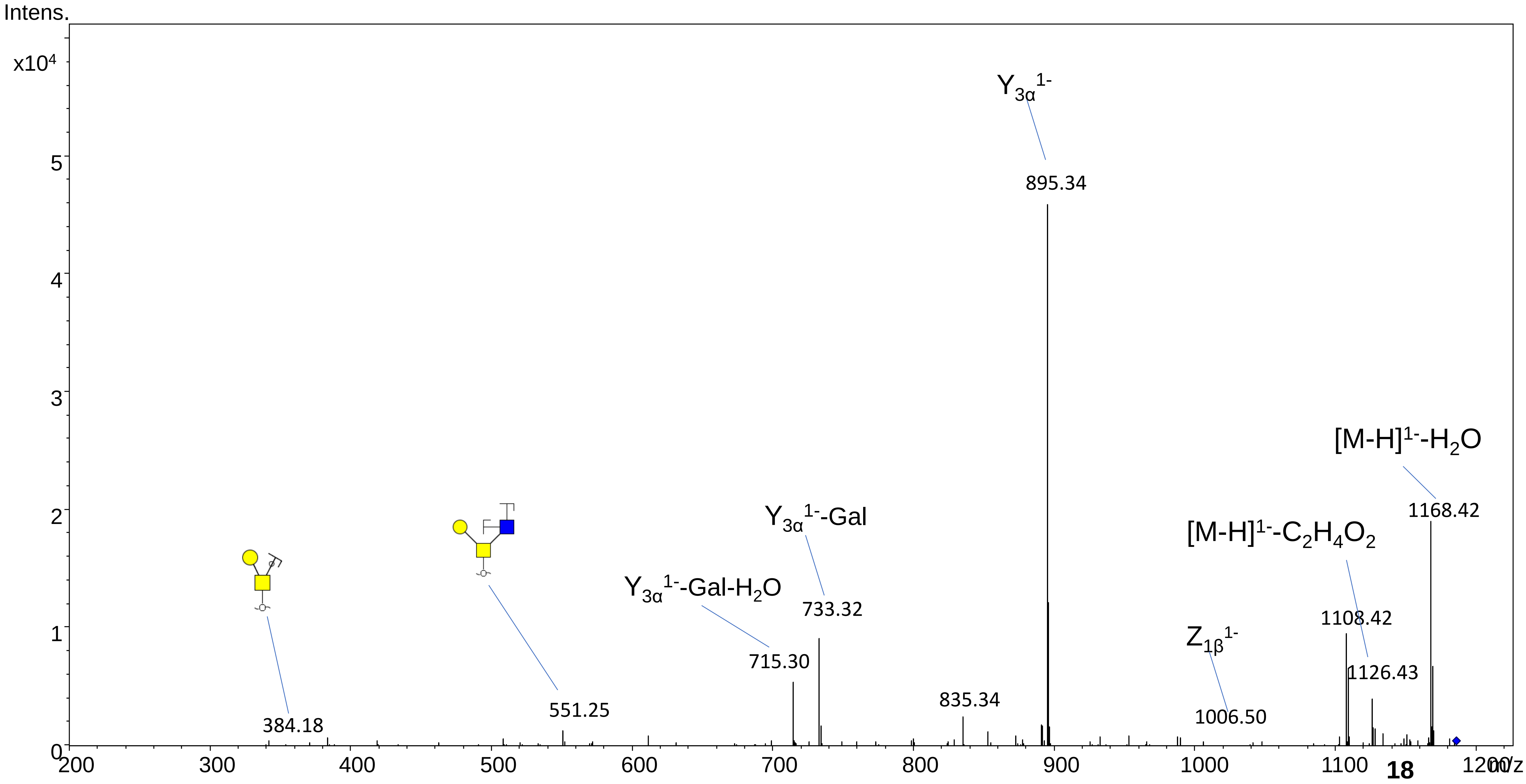

Glycan 18

H2N2S2

**Monoisotopic mass:**	**1332.48 Da**
**Charge observed:**	**2-**
**Theoretical ion:**	***m/z* 665.23**
**Observed ion:**	***m/z* 665.21**
**Mass deviation:**	***m/z* 0.02**
**Retention time:**	**55.1 min**

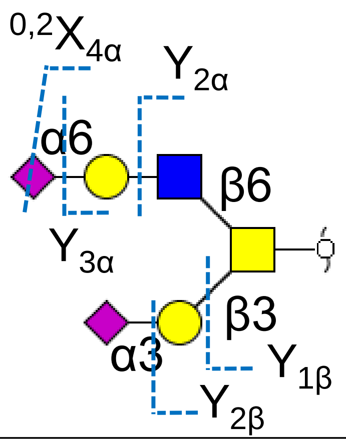

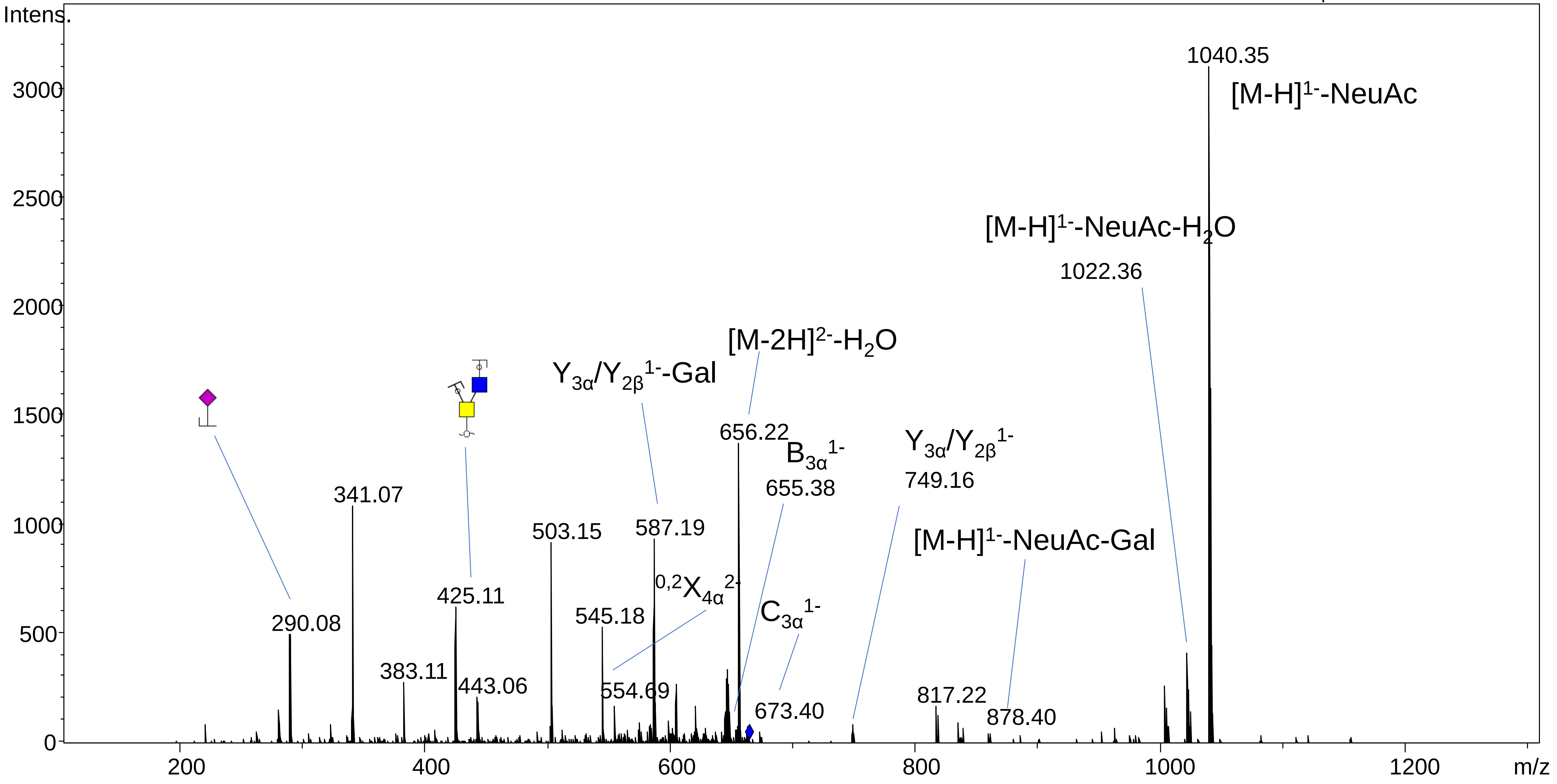

Glycan 19

H2N2S2

**Monoisotopic mass:**	**1332.48 Da**
**Charge observed:**	**2-**
**Theoretical ion:**	***m/z* 665.23**
**Observed ion:**	***m/z* 665.23**
**Mass deviation:**	***m/z* 0.00**
**Retention time:**	**69.0 min**

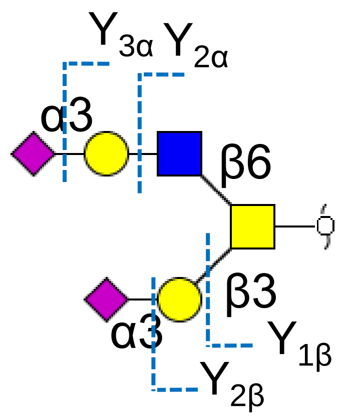

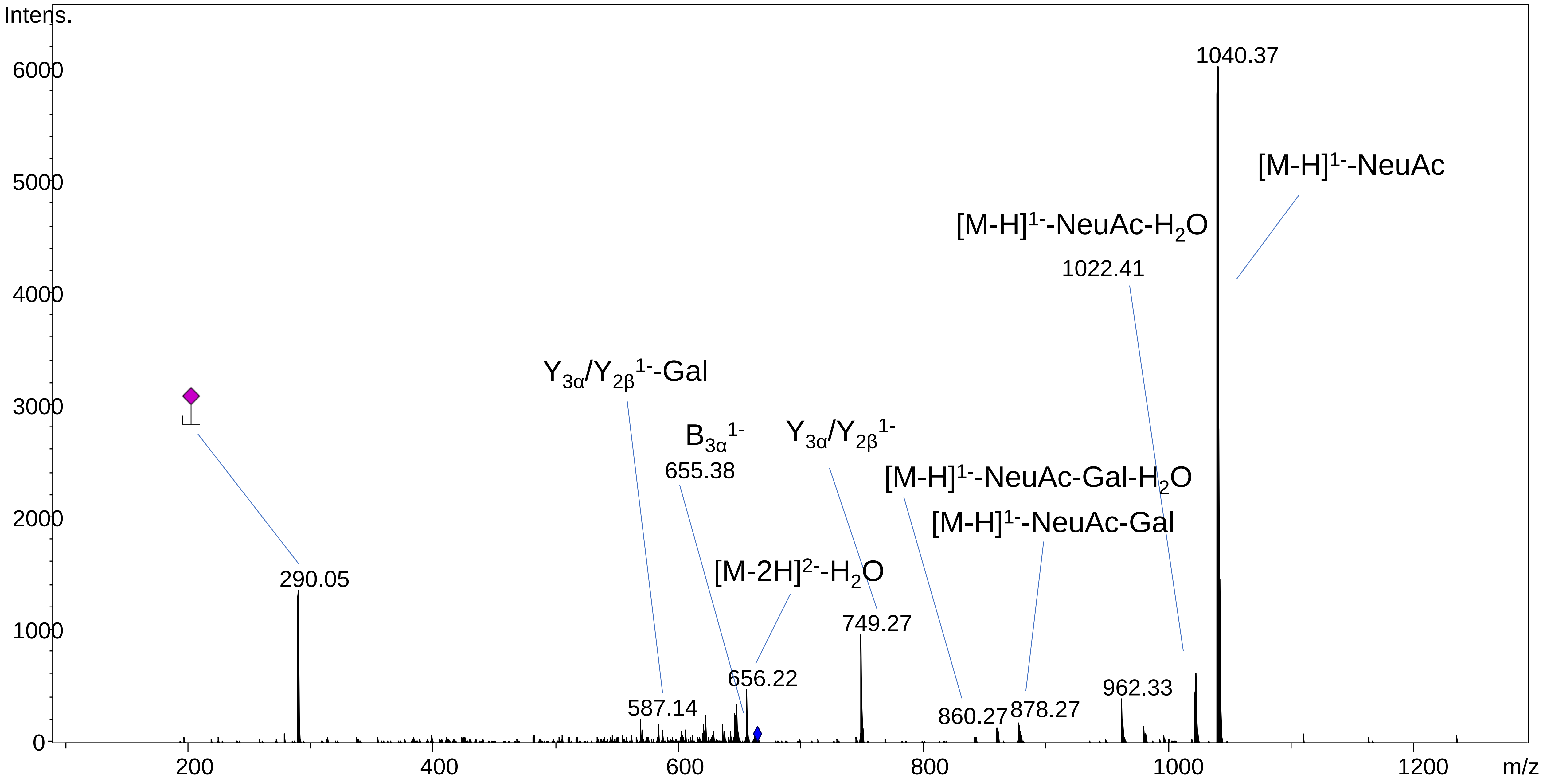

Glycan 20

H4N4

**Monoisotopic mass:**	**1480.56 Da**
**Charge observed:**	**2-**
**Theoretical ion:**	***m/z* 739.27**
**Observed ion:**	***m/z* 739.27**
**Mass deviation:**	***m/z* 0.00**
**Retention time:**	**67.3 min**

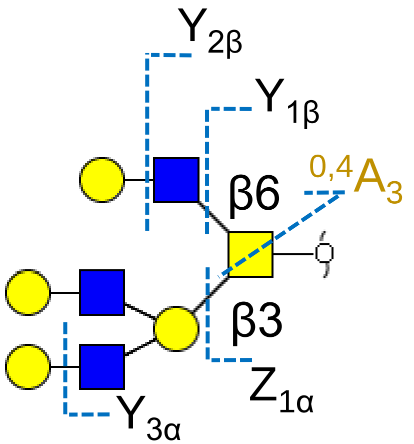

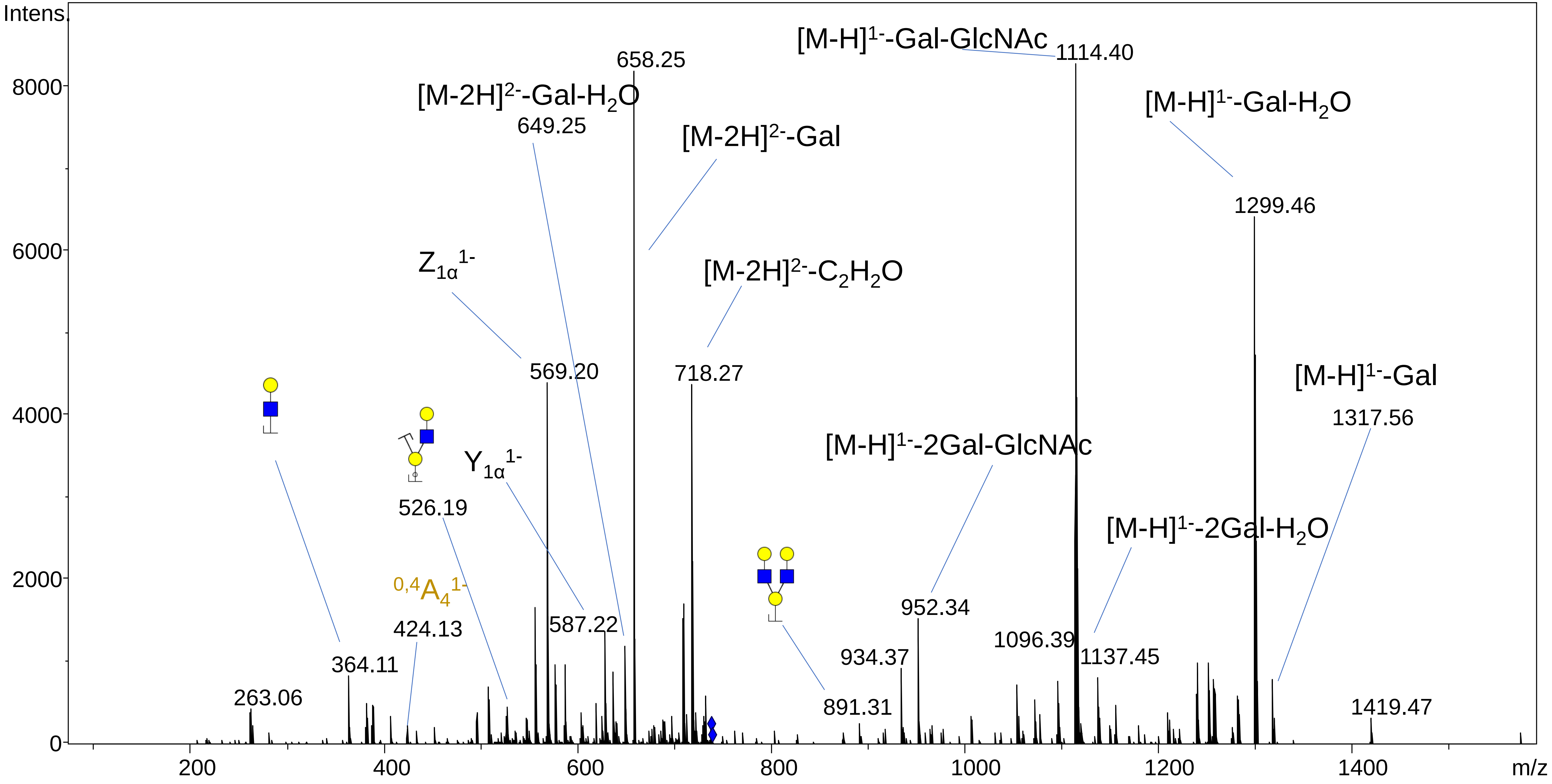

Supplement: Supplemental Figure S11 [file mmc3.pdf]
