# Supplementary material for: Transforming growth factor-β challenge alters the N-, O-, and glycosphingolipid glycomes in PaTu-S pancreatic adenocarcinoma cells
Source: J Biol Chem. 2022 Feb 11;298(3):101717. doi: 10.1016/j.jbc.2022.101717 (PMC8914387; doi:10.1016/j.jbc.2022.101717)

**Supplementary Figure S12**
**for**

# Transforming growth factor-β challenge alters the *N*-, *O*-, and glycosphingolipid glycomes in PaTu-S pancreatic adenocarcinoma cells

Jing Zhang[1], Zejian Zhang[2,3], Stephanie Holst[2], Constantin Blöchl[2,4], Katarina Madunic[2], Manfred Wuhrer[2], Peter ten Dijke[1*] and Tao Zhang[2*]

[1]Oncode Institute and Dept. of Cell Chemical Biology, Leiden University Medical Center, 2300 RC Leiden, The Netherlands.
[2]Center for Proteomics and Metabolomics, Leiden University Medical Center, Leiden, The Netherlands.
[3]Current address: Department of Medical Research Center, Peking Union Medical College Hospital, Chinese Academy of Medical Sciences and Peking Union Medical College, Beijing, China
[4]Department of Biosciences, University of Salzburg, Salzburg, Austria.

**Supplementary Figure S12**. Annotated MS/MS for GSL-glycans. GSL-glycans have been numbered according to Supplementary Table S3. Glycan schemes were derived from GlycoWorkbench. Annotation was based on the presence of structural feature ions and common knowledge of known glycan synthetic pathways.

# Glycan 1

Gb3

H3

**Monoisotopic mass:** **506.34 Da**
**Charge observed:** **1-**
**Theoretical ion:** ***m/z* 505.34**
**Observed ion:** ***m/z* 505.19**
**Mass deviation:** ***m/z* 0.15**
**Retention time:** **25.0 min**

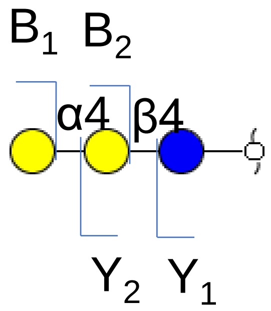


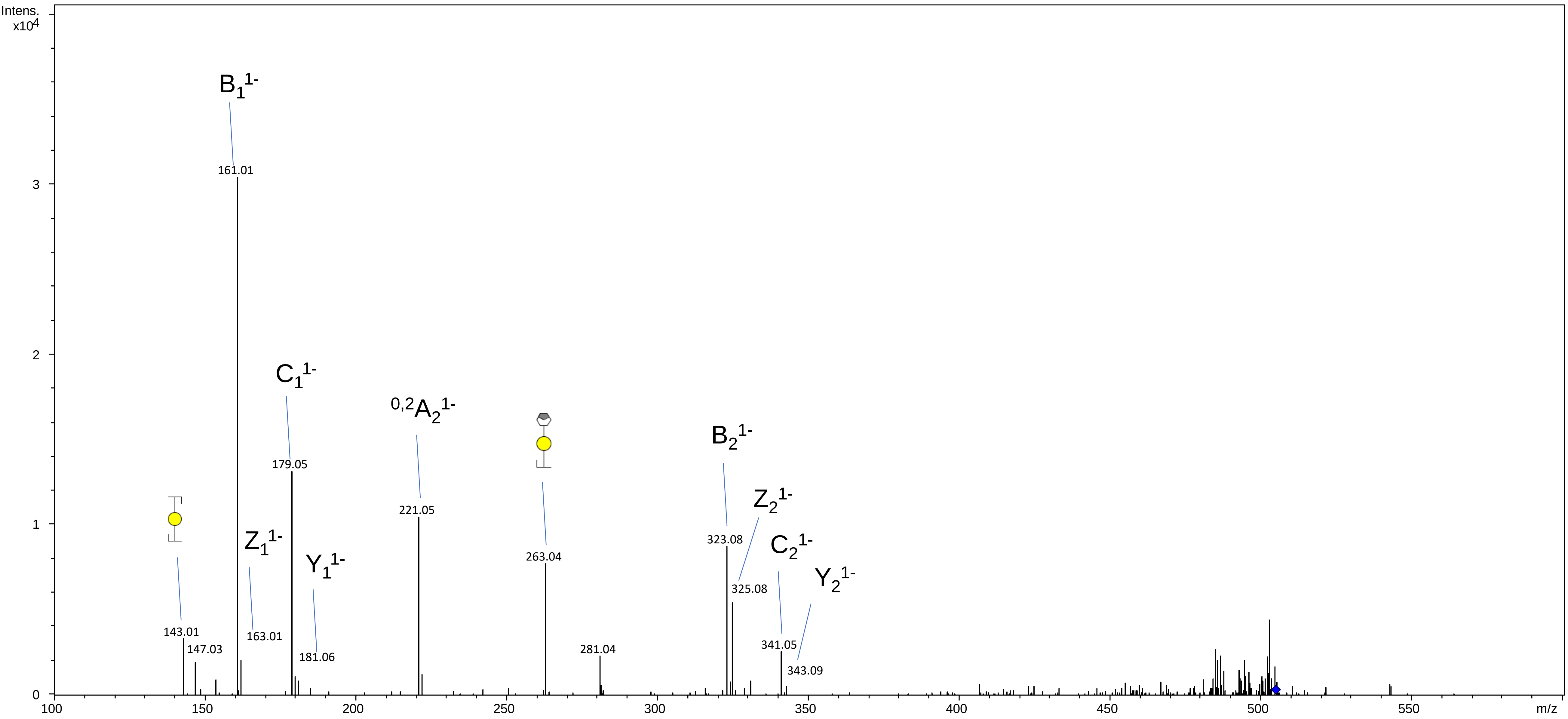

# Glycan 2

Gb4

H3N1

**Monoisotopic mass:** **709.28 Da**
**Charge observed:** **1-**
**Theoretical ion:** ***m/z* 708.26**
**Observed ion:** ***m/z* 708.28**
**Mass deviation:** **0.02 *m/z***
**Retention time:** **22.3 min**

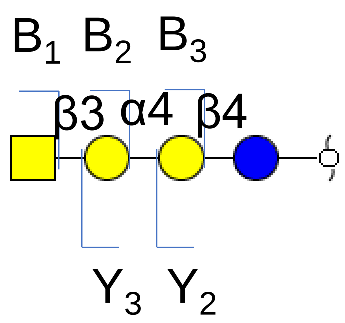


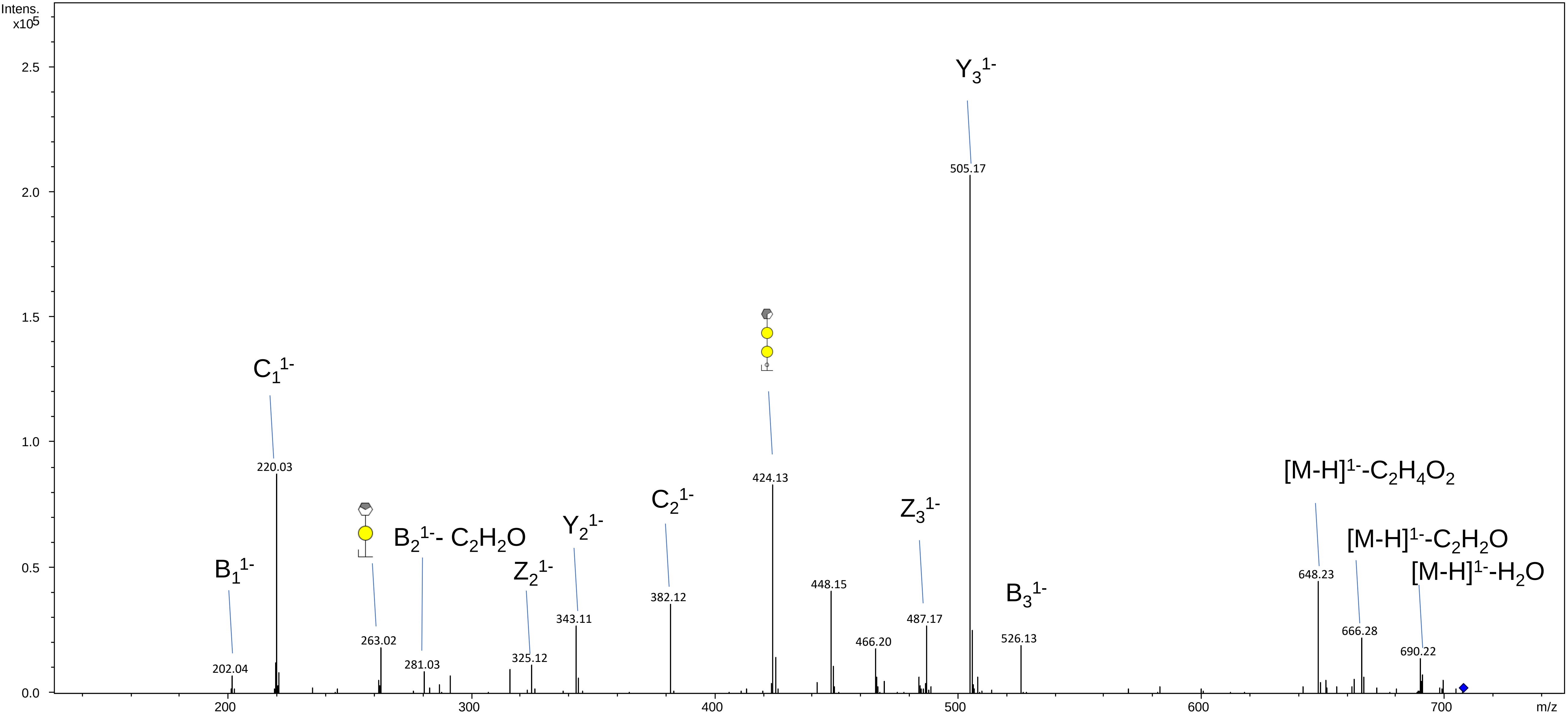

# Glycan 3

GM3
H2S1

| | |
|---|---|
| **Monoisotopic mass:** | **635.22 Da** |
| **Charge observed:** | **1-** |
| **Theoretical ion:** | ***m/z* 634.22** |
| **Observed ion:** | ***m/z* 634.27** |
| **Mass deviation:** | ***m/z* 0.05** |
| **Retention time:** | **40.3 min** |

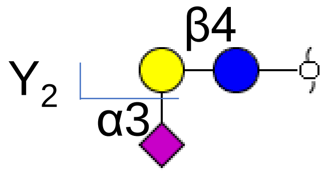


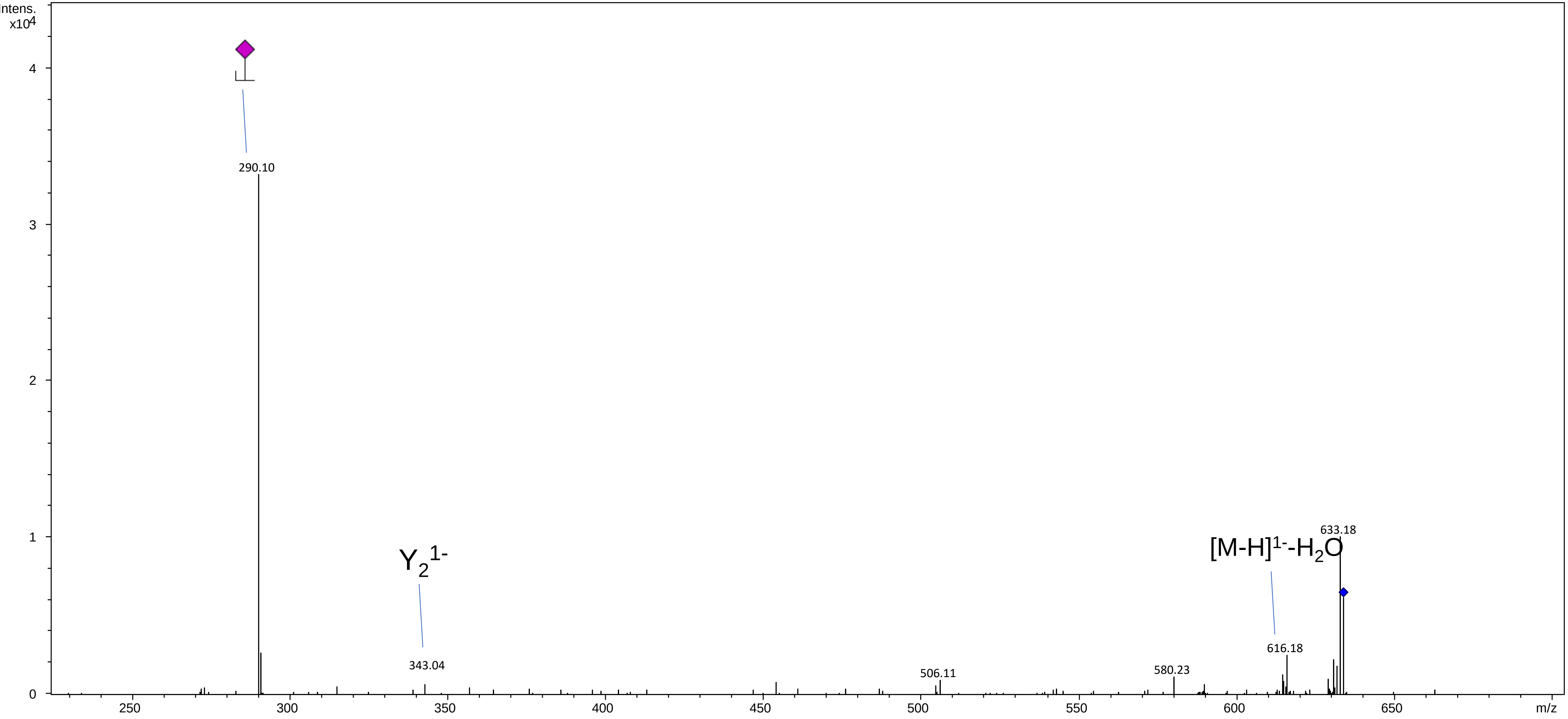

# Glycan 4

GM2
H2N1S1

| | |
|---|---|
| **Monoisotopic mass:** | **838.30 Da** |
| **Charge observed:** | **1-** |
| **Theoretical ion:** | ***m/z* 837.30** |
| **Observed ion:** | ***m/z* 837.32** |
| **Mass deviation:** | ***m/z* 0.02** |
| **Retention time:** | **21.7 min** |

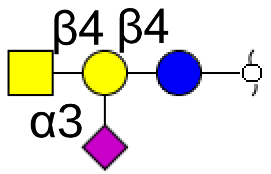


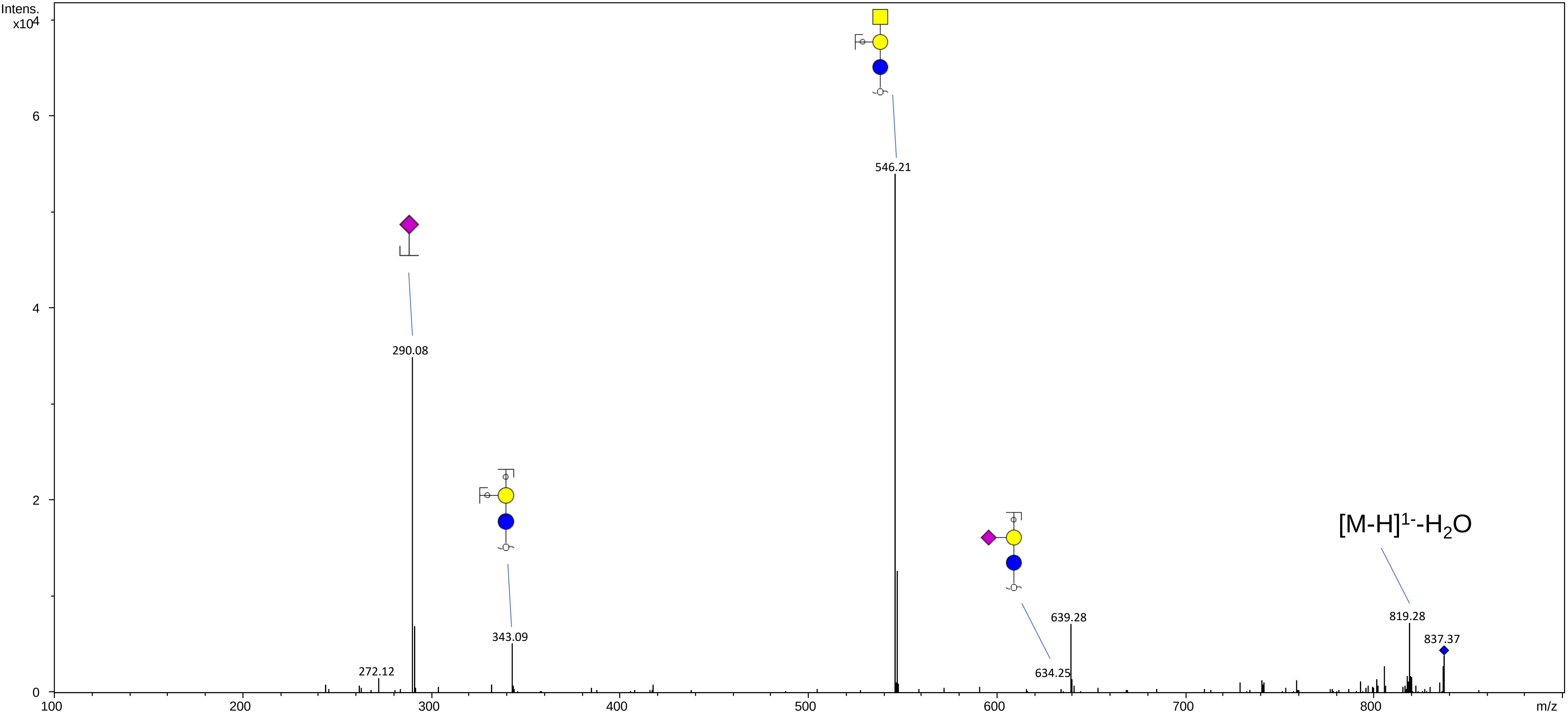

# Glycan 5

## GM1a
## H3N1S1

| | |
|---|---|
| **Monoisotopic mass:** | **1000.35 Da** |
| **Charge observed:** | **1-** |
| **Theoretical ion:** | ***m/z* 999.35** |
| **Observed ion:** | ***m/z* 999.35** |
| **Mass deviation:** | ***m/z* 0.00** |
| **Retention time:** | **23.5 min** |

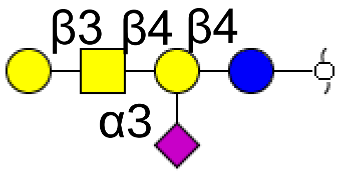


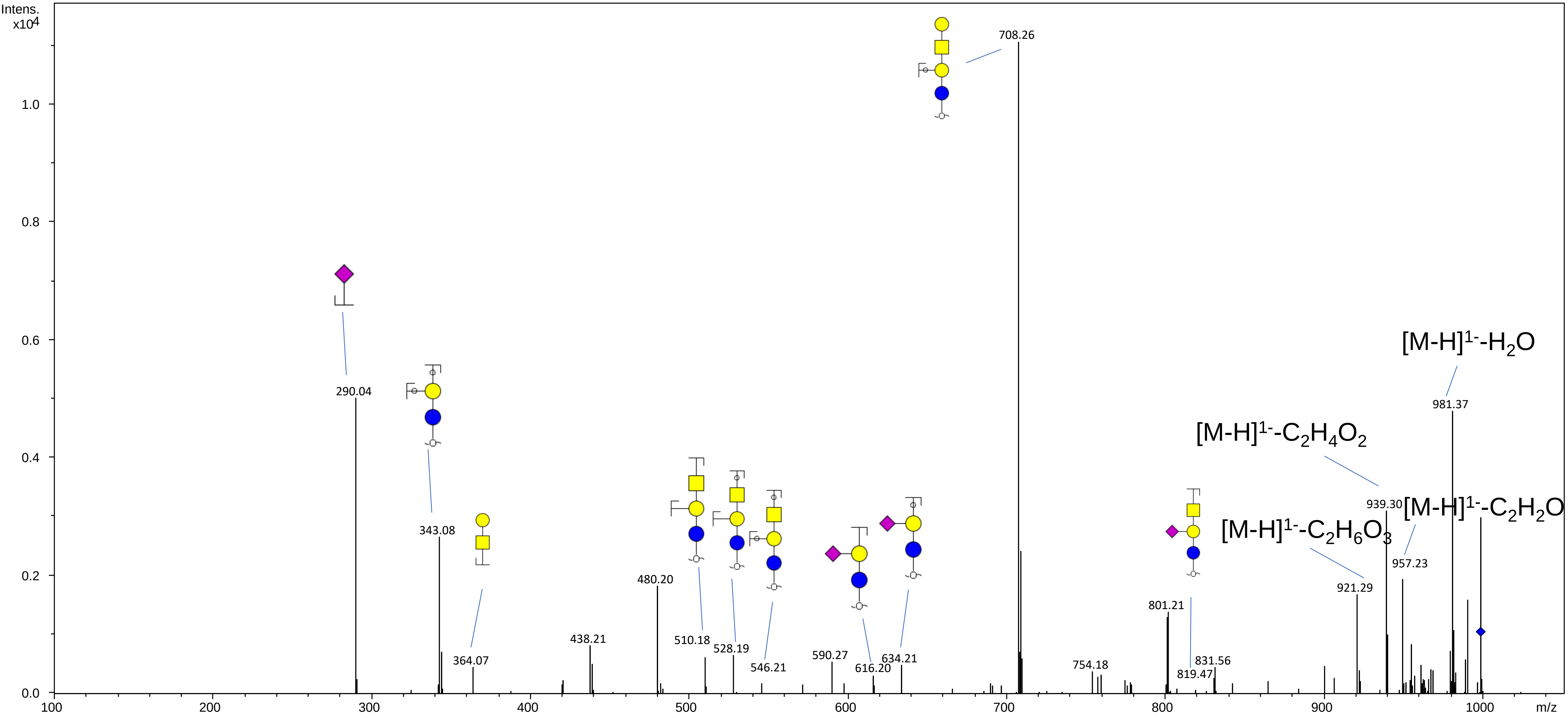

# Glycan 6

## GD1a
## H3N1S2

| | |
|---|---|
| **Monoisotopic mass:** | **1291.48 Da** |
| **Charge observed:** | **2-** |
| **Theoretical ion:** | **644.74 *m/z*** |
| **Observed ion:** | **644.73 *m/z*** |
| **Mass deviation:** | **0.01 *m/z*** |
| **Retention time:** | **23.5 min** |

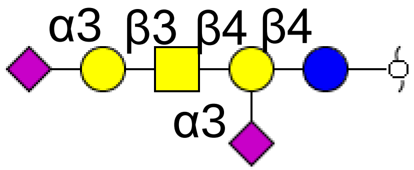


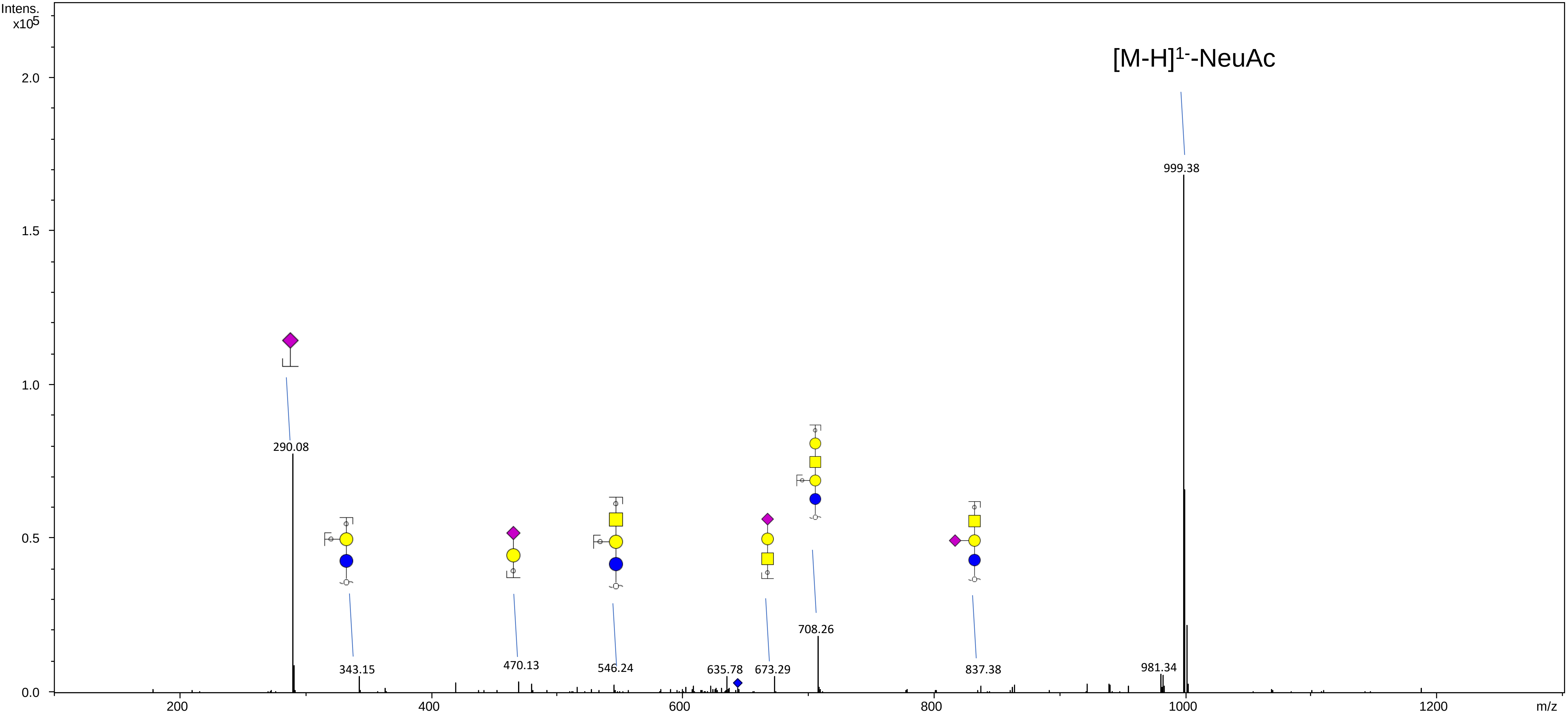

# Glycan 7

(n)Lc4
H3N1

| | |
|---|---|
| **Monoisotopic mass:** | **709.26 Da** |
| **Charge observed:** | **1-** |
| **Theoretical ion:** | **708.26 *m/z*** |
| **Observed ion:** | **708.28 *m/z*** |
| **Mass deviation:** | **0.02 *m/z*** |
| **Retention time:** | **40.1 min** |

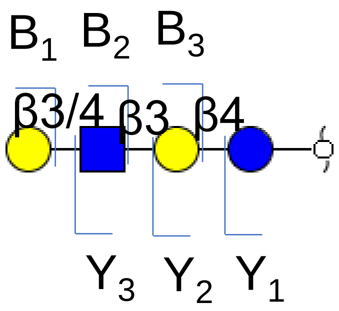


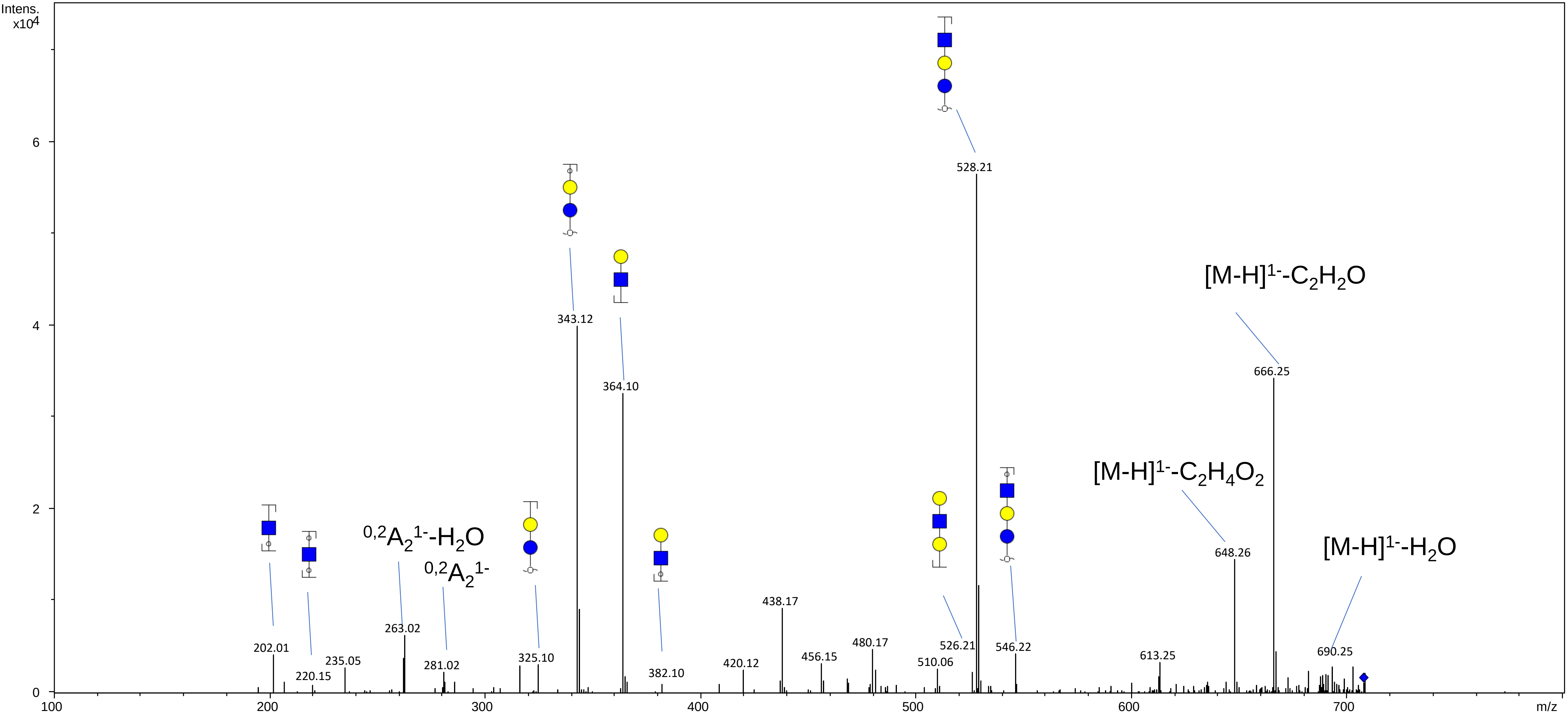

# Glycan 8

## H3N1S1a

| | |
|---|---|
| **Monoisotopic mass:** | **1000.35 Da** |
| **Charge observed:** | **1-** |
| **Theoretical ion:** | ***m/z* 999.35** |
| **Observed ion:** | ***m/z* 999.39** |
| **Mass deviation:** | ***m/z* 0.04** |
| **Retention time:** | **46.9 min** |

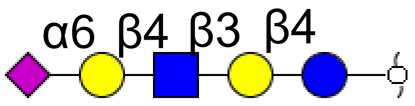


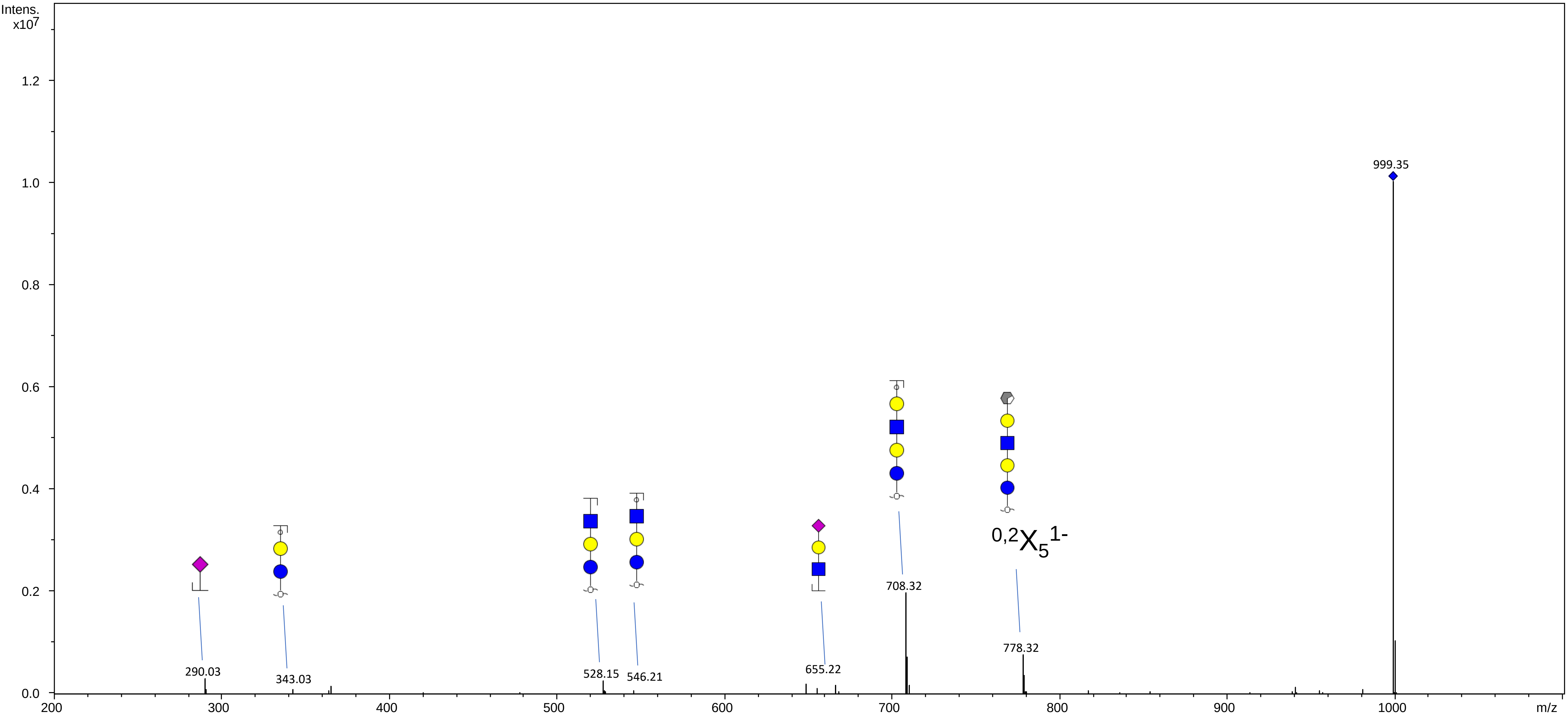

# Glycan 9

**Monoisotopic mass:** 1000.35 Da
**Charge observed:** 1-
**Theoretical ion:** *m/z* 999.35
**Observed ion:** *m/z* 999.37
**Mass deviation:** *m/z* 0.02
**Retention time:** 48.0 min

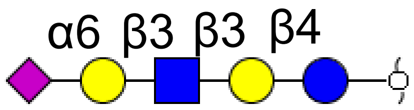


## H3N1S1b

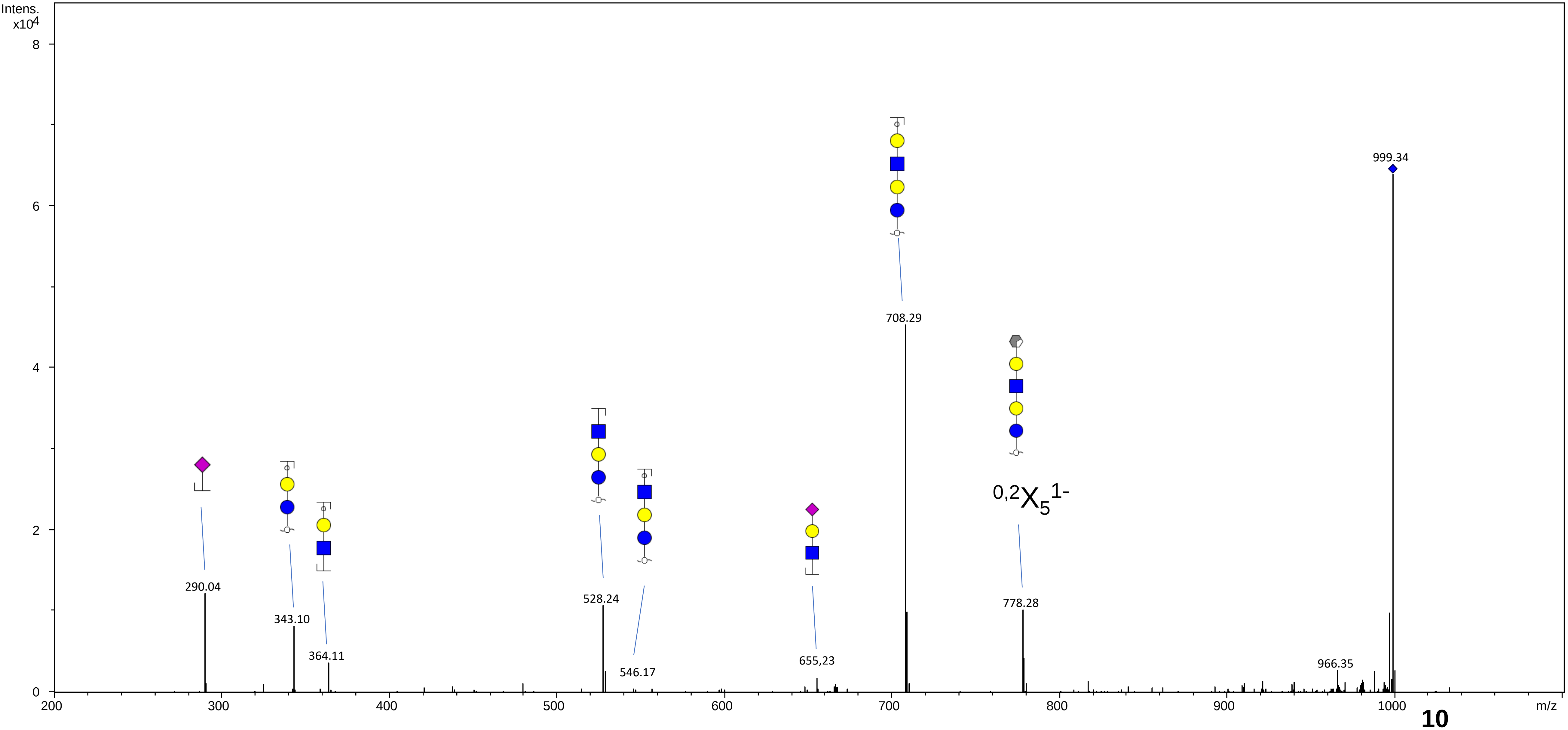

# Glycan 10

**Monoisotopic mass:** **999.35 Da**
**Charge observed:** **1-**
**Theoretical ion:** **999.35 *m/z***
**Observed ion:** **999.37 *m/z***
**Mass deviation:** **0.00 *m/z***
**Retention time:** **53.0 min**

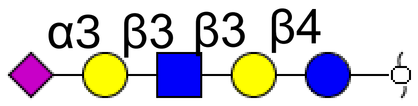


H3N1S1c

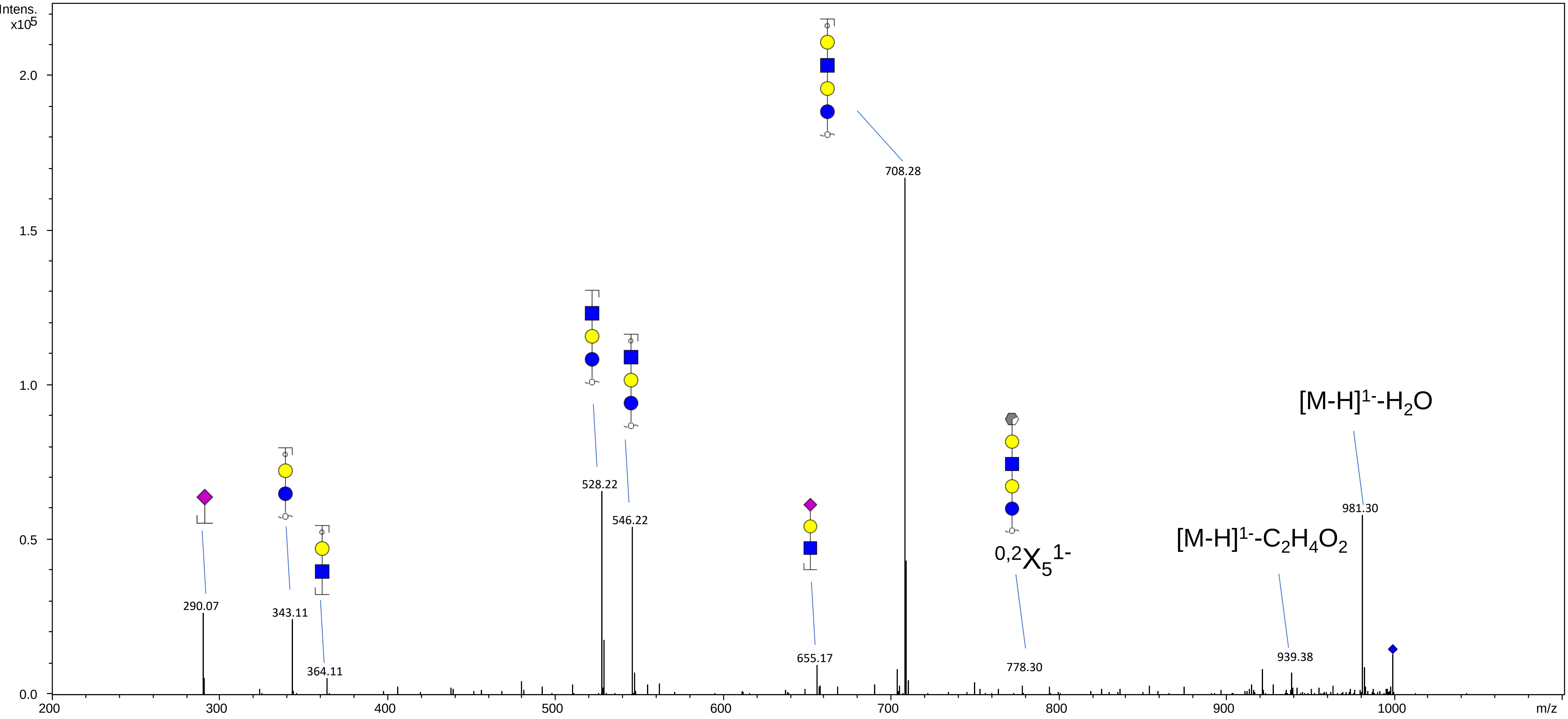

# Glycan 11

**Monoisotopic mass:** **1000.35 Da**
**Charge observed:** **1-**
**Theoretical ion:** ***m/z* 999.35**
**Observed ion:** ***m/z* 999.37**
**Mass deviation:** ***m/z* 0.02**
**Retention time:** **58.8 min**

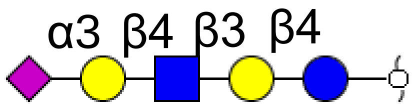


H3N1S1d

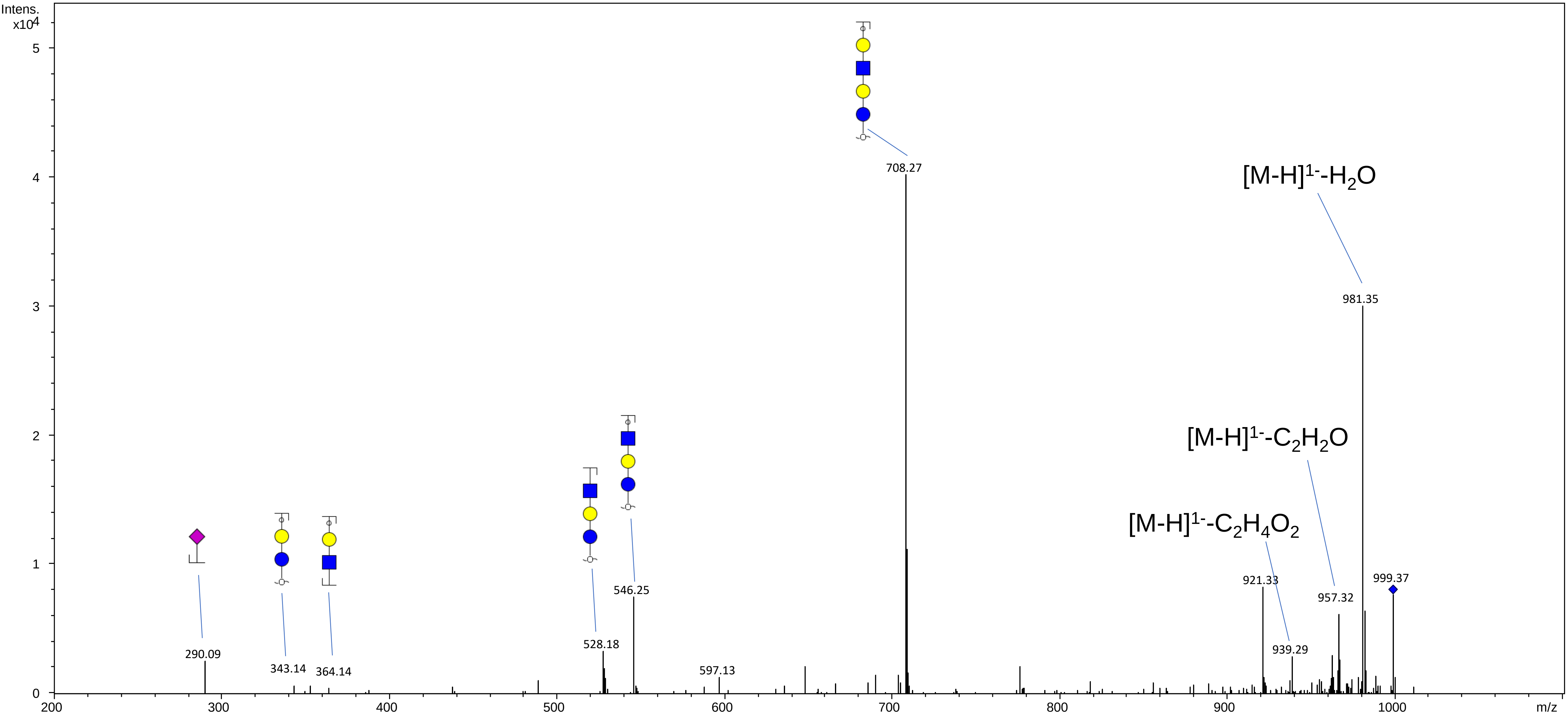

# Glycan 12

H3N2F1

**Monoisotopic mass:** 1058.39 Da
**Charge observed:** 1-
**Theoretical ion:** *m/z* 1057.39
**Observed ion:** *m/z* 1057.43
**Mass deviation:** *m/z* 0.04
**Retention time:** 31.8 min

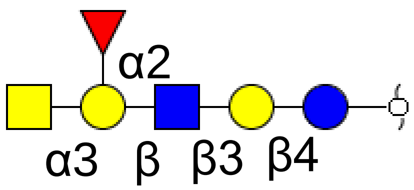


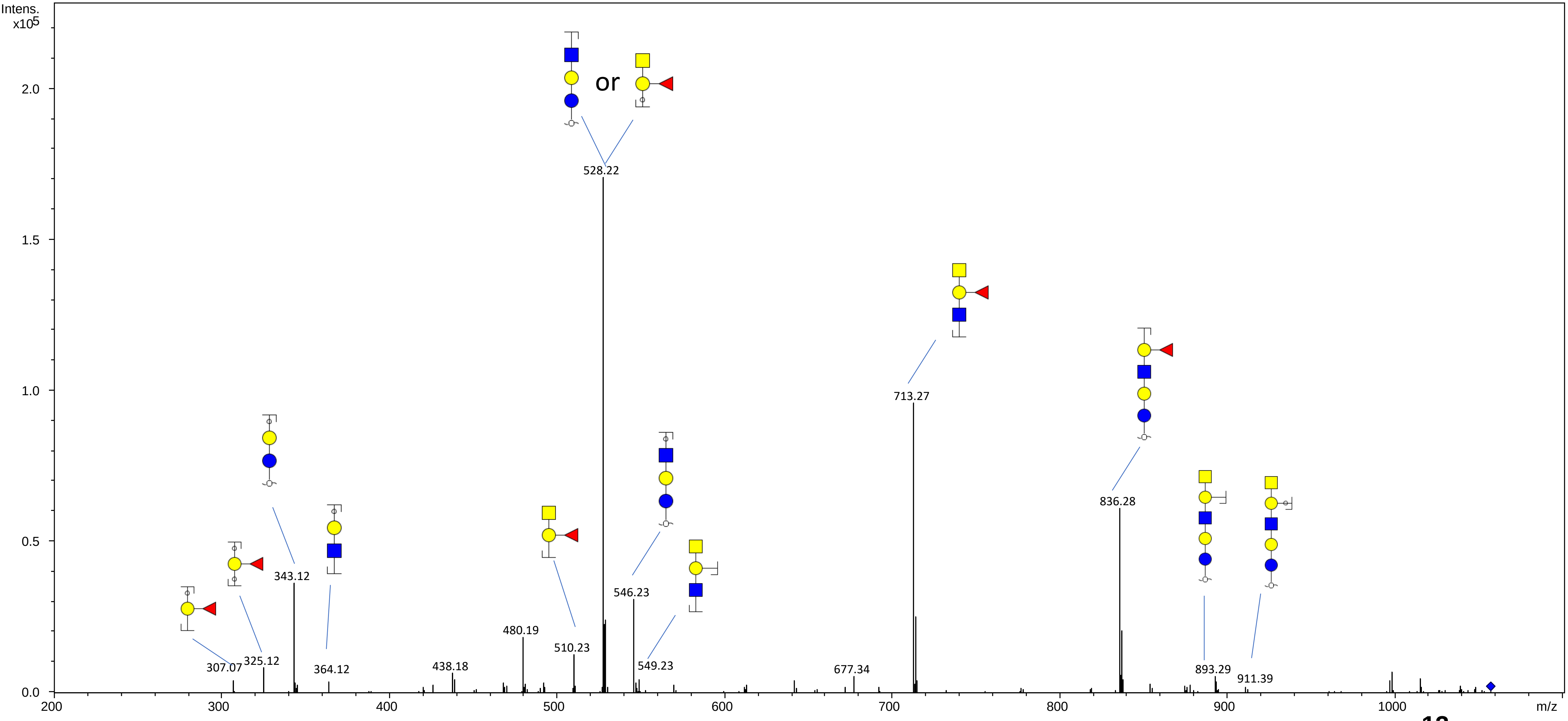

# Glycan 13

| | |
|---|---|
| **Monoisotopic mass:** | **1423.53 Da** |
| **Charge observed:** | **1-** |
| **Theoretical ion:** | ***m/z* 1422.53** |
| **Observed ion:** | ***m/z* 1422.55** |
| **Mass deviation:** | ***m/z* 0.02** |
| **Retention time:** | **41.6 min** |

## H4N3F1

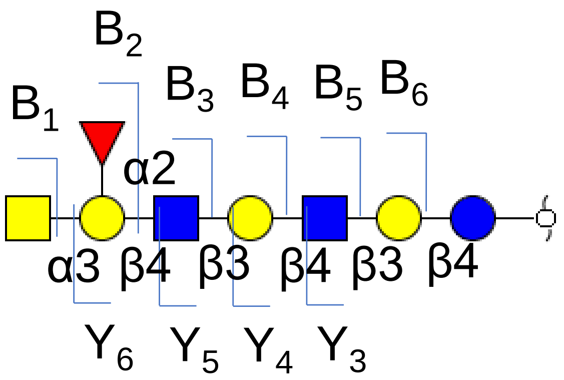


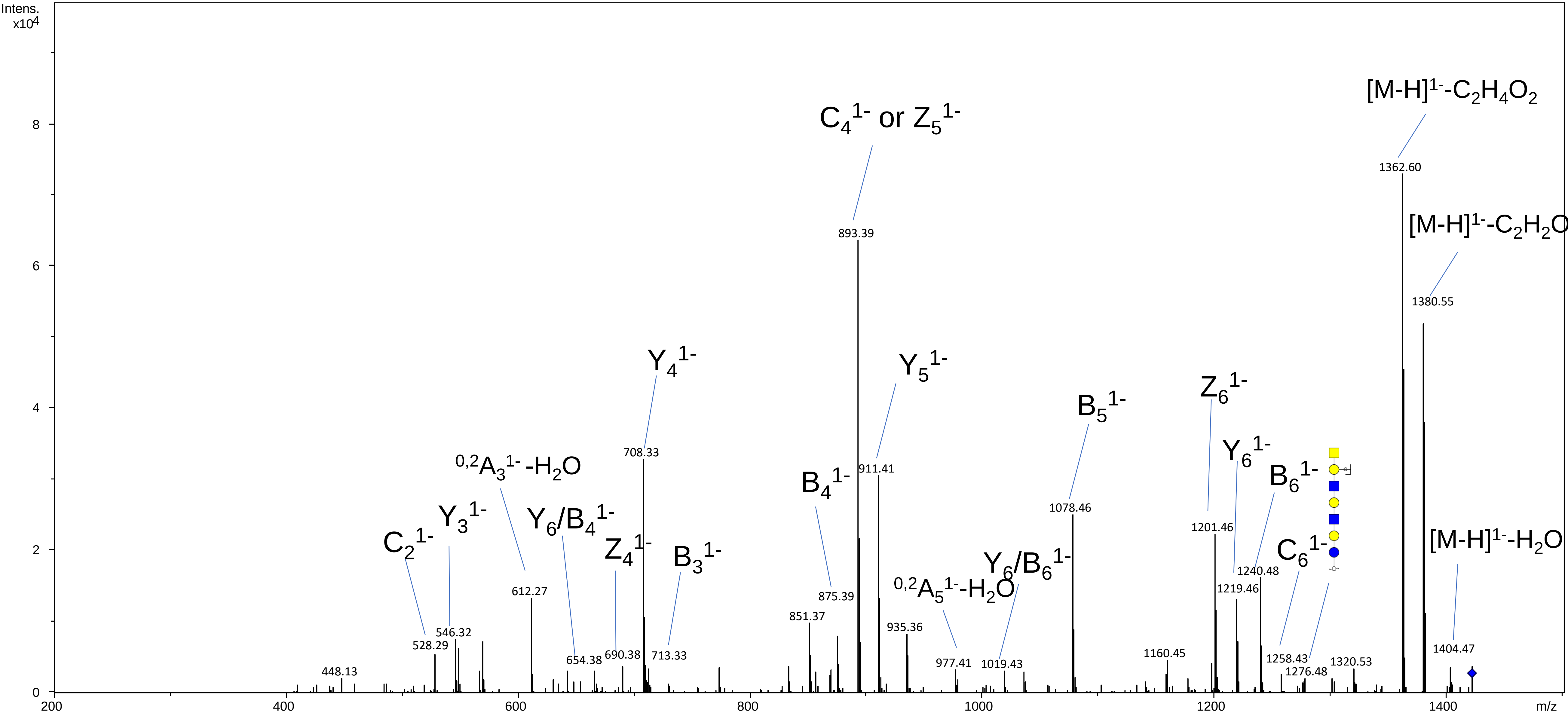

# Glycan 14

## H4N2S1a

| | |
|---|---|
| **Monoisotopic mass:** | **1365.48 Da** |
| **Charge observed:** | **1-** |
| **Theoretical ion:** | ***m/z* 1364.48** |
| **Observed ion:** | ***m/z* 1364.56** |
| **Mass deviation:** | ***m/z* 0.08** |
| **Retention time:** | **53.7 min** |

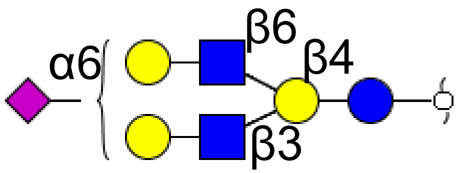


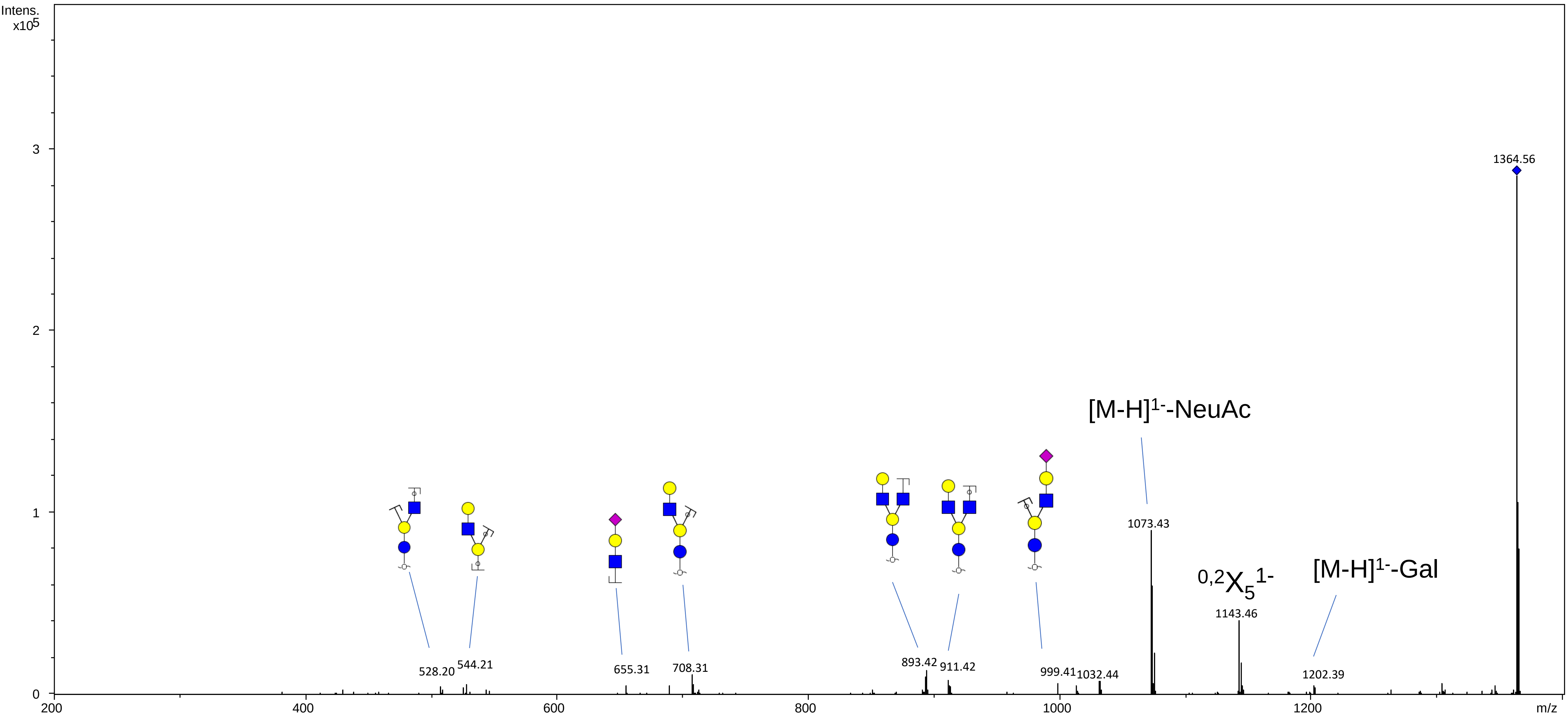

# Glycan 15

**Monoisotopic mass:** 1365.48 Da
**Charge observed:** 1-
**Theoretical ion:** *m/z* 1364.48
**Observed ion:** *m/z* 1364.52
**Mass deviation:** *m/z* 0.04
**Retention time:** 61.4 min

## H4N2S1b

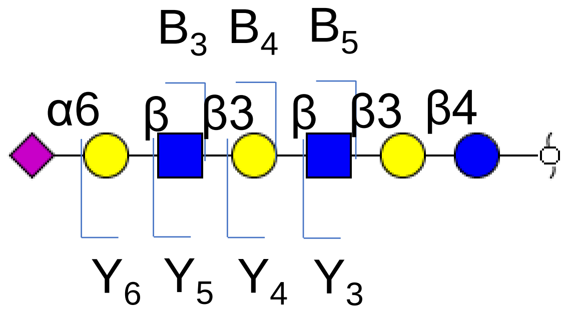


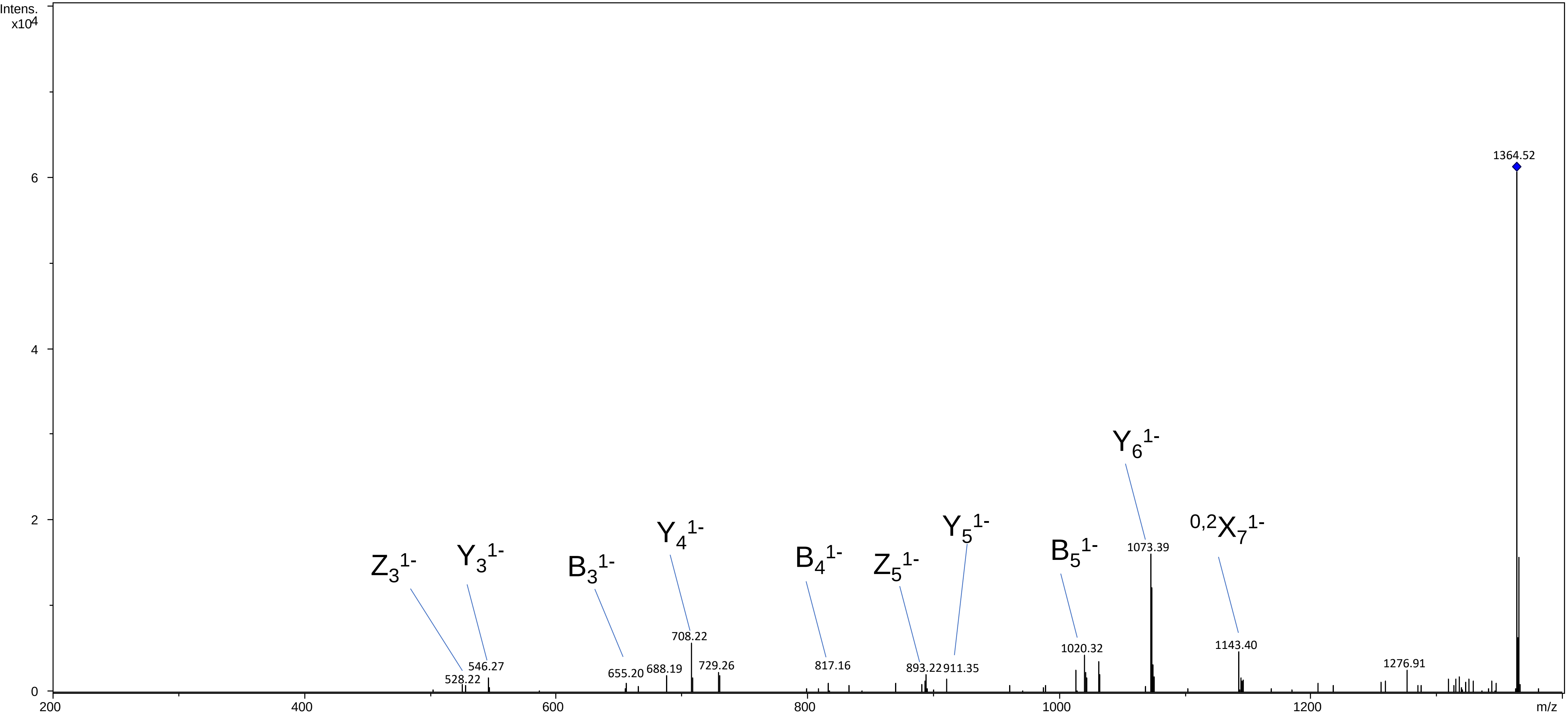

# Glycan 16

H4N2

**Monoisotopic mass:** 1074.39 Da
**Charge observed:** 1-
**Theoretical ion:** 1073.39*m/z*
**Observed ion:** 1073.36 *m/z*
**Mass deviation:** 0.03*m/z*
**Retention time:** 61.4 min

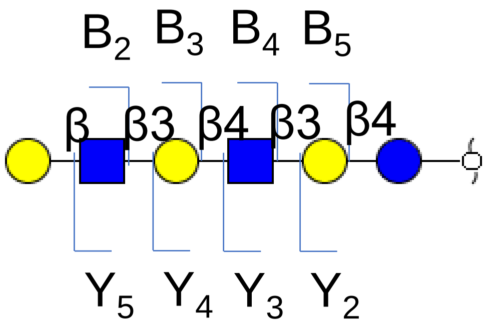


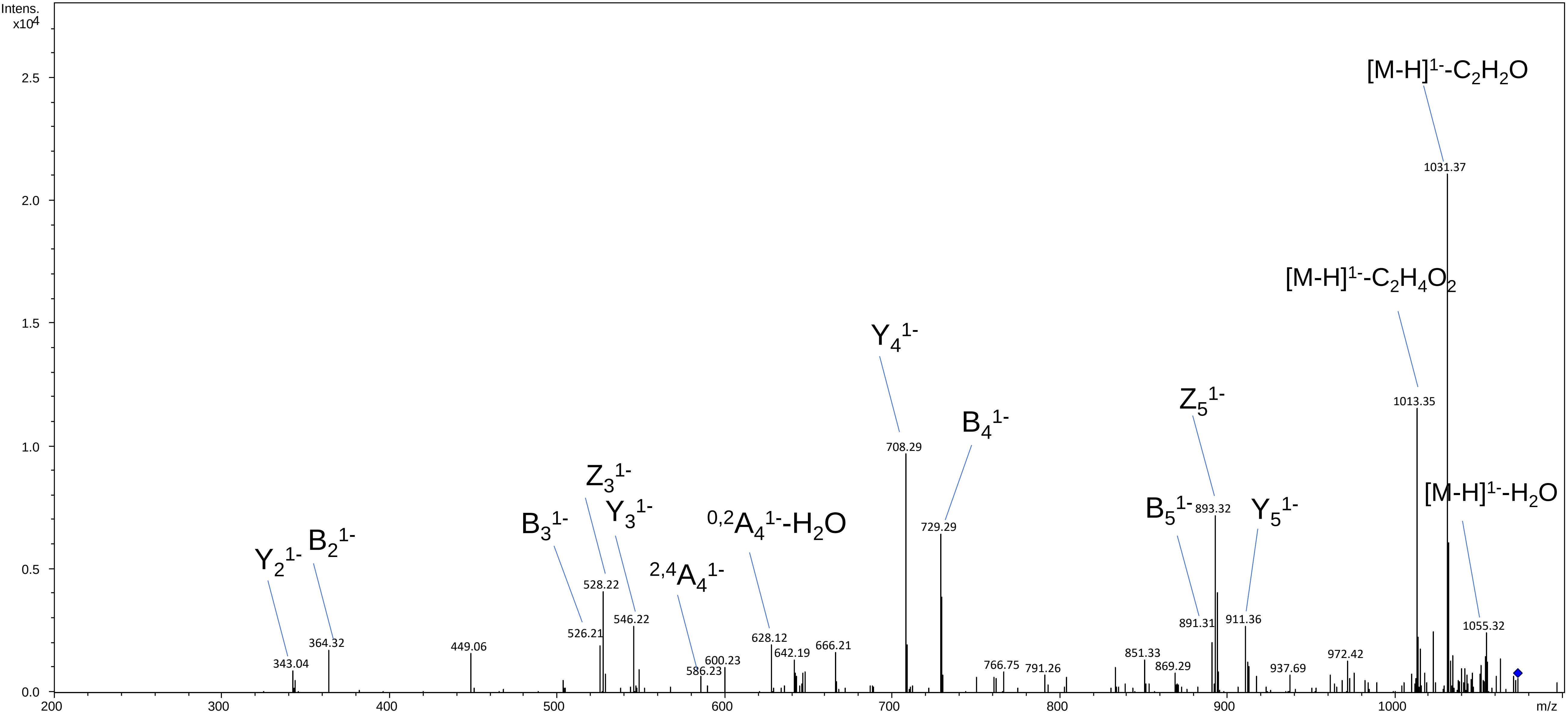

# Glycan 17

H5N4F1S1

| | |
|---|---|
| **Monoisotopic mass:** | **2079.75 Da** |
| **Charge observed:** | **2-** |
| **Theoretical ion:** | ***m/z* 1038.87** |
| **Observed ion:** | ***m/z* 1038.88** |
| **Mass deviation:** | ***m/z* 0.01** |
| **Retention time:** | **58.2 min** |

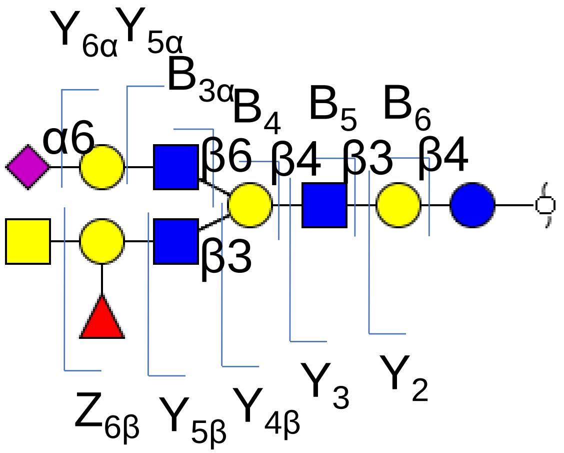


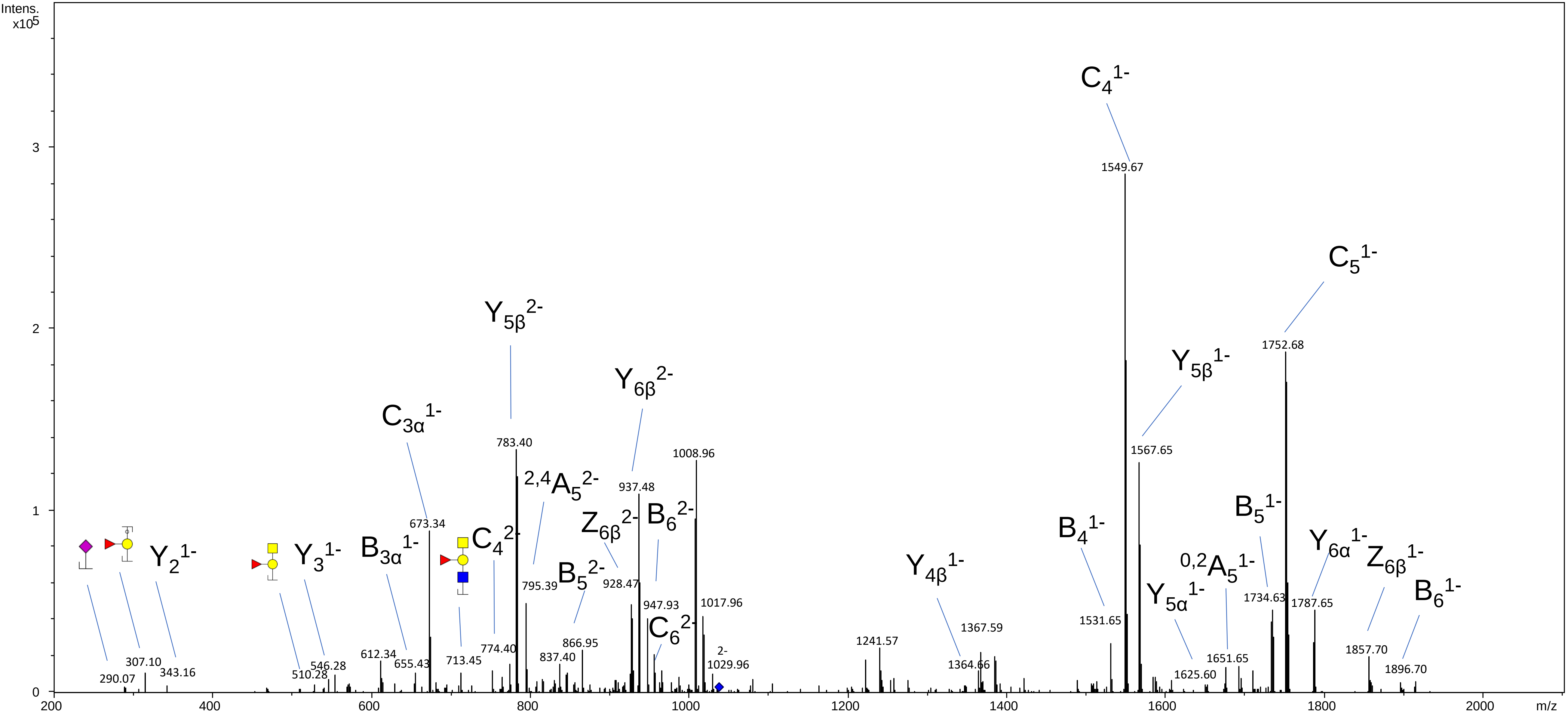

# Glycan 18

| | |
|---|---|
| **Monoisotopic mass:** | **1730.64 Da** |
| **Charge observed:** | **2-** |
| **Theoretical ion:** | ***m/z* 864.32** |
| **Observed ion:** | ***m/z* 864.32** |
| **Mass deviation:** | ***m/z* 0.00** |
| **Retention time:** | **63.7 min** |

## H5N3S1a

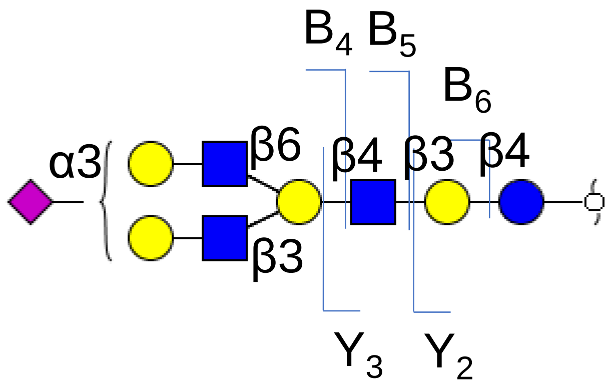


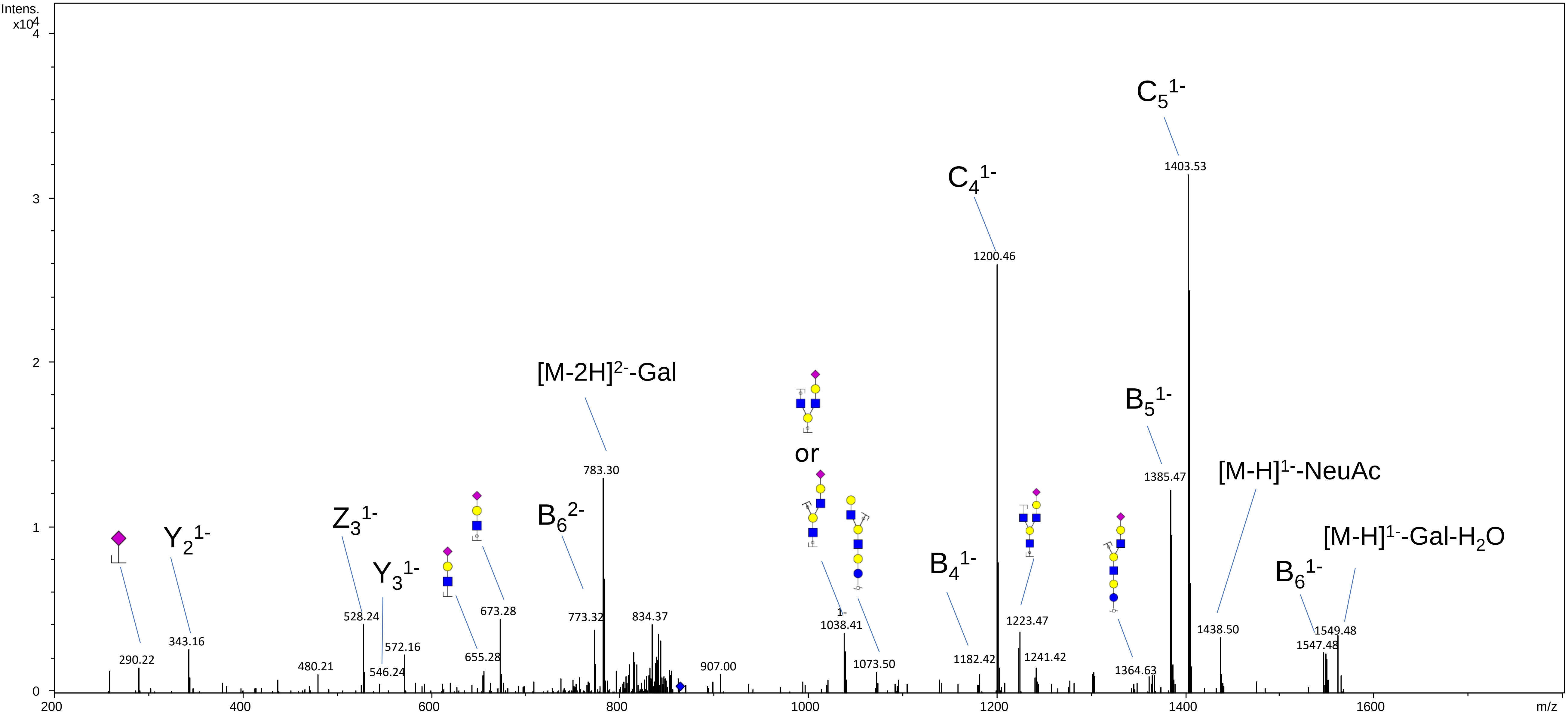

# Glycan 19

H5N3S1b

**Monoisotopic mass:** 1730.64 Da
**Charge observed:** 2-
**Theoretical ion:** *m/z* 864.32
**Observed ion:** *m/z* 864.32
**Mass deviation:** *m/z* 0.00
**Retention time:** 64.5 min

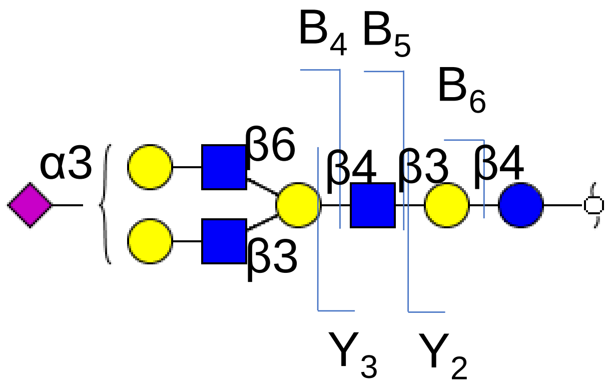


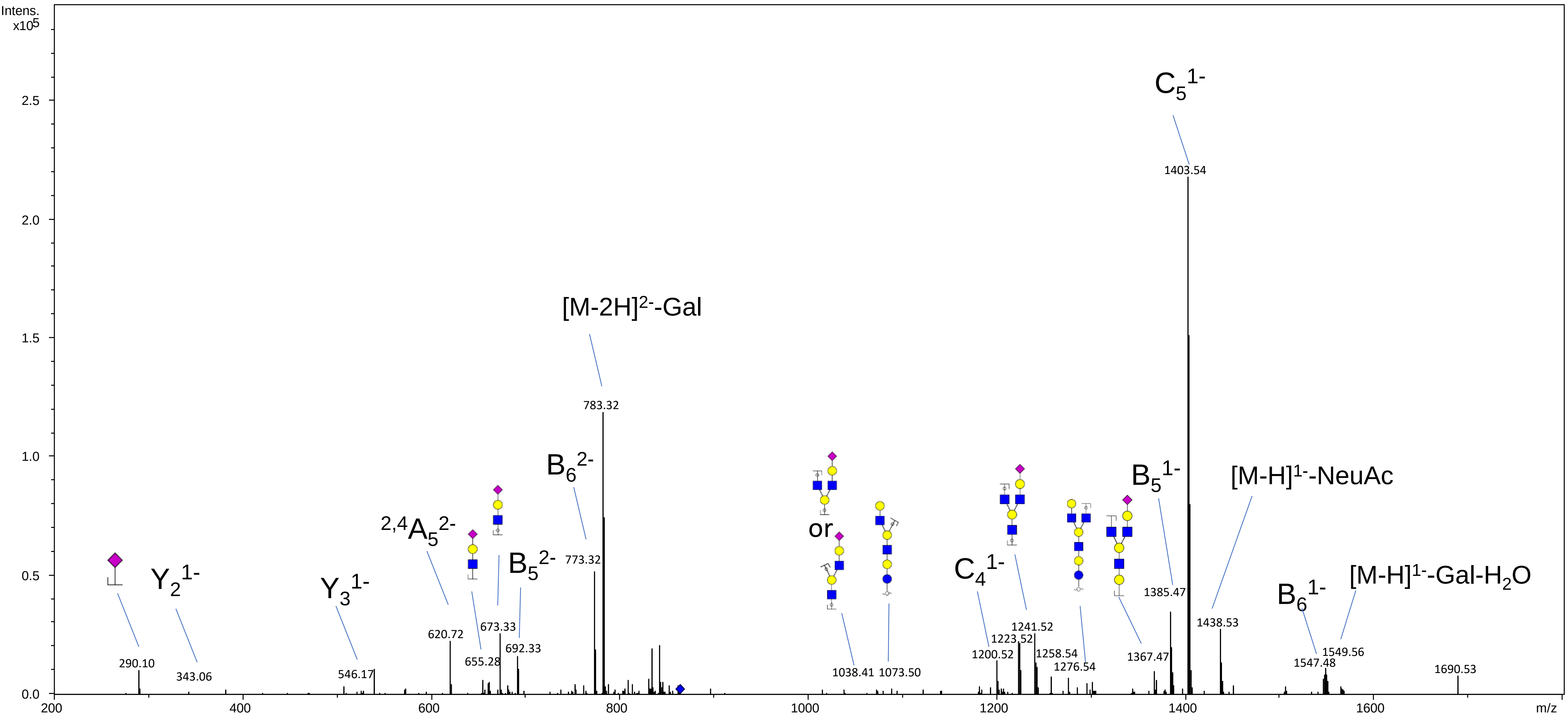

# Glycan 20

## H5N3S2a

**Monoisotopic mass:** **2021.71 Da**
**Charge observed:** **2-**
**Theoretical ion:** ***m/z* 1009.86**
**Observed ion:** ***m/z* 1009.84**
**Mass deviation:** ***m/z* 0.02**
**Retention time:** **67.2 min**

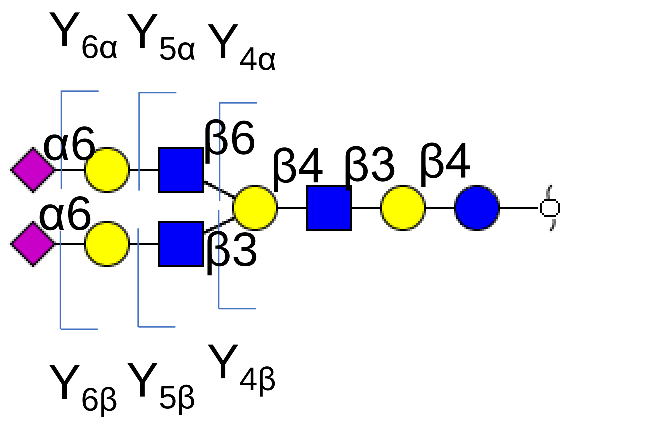


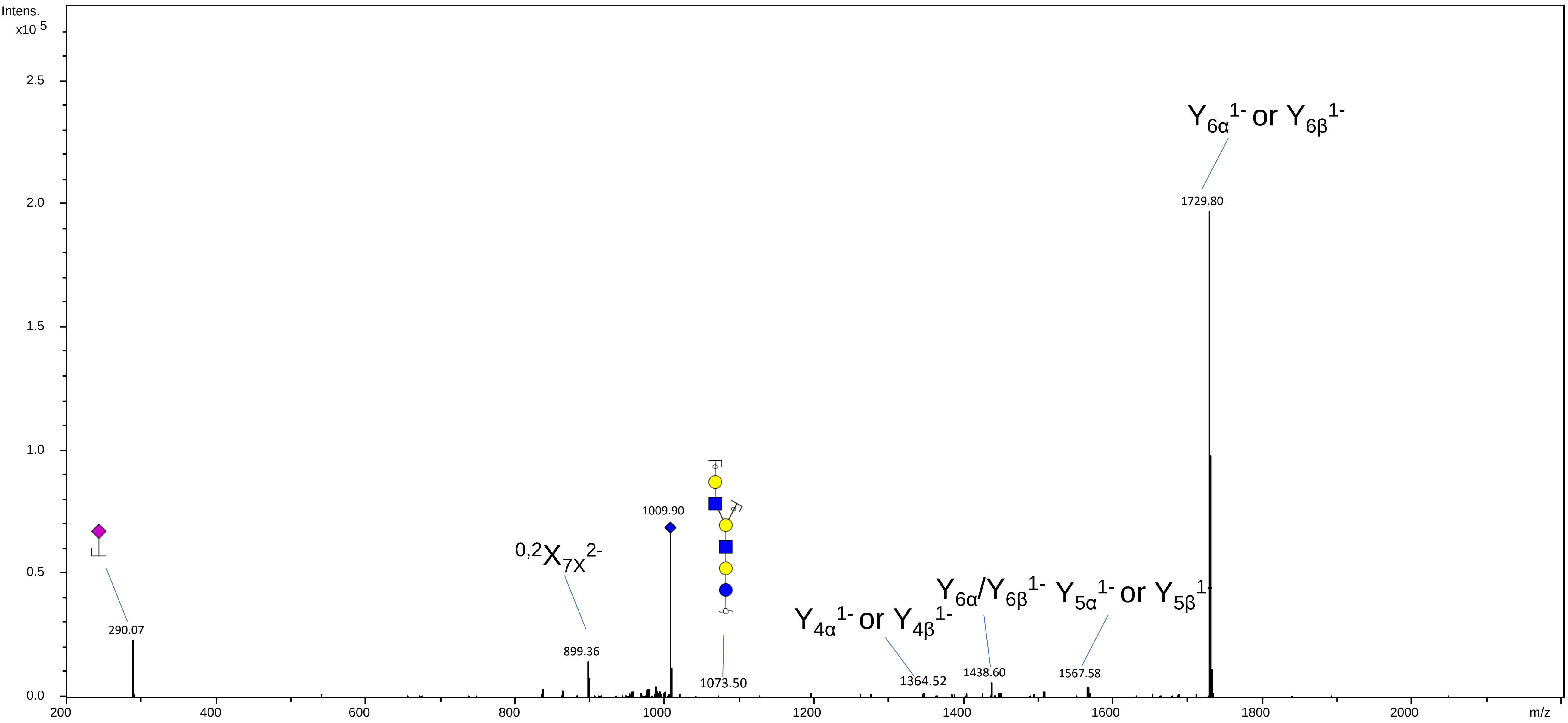

# Glycan 21

## H5N3S2b

**Monoisotopic mass:** 2021.71 Da
**Charge observed:** 2-
**Theoretical ion:** *m/z* 1009.86
**Observed ion:** *m/z* 1009.84
**Mass deviation:** *m/z* 0.02
**Retention time:** 67.2 min

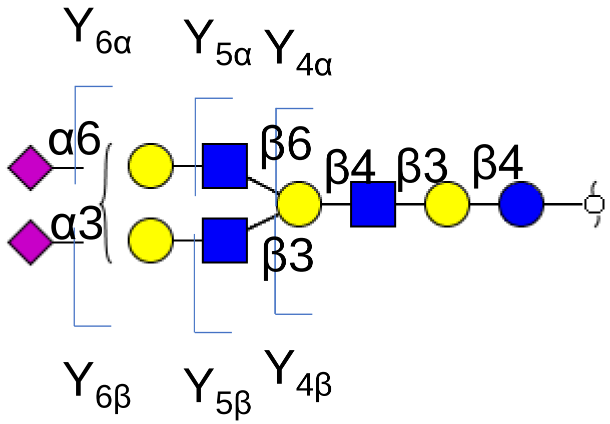


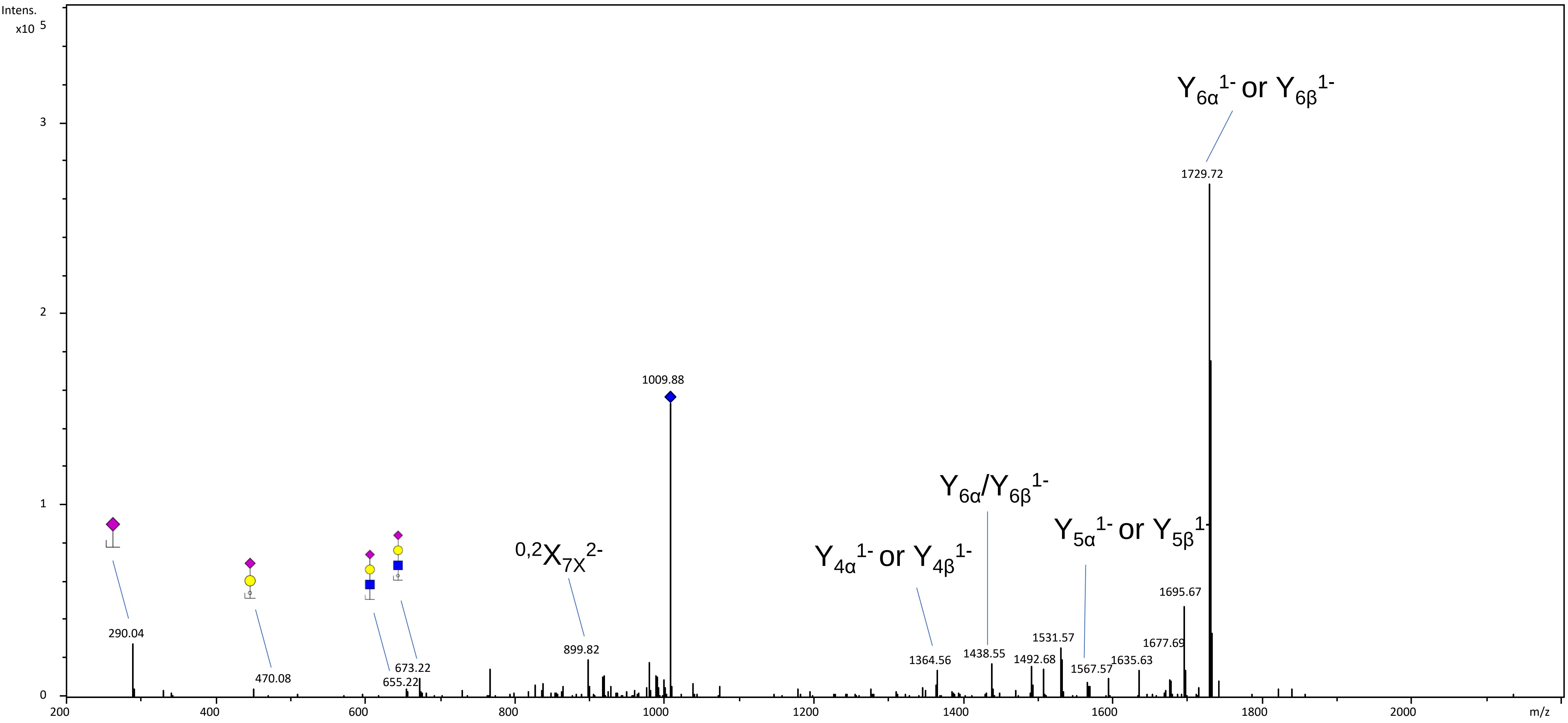

# Glycan 22

H5N3F1S1

| | |
|---|---|
| **Monoisotopic mass:** | **1876.67 Da** |
| **Charge observed:** | **2-** |
| **Theoretical ion:** | ***m/z* 937.34** |
| **Observed ion:** | ***m/z* 937.32** |
| **Mass deviation:** | ***m/z* 0.02** |
| **Retention time:** | **70.6 min** |

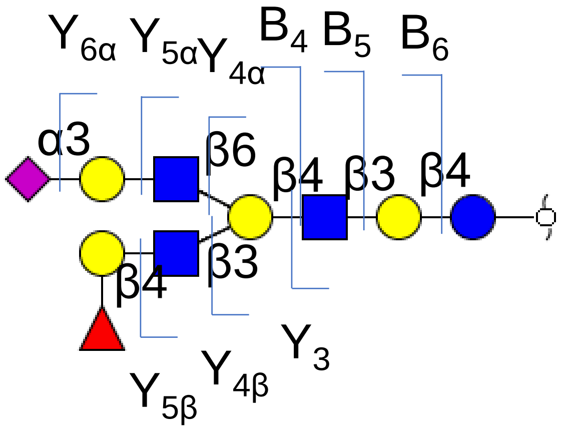


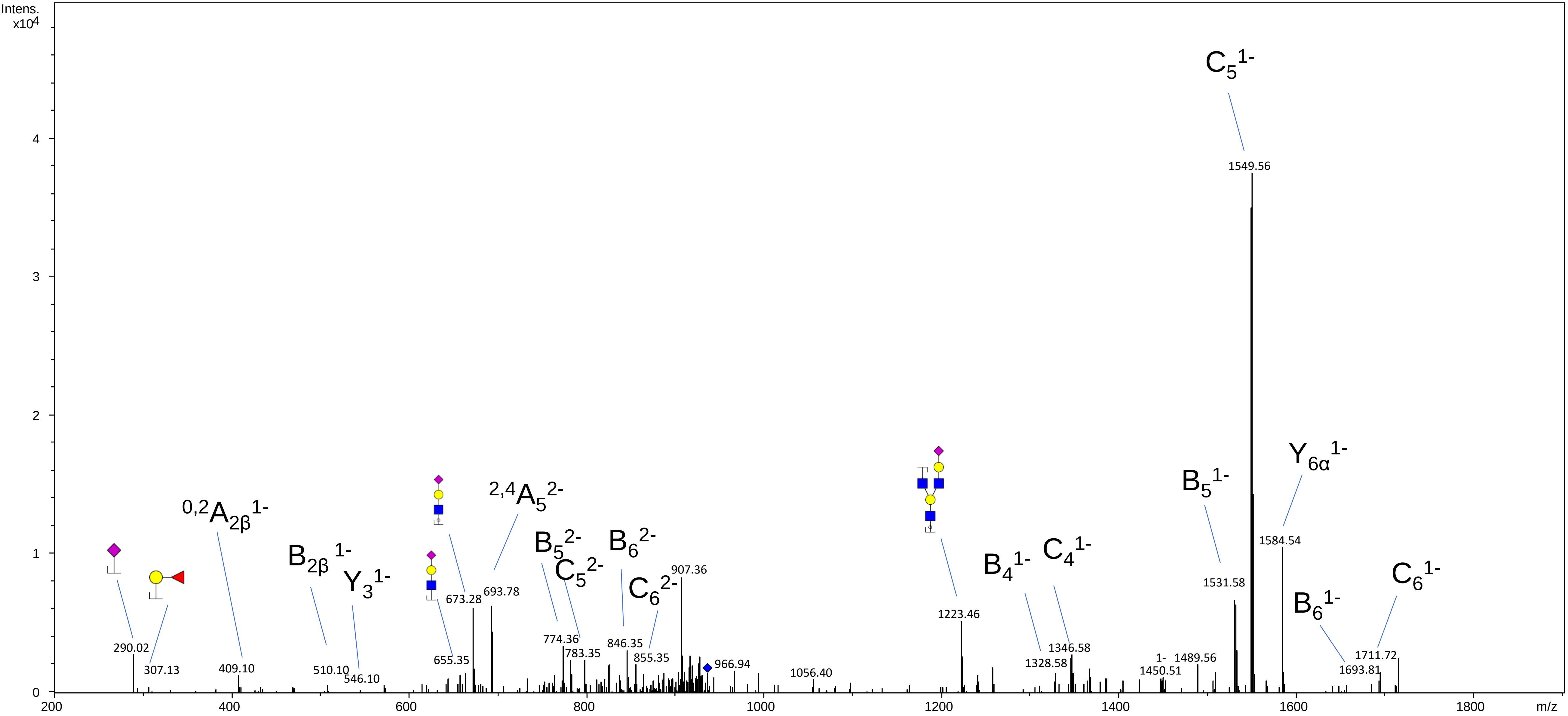

Supplement: Supplemental Figure S12 [file mmc4.pdf]
